# Antiviral and Cytotoxic Activity of Different Plant Parts of Banana (*Musa* *spp*.)

**DOI:** 10.3390/v12050549

**Published:** 2020-05-15

**Authors:** Sujogya Kumar Panda, Ana Hortência Fonsêca Castro, Ramin Saleh Jouneghani, Pieter Leyssen, Johan Neyts, Rony Swennen, Walter Luyten

**Affiliations:** 1 Department of Biology, Katholieke Universiteit Leuven, 3000 Leuven, Belgium; acastro905@gmail.com (A.H.F.C.); r.saleh.j@gmail.com (R.S.J.); walter.luyten@kuleuven.be (W.L.); 2 Mayurbhanj Biological Research (MBR), Bhanjpur, Baripada 757002, Odisha, India; 3 Plant Physiology and Biochemistry, Universidade Federal de São João Del-Rei, Av. Sebastião Gonçalves Coelho, 400–Chanandour, Divinópolis MG 35501-296, Brazil; 4 Rega Institute for Medical Research, Laboratory of Virology and Chemotherapy, Katholieke Universiteit Leuven, 3000 Leuven, Belgium; pieter.leyssen@kuleuven.be (P.L.); johan.neyts@kuleuven.be (J.N.); 5 International Institute of Tropical Agriculture, Arusha P.O. Box 447, Tanzania; rony.swennen@kuleuven.be; 6 Laboratory of Tropical Crop Improvement, Division of Crop Biotechnics, Katholieke Universiteit Leuven, 3001 Leuven, Belgium; 7 Bioversity International, 3001 Leuven, Belgium

**Keywords:** banana plant extracts, Chikungunya virus, cytotoxicity, enterovirus 71, yellow fever virus

## Abstract

Chikungunya and yellow fever virus cause vector-borne viral diseases in humans. There is currently no specific antiviral drug for either of these diseases. Banana plants are used in traditional medicine for treating viral diseases such as measles and chickenpox. Therefore, we tested selected banana cultivars for their antiviral but also cytotoxic properties. Different parts such as leaf, pseudostem and corm, collected separately and extracted with four different solvents (hexane, acetone, ethanol, and water), were tested for in vitro antiviral activity against Chikungunya virus (CHIKV), enterovirus 71 (EV71), and yellow fever virus (YFV). Extracts prepared with acetone and ethanol from leaf parts of several cultivars exhibited strong (EC_50_ around 10 μg/mL) anti-CHIKV activity. Interestingly, none of the banana plant extracts (concentration 1–100 µg/mL) were active against EV71. Activity against YFV was restricted to two cultivars: Namwa Khom–Pseudostem–Ethanol (5.9 ± 5.4), Namwa Khom–Corm–Ethanol (0.79 ± 0.1) and Fougamou–Corm–Acetone (2.5 ± 1.5). In most cases, the cytotoxic activity of the extracts was generally 5- to 10-fold lower than the antiviral activity, suggesting a reasonable therapeutic window.

## 1. Introduction

Banana (*Musa* spp.) is a perennial herb that produces the second-most important fruit after citrus. Currently, worldwide banana production is over 144 million tonnes annually and includes dessert and cooking bananas [1]. More than 1000 genotypes exist, derived from intra- or inter-specific hybridizations of the wild diploid (2n = 2x = 22 chromosomes) ancestral species *M. acuminata* Colla (A genome) and *M. balbisiana* Colla (B genome) [2]. The edible bananas are parthenocarpic, with the following groups: diploids (AA, AB, possibly BB), triploids (AAA, AAB, ABB, possibly BBB), or tetraploids (AAAA, AAAB, AABB, ABBB). Sweet bananas are widely cultivated on all continents except Antarctica. They include AA (Pisang Mas-Amas, Kluai Khai, Bocadillo, Figue sucree, sucrier), AAA (Cavendish-Giant and Dwarf, Grande Naine, Poyo, Robusta), and AAB (Silk, Mysore-Inangel, Pisang Keling, Prata, Pacovan, Prata Ana). Cooking bananas are also important and belong to the ABB group and possibly BBB. Important cultivars are Saba, Cachaco, Pisang Awak, Pelipita, and Cardaba [3]. There is ample evidence that banana plants contain (poly)phenolic compounds [4] and carotenoids (α-carotene, *trans*-β-carotene, lutein, 13-*cis*-β-carotene and 9-*cis*-β-carotene) [5,6], which are useful in the treatment of multiple diseases [7]. The banana pulp and peel contain carbohydrates, minerals (K, P, Ca, Mg, Mn and Zn), antioxidants, vitamins (C, A and E), catecholamines, as well as pyridoxine [6,7,8].

Plantains as well as dessert bananas, and the other parts of the *Musa* spp. plant, which include roots, corm (i.e., underground stem), pseudostem (i.e., aboveground false stem), leaves, flowers, and peels, have long been used in traditional medicine around the world to treat fevers, burns, liver problems, diarrhea, inflammation, pain, snakebite, and diabetes [9,10,11]. Ethnopharmacological studies have documented several traditional uses of bananas, and different plant parts (flower, leaves, pseudostem, corm, fruit pulp and peels) have been studied for their anti-ulcerogenic [12], hypolipidemic [13], hypoglycemic [14], and wound-healing activity [15]. BanLec, a jacalin-related lectin, was found to be a potent inhibitor of HIV replication [16]. Later, Swanson and coworkers demonstrated that “a single amino-acid substitution in a banana lectin, replacing histidine84 with threonine, significantly reduces its mitogenicity, while preserving its broad-spectrum antiviral potency” [17]. Except for that study, to the best of our knowledge, no scientific study on antiviral properties of *Musa* has been reported. Therefore, we studied the antiviral (and also cytotoxic) properties of different parts of 10 dessert or cooking banana plants.

## 2. Materials and Methods

### 2.1. Collection of Samples

Leaves, pseudostems and corms of 10 adult banana cultivars were collected in March 2015 from the tropical greenhouses, KU Leuven, Heverlee Campus (Leuven Belgium), and one variety was obtained from Africa (Table 1). The greenhouse plants were grown in DCM pot soil type 7.

### 2.2. Extraction Preparation

The plant parts (e.g., leaves, pseudostems and corms) were cut into small slices and dried in an oven at 70 °C. All samples were then ground to a fine powder using a high-powered HK-10B plant mill (Guangzhou Xulang Machinery & Equipment Co. Ltd., Guangzhou, China). Afterwards, the powdered samples were stored completely dried in a cold room at 4 °C to avoid growth of fungi, molds, bacteria or other microorganisms [19]. One gram of the fine plant powder was extracted in 15 mL conical Falcon tubes with screwcaps using 10 mL of four different solvents (hexane, acetone, ethanol, and water) at ambient temperature with the aid of sonication (4 × 15 min over a 24 h period) in a water bath (Branson) and repeated vortexing. After 1 day, the tubes were centrifuged for 10 min at 3500× *g*, and the supernatant transferred in 1 mL aliquots to tubes (Abgene™ 2D barcoded 2mL screw cap storage tubes, Thermo Scientific™, Geel, Belgium). After evaporation of water and ethanol in a Savant SpeedVac Concentrator (Thermo Scientific™, Geel, Belgium), and acetone and hexane evaporation at ambient temperature in a chemical fume hood, the dry weight of each sample was determined. A final stock concentration in DMSO (10 mg/mL) of each sample was prepared after drying. The samples were stored at 4 °C until further testing.

### 2.3. Antiviral Test

Antiviral activity was tested as described earlier [20,21,22]. Three different viruses were used: Chikungunya virus (CHIKV) (899 strain) propagated in Vero cells subtype A, enterovirus 71 (EV71) (BRCR strain) cultured in human rhabdomyosarcoma (RD) cells, and yellow fever virus (YFV) (17D Stamari l strain) cultured in human liver (Huh) cells subtype 7. The final, maximal DMSO concentration in the assay wells with the highest sample input (1%) was well tolerated by the cells. Favipiravir, rupintrivir and 2′,5′-*bis*-*O*-trityl uridine were used as a positive control for CHIKV, EV71 and YFV, respectively. Plant extracts that showed antiviral activity were also evaluated for cytotoxicity by the MTS (3-(4,5-dimethylthiazol-2-yl)-5-(3-carboxymethoxyphenyl)-2-(4-sulfophenyl)-2*H*-tetrazolium) method. Briefly, the same experimental set–up was used as for the antiviral assay, except that uninfected cultures were incubated with a serial dilution of plant extract for 3 days at 37 °C, then stained with MTS. Results were expressed as EC_50_ and CC_50_. The cytotoxic concentration was calculated as the CC_50_, or the concentration of plant extract required to reduce cell proliferation by 50% relative to the number of cells in the solvent-treated controls. The formula used to calculate cytotoxic activity was cytopathic effect (CPE)% = (ODcc − OD_plant extract_)/OD_CC_, where ODcc corresponds to the optical density of the uninfected and untreated cell cultures and OD_plant extract_ corresponds to the OD of uninfected cultures, treated with the extract. In addition, the selectivity index (SI) was calculated as the ratio of the CC_50_ for cell growth to the antiviral EC_50_ (CC_50_/EC_50_). SS = Selectivity Surface (integrated surface delineated by the EC_50_ curve, the CC_50_ curve and the 50% horizontal). Therapeutic Index (TI) is defined as SS × 10logSI [20].

### 2.4. Thin-Layer Chromatography (TLC) and Determination of Total Phenolic Content (TPC)

Phytochemical analysis by means of TLC was carried out for selected crude extracts, as previously described by Panda et al. [23]. The TLC plate (dimensions 2.5 × 7.5 cm, coated with silica gel 60 F254, Merck, Darmstadt, Germany) was developed with methanol:dichloromethane (9:1, *v*/*v*), methanol:dichloromethane (1:1, *v*/*v*), methanol:dichloromethane (1:9, *v*/*v*), hexane:ethyl acetate (1:3, *v*/*v*), hexane:ethyl acetate (1:1, *v*/*v*), and hexane:ethyl acetate (3:1, *v*/*v*) at ambient temperature (approximately 20 °C), and dried in an oven at 90 °C for 5 min to evaporate the solvent. The plate was visualized under ultra-violet (UV) light at (254 and 360 nm). Later, the same plate was used for visualization of the spots by spraying with 5% sulphuric acid in ethanol, followed by heating at 100 °C for 5 min. Phytochemicals were tentatively identified by comparison of Rf values and spot colours with literature data. The TPC was estimated using the Folin–Ciocalteu’s reagent according to the method described in Jouneghani et al. [24]. 

### 2.5. Statistical Analysis

The experimental results were expressed as an average of three replicates. Extracts showing interesting properties under the microscope (cell morphology was examined for minor signs of CPE or for adverse effects) were repeated three times. Heat maps were constructed using ClustVis: a web tool for visualizing clustering of multivariate data (BETA) (https://biit.cs.ut.ee/clustvis/). Further, dendrograms were constructed of the genetic relationship between the banana plant cultivars, based on Unweighted Pair Group Method with Arithmetic Mean (UPGMA) analyses [25]. For more information on genetic relationship between the cultivars, see Christelová et al. [26].

## 3. Results

None of the banana plant extracts were active against EV71 in the viral CPE reduction assay (Supplementary Material I, Figure S1).

### 3.1. Activity of Extracts against CHIKV

**Figure 1:**
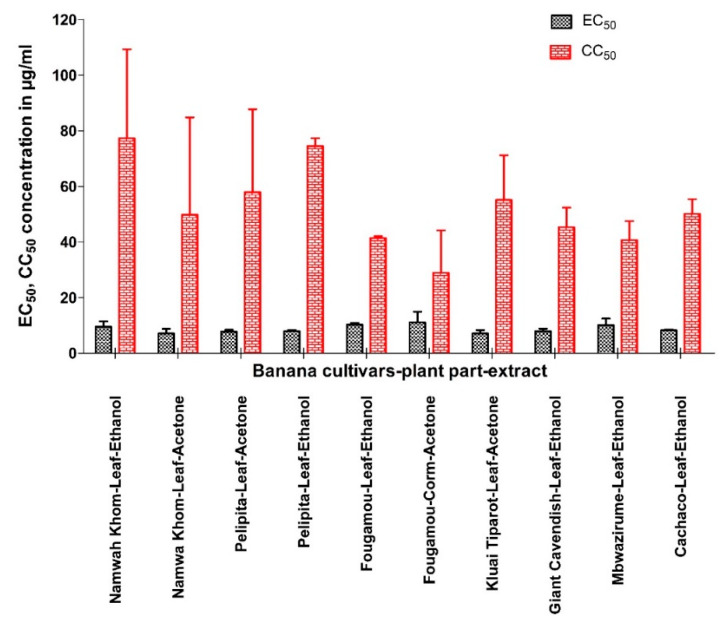
Comparison of EC_50_ and CC_50_ (mean ± SD) of selected banana cultivars-plant part-extract against Chikungunya virus.

**Figure 2:**
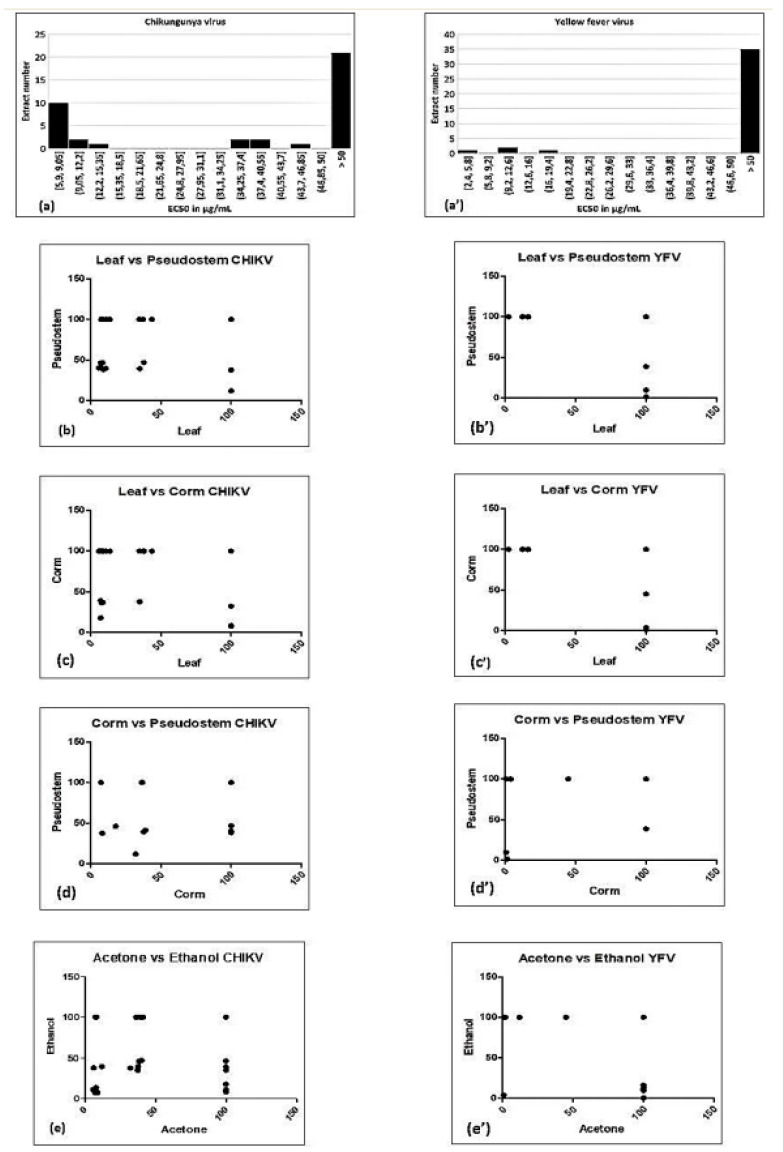
Histogram of EC_50_ values (**a**,**a’**), and correlation of bioactivity against Chikungunya virus (CHIKV) (**a**–**e**) or yellow fever virus (YFV) (**a’**–**e’**) between different plant parts (**b**–**d**,**b’**–**d’**) and different solvents (**e**,**e’**) used to prepare the extracts.

None of aqueous extracts was active against CHIKV. Extracts that show prominent antiviral activity against CHIKV are presented in Figure 1 (see also Supplementary Material II, Figure S2, Table 2). The EC_50_ varied with the solvent used to prepare an extract (~6 to 47 µg/mL). Unexpectedly, the EC_50_ values cluster in three groups: 5–15 µg/mL, 34–47 µg/mL, and inactive (Figure 2a). Possibly the activity is due to a single compound, which is present at a high or a low concentration, or absent altogether. The CC_50_ did not correlate with the EC_50_ values of the same extract, suggesting that the antiviral compound is different from the one(s) causing cytotoxicity.

Both acetone and ethanol extract(s) often exhibited significant antiviral activity, while only one hexane extract was active. With acetone, 19 extracts were active out of 30, while with ethanol 18 out of 30 extracts were active; consequently, both solvents appear equally effective. However, the two solvents do not appear to extract the same bioactive compound. Indeed, for several cultivars and plant parts, the ethanol extract is active, but not the acetone extract, or vice versa. Indeed, there is no significant correlation between the bioactivity of the ethanol versus acetone extracts (Table 2 and Figure 2e). Activity also depended on the plant part; leaf extracts (18 out of 40) were most often effective, followed by pseudostem (12 out of 40) and corm (8 out of 40). It could be assumed that if a bioactive compound is produced in one plant part, then the chance is high that it would also be produced in the immediately connecting part of the same plant. Indeed, the bioactivity in leaf is significantly correlated with that of pseudostem, and that of pseudostem with corm (Table 2 and Figure 2b–d). However, the bioactivity in the corm is not significantly correlated with that of leaf.

**Figure 3:**
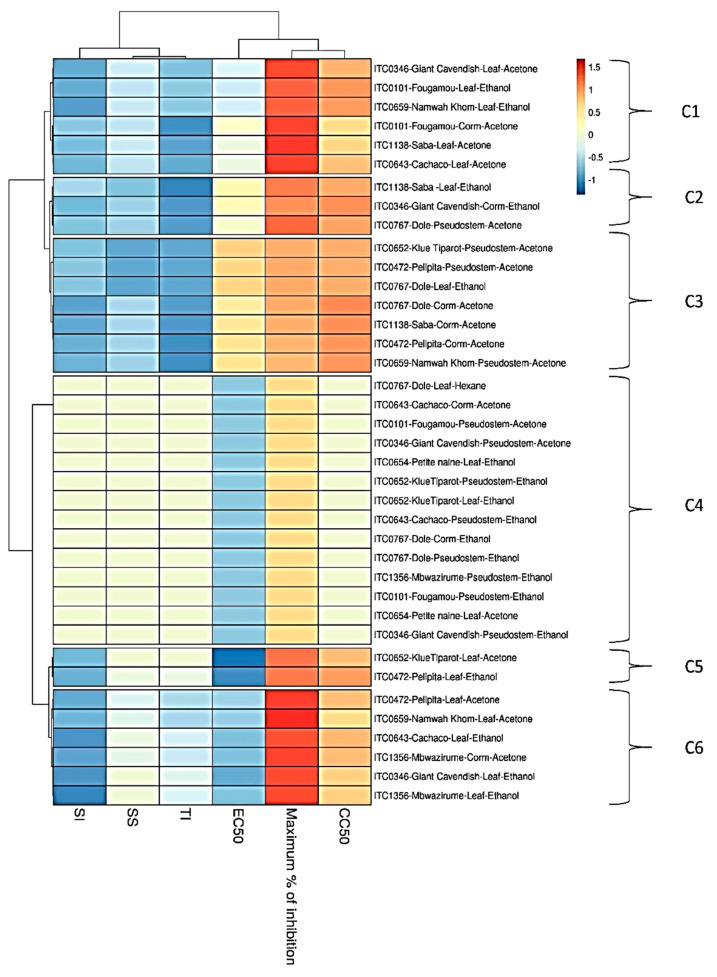
Heat map and clustering of anti-CHIKV activity of extracts from banana cultivars’ plant part. EC_50_ = 50% Effective Concentration (concentration at which 50% inhibition of virus replication is observed). CC_50_ = 50% Cytostatic/Cytotoxic Concentration (concentration at which 50% adverse effect is observed on Vero cells in parallel with antiviral assay). SI = Selectivity Index (CC_50_/EC_50_). SS = Selectivity Surface (integrated surface delineated by the EC_50_ curve, the CC_50_ curve and the 50% horizontal). TI = Therapeutic Index (SS × 10logSI). C1–C6: different clusters.

The relationship between the activities of the different extracts is visualized by a heat map using parameters such as EC_50_, CC_50_, SI, SS, and TI (Figure 3). Cluster 1 (C1) comprises four acetone extracts (leaf extracts from Giant Cavendish, Saba, Cachaco and Fougamou corm) (EC_50s_ ~ 8.5 µg/mL, SI~3.5) and two ethanol extracts from leaves of Namwa Khom and Fougamou (EC_50s_ = 11 µg/mL, SI~5). The next cluster (C2) is quite similar in antiviral activity but has a somewhat higher cytotoxicity; it comprises the ethanol extract from leaves of Saba (EC_50_ = 14 µg/mL, SI~3) and Giant Cavendish corm (EC_50_ = 18 µg/mL, SI~3.5) and the acetone extract from pseudostem of Dole (EC_50s_ = 12 µg/mL, SI~4). The neighbouring cluster (C3) is dominated by acetone extracts from the corm of Dole, Saba, Pelipita, and from the pseudostem of Klue Tiparot, Namwa Khom and Pelipita (EC_50s_ 30–40 µg/mL, maximum% of inhibition ~70%). Cluster 4 (C4), which is well separated from the other clusters, comprised the largest number of extracts: 14 (9 ethanol + 4 acetone + 1 hexane), and most extracts (8) are from the pseudostem. This cluster is characterized by high EC_50s_ as well as cytotoxicity. Cluster 5 (C5) has the most interesting antiviral properties due to low EC_50s_ combined with high CC_50_. It comprises an ethanol extract from the leaf of Pelipita and the acetone extract of Kluai Tiparot (EC_50s_ < 7 µg/mL, SI~10). Cluster 6 (C6) is dominated by leaf parts (ethanol extracts of Giant Cavendish and Cachaco, acetone extracts of Pelipita, Namwa Khom and Mbwazirume). In addition to leaf extracts, this cluster also includes acetone extract from the corm of Mbwazirume. The delineation of this cluster is due to lower EC_50s_ (<9 µg/mL) with maximum growth inhibition. Extracts showing interesting properties by microscopic examination were assayed again, and this confirmed the first results (Supplementary Material II, Figure S2).

### 3.2. Activity of Extracts against YFV

All extracts were also tested against YFV (Supplementary Material III, Figure S3, Table 3), but far fewer were actively compared to CHIKV. Activity was mostly found in corm extracts (5 out of 40), followed by leaf (4 out of 40) and pseudostem (3 out of 40). As with YFV, three clusters of EC_50_ values were seen, though with different numerical values than for CHIKV (Figure 2a’). Again, the straightforward explanation is that of a single bioactive compound being present at high, low or negligible levels. It could be the same bioactive compound as for CHIKV, whose affinity for the (presumably related) YFV target would be different. Alternatively, two different compounds could be responsible for the bioactivity against these two viruses. The latter seems more likely since the activity on CHIKV and on YFV does not correlate (Spearman rank coefficient *R* = 0.08708, 95% confidence interval −0.09898 to +0.2672).

Ethanol (6 extracts out of 30) was found to be more effective as a solvent than acetone (4 extracts out of 30) and hexane (2 extracts out of 30) (Supplementary Material III, Figure S3, Table 3). However, as for YFV, bioactivity in ethanol did not correlate with that in acetone (Table 4 and Figure 2e’). For different plant parts, activity correlated only between corm and pseudostem (Table 4 and Figure 2d’), although it is difficult to draw strong conclusions since the number of active extracts is rather small.

**Figure 4:**
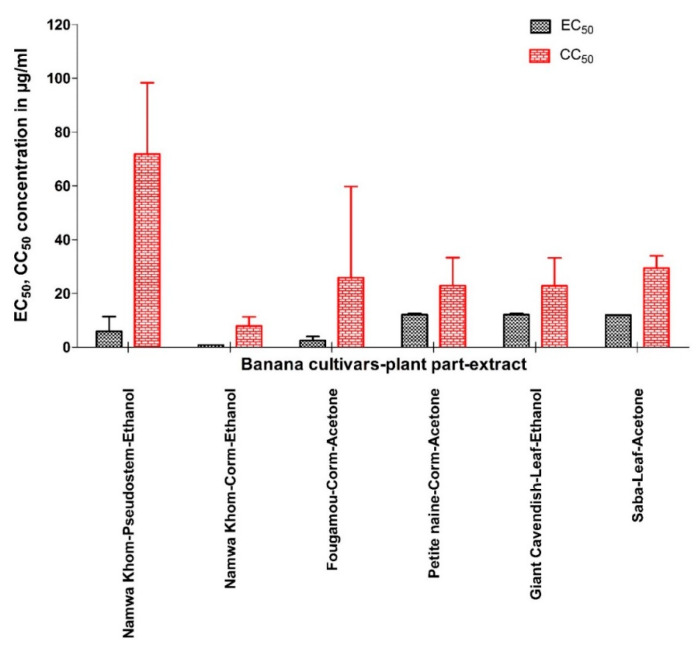
Comparison of EC_50_ and CC_50_ (mean ± SD) of selected banana cultivars’ plant part extract against yellow fever virus.

**Figure 5:**
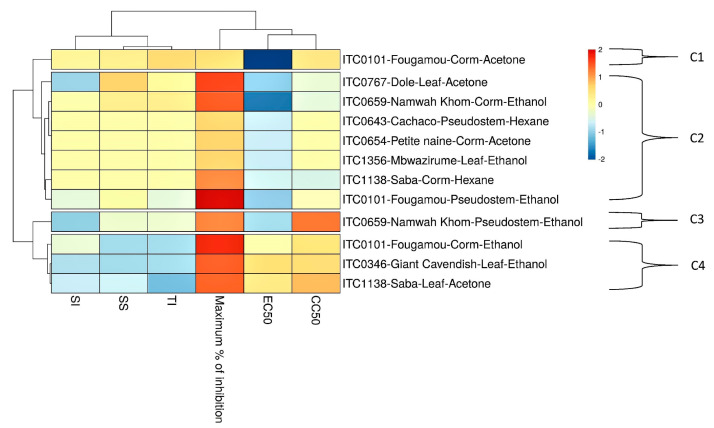
Heat map and clustering of anti-YFV activity of extracts from banana cultivars’ plant part. EC_50_ = 50% Effective Concentration (concentration at which 50% inhibition of virus replication is observed). EC_90_ = 90% Effective Concentration (concentration at which 90% inhibition of virus replication is observed). CC_50_ = 50% Cytostatic/Cytotoxic Concentration (concentration at which 50% adverse effect is observed on Huh cells in parallel with antiviral assay). SI = Selectivity Index (CC_50_/EC_50_). SS = Selectivity Surface (integrated surface delineated by the EC_50_ curve, the CC_50_ curve and the 50% horizontal). TI = Therapeutic Index (SS × 10logSI). C1–C4: different clusters.

Extracts that showed prominent antiviral activity are presented in Figure 4 with their EC_50_ and CC_50_ values. Like for CHIKV, the two appear not to be correlated. The relationship between the activities of the different extracts is visualized by a heat map using parameters such as EC_50_, CC_50_, maximum% of inhibition, SI, SS, and TI (Figure 5). The acetone extract of corm of Fougamou represents an isolated cluster (C1) due to its low EC_50_ (1.4 µg/mL) and low cytostatic/cytotoxic effect on Huh cells (CC_50_ = 76.5 µg/mL) (Supplementary Material III, Figure S3, Table 3, Figure 5). The second cluster (C2) comprises most extracts, ethanol extract of Mbwazirume leaf, Fougamou–pseudostem, Namwa Khom–corm, the acetone extracts of Dole leaf, Petite naine–corm and the hexane extracts Cachaco–pseudostem and Saba–corm. The neighboring cluster (C3) contains one extract i.e., ethanol extract from the pseudostem of Namwa Khom with attractive antiviral properties (EC_50_ = 9 µg/mL, CC_50_ = 90 µg/mL). The last cluster (C4) contains mostly extracts with strong antiviral activity with maximum inhibition but often accompanied by cytotoxicity. This cluster comprises the ethanol extracts of Fougamou–corm, leaf parts of Giant Cavendish and acetone extract of Saba leaf. Extracts showing interesting properties upon microscopic examination were assayed again, and the results were similar (Supplementary Material III, Figure S3). In summary, the most promising banana cultivars with antiviral properties against YFV are Namwa Khom (ethanol extract from corm and pseudostem) and Fougamou (corm–acetone and pseudostem–ethanol) (Figure 4).

### 3.3. TLC and TPC

Using methanol:dichloromethane (1:9, *v*/*v*) as mobile phase, numerous absorbing bands were observed under UV light (254 nm and 360 nm) with ethanol extracts. Furthermore, after exposing the plates to 5% sulphuric acid in ethanol, spots were visualized, and the reaction products were compared under UV light. Interestingly, several extracts show similar fingerprints (observed colour as well as Rf), signifying the presence of similar chemical classes or even compounds. Most of the plant extracts showed brown/dark brown spots at 254 nm, and pink, light pink, or yellow spots at 360 nm UV, confirming the presence of phenolics and flavonoids, while blue fluorescence indicates saponins and terpenoids (Supplementary Material IV, Figure S4a–c; Table S1ab). Similarly, acetone and hexane extracts were well separated with hexane:ethyl acetate (1:1) as mobile phase but we could not observe as diverse a range of phytoconstituents as in the ethanol extracts; only the presence of phenolics was observed (Supplementary Material IV, Figure S4d–f; Table S1c–d). Interestingly, several extracts show similar fingerprints (observed colour as well as Rf), indicating the presence of similar chemical classes or even compounds, but which of these constitute the bioactive compounds responsible for the detected activities requires further study. Selected extracts were also quantified for their total phenolic content (TPC) from the regression equation of the gallic acid calibration curve (*R*^2^ = 0.9902), expressed in gallic acid equivalents (GAE) as µg/mg of the crude extract (Supplementary Material IV, Figure S4g). There is over a 10-fold variation in TPC between extracts in various solvents of different cultivars (24 to 309 µg of GAE/mg extracts) (Supplementary Material IV, Table S1e), although most extracts show values between 140 and 198 µg of GAE/mg extract. For more information see Supplementary Material IV, Figure S4g and Table S1e. Across all samples, TPC correlates best between the corresponding extracts of leaf versus pseudostem (*r* = 0.82), suggesting that the TPC in most cultivars does not differ very much between plant parts.

## 4. Discussion

Notwithstanding continuous advances made in antiviral therapy, millions of people are still affected by viral diseases. This may lead to death in severe cases, especially if no drug treatment is available. Even when effective antiviral drugs exist, treatment may not be successful due to the emergence of resistant strains. Viral replication is largely dependent on the host cell (the virus acts as an intracellular parasite). Therefore, it is difficult to find an effective antiviral compound that acts only on the virus without affecting the host cell. This has been achieved for viruses with essential enzymes absent in the host (like reverse transcriptase), or with viral enzymes sufficiently different from the host orthologues (like some protease inhibitors).

Many currently used antiviral drugs are expensive and have side effects. Hence, it is interesting to look for novel sources of antiviral compounds. Because of prior reports of activity against HIV in banana plants, we tested the antiviral properties of banana extracts as a potential source of novel antiviral drugs.

CHIKV is one of the re-emerging vector-borne viral diseases and considered as a neglected tropical disease, mainly because the affected regions are in Africa and Southeast Asia. CHIKV is transmitted to humans by infected mosquitoes, and there is at present no cure for this disease (https://www.who.int/news-room/fact-sheets/detail/chikungunya). It causes fever and severe joint pains. Moreover, there are no currently approved vaccines or antiviral treatments available yet for the prevention or treatment of CHIKV infection; it is therefore important to search for new bioactive molecules. 

Similarly, another important viral disease (yellow fever) is an acute viral haemorrhagic disease transmitted by mosquitos. The virus is endemic to tropical areas of Africa and Central and South America. There is currently no specific antiviral drug for yellow fever. It can be prevented by vaccination, but due to poor coverage of vaccination in Africa, there are threats of re-emergence in its endemic habitat, and new drugs are urgently needed.

Enterovirus 71 infections can cause mild hand, foot and mouth disease, but also lead (more rarely) to severe fatal neurological complications. Vaccines have been developed in China, but are not used population-wide due to concerns about side-effects. Antiviral drugs would therefore be welcome.

Banana (*Ensete superbum* Roxb., Cheesman, Family: Musaceae), is commonly used by Indian tribes for treating measles (Nandurbar district, state-Maharashtra, India) as well as for chickenpox (tribes of Dang district, state-Gujrat, India [27,28]. However, there is no scientific evidence that this banana has antiviral properties except for one study [29]. A lectin (BanLec) from *Musa* *acuminata* was found to be a potent inhibitor of HIV replication [16,17]. Therefore, in the present study, we systematically tested different parts of 10 banana cultivars for antiviral activity. We did not find any prior reports on antiviral activity against YFV, CHIKV or EV71.

In the cluster analysis for both viruses, the antiviral parameters cluster in the same way. SS, SI and TI cluster very tightly together, which is not surprising since they all describe aspects of the therapeutic window. Logically, EC_50_ and maximum % inhibition also correlate well, since both reflect viral inhibition. Neither correlate well with CC_50_, which stands to reason since antiviral and cytotoxic effects presumably have different mechanisms.

Although some solvents (ethanol, acetone) extract active compounds much more often than others (hexane, water) in our study, this cannot necessarily be generalized to other plants or other bioactivities. It does suggest that our bioactive compounds are not very hydrophobic nor strongly hydrophilic. Not only does the solubility in acetone and ethanol differ for most chemicals, but the fact that the two solvents give different activity results for many plant samples indicates that they probably do not extract the same compounds.

In the present study, phytochemical analysis of different extracts revealed the presence of flavonoids, saponins and terpenoids. Fahim et al. recently investigated the phytochemical spectrum from fruits of *M.* x *paradisiaca* oils from different geographical areas of India. They found an intense peak with similar Rf value in the TLC (0.55, 0.68, 0.81, 0.94). Phenolic compounds, such as caffeic acid, ferulic acid, catecholamines, flavanones, flavanols, and tocopherols were reported in banana previously [30,31]. Several phenolic compounds are well known for their antiviral activity [32,33].

We also estimated TPC in a number of our crude extracts (Supplementary Material IV, Table S1e). There is a more than 20-fold difference in TPC among extracts, and even the extract with the lowest TPC i.e., Fougamou–leaf–ethanol (24 µg of GAE/mg extract) showed strong anti-CHIKV activity (EC_50s_ = 10.8 µg/mL, SI~5.65, SS = 9.66, TI = 7.26). This does not preclude that a specific phenolic compound may contribute to the observed activity. Aquino et al. also observed variation among the different plant parts, but not so much among the cultivars, viz., TPC among 15 cultivars ranged from 23.15 to 33.28 mg/100 gm GAE for unripe pulp, 42.4 to 77.07 mg/100 gm GAE for ripe pulp, 32 to 61 mg/100 gm GAE for unripe peel, and 60.39 to 115.7 mg/100 gm GAE for ripe peel [34]. We find typical values of 160 µg/mg GAE in our crude extracts. Since the yields of the crude extracts are typically 25–75 mg/g dried plant material for the organic solvents, and the weight loss upon drying is typically 90%, this means 40–120 mg/100 g original plant material. These TPC values are in the same range as those reported in the literature. Although the concentration of metabolites may differ considerably between plant parts, it is reasonable to assume that the presence of a bioactive compound in one plant part of a cultivar renders it more likely that it is also present in neighboring parts of the same cultivar.

**Figure 6:**
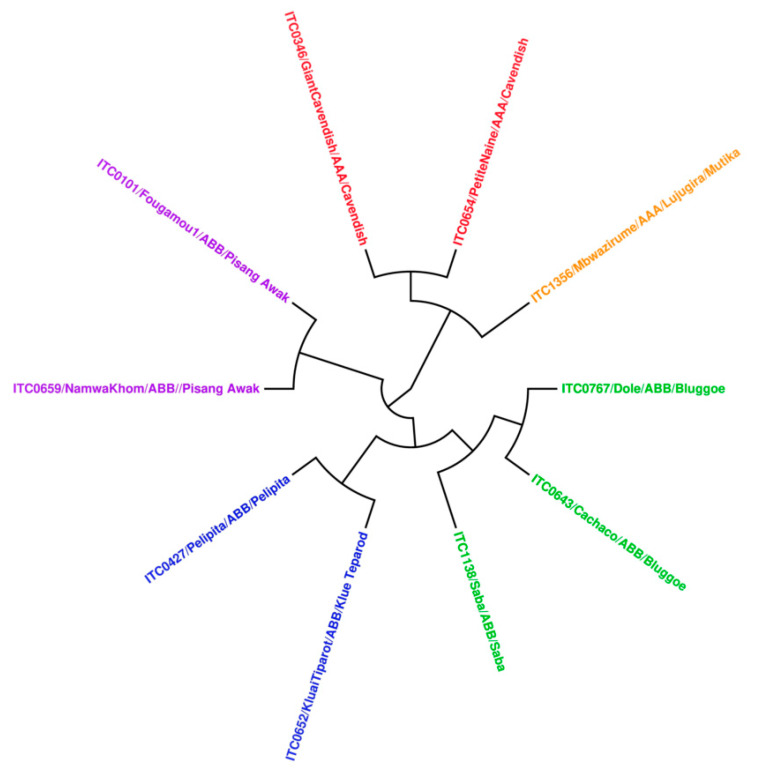
Construction of UPGMA dendrogram using all tested cultivars.

Assuming that the metabolomes of two plant cultivars will resemble each other more the closer the cultivars are related genetically; we expect a correlation between the bioactivities of cultivars and their genetic relatedness. Therefore, a dendrogram was constructed based on DNA sequence data from the tested cultivars, using the unweighted pair group method with arithmetic mean (UPGMA) [26]. This cladogram mainly distributed into two main clades, corresponding to karyotypes AAA and ABB (Figure 6). The ABB clade is nicely correlated with activity, and the cultivars with genome ABB were typically found to have anti-CHIKV activity.

Two banana cultivars showed interesting antiviral activity against YFV: ethanol extracts from pseudostem of the very close genetically-related Fougamou and Namwa Khom (genome ABB) (Figure 6). Three other cultivars (Saba–leaf–acetone, Petite naine–corm–acetone, Giant Cavendish–corm–ethanol) were also found to be effective, but they are also cytotoxic for Huh cells. Therefore, also for YFV, potent antiviral activity is found in some cultivars with genome ABB, but no activity in cultivars with genome AAA (Giant Cavendish, Mbwazirume and Petite naine). All extracts were tested on EV71, but none inhibited the infection of RD host cells by EV 71.

The crude extracts that we tested are complex mixtures, where individual compounds or combinations thereof can contribute to the antiviral effect. Given that we tested crude extracts, the EC_50_ values are quite impressive for certain cultivars compared to previous studies with medicinal plants [21,35]. The results of the present study establish a base to start bioassay-guided purification to identify the active compounds responsible for the antiviral activity.

## 5. Conclusions

In conclusion, the present results demonstrate that the genetically closely-related banana cultivars with genome ABB such as Namwa Khom, Pelipita, Fougamou and Kluai Tiparot are potential sources for developing antiviral drugs against CHIKV, while Namwa Khom and Fougamou cultivars can provide antiviral compounds against YFV. 

## Figures and Tables

**Table 1:** List and characteristics of banana cultivars studied for antiviral activity.

ITC	Cultivar	Genome	Subgroup	DArT
ITC0767	Dole	ABB	Bluggoe	Other ABB
ITC0643	Cachaco	ABB	Bluggoe	*Musa balbisiana*
ITC1138	Saba	ABB	Saba	Other ABB
ITC0652	Kluai Tiparot	ABB	unknown	*Musa balbisiana*
ITC0472	Pelipita	ABB	unknown	Other ABB
ITC0659	Namwah Khom	ABB	Pisang Awak	Pisang Awak
ITC0101	Fougamou	ABB	Pisang Awak	Pisang Awak
ITC0654 *	Petite Naine	AAA	Cavendish	AAA Cavendish
ITC0346	Giant Cavendish	AAA	Cavendish	AAA Cavendish
ITC1356	Mbwazirume	AAA	Mutika/Lujugira	AAAh

* From Africa, all others collected from the Laboratory of Tropical Crop Improvement greenhouse, Leuven, DArT—Diversity Array Technology [18].

**Table 2:** Antiviral and cytotoxic activity of banana varieties against Chikungunya virus.

Code No.	Variety	Parts Used	Extract Tested	EC_50_ (µg/mL)	EC_90_ (µg/mL)	Maximum % Inhibition	CC_50_ (µg/mL)	SI	SS	TI
BAVARIE1_001	Saba	Leaf	Acetone	7.88	12.6	72.2	23.2	2.94	5.05	2.36
BAVARIE1_003	Saba	Corm	Acetone	36.3	-	73.3	100	2.76	5.13	2.26
BAVARIE1_004	Pelipita	Leaf	Acetone	7.21	-	80.6	38.1	5.28	11	7.97
BAVARIE1_005	Pelipita	Pseudostem	Acetone	41.2	-	69.9	69	1.67	0.026	0.006
BAVARIE1_006	Pelipita	Corm	Acetone	38.9	-	71	100	2.57	4.31	1.77
BAVARIE1_007	Kluai Tiparot	Leaf	Acetone	6.27	16	100	63.1	10.1	25.3	25.4
BAVARIE1_008	Kluai Tiparot	Pseudostem	Acetone	40.3	-	60	63	1.57	0.093	0.018
BAVARIE1_010	Petit Naine	Leaf	Acetone	37.5	-	76.8	>100	>2.67	>6.4	>2.72
BAVARIE1_0013	Cavendish	Leaf	Ethanol	7.29	16.1	100	44.7	6.13	19.8	15.6
BAVARIE1_0014	Cavendish	Pseudostem	Ethanol	46.2	89	97.1	>100	>2.16	>7.89	>2.64
BAVARIE1_0015	Cavendish	Corm	Ethanol	17.8	-	65.4	63.1	3.55	4.91	2.7
BAVARIE1_0016	Fougamou	Leaf	Ethanol	10.8	-	82.8	60.9	5.65	9.66	7.26
BAVARIE1_0017	Fougamou	Pseudostem	Ethanol	39.7	67	100	>100	>2.52	>11.6	>4.65
BAVARIE1_0019	Mbwazirume	Leaf	Ethanol	8.41	15	100	44.7	5.32	18.9	13.7
BAVARIE1_0020	Mbwazirume	Pseudostem	Ethanol	39	67.6	92.2	>100	>2.56	>10.3	>4.22
BAVARIE1_0022	Dole	Leaf	Ethanol	35	-	59.28	58.5	1.67	0.164	0.036
BAVARIE1_0023	Dole	Pseudostem	Ethanol	39.4	-	85.4	>100	>2.54	>8.76	>3.55
BAVARIE1_0024	Dole	Corm	Ethanol	37.8	-	85.8	>100	>2.65	>9.3	>3.93
BAVARIE1_0025	Cachaco	Leaf	Ethanol	8.41	15.7	91.4	50.2	5.97	17	13.2
BAVARIE1_0026	Cachaco	Pseudostem	Ethanol	46.5	-	82.5	>100	>2.15	>6.07	>2.02
BAVARIE1_0028	Namwah Khom	Leaf	Ethanol	10.9	>20	79.3	54.7	5	10.2	7.17
BAVARIE1_0031	Saba	Leaf	Ethanol	13.7	-	65.2	44.7	3.27	2.46	1.26
BAVARIE1_0034	Pelipita	Leaf	Ethanol	7.59	13.3	92.3	72.5	9.55	21.9	21.5
BAVARIE1_0037	Kluai Tiparot	Leaf	Ethanol	37.9	63.7	92.6	>100	>2.64	>11	>4.62
BAVARIE1_0038	Kluai Tiparot	Pseudostem	Ethanol	47	-	82.5	>100	>2.13	>6.01	>1.97
BAVARIE1_0040	Petit Naine	Leaf	Ethanol	34.8	-	77.6	>100	>2.87	>7.28	>3.33
BAVARIE1_0043	Cavendish	Leaf	Acetone	9.06	17.8	76.4	38.1	4.2	8.15	5.08
BAVARIE1_0044	Cavendish	Pseudostem	Acetone	38.4	-	85.7	>100	>2.6	>9.09	>3.78
BAVARIE1_0047	Fougamou	Pseudostem	Acetone	37.7	-	80.6	>100	>2.65	>7.93	>3.36
BAVARIE1_0048	Fougamou	Corm	Acetone	8.25	-	68.47	20	2.43	3.55	1.37
BAVARIE1_0051	Mbwazirume	Corm	Acetone	7.47	20.0	90	44.7	5.99	14.9	11.6
BAVARIE1_0053	Dole	Pseudostem	Acetone	12.1	-	70.72	44.7	3.69	4.41	2.5
BAVARIE1_0054	Dole	Corm	Acetone	32.2	-	74.8	100	3.11	6.1	3.01
BAVARIE1_0055	Cachaco	Leaf	Acetone	8.56	15.9	72.6	29.9	3.49	5.64	3.06
BAVARIE1_0057	Cachaco	Corm	Acetone	36.7	-	66.7	>100	>2.73	>6.69	>2.92
BAVARIE1_0058	Namwah Khom	Leaf	Acetone	5.94	10.4	79.86	27.6	4.65	9.67	6.45
BAVARIE1_0059	Namwah Khom	Pseudostem	Acetone	40.2	-	69.91	100	2.49	3.95	1.56
BAVARIE1_0100	Dole	Leaf	Hexane	43.7	-	77.38	>100	>2.29	>5.87	>2.11
Positive control	Favipiravir	-	-	2.65	-	-	>100	>37.7	-	-

EC_50_ = 50% Effective Concentration (concentration at which 50% inhibition of virus replication is observed); EC_90_ = 90% Effective Concentration (concentration at which 90% inhibition of virus replication is observed); CC_50_ = 50% Cytostatic/Cytotoxic Concentration (concentration at which 50% adverse effect is observed on Vero cells in parallel with antiviral assay); SI = Selectivity Index (CC_50_/EC_50_); SS = Selectivity Surface (integrated surface delineated by the EC_50_ curve, the CC_50_ curve and the 50% horizontal); TI = Therapeutic Index (SS × 10logSI). (-) Data absent

**Table 3:** Antiviral and cytotoxic activity of banana varieties against yellow fever virus.

Code No.	Variety	Parts Used	Extract Tested	EC_50_ (µg/mL)	EC_90_ (µg/mL)	Maximum % Inhibition	CC_50_ (µg/mL)	SI	SS	TI
BAVARIE1_001	Saba	Leaf	Acetone	12.2	>20	64	26.2	2.14	2.27	0.75
BAVARIE1_0012	Petit Naine	Corm	Acetone	44.8	88	97.57	>100	>2.23	>8.29	>2.89
BAVARIE1_0013	Cavendish	Leaf	Ethanol	12.5	>20	52.05	13.8	1.1	0.358	0.015
BAVARIE1_0017	Fougamou	Pseudostem	Ethanol	1.55	2.62	75.27	5.23	3.37	6.56	3.46
BAVARIE1_0019	Mbwazirume	Leaf	Ethanol	16.3	-	89.81	>100	>6.15	>19.2	>15.1
BAVARIE1_0029	Namwah Khom	Pseudostem	Ethanol	9.77	-	89.64	90.6	9.27	19.2	18.5
BAVARIE1_0030	Namwah Khom	Corm	Ethanol	0.57	<0.8	80	5.77	9.97	17.1	17
BAVARIE1_0048	Fougamou	Corm	Acetone	1.44	2.31	100	76.5	53.1	63.2	109
BAVARIE1_0052	Dole	Leaf	Acetone	2.41	51.7	100	6.76	2.81	29.3	13.1
BAVARIE1_0104	Cachaco	Pseudostem	Hexane	38.5	74.8	107	>100	>2.6	>11.9	>4.93
BAVARIE1_0111	Saba	Corm	Hexane	<0.8	<0.8	57.4	1.12	>1.4	>0.54	>0.08
BAVARIE1_0118	Fougamou	Corm	Ethanol	3.84	>100	51.31	8.94	2.33	0.241	0.088
Positive control	2′,5′-bis-*O*-trityl uridine		-	1.2 μM	-	-	>100 μM	>80	-	-

EC_50_ = 50% Effective Concentration (concentration at which 50% inhibition of virus replication is observed); EC_90_ = 90% Effective Concentration (concentration at which 90% inhibition of virus replication is observed); CC_50_ = 50% Cytostatic/Cytotoxic Concentration (concentration at which 50% adverse effect is observed on Huh cells in parallel with antiviral assay; SI = Selectivity Index (CC_50_/EC_50_); SS = Selectivity Surface (integrated surface delineated by the EC_50_ curve, the CC_50_ curve and the 50% horizontal); TI = Therapeutic Index (SS × 10logSI); (-) Data absent.

**Table 4:** Calculation of nonparametric correlation (Spearman r) of EC_50s_ between different plant parts.

Parts	Parameters	CHIKV	YFV
Leaf vs. Pseudostem	Spearman r	0.4965	−0.09467
	95% confidence interval	0.2096 to 0.7046	−0.4026 to 0.2325
	P value (two-tailed)	0.0011	0.5612
	Is the correlation significant? (alpha = 0.05)	Yes	No
Leaf vs. Corm	Spearman r	0.1872	−0.1392
	95% confidence interval	−0.1414 to 0.4787	−0.4398 to 0.1894
	P value (two-tailed)	0.2473	0.3916
	Is the correlation significant? (alpha = 0.05)	No	No
Corm vs. Pseudostem	Spearman r	0.3755	0.4228
	95% confidence interval	0.06294 to 0.6210	0.1188 to 0.6544
	P value (two-tailed)	0.0169	0.0066
	Is the correlation significant? (alpha = 0.05)	Yes	Yes
Acetone vs. Ethanol	Spearman r	0.2695	0.0442
	95% confidence interval	−0.1117 to 0.5815	−0.2799 to 0.3593
	P value (two-tailed)	0.1499	0.7863
	Is the correlation significant? (alpha = 0.05)	No	No

## Supplementary Materials

The following are available online at https://www.mdpi.com/1999-4915/12/5/549/s1, Figure S1: Antiviral activity of extracts against enterovirus; Figure S2: Antiviral activity of extracts against Chikungunya virus; Figure S3: Antiviral activity of extracts against yellow fever virus; Figures S4a–f: TLC of selected extracts (acetone, ethanol, hexane); Figure S4g: Regression curve for gallic acid assayed with the Folin–Ciocalteu reagent. Figures S4h: Distribution of total phenolic content across all samples analysed. Table S1a–d: Rf value of TLC (acetone, ethanol, hexane extracts) Table S1e: Total phenolic content (gallic acid equivalents, µg/mg) of different extracts.
