# Supplementary material for: Antiviral and Cytotoxic Activity of Different Plant Parts of Banana (Musa spp.)
Source: Viruses. 2020 May 15;12(5):549. doi: 10.3390/v12050549 (PMC7291111; doi:10.3390/v12050549)
Supplement: Supplementary file 1 [file viruses-12-00549-s001.zip › Supplementary Files~/Supplementary material I-Enterovirus-SP.pdf]

Antiviral and Cytotoxic Activity of Different Plant Parts of Banana (*Musa* spp.)

Sujogya Kumar Panda [1,2,*], Ana Hortência Fonsêca Castro [1,3], Ramin Saleh Jouneghani [1], Pieter Leyssen [4], Johan Neyts [4], Rony Swennen [5,6,7] and Walter Luyten [1]

[1] Department of Biology, Katholieke Universiteit Leuven, 3000 Leuven, Belgium; acastro905@gmail.com (A.H.F.C.); r.saleh.j@gmail.com (R.S.J.); walter.luyten@kuleuven.be (W.L.)
[2] Mayurbhanj Biological Research (MBR), Bhanjpur, Baripada 757002, Odisha, India
[3] Plant Physiology and Biochemistry, Universidade Federal de São João Del-Rei, Av. Sebastião Gonçalves Coelho, 400—Chanandour, Divinópolis MG 35501-296, Brazil
[4] Rega Institute for Medical Research, Laboratory of Virology and Chemotherapy, Katholieke Universiteit Leuven, 3000 Leuven, Belgium; pieter.leyssen@kuleuven.be (P.L.); johan.neyts@kuleuven.be (J.N.)
[5] International Institute of Tropical Agriculture, Arusha P.O. Box 447, Tanzania; rony.swennen@kuleuven.be
[6] Laboratory of Tropical Crop Improvement, Division of Crop Biotechnics, Katholieke Universiteit Leuven, 3001 Leuven, Belgium
[7] Bioversity International, 3001 Leuven, Belgium
* Correspondence: sujogya.panda@kuleuven.be; Tel.: +32-16-373467

Supplementary material I

Figure S1: Antiviral activity of extracts against enterovirus. Extracts (primary code) tested mentioned under the header compounds in each panel. EC_{50} = 50% Effective Concentration (concentration at which 50% inhibition of virus replication is observed); EC_{90} = 90% Effective Concentration (concentration at which 90% inhibition of virus replication is observed); CC_{50} = 50% Cytostatic/Cytotoxic Concentration (concentration at which 50% adverse effect is observed on RD cells in parallel with antiviral assay); SI = Selectivity Index (CC_{50}/EC_{50}); SS = Selectivity Surface (integrated surface delineated by the EC_{50} curve, the CC_{50} curve and the 50% horizontal); TI = Therapeutic Index (SS × 10logSI); AV-Antiviral; AM-Antimetabolic.

Compound	Virus			Cell	AV Method		AM Method	
Primary code	Species	Type	Strain	Type	Method	Type	Method	Type
BAVARIE1_0001	Enterovirus	71	BRCR	RD	Absorbance	MTS - 498nm	Microscopy	Tox scoring

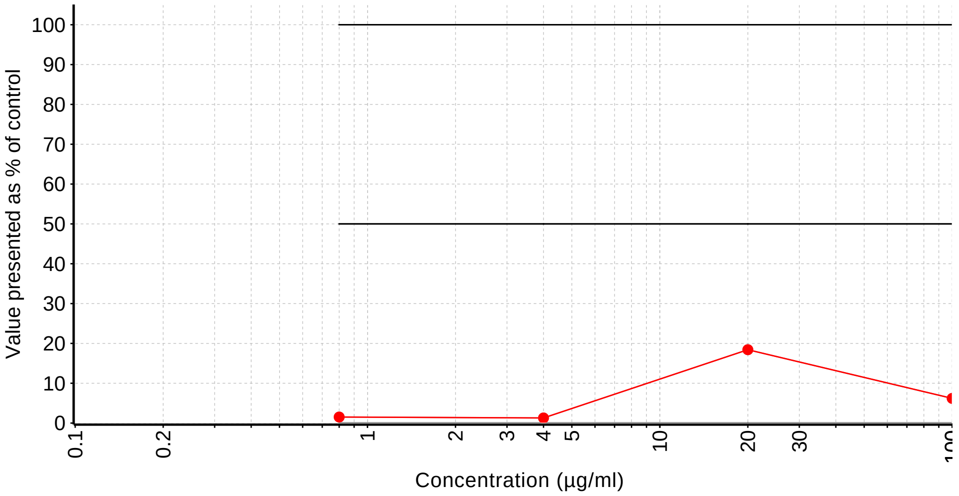

$CC50_{av}$		
EC50	>	100
EC90	>	100
SI_{av}		
SS_{av}		
TI_{av}		
Max %		18.42
[] Max		20
Approved AV		1
Interesting AV		0
Page		1 / 96

Anti-Viral
Anti-Metabolic

Export chart data to CSV

Summary values			
**Statistic**	**CC50**	**EC50**	**EC90**
Median		> 100	> 100
Med.Abs.Dev.			
Mean		> 100	> 100
Stdev.			

Compound	Virus			Cell	AV Method		AM Method	
Primary code	Species	Type	Strain	Type	Method	Type	Method	Type
BAVARIE1_0002	Enterovirus	71	BRCR	RD	Absorbance	MTS - 498nm	Microscopy	Tox scoring

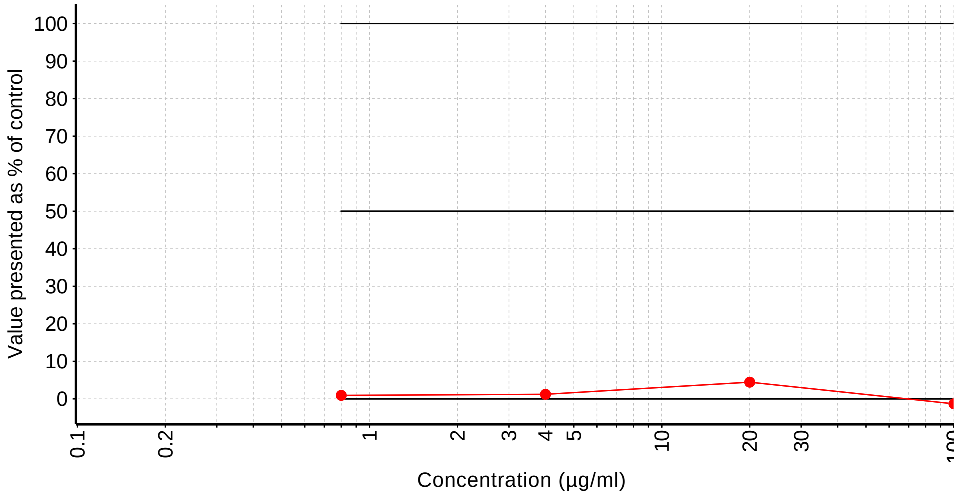

$CC50_{av}$		
EC50	>	100
EC90	>	100
SI_{av}		
SS_{av}		
TI_{av}		
Max %		4.44
[] Max		20
Approved AV		1
Interesting AV		0
Page		2 / 96

Anti-Viral
Anti-Metabolic

Export chart data to CSV

Summary values			
**Statistic**	**CC50**	**EC50**	**EC90**
Median		> 100	> 100
Med.Abs.Dev.			
Mean		> 100	> 100
Stdev.			

Compound	Virus			Cell	AV Method		AM Method	
Primary code	Species	Type	Strain	Type	Method	Type	Method	Type
BAVARIE1_0003	Enterovirus	71	BRCR	RD	Absorbance	MTS - 498nm	Microscopy	Tox scoring

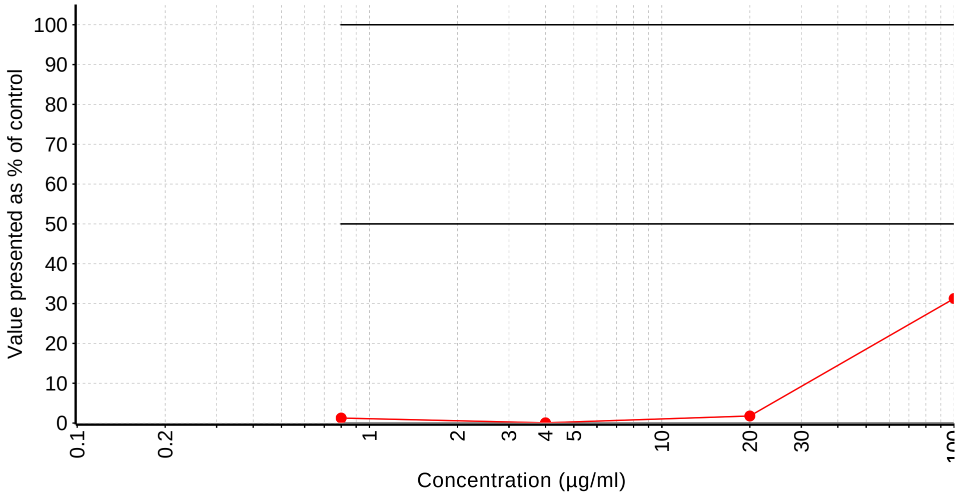

$CC50_{av}$		
EC50	>	100
EC90	>	100
SI_{av}		
SS_{av}		
TI_{av}		
Max %		31.27
[] Max		100
Approved AV		1
Interesting AV		0
Page		3 / 96

Anti-Viral
Anti-Metabolic

Export chart data to CSV

Summary values			
**Statistic**	**CC50**	**EC50**	**EC90**
Median		> 100	> 100
Med.Abs.Dev.			
Mean		> 100	> 100
Stdev.			

Compound	Virus			Cell	AV Method		AM Method	
Primary code	Species	Type	Strain	Type	Method	Type	Method	Type
BAVARIE1_0004	Enterovirus	71	BRCR	RD	Absorbance	MTS - 498nm	Microscopy	Tox scoring

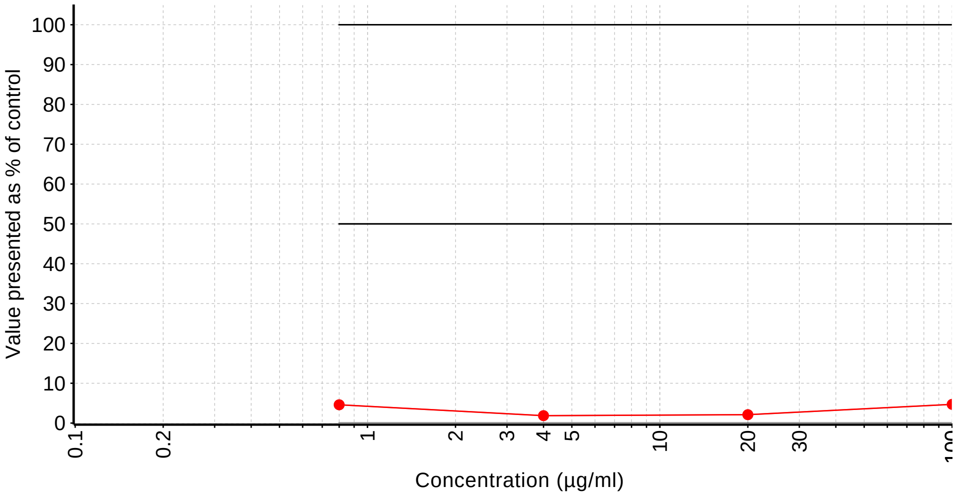

$CC50_{av}$	
EC50	> 100
EC90	> 100
SI_{av}	
SS_{av}	
TI_{av}	
Max %	4.70
[] Max	100
Approved AV	1
Interesting AV	0
Page	4 / 96

Anti-Viral
Anti-Metabolic

Export chart data to CSV

Summary values			
**Statistic**	**CC50**	**EC50**	**EC90**
Median		> 100	> 100
Med.Abs.Dev.			
Mean		> 100	> 100
Stdev.			

Compound	Virus			Cell	AV Method		AM Method	
Primary code	Species	Type	Strain	Type	Method	Type	Method	Type
BAVARIE1_0005	Enterovirus	71	BRCR	RD	Absorbance	MTS - 498nm	Microscopy	Tox scoring

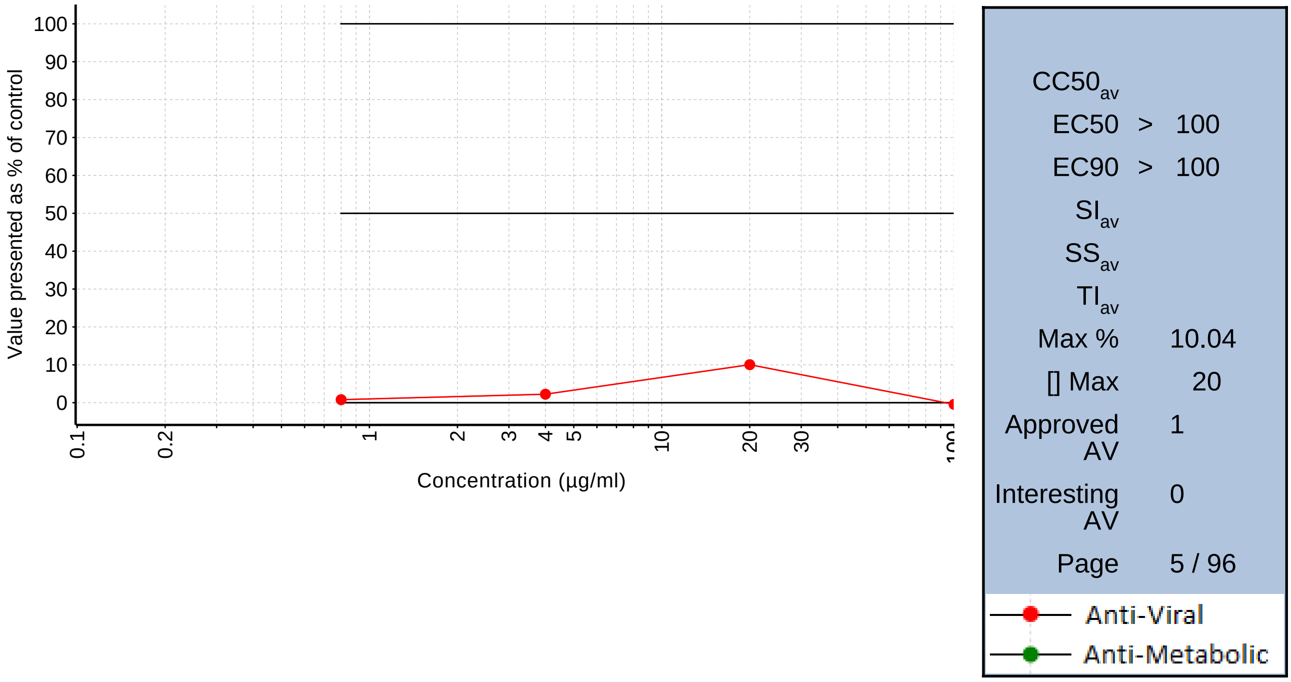

Export chart data to CSV

Summary values			
**Statistic**	**CC50**	**EC50**	**EC90**
Median		> 100	> 100
Med.Abs.Dev.			
Mean		> 100	> 100
Stdev.			

Compound	Virus			Cell	AV Method		AM Method	
Primary code	Species	Type	Strain	Type	Method	Type	Method	Type
BAVARIE1_0006	Enterovirus	71	BRCR	RD	Absorbance	MTS - 498nm	Microscopy	Tox scoring

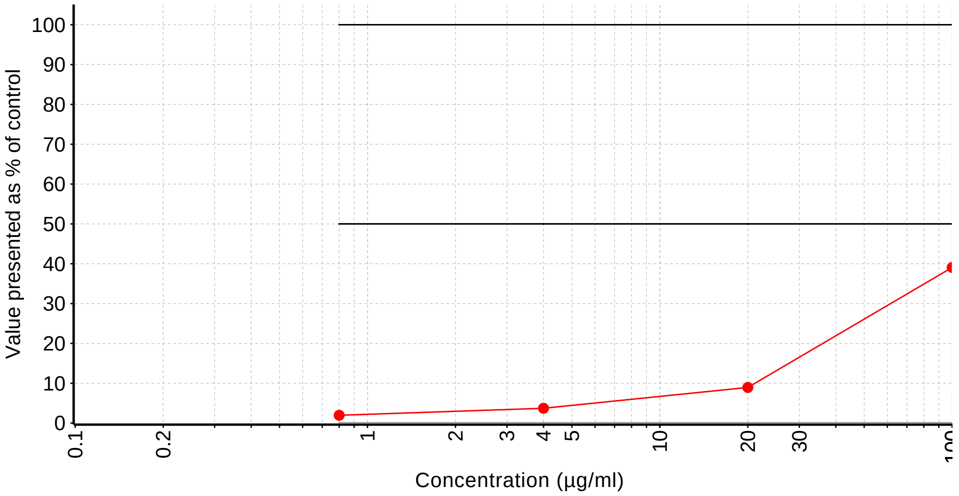

$CC50_{av}$		
EC50	>	100
EC90	>	100
SI_{av}		
SS_{av}		
TI_{av}		
Max %		39.07
[] Max		100
Approved AV		1
Interesting AV		0
Page		6 / 96

Anti-Viral
Anti-Metabolic

Export chart data to CSV

Summary values			
**Statistic**	**CC50**	**EC50**	**EC90**
Median		> 100	> 100
Med.Abs.Dev.			
Mean		> 100	> 100
Stdev.			

**Compound**	**Virus**			**Cell**	**AV Method**		**AM Method**	
Primary code	Species	Type	Strain	Type	Method	Type	Method	Type
BAVARIE1_0007	Enterovirus	71	BRCR	RD	Absorbance	MTS - 498nm	Microscopy	Tox scoring

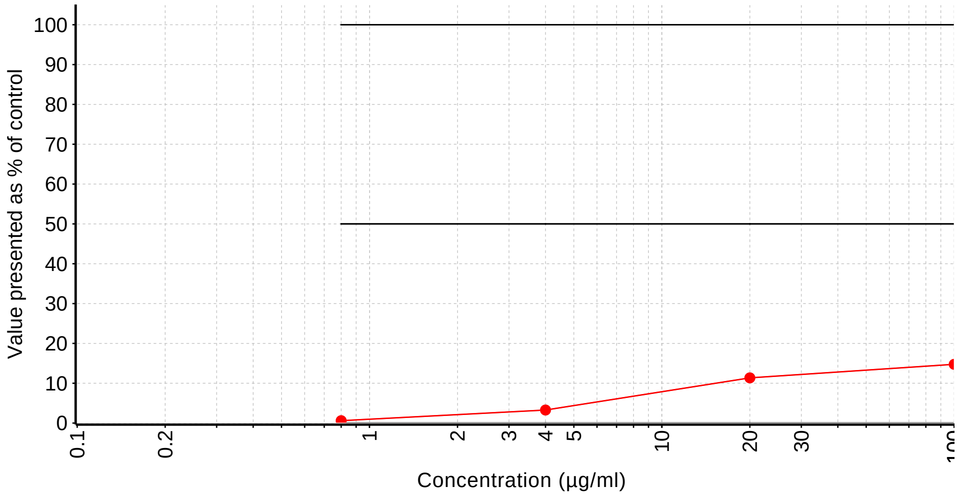

$CC50_{av}$		
EC50	>	100
EC90	>	100
SI_{av}		
SS_{av}		
TI_{av}		
Max %		14.75
[] Max		100
Approved AV		1
Interesting AV		0
Page		7 / 96

Anti-Viral
Anti-Metabolic

Export chart data to CSV

**Summary values**			
**Statistic**	**CC50**	**EC50**	**EC90**
Median		> 100	> 100
Med.Abs.Dev.			
Mean		> 100	> 100
Stdev.			

**Compound**	**Virus**			**Cell**	**AV Method**		**AM Method**	
Primary code	Species	Type	Strain	Type	Method	Type	Method	Type
BAVARIE1_0008	Enterovirus	71	BRCR	RD	Absorbance	MTS - 498nm	Microscopy	Tox scoring

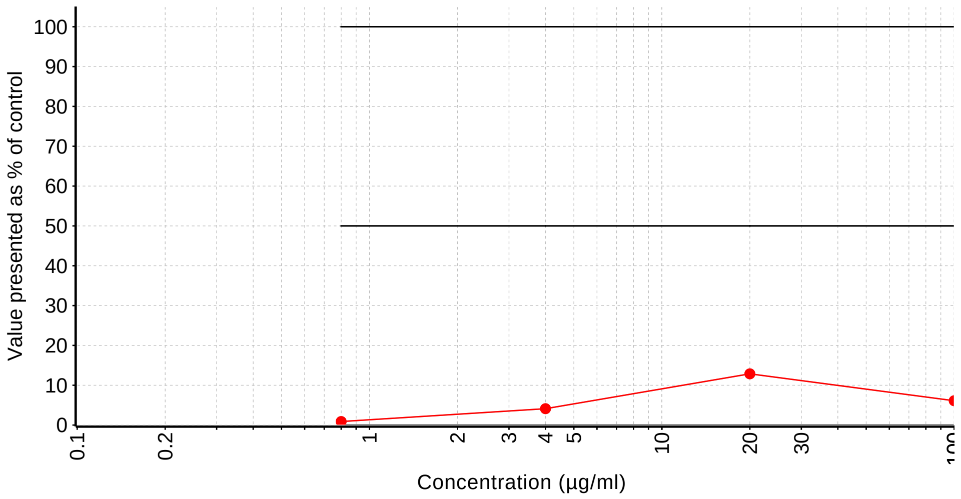

$CC50_{av}$		
EC50	>	100
EC90	>	100
SI_{av}		
SS_{av}		
TI_{av}		
Max %		12.87
[] Max		20
Approved AV		1
Interesting AV		0
Page		8 / 96

Anti-Viral
Anti-Metabolic

Export chart data to CSV

Summary values			
**Statistic**	**CC50**	**EC50**	**EC90**
Median		> 100	> 100
Med.Abs.Dev.			
Mean		> 100	> 100
Stdev.			

Compound	Virus			Cell	AV Method		AM Method	
Primary code	Species	Type	Strain	Type	Method	Type	Method	Type
BAVARIE1_0009	Enterovirus	71	BRCR	RD	Absorbance	MTS - 498nm	Microscopy	Tox scoring

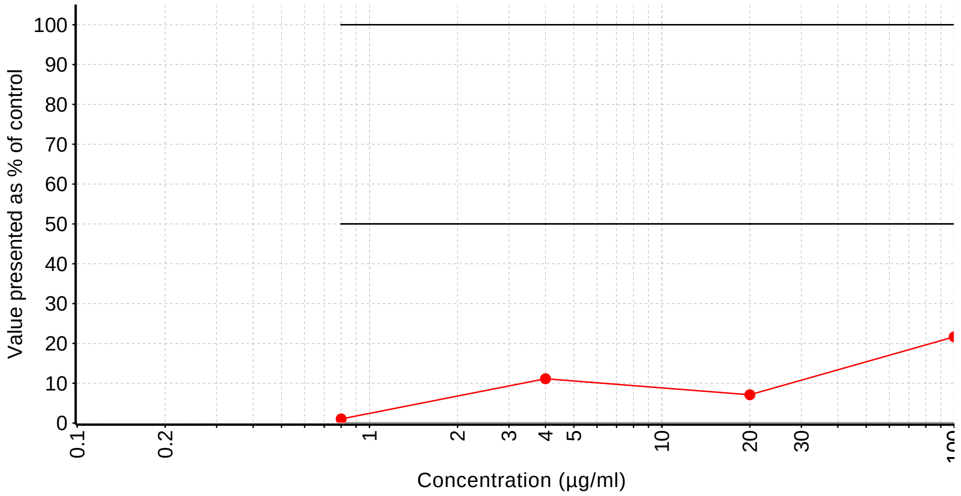

$CC50_{av}$		
EC50	>	100
EC90	>	100
SI_{av}		
SS_{av}		
TI_{av}		
Max %		21.64
[] Max		100
Approved AV		1
Interesting AV		0
Page		9 / 96

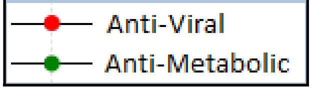

Export chart data to CSV

Summary values			
**Statistic**	**CC50**	**EC50**	**EC90**
Median		> 100	> 100
Med.Abs.Dev.			
Mean		> 100	> 100
Stdev.			

Compound	Virus			Cell	AV Method		AM Method	
Primary code	Species	Type	Strain	Type	Method	Type	Method	Type
BAVARIE1_0010	Enterovirus	71	BRCR	RD	Absorbance	MTS - 498nm	Microscopy	Tox scoring

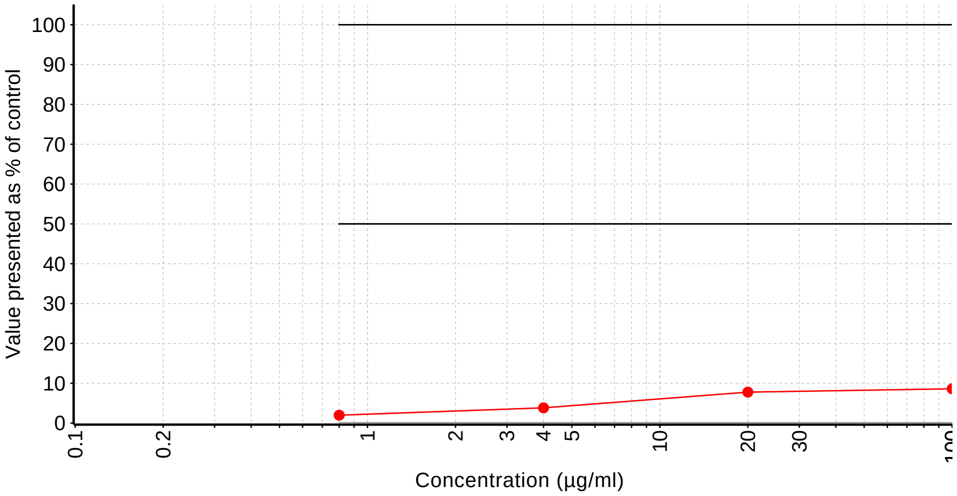

$CC50_{av}$		
EC50	>	100
EC90	>	100
SI_{av}		
SS_{av}		
TI_{av}		
Max %		8.61
[] Max		100
Approved AV		1
Interesting AV		0
Page		10 / 96

Anti-Viral
Anti-Metabolic

Export chart data to CSV

Summary values			
**Statistic**	**CC50**	**EC50**	**EC90**
Median		> 100	> 100
Med.Abs.Dev.			
Mean		> 100	> 100
Stdev.			

**Compound**	**Virus**			**Cell**	**AV Method**		**AM Method**	
Primary code	Species	Type	Strain	Type	Method	Type	Method	Type
BAVARIE1_0011	Enterovirus	71	BRCR	RD	Absorbance	MTS - 498nm	Microscopy	Tox scoring

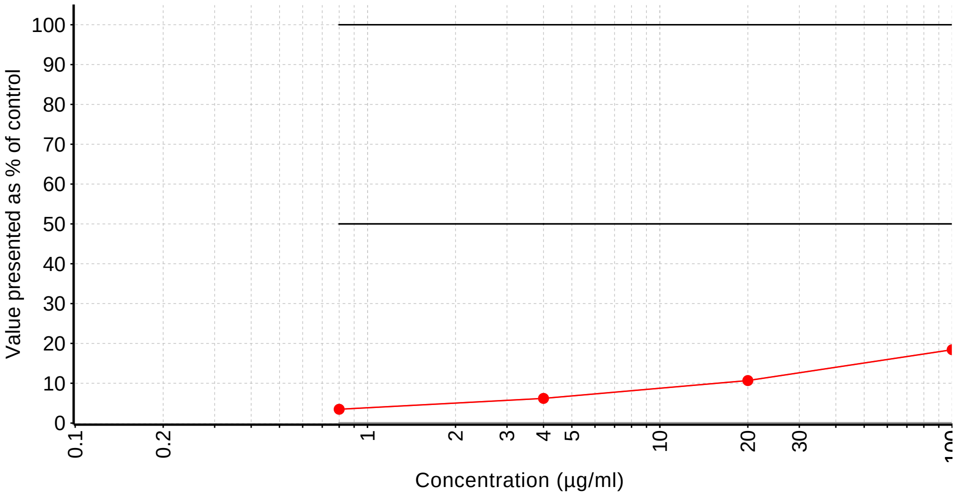

$CC50_{av}$		
EC50	>	100
EC90	>	100
SI_{av}		
SS_{av}		
TI_{av}		
Max %		18.42
[] Max		100
Approved AV		1
Interesting AV		0
Page		11 / 96

Anti-Viral
Anti-Metabolic

Export chart data to CSV

**Summary values**			
**Statistic**	**CC50**	**EC50**	**EC90**
Median		> 100	> 100
Med.Abs.Dev.			
Mean		> 100	> 100
Stdev.			

Compound	Virus			Cell	AV Method		AM Method	
Primary code	Species	Type	Strain	Type	Method	Type	Method	Type
BAVARIE1_0012	Enterovirus	71	BRCR	RD	Absorbance	MTS - 498nm	Microscopy	Tox scoring

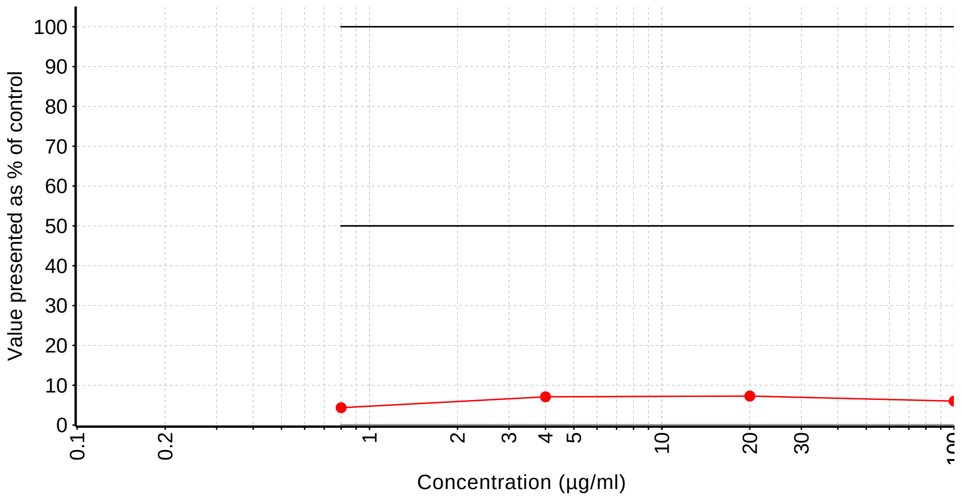

$CC50_{av}$		
EC50	>	100
EC90	>	100
SI_{av}		
SS_{av}		
TI_{av}		
Max %		7.28
[] Max		20
Approved AV		1
Interesting AV		0
Page		12 / 96

Anti-Viral
Anti-Metabolic

Export chart data to CSV

Summary values			
**Statistic**	**CC50**	**EC50**	**EC90**
Median		> 100	> 100
Med.Abs.Dev.			
Mean		> 100	> 100
Stdev.			

Compound	Virus			Cell	AV Method		AM Method	
Primary code	Species	Type	Strain	Type	Method	Type	Method	Type
BAVARIE1_0013	Enterovirus	71	BRCR	RD	Absorbance	MTS - 498nm	Microscopy	Tox scoring

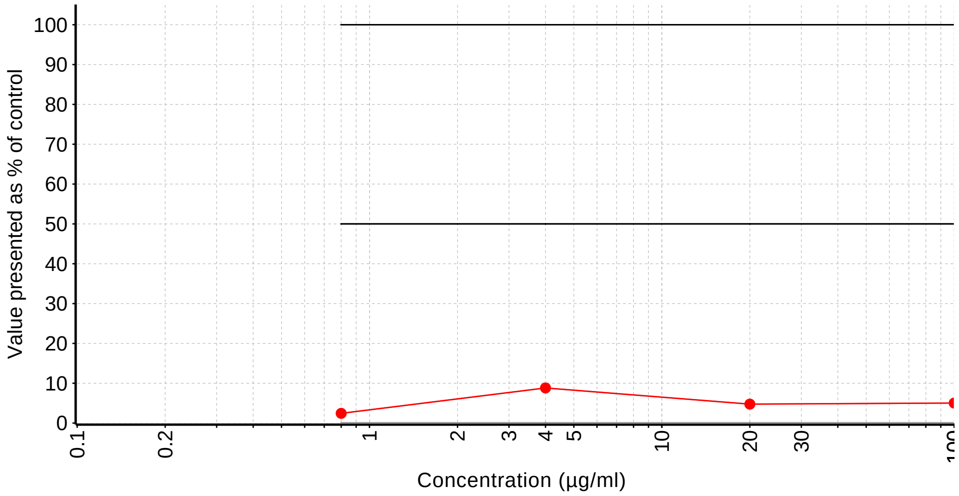

CC50$_{av}$		
EC50	>	100
EC90	>	100
SI$_{av}$		
SS$_{av}$		
TI$_{av}$		
Max %		8.82
[] Max		4
Approved AV		1
Interesting AV		0
Page		13 / 96

Anti-Viral
Anti-Metabolic

Export chart data to CSV

Summary values			
**Statistic**	**CC50**	**EC50**	**EC90**
Median		> 100	> 100
Med.Abs.Dev.			
Mean		> 100	> 100
Stdev.			

Compound	Virus			Cell	AV Method		AM Method	
Primary code	Species	Type	Strain	Type	Method	Type	Method	Type
BAVARIE1_0014	Enterovirus	71	BRCR	RD	Absorbance	MTS - 498nm	Microscopy	Tox scoring

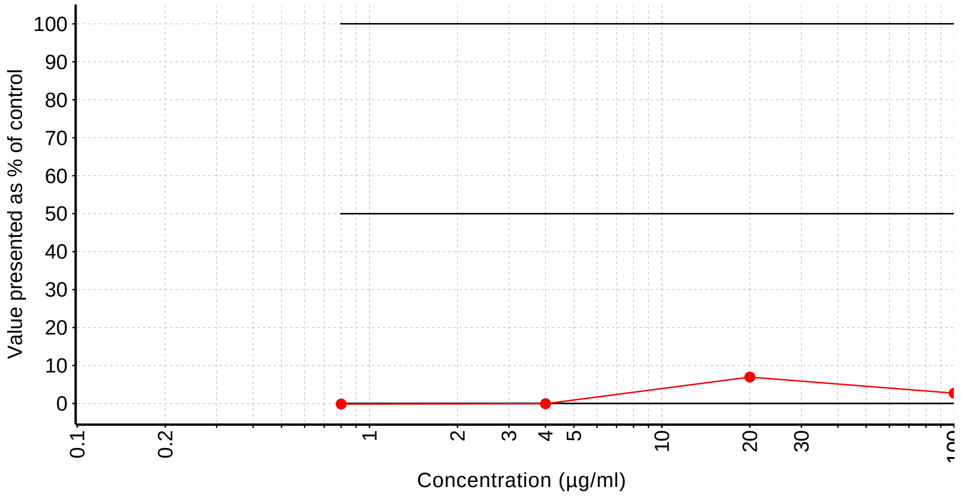

CC50$_{av}$		
EC50	>	100
EC90	>	100
SI$_{av}$		
SS$_{av}$		
TI$_{av}$		
Max %		6.94
[] Max		20
Approved AV		1
Interesting AV		0
Page		14 / 96

Anti-Viral
Anti-Metabolic

Export chart data to CSV

Summary values			
**Statistic**	**CC50**	**EC50**	**EC90**
Median		> 100	> 100
Med.Abs.Dev.			
Mean		> 100	> 100
Stdev.			

**Compound**	**Virus**			**Cell**	**AV Method**		**AM Method**	
Primary code	Species	Type	Strain	Type	Method	Type	Method	Type
BAVARIE1_0015	Enterovirus	71	BRCR	RD	Absorbance	MTS - 498nm	Microscopy	Tox scoring

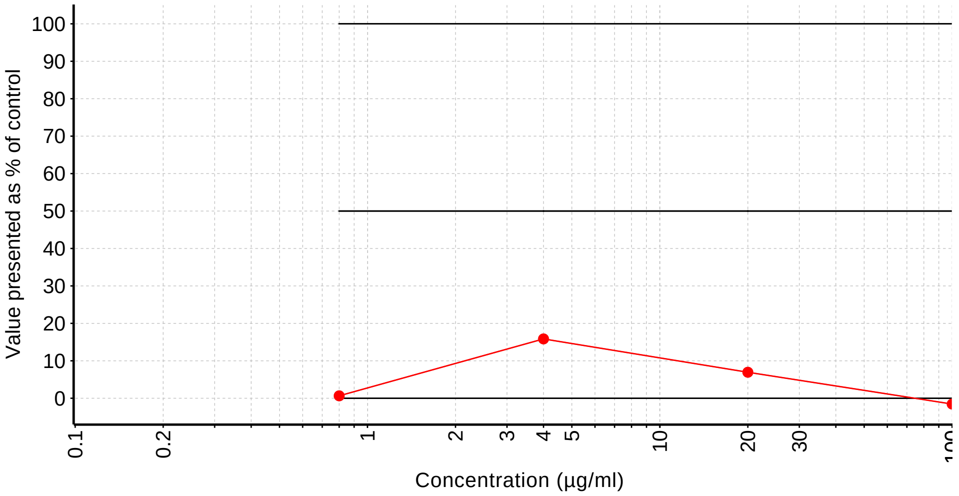

$CC50_{av}$		
EC50	>	100
EC90	>	100
SI_{av}		
SS_{av}		
TI_{av}		
Max %		15.85
[] Max		4
Approved AV		1
Interesting AV		0
Page		15 / 96

Anti-Viral
Anti-Metabolic

Export chart data to CSV

**Summary values**			
**Statistic**	**CC50**	**EC50**	**EC90**
Median		> 100	> 100
Med.Abs.Dev.			
Mean		> 100	> 100
Stdev.			

Compound	Virus			Cell	AV Method		AM Method	
Primary code	Species	Type	Strain	Type	Method	Type	Method	Type
BAVARIE1_0016	Enterovirus	71	BRCR	RD	Absorbance	MTS - 498nm	Microscopy	Tox scoring

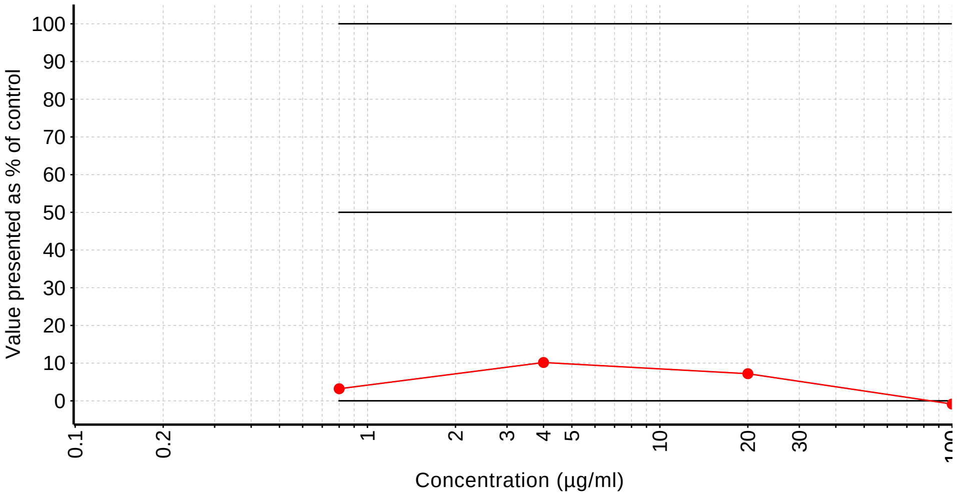

$CC50_{av}$		
EC50	>	100
EC90	>	100
SI_{av}		
SS_{av}		
TI_{av}		
Max %		10.17
[] Max		4
Approved AV		1
Interesting AV		0
Page		16 / 96

Anti-Viral
Anti-Metabolic

Export chart data to CSV

Summary values			
**Statistic**	**CC50**	**EC50**	**EC90**
Median		> 100	> 100
Med.Abs.Dev.			
Mean		> 100	> 100
Stdev.			

Compound	Virus			Cell	AV Method		AM Method	
Primary code	Species	Type	Strain	Type	Method	Type	Method	Type
BAVARIE1_0017	Enterovirus	71	BRCR	RD	Absorbance	MTS - 498nm	Microscopy	Tox scoring

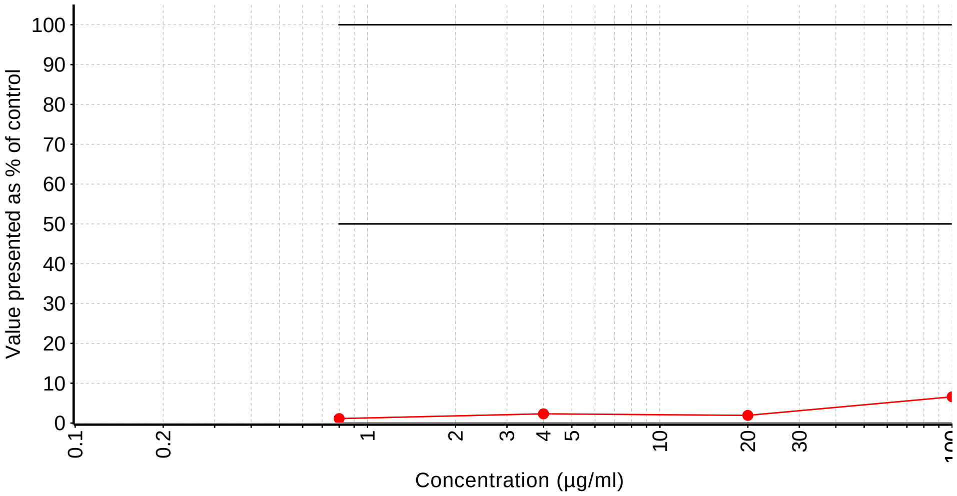

$CC50_{av}$		
EC50	>	100
EC90	>	100
SI_{av}		
SS_{av}		
TI_{av}		
Max %		6.59
[] Max		100
Approved AV		1
Interesting AV		0
Page		17 / 96

Anti-Viral
Anti-Metabolic

Export chart data to CSV

Summary values			
**Statistic**	**CC50**	**EC50**	**EC90**
Median		> 100	> 100
Med.Abs.Dev.			
Mean		> 100	> 100
Stdev.			

**Compound**	**Virus**			**Cell**	**AV Method**		**AM Method**	
Primary code	Species	Type	Strain	Type	Method	Type	Method	Type
BAVARIE1_0018	Enterovirus	71	BRCR	RD	Absorbance	MTS - 498nm	Microscopy	Tox scoring

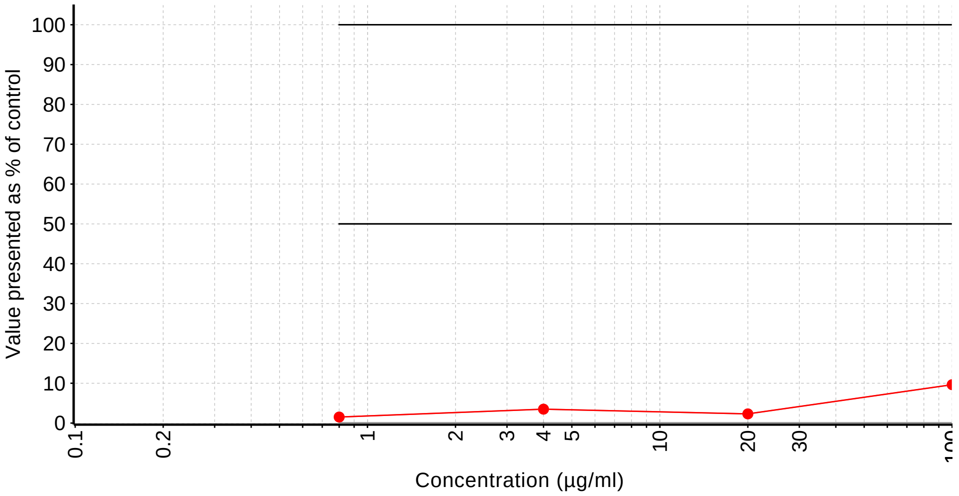

$CC50_{av}$		
EC50	>	100
EC90	>	100
SI_{av}		
SS_{av}		
TI_{av}		
Max %		9.64
[] Max		100
Approved AV		1
Interesting AV		0
Page		18 / 96

Anti-Viral
Anti-Metabolic

Export chart data to CSV

Summary values			
**Statistic**	**CC50**	**EC50**	**EC90**
Median		> 100	> 100
Med.Abs.Dev.			
Mean		> 100	> 100
Stdev.			

Compound	Virus			Cell	AV Method		AM Method	
Primary code	Species	Type	Strain	Type	Method	Type	Method	Type
BAVARI E1_0019	Enterovirus	71	BRCR	RD	Absorbance	MTS - 498nm	Microscopy	Tox scoring

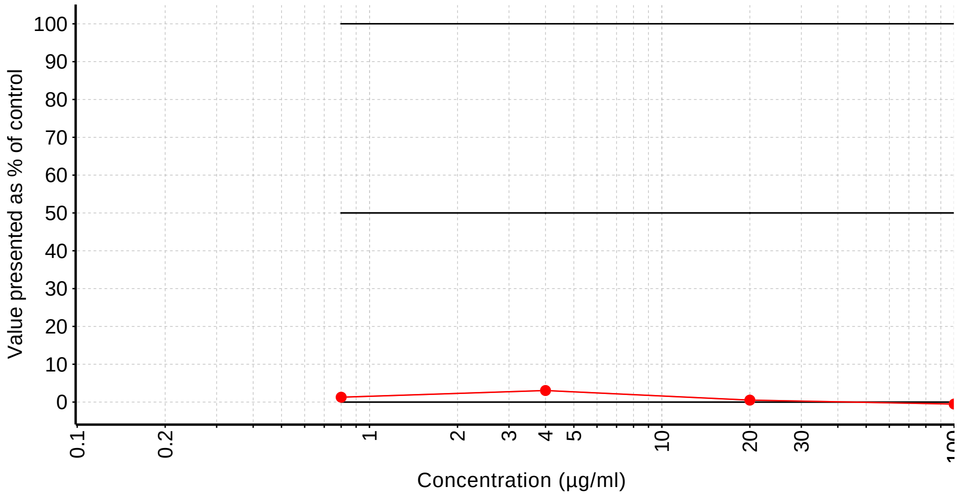

$CC50_{av}$		
EC50	>	100
EC90	>	100
SI_{av}		
SS_{av}		
TI_{av}		
Max %		3.05
[] Max		4
Approved AV		1
Interesting AV		0
Page		19 / 96

Anti-Viral
Anti-Metabolic

Export chart data to CSV

Summary values			
**Statistic**	**CC50**	**EC50**	**EC90**
Median		> 100	> 100
Med.Abs.Dev.			
Mean		> 100	> 100
Stdev.			

Compound	Virus			Cell	AV Method		AM Method	
Primary code	Species	Type	Strain	Type	Method	Type	Method	Type
BAVARIE1_0020	Enterovirus	71	BRCR	RD	Absorbance	MTS - 498nm	Microscopy	Tox scoring

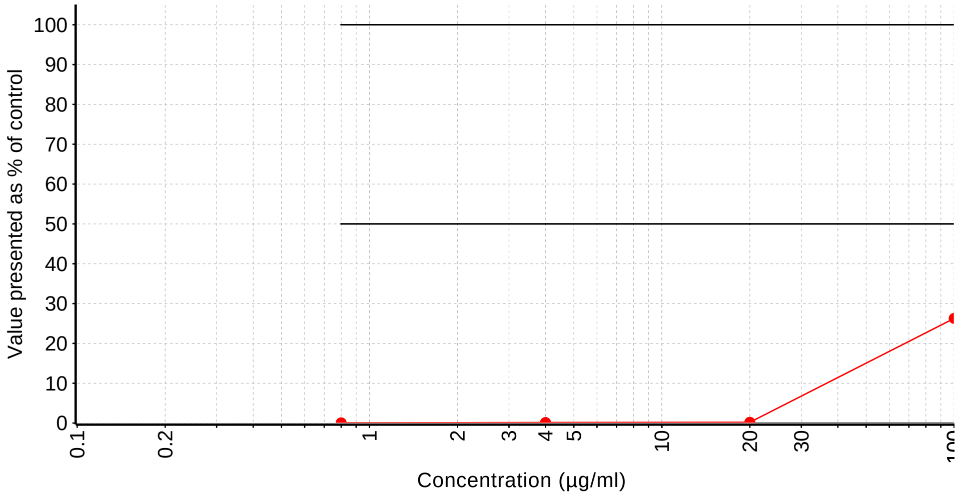

$CC50_{av}$		
EC50	>	100
EC90	>	100
SI_{av}		
SS_{av}		
TI_{av}		
Max %		26.28
[] Max		100
Approved AV		1
Interesting AV		0
Page		20 / 96

Anti-Viral
Anti-Metabolic

Export chart data to CSV

Summary values			
**Statistic**	**CC50**	**EC50**	**EC90**
Median		> 100	> 100
Med.Abs.Dev.			
Mean		> 100	> 100
Stdev.			

Compound	Virus			Cell	AV Method		AM Method	
Primary code	Species	Type	Strain	Type	Method	Type	Method	Type
BAVARIE1_0021	Enterovirus	71	BRCR	RD	Absorbance	MTS - 498nm	Microscopy	Tox scoring

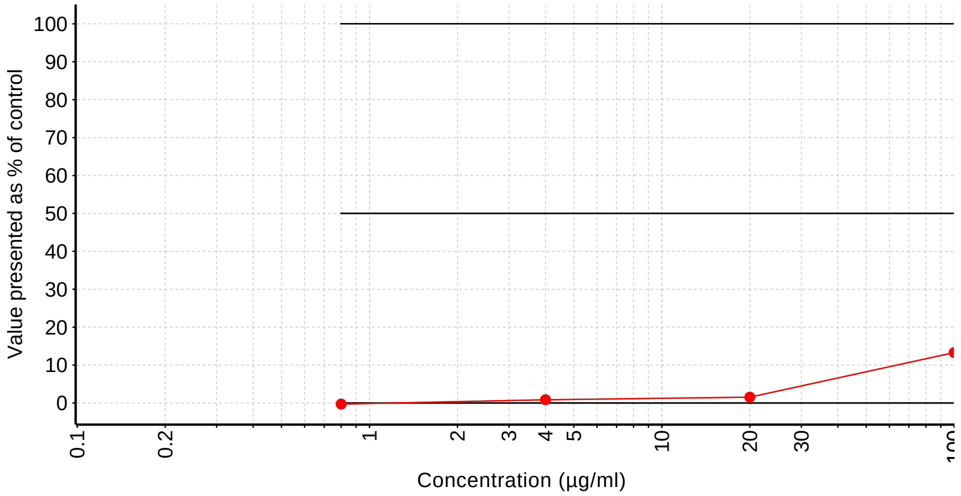

$CC50_{av}$		
EC50	>	100
EC90	>	100
SI_{av}		
SS_{av}		
TI_{av}		
Max %		13.30
[] Max		100
Approved AV		1
Interesting AV		0
Page		21 / 96

Anti-Viral
Anti-Metabolic

Export chart data to CSV

Summary values			
**Statistic**	**CC50**	**EC50**	**EC90**
Median		> 100	> 100
Med.Abs.Dev.			
Mean		> 100	> 100
Stdev.			

Compound	Virus			Cell	AV Method		AM Method	
Primary code	Species	Type	Strain	Type	Method	Type	Method	Type
BAVARIE1_0022	Enterovirus	71	BRCR	RD	Absorbance	MTS - 498nm	Microscopy	Tox scoring

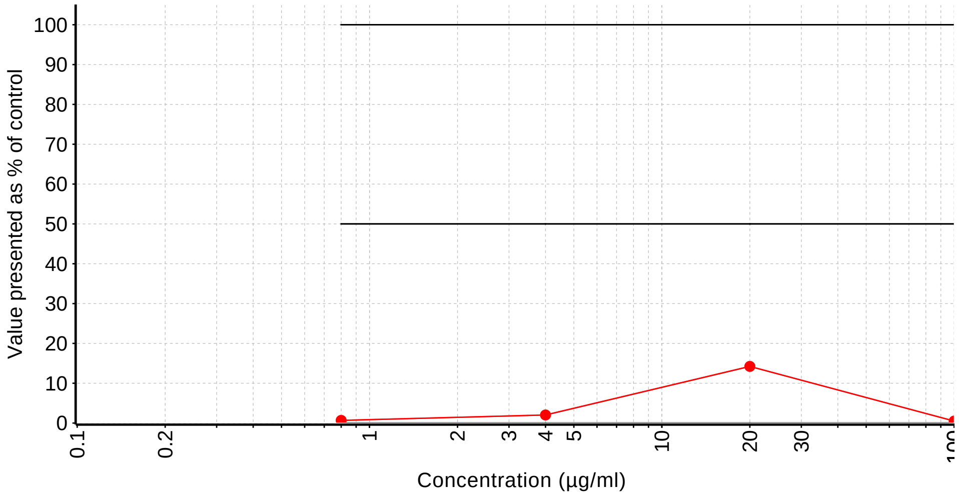

$CC50_{av}$		
EC50	>	100
EC90	>	100
SI_{av}		
SS_{av}		
TI_{av}		
Max %		14.23
[] Max		20
Approved AV		1
Interesting AV		0
Page		22 / 96

Anti-Viral
Anti-Metabolic

Export chart data to CSV

Summary values			
**Statistic**	**CC50**	**EC50**	**EC90**
Median		> 100	> 100
Med.Abs.Dev.			
Mean		> 100	> 100
Stdev.			

Compound	Virus			Cell	AV Method		AM Method	
Primary code	Species	Type	Strain	Type	Method	Type	Method	Type
BAVARIE1_0023	Enterovirus	71	BRCR	RD	Absorbance	MTS - 498nm	Microscopy	Tox scoring

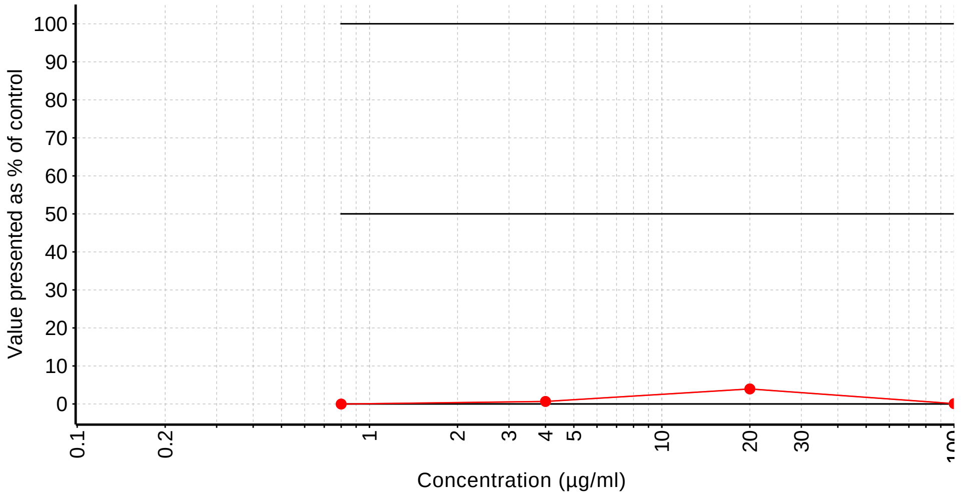

$CC50_{av}$		
EC50	>	100
EC90	>	100
SI_{av}		
SS_{av}		
TI_{av}		
Max %		3.95
[] Max		20
Approved AV		1
Interesting AV		0
Page		23 / 96

Anti-Viral
Anti-Metabolic

Export chart data to CSV

Summary values			
**Statistic**	**CC50**	**EC50**	**EC90**
Median		> 100	> 100
Med.Abs.Dev.			
Mean		> 100	> 100
Stdev.			

Compound	Virus			Cell	AV Method		AM Method	
Primary code	Species	Type	Strain	Type	Method	Type	Method	Type
BAVARIE1_0024	Enterovirus	71	BRCR	RD	Absorbance	MTS - 498nm	Microscopy	Tox scoring

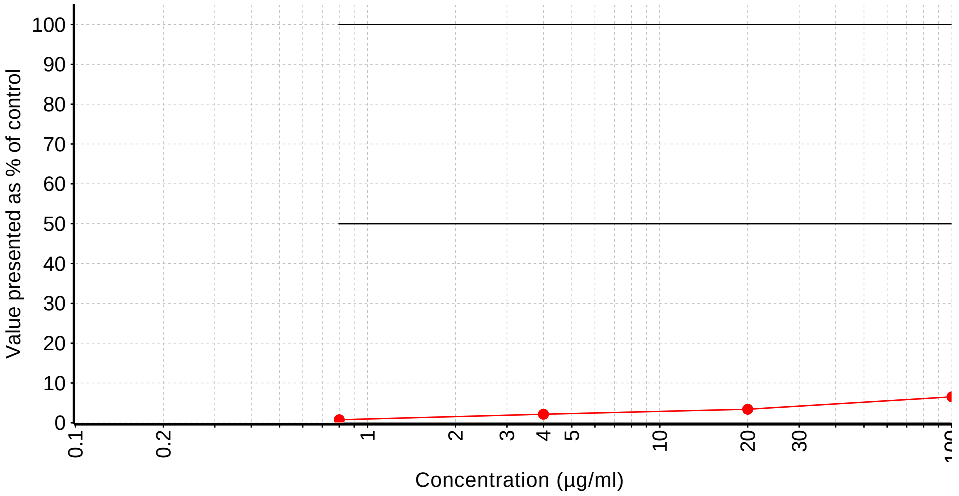

$CC50_{av}$		
EC50	>	100
EC90	>	100
SI_{av}		
SS_{av}		
TI_{av}		
Max %		6.52
[] Max		100
Approved AV		1
Interesting AV		0
Page		24 / 96

Anti-Viral
Anti-Metabolic

Export chart data to CSV

Summary values			
**Statistic**	**CC50**	**EC50**	**EC90**
Median		> 100	> 100
Med.Abs.Dev.			
Mean		> 100	> 100
Stdev.			

Compound	Virus			Cell	AV Method		AM Method	
Primary code	Species	Type	Strain	Type	Method	Type	Method	Type
BAVARI E1_0025	Enterovirus	71	BRCR	RD	Absorbance	MTS - 498nm	Microscopy	Tox scoring

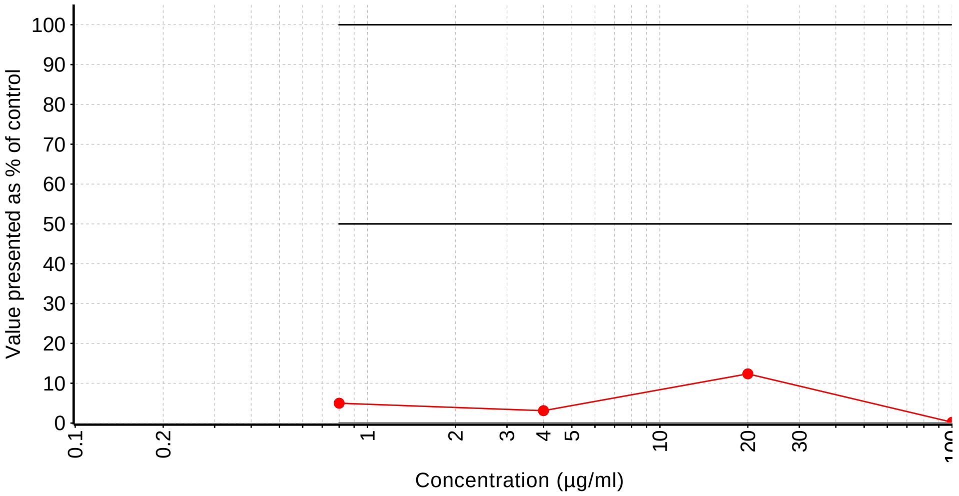

$CC50_{av}$		
EC50	>	100
EC90	>	100
SI_{av}		
SS_{av}		
TI_{av}		
Max %		12.36
[] Max		20
Approved AV		1
Interesting AV		0
Page		25 / 96

Anti-Viral
Anti-Metabolic

Export chart data to CSV

Summary values			
**Statistic**	**CC50**	**EC50**	**EC90**
Median		> 100	> 100
Med.Abs.Dev.			
Mean		> 100	> 100
Stdev.			

Compound	Virus			Cell	AV Method		AM Method	
Primary code	Species	Type	Strain	Type	Method	Type	Method	Type
BAVARIE1_0026	Enterovirus	71	BRCR	RD	Absorbance	MTS - 498nm	Microscopy	Tox scoring

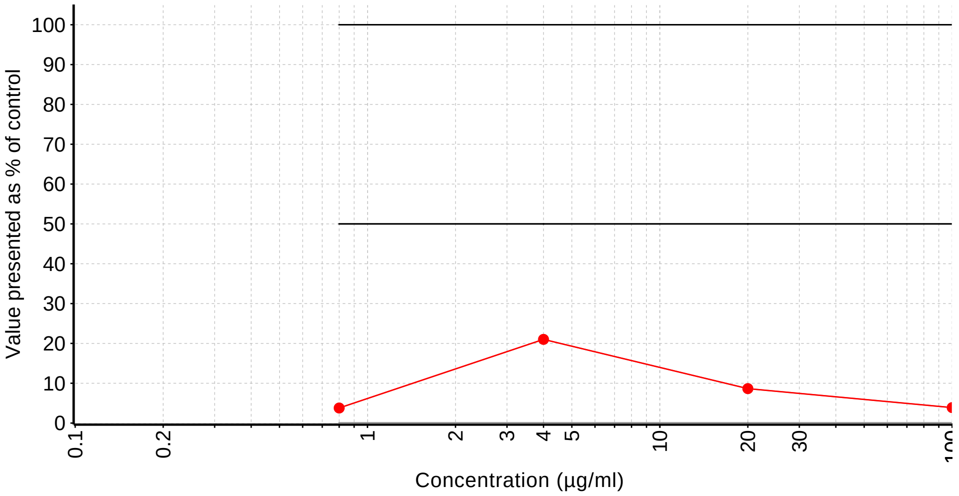

$CC50_{av}$	
EC50	
EC90	
SI_{av}	
SS_{av}	
TI_{av}	
Max %	21.01
[] Max	4
Approved AV	1
Interesting AV	0
Page	26 / 96

Anti-Viral
Anti-Metabolic

Export chart data to CSV

Summary values			
**Statistic**	**CC50**	**EC50**	**EC90**
Median			
Med.Abs.Dev.			
Mean			
Stdev.			

Compound	Virus			Cell	AV Method		AM Method	
Primary code	Species	Type	Strain	Type	Method	Type	Method	Type
BAVARIE1_0027	Enterovirus	71	BRCR	RD	Absorbance	MTS - 498nm	Microscopy	Tox scoring

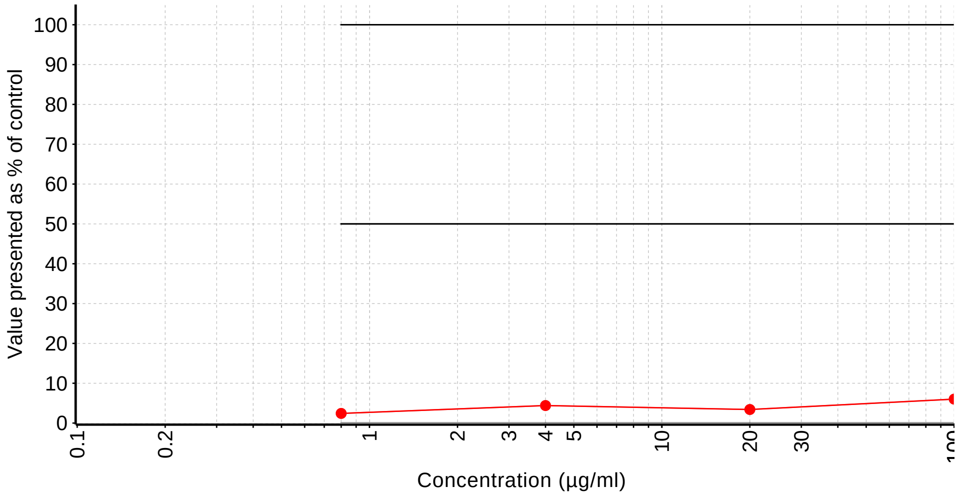

CC50$_{av}$		
EC50	>	100
EC90	>	100
SI$_{av}$		
SS$_{av}$		
TI$_{av}$		
Max %		6.01
[] Max		100
Approved AV		1
Interesting AV		0
Page		27 / 96

Anti-Viral
Anti-Metabolic

Export chart data to CSV

Summary values			
**Statistic**	**CC50**	**EC50**	**EC90**
Median		> 100	> 100
Med.Abs.Dev.			
Mean		> 100	> 100
Stdev.			

Compound	Virus			Cell	AV Method		AM Method	
Primary code	Species	Type	Strain	Type	Method	Type	Method	Type
BAVARIE1_0028	Enterovirus	71	BRCR	RD	Absorbance	MTS - 498nm	Microscopy	Tox scoring

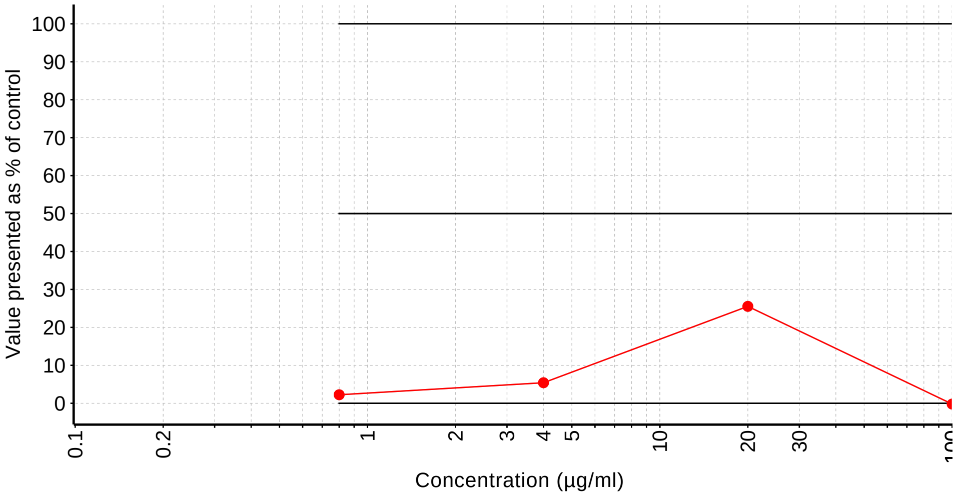

$CC50_{av}$	
EC50	
EC90	
SI_{av}	
SS_{av}	
TI_{av}	
Max %	25.54
[] Max	20
Approved AV	1
Interesting AV	0
Page	28 / 96

Anti-Viral
Anti-Metabolic

Export chart data to CSV

Summary values			
**Statistic**	**CC50**	**EC50**	**EC90**
Median			
Med.Abs.Dev.			
Mean			
Stdev.			

Compound	Virus			Cell	AV Method		AM Method	
Primary code	Species	Type	Strain	Type	Method	Type	Method	Type
BAVARIE1_0029	Enterovirus	71	BRCR	RD	Absorbance	MTS - 498nm	Microscopy	Tox scoring

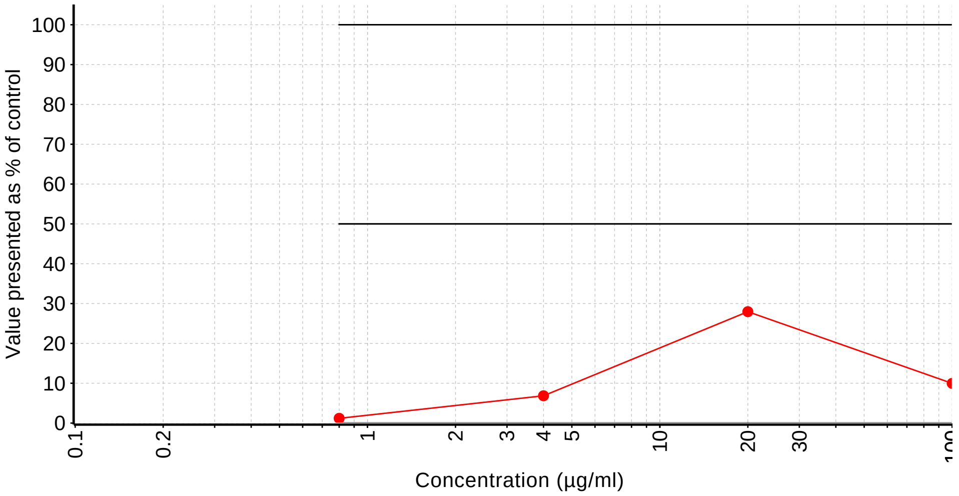

$CC50_{av}$	
EC50	
EC90	
SI_{av}	
SS_{av}	
TI_{av}	
Max %	27.96
[] Max	20
Approved AV	1
Interesting AV	0
Page	29 / 96

Anti-Viral
Anti-Metabolic

Export chart data to CSV

Summary values			
**Statistic**	**CC50**	**EC50**	**EC90**
Median			
Med.Abs.Dev.			
Mean			
Stdev.			

Compound	Virus			Cell	AV Method		AM Method	
Primary code	Species	Type	Strain	Type	Method	Type	Method	Type
BAVARIE1_0030	Enterovirus	71	BRCR	RD	Absorbance	MTS - 498nm	Microscopy	Tox scoring

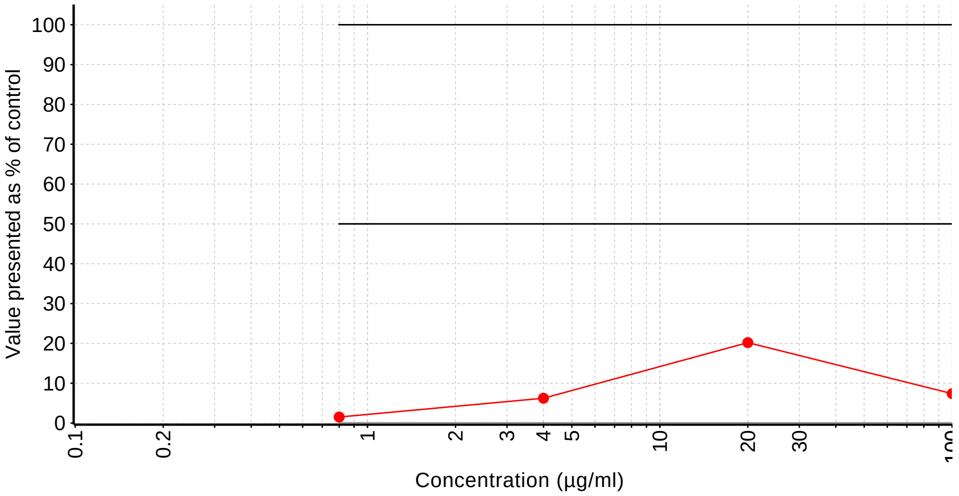

$CC50_{av}$	
EC50	
EC90	
SI_{av}	
SS_{av}	
TI_{av}	
Max %	20.20
[] Max	20
Approved AV	1
Interesting AV	0
Page	30 / 96

Anti-Viral
Anti-Metabolic

Export chart data to CSV

Summary values			
**Statistic**	**CC50**	**EC50**	**EC90**
Median			
Med.Abs.Dev.			
Mean			
Stdev.			

Compound	Virus			Cell	AV Method		AM Method	
Primary code	Species	Type	Strain	Type	Method	Type	Method	Type
BAVARIE1_0031	Enterovirus	71	BRCR	RD	Absorbance	MTS - 498nm	Microscopy	Tox scoring

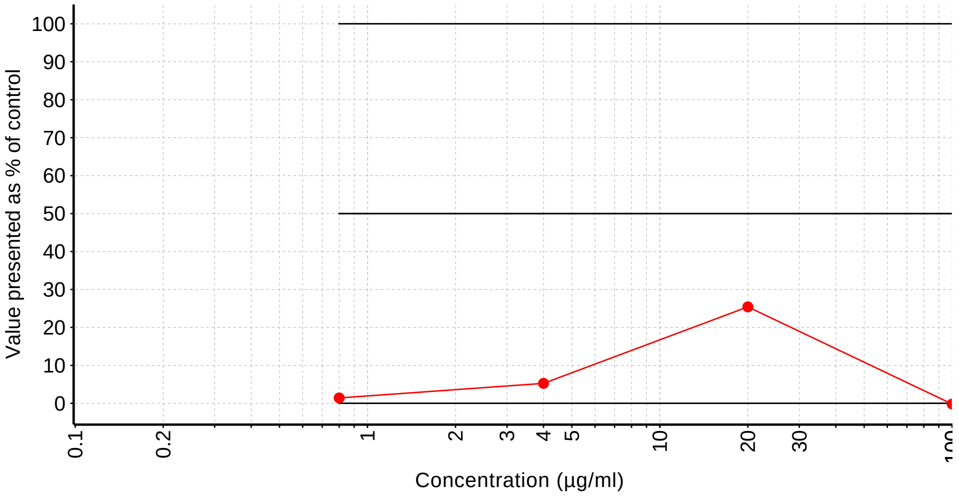

$CC50_{av}$	
EC50	
EC90	
SI_{av}	
SS_{av}	
TI_{av}	
Max %	25.41
[] Max	20
Approved AV	1
Interesting AV	0
Page	31 / 96

Anti-Viral
Anti-Metabolic

Export chart data to CSV

Summary values			
**Statistic**	**CC50**	**EC50**	**EC90**
Median			
Med.Abs.Dev.			
Mean			
Stdev.			

Compound	Virus			Cell	AV Method		AM Method	
Primary code	Species	Type	Strain	Type	Method	Type	Method	Type
BAVARIE1_0032	Enterovirus	71	BRCR	RD	Absorbance	MTS - 498nm	Microscopy	Tox scoring

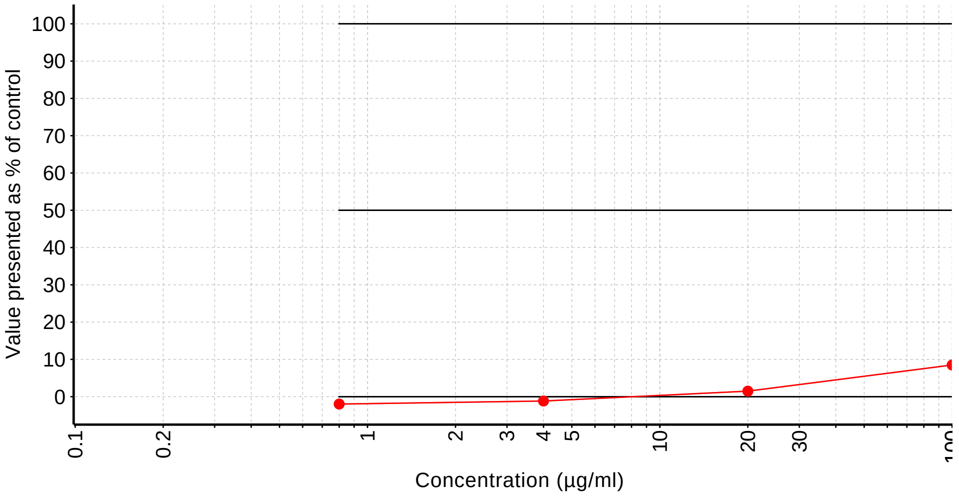

$CC50_{av}$		
EC50	>	100
EC90	>	100
SI_{av}		
SS_{av}		
TI_{av}		
Max %		8.49
[] Max		100
Approved AV		1
Interesting AV		0
Page		32 / 96

Anti-Viral
Anti-Metabolic

Export chart data to CSV

Summary values			
**Statistic**	**CC50**	**EC50**	**EC90**
Median		> 100	> 100
Med.Abs.Dev.			
Mean		> 100	> 100
Stdev.			

Compound	Virus			Cell	AV Method		AM Method	
Primary code	Species	Type	Strain	Type	Method	Type	Method	Type
BAVARIE1_0033	Enterovirus	71	BRCR	RD	Absorbance	MTS - 498nm	Microscopy	Tox scoring

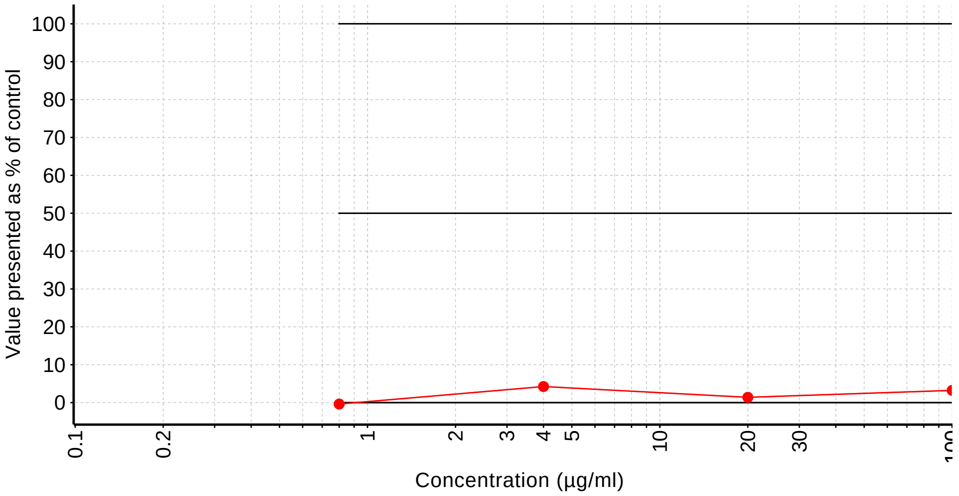

$CC50_{av}$		
EC50	>	100
EC90	>	100
SI_{av}		
SS_{av}		
TI_{av}		
Max %		4.24
[] Max		4
Approved AV		1
Interesting AV		0
Page		33 / 96

Anti-Viral
Anti-Metabolic

Export chart data to CSV

Summary values			
**Statistic**	**CC50**	**EC50**	**EC90**
Median		> 100	> 100
Med.Abs.Dev.			
Mean		> 100	> 100
Stdev.			

**Compound**	**Virus**			**Cell**	**AV Method**		**AM Method**	
Primary code	Species	Type	Strain	Type	Method	Type	Method	Type
BAVARIE1_0034	Enterovirus	71	BRCR	RD	Absorbance	MTS - 498nm	Microscopy	Tox scoring

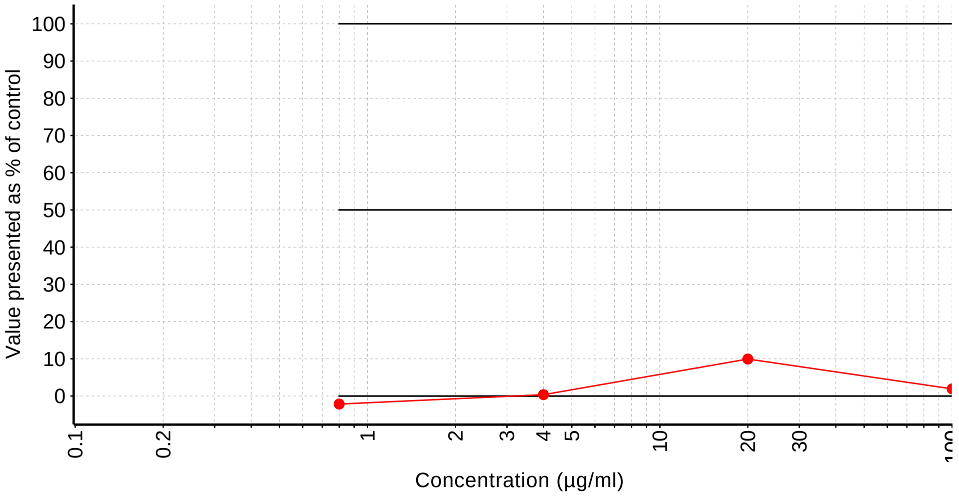

$CC50_{av}$		
EC50	>	100
EC90	>	100
SI_{av}		
SS_{av}		
TI_{av}		
Max %		9.94
[] Max		20
Approved AV		1
Interesting AV		0
Page		34 / 96

Anti-Viral
Anti-Metabolic

Export chart data to CSV

Summary values			
**Statistic**	**CC50**	**EC50**	**EC90**
Median		> 100	> 100
Med.Abs.Dev.			
Mean		> 100	> 100
Stdev.			

Compound	Virus			Cell	AV Method		AM Method	
Primary code	Species	Type	Strain	Type	Method	Type	Method	Type
BAVARIE1_0035	Enterovirus	71	BRCR	RD	Absorbance	MTS - 498nm	Microscopy	Tox scoring

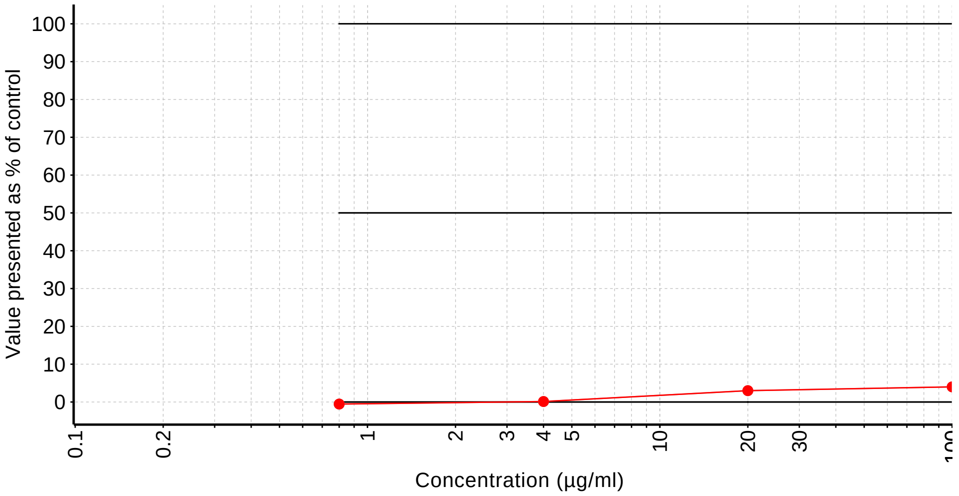

$CC50_{av}$		
EC50	>	100
EC90	>	100
SI_{av}		
SS_{av}		
TI_{av}		
Max %		4.00
[] Max		100
Approved AV		1
Interesting AV		0
Page		35 / 96

Anti-Viral
Anti-Metabolic

Export chart data to CSV

Summary values			
**Statistic**	**CC50**	**EC50**	**EC90**
Median		> 100	> 100
Med.Abs.Dev.			
Mean		> 100	> 100
Stdev.			

Compound	Virus			Cell	AV Method		AM Method	
Primary code	Species	Type	Strain	Type	Method	Type	Method	Type
BAVARIE1_0036	Enterovirus	71	BRCR	RD	Absorbance	MTS - 498nm	Microscopy	Tox scoring

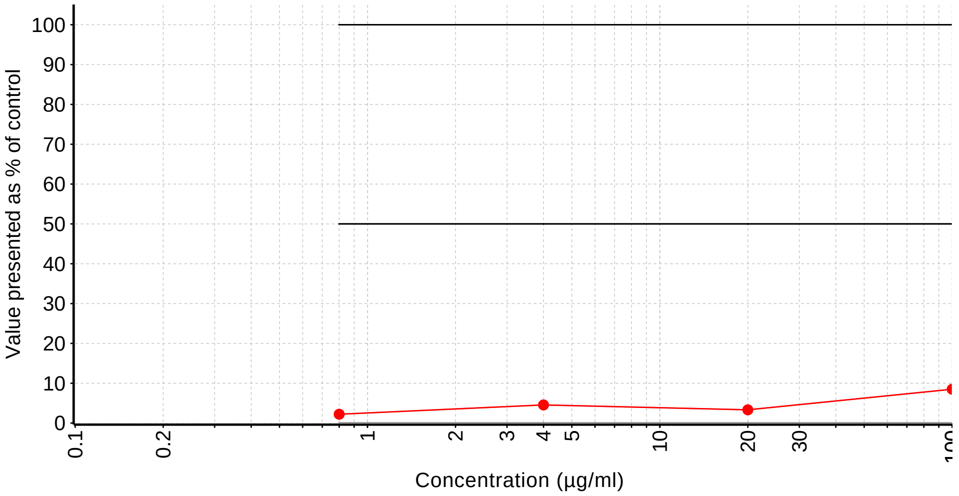

$CC50_{av}$		
EC50	>	100
EC90	>	100
SI_{av}		
SS_{av}		
TI_{av}		
Max %		8.49
[] Max		100
Approved AV		1
Interesting AV		0
Page		36 / 96

Anti-Viral
Anti-Metabolic

Export chart data to CSV

Summary values			
**Statistic**	**CC50**	**EC50**	**EC90**
Median		> 100	> 100
Med.Abs.Dev.			
Mean		> 100	> 100
Stdev.			

**Compound**	**Virus**			**Cell**	**AV Method**		**AM Method**	
Primary code	Species	Type	Strain	Type	Method	Type	Method	Type
BAVARIE1_0037	Enterovirus	71	BRCR	RD	Absorbance	MTS - 498nm	Microscopy	Tox scoring

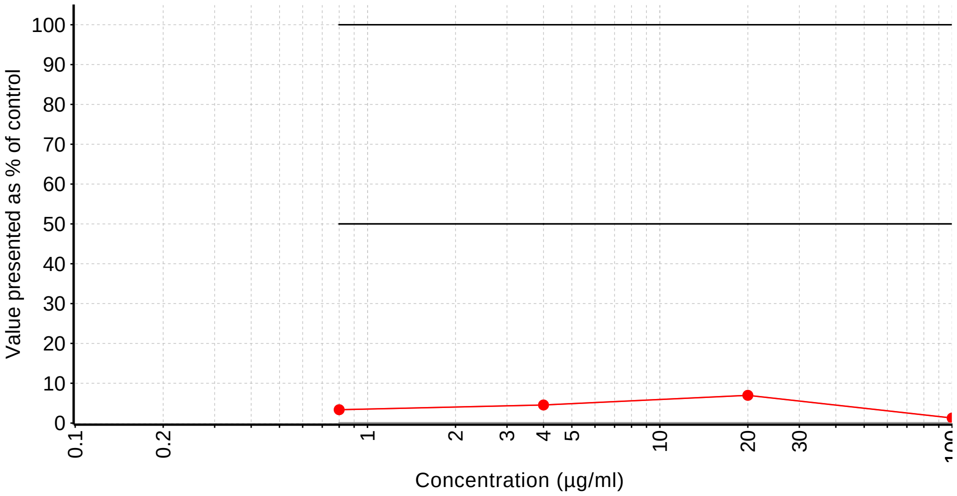

$CC50_{av}$		
EC50	>	100
EC90	>	100
SI_{av}		
SS_{av}		
TI_{av}		
Max %		6.97
[] Max		20
Approved AV		1
Interesting AV		0
Page		37 / 96

Anti-Viral
Anti-Metabolic

Export chart data to CSV

**Summary values**			
**Statistic**	**CC50**	**EC50**	**EC90**
Median		> 100	> 100
Med.Abs.Dev.			
Mean		> 100	> 100
Stdev.			

Compound	Virus			Cell	AV Method		AM Method	
Primary code	Species	Type	Strain	Type	Method	Type	Method	Type
BAVARIE1_0038	Enterovirus	71	BRCR	RD	Absorbance	MTS - 498nm	Microscopy	Tox scoring

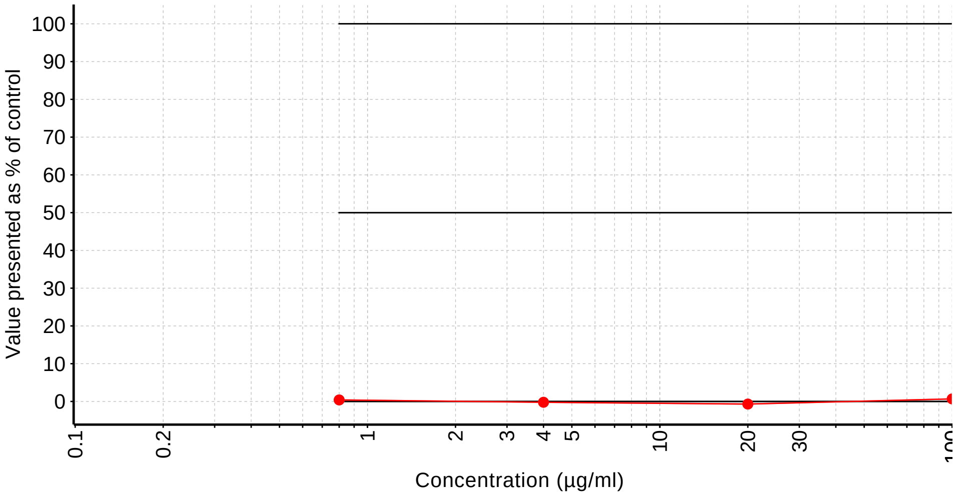

$CC50_{av}$		
EC50	>	100
EC90	>	100
SI_{av}		
SS_{av}		
TI_{av}		
Max %		0.68
[] Max		100
Approved AV		1
Interesting AV		0
Page		38 / 96

Anti-Viral
Anti-Metabolic

Export chart data to CSV

Summary values			
**Statistic**	**CC50**	**EC50**	**EC90**
Median		> 100	> 100
Med.Abs.Dev.			
Mean		> 100	> 100
Stdev.			

Compound	Virus			Cell	AV Method		AM Method	
Primary code	Species	Type	Strain	Type	Method	Type	Method	Type
BAVARIE1_0039	Enterovirus	71	BRCR	RD	Absorbance	MTS - 498nm	Microscopy	Tox scoring

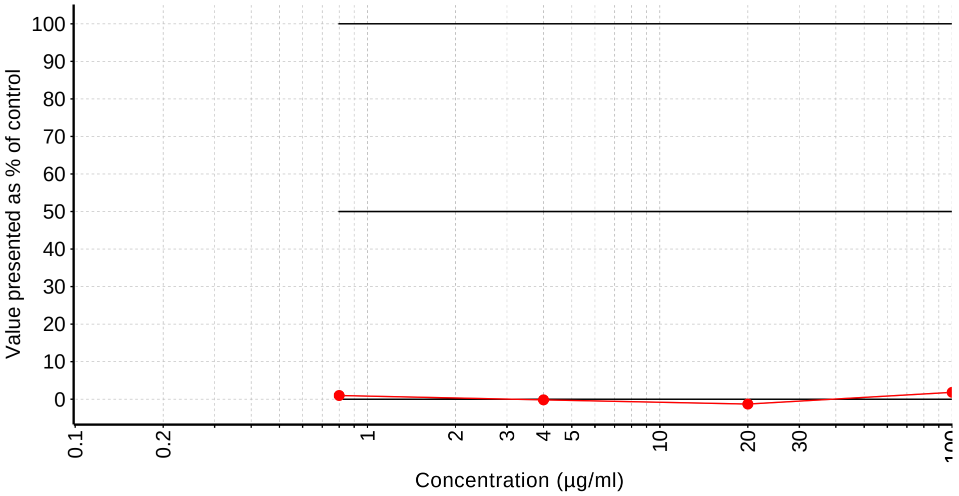

$CC50_{av}$		
EC50	>	100
EC90	>	100
SI_{av}		
SS_{av}		
TI_{av}		
Max %		1.83
[] Max		100
Approved AV		1
Interesting AV		0
Page		39 / 96

Anti-Viral
Anti-Metabolic

Export chart data to CSV

Summary values			
**Statistic**	**CC50**	**EC50**	**EC90**
Median		> 100	> 100
Med.Abs.Dev.			
Mean		> 100	> 100
Stdev.			

Compound	Virus			Cell	AV Method		AM Method	
Primary code	Species	Type	Strain	Type	Method	Type	Method	Type
BAVARIE1_0040	Enterovirus	71	BRCR	RD	Absorbance	MTS - 498nm	Microscopy	Tox scoring

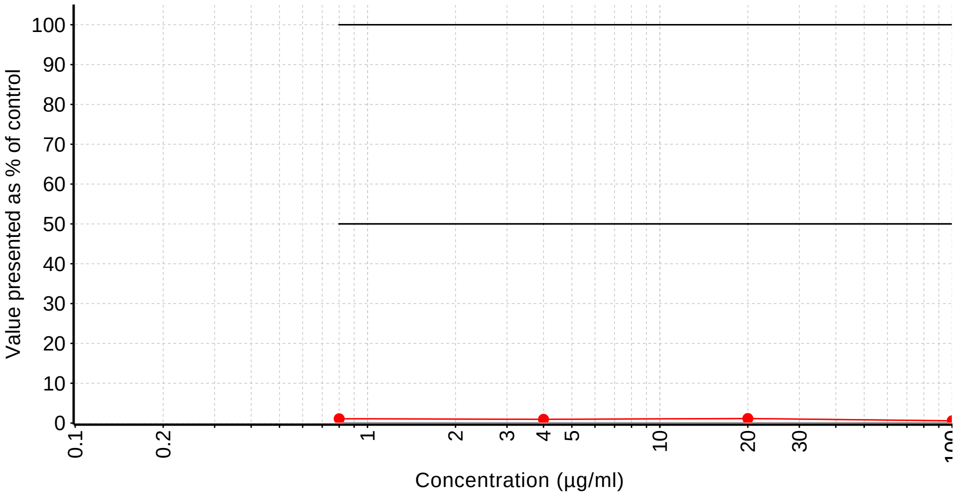

$CC50_{av}$		
EC50	>	100
EC90	>	100
SI_{av}		
SS_{av}		
TI_{av}		
Max %		1.14
[] Max		20
Approved AV		1
Interesting AV		0
Page		40 / 96

Anti-Viral
Anti-Metabolic

Export chart data to CSV

Summary values			
**Statistic**	**CC50**	**EC50**	**EC90**
Median		> 100	> 100
Med.Abs.Dev.			
Mean		> 100	> 100
Stdev.			

Compound	Virus			Cell	AV Method		AM Method	
Primary code	Species	Type	Strain	Type	Method	Type	Method	Type
BAVARIE1_0041	Enterovirus	71	BRCR	RD	Absorbance	MTS - 498nm	Microscopy	Tox scoring

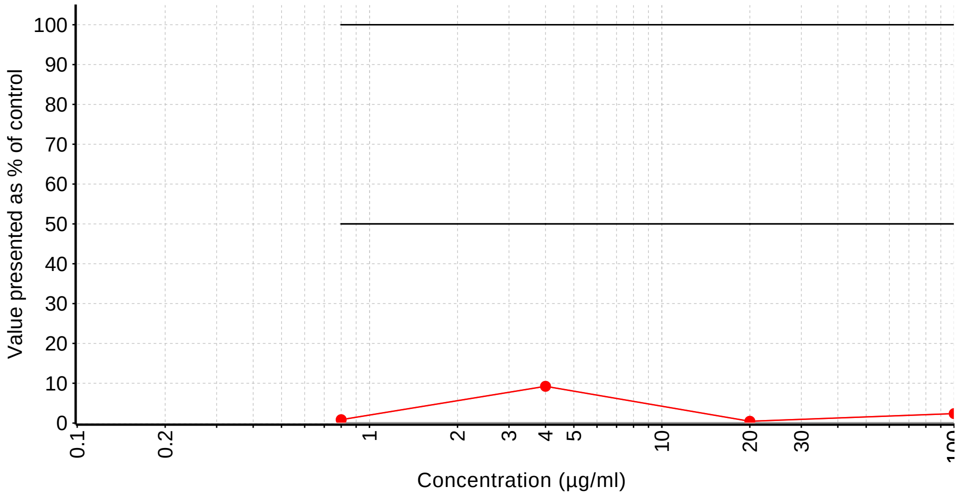

$CC50_{av}$		
EC50	>	100
EC90	>	100
SI_{av}		
SS_{av}		
TI_{av}		
Max %		9.23
[] Max		4
Approved AV		1
Interesting AV		0
Page		41 / 96

Anti-Viral
Anti-Metabolic

Export chart data to CSV

Summary values			
**Statistic**	**CC50**	**EC50**	**EC90**
Median		> 100	> 100
Med.Abs.Dev.			
Mean		> 100	> 100
Stdev.			

Compound	Virus			Cell	AV Method		AM Method	
Primary code	Species	Type	Strain	Type	Method	Type	Method	Type
BAVARIE1_0042	Enterovirus	71	BRCR	RD	Absorbance	MTS - 498nm	Microscopy	Tox scoring

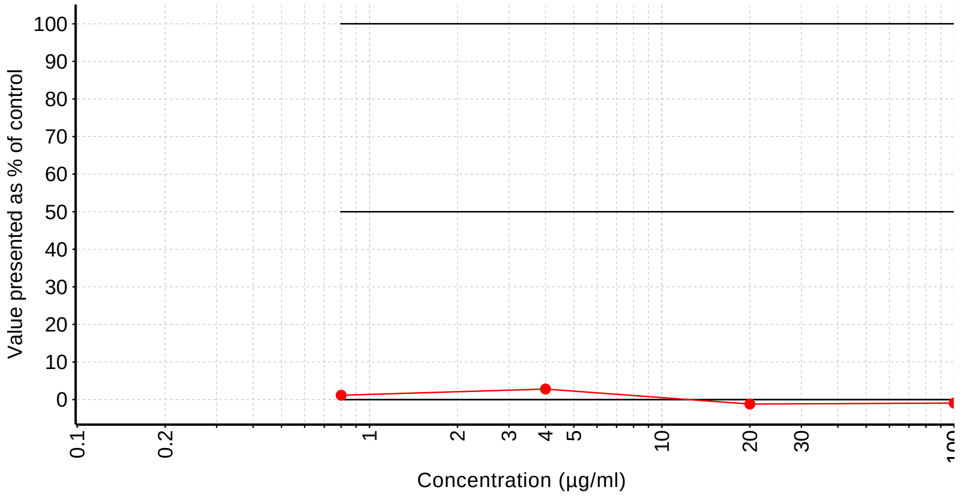

$CC50_{av}$		
EC50	>	100
EC90	>	100
SI_{av}		
SS_{av}		
TI_{av}		
Max %		2.80
[] Max		4
Approved AV		1
Interesting AV		0
Page		42 / 96

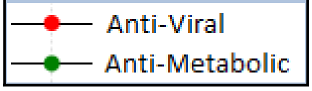

Export chart data to CSV

Summary values

Statistic	CC50	EC50	EC90
Median		> 100	> 100
Med.Abs.Dev.			
Mean		> 100	> 100
Stdev.			

Compound	Virus			Cell	AV Method		AM Method	
Primary code	Species	Type	Strain	Type	Method	Type	Method	Type
BAVARI E1_0043	Enterovirus	71	BRCR	RD	Absorbance	MTS - 498nm	Microscopy	Tox scoring

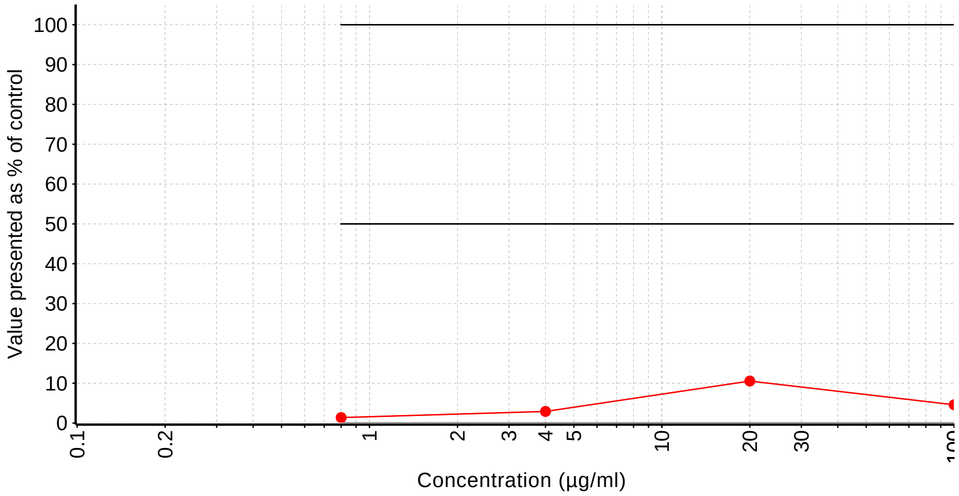

CC50av		
EC50	>	100
EC90	>	100
SIav		
SSav		
TIav		
Max %		10.55
[] Max		20
Approved AV		1
Interesting AV		0
Page		43 / 96

Anti-Viral
Anti-Metabolic

Export chart data to CSV

Summary values			
**Statistic**	**CC50**	**EC50**	**EC90**
Median		> 100	> 100
Med.Abs.Dev.			
Mean		> 100	> 100
Stdev.			

Compound	Virus			Cell	AV Method		AM Method	
Primary code	Species	Type	Strain	Type	Method	Type	Method	Type
BAVARIE1_0044	Enterovirus	71	BRCR	RD	Absorbance	MTS - 498nm	Microscopy	Tox scoring

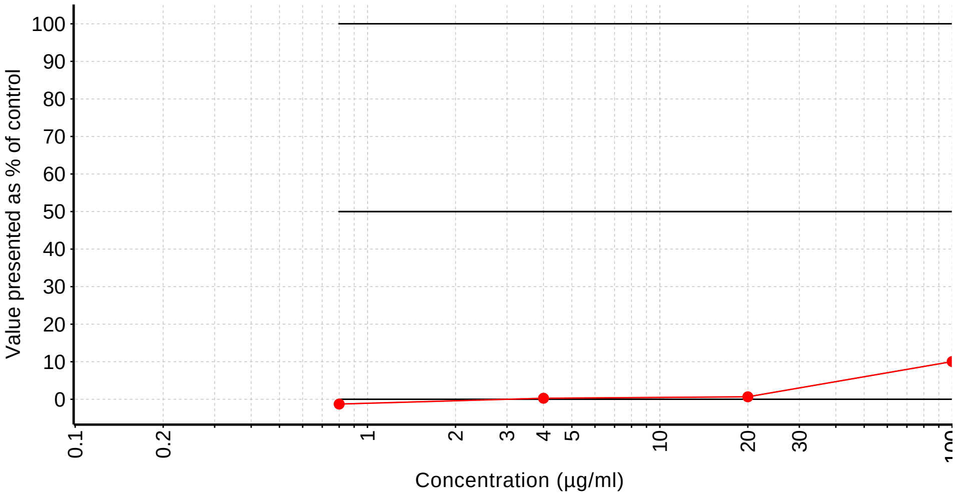

$CC50_{av}$		
EC50	>	100
EC90	>	100
SI_{av}		
SS_{av}		
TI_{av}		
Max %		10.04
[] Max		100
Approved AV		1
Interesting AV		0
Page		44 / 96

Anti-Viral
Anti-Metabolic

Export chart data to CSV

Summary values			
**Statistic**	**CC50**	**EC50**	**EC90**
Median		> 100	> 100
Med.Abs.Dev.			
Mean		> 100	> 100
Stdev.			

Compound	Virus			Cell	AV Method		AM Method	
Primary code	Species	Type	Strain	Type	Method	Type	Method	Type
BAVARIE1_0045	Enterovirus	71	BRCR	RD	Absorbance	MTS - 498nm	Microscopy	Tox scoring

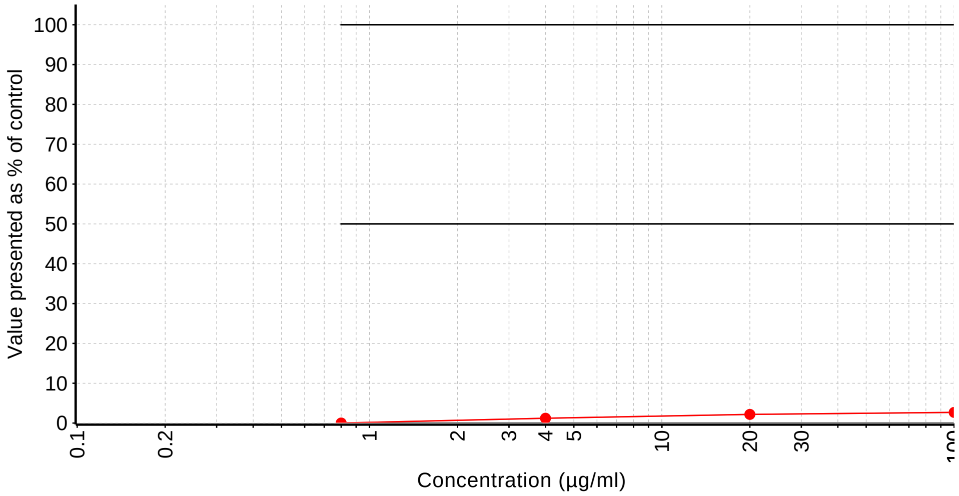

$CC50_{av}$		
EC50	>	100
EC90	>	100
SI_{av}		
SS_{av}		
TI_{av}		
Max %		2.68
[] Max		100
Approved AV		1
Interesting AV		0
Page		45 / 96

Anti-Viral
Anti-Metabolic

Export chart data to CSV

Summary values			
**Statistic**	**CC50**	**EC50**	**EC90**
Median		> 100	> 100
Med.Abs.Dev.			
Mean		> 100	> 100
Stdev.			

Compound	Virus			Cell	AV Method		AM Method	
Primary code	Species	Type	Strain	Type	Method	Type	Method	Type
BAVARIE1_0046	Enterovirus	71	BRCR	RD	Absorbance	MTS - 498nm	Microscopy	Tox scoring

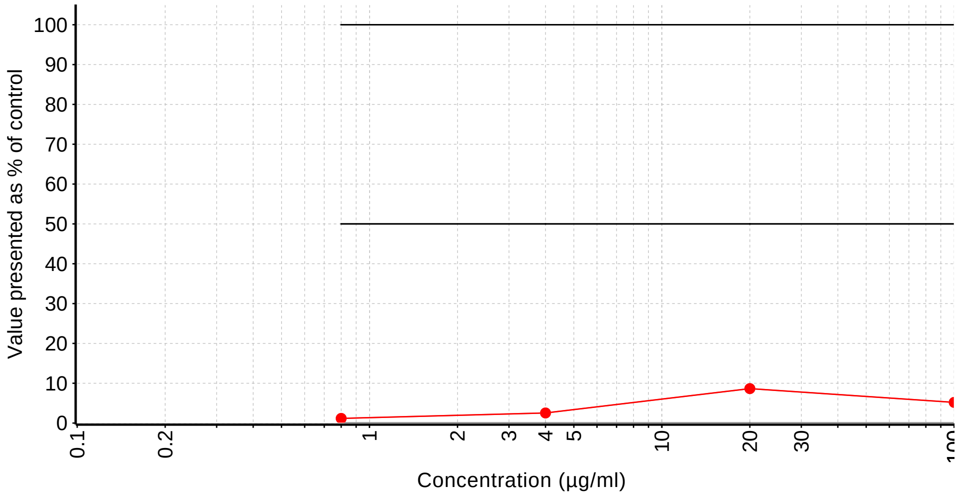

$CC50_{av}$		
EC50	>	100
EC90	>	100
SI_{av}		
SS_{av}		
TI_{av}		
Max %		8.65
[] Max		20
Approved AV		1
Interesting AV		0
Page		46 / 96

Anti-Viral
Anti-Metabolic

Export chart data to CSV

Summary values			
**Statistic**	**CC50**	**EC50**	**EC90**
Median		> 100	> 100
Med.Abs.Dev.			
Mean		> 100	> 100
Stdev.			

Compound	Virus			Cell	AV Method		AM Method	
Primary code	Species	Type	Strain	Type	Method	Type	Method	Type
BAVARIE1_0047	Enterovirus	71	BRCR	RD	Absorbance	MTS - 498nm	Microscopy	Tox scoring

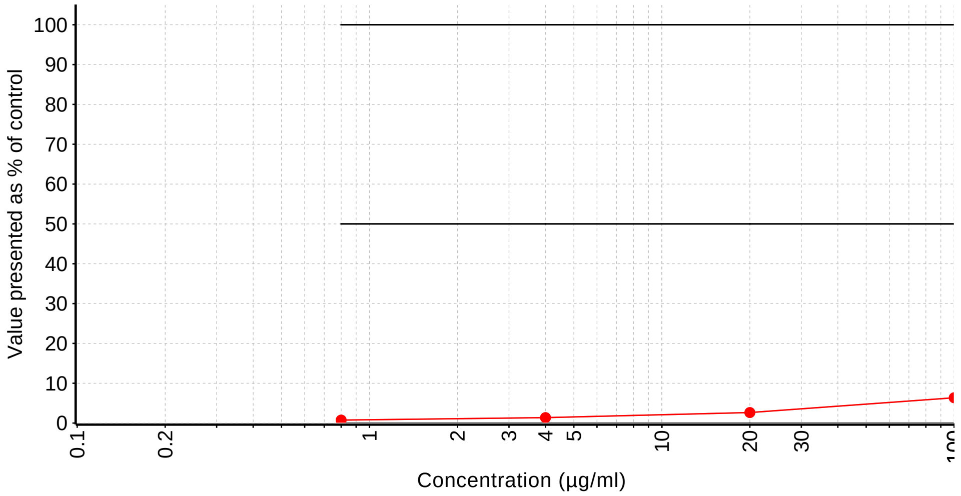

$CC50_{av}$		
EC50	>	100
EC90	>	100
SI_{av}		
SS_{av}		
TI_{av}		
Max %		6.36
[] Max		100
Approved AV		1
Interesting AV		0
Page		47 / 96

Anti-Viral
Anti-Metabolic

Export chart data to CSV

Summary values			
**Statistic**	**CC50**	**EC50**	**EC90**
Median		> 100	> 100
Med.Abs.Dev.			
Mean		> 100	> 100
Stdev.			

Compound	Virus			Cell	AV Method		AM Method	
Primary code	Species	Type	Strain	Type	Method	Type	Method	Type
BAVARIE1_0048	Enterovirus	71	BRCR	RD	Absorbance	MTS - 498nm	Microscopy	Tox scoring

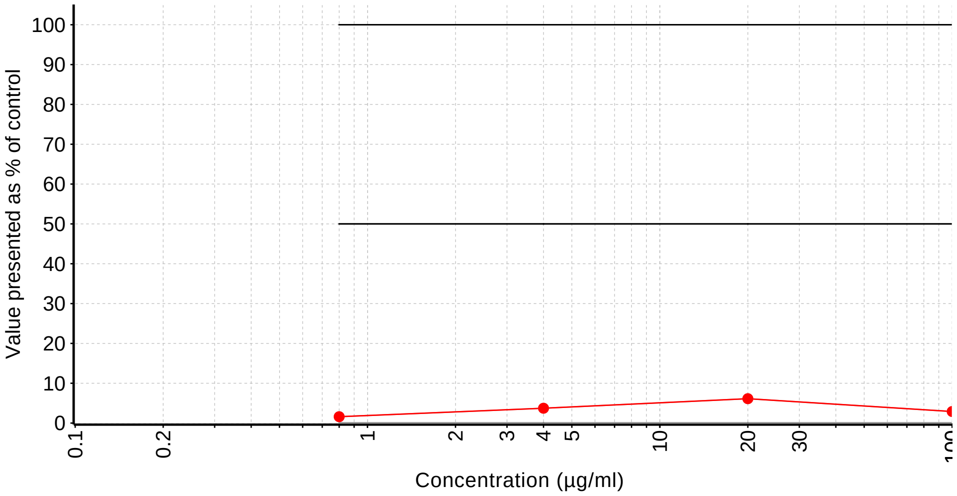

$CC50_{av}$		
EC50	>	100
EC90	>	100
SI_{av}		
SS_{av}		
TI_{av}		
Max %		6.12
[] Max		20
Approved AV		1
Interesting AV		0
Page		48 / 96

Anti-Viral
Anti-Metabolic

Export chart data to CSV

Summary values			
**Statistic**	**CC50**	**EC50**	**EC90**
Median		> 100	> 100
Med.Abs.Dev.			
Mean		> 100	> 100
Stdev.			

**Compound**	**Virus**			**Cell**	**AV Method**		**AM Method**	
Primary code	Species	Type	Strain	Type	Method	Type	Method	Type
BAVARIE1_0049	Enterovirus	71	BRCR	RD	Absorbance	MTS - 498nm	Microscopy	Tox scoring

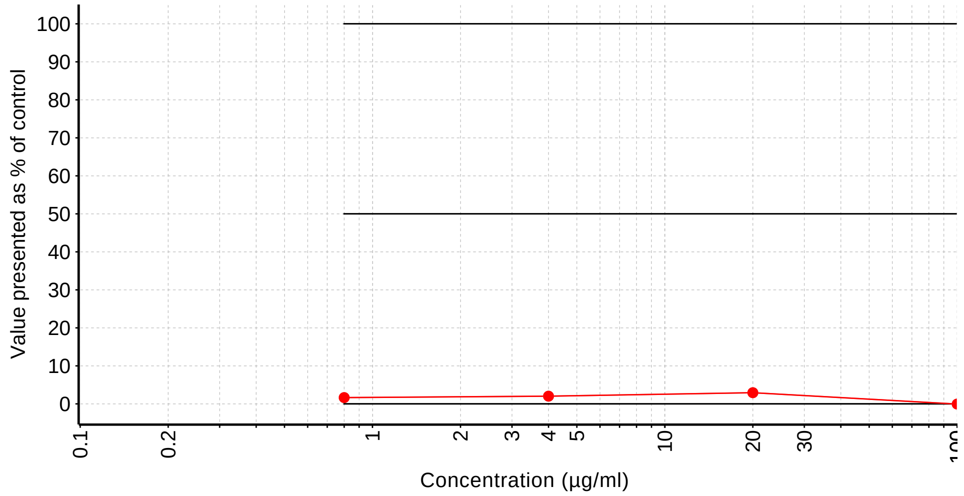

$CC50_{av}$
EC50 > 100
EC90 > 100
SI_{av}
SS_{av}
TI_{av}
Max % 2.94
[] Max 20
Approved AV 1
Interesting AV 0
Page 49 / 96

Anti-Viral
Anti-Metabolic

Export chart data to CSV

**Summary values**			
**Statistic**	**CC50**	**EC50**	**EC90**
Median		> 100	> 100
Med.Abs.Dev.			
Mean		> 100	> 100
Stdev.			

**Compound**	**Virus**			**Cell**	**AV Method**		**AM Method**	
Primary code	Species	Type	Strain	Type	Method	Type	Method	Type
BAVARIE1_0050	Enterovirus	71	BRCR	RD	Absorbance	MTS - 498nm	Microscopy	Tox scoring

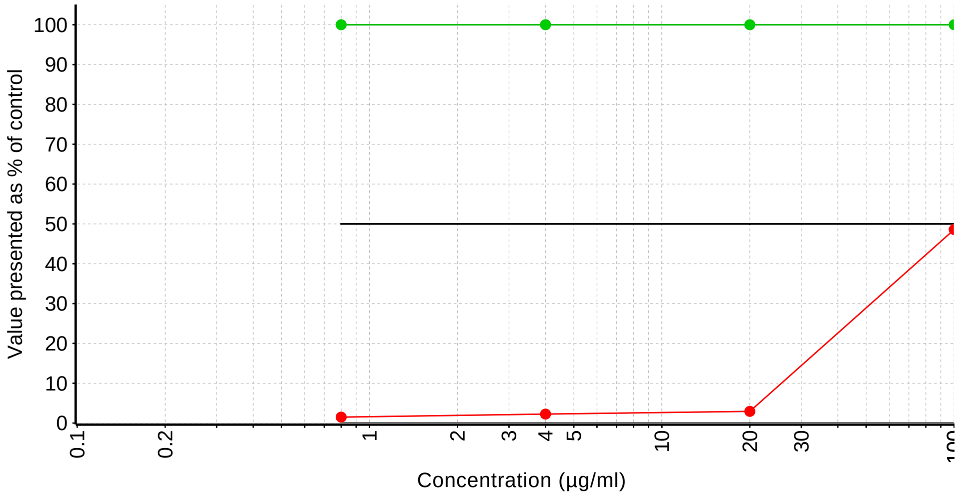

CC50	>	100
EC50	>	100
EC90	>	100
SI		
SS		
TI		
Max %		48.58
[] Max		100
Approved AV		1
Interesting AV		0
Page		50 / 96

Anti-Viral
Anti-Metabolic

Export chart data to CSV

**Summary values**			
**Statistic**	**CC50**	**EC50**	**EC90**
Median		> 100	> 100
Med.Abs.Dev.			
Mean		> 100	> 100
Stdev.			

Compound	Virus			Cell	AV Method		AM Method	
Primary code	Species	Type	Strain	Type	Method	Type	Method	Type
BAVARIE1_0051	Enterovirus	71	BRCR	RD	Absorbance	MTS - 498nm	Microscopy	Tox scoring

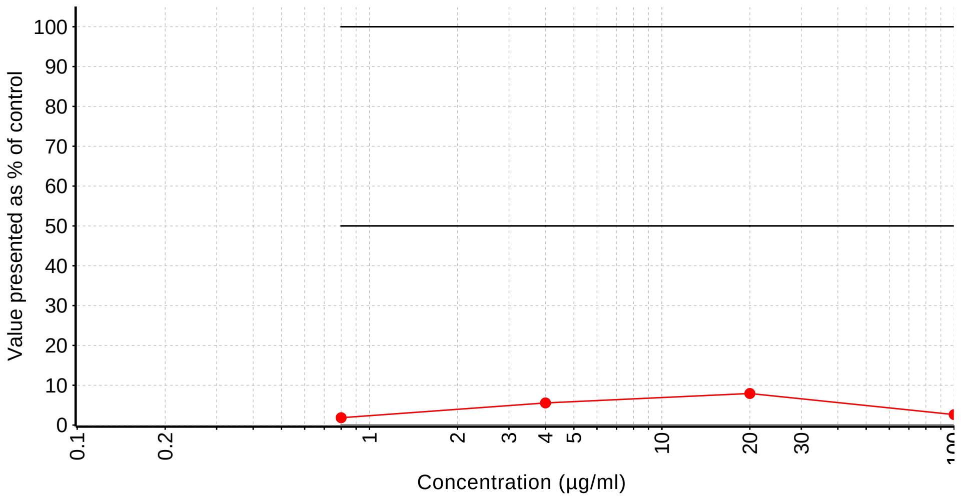

$CC50_{av}$		
EC50	>	100
EC90	>	100
SI_{av}		
SS_{av}		
TI_{av}		
Max %		7.93
[] Max		20
Approved AV		1
Interesting AV		0
Page		51 / 96

Anti-Viral
Anti-Metabolic

Export chart data to CSV

Summary values			
**Statistic**	**CC50**	**EC50**	**EC90**
Median		> 100	> 100
Med.Abs.Dev.			
Mean		> 100	> 100
Stdev.			

Compound	Virus			Cell	AV Method		AM Method	
Primary code	Species	Type	Strain	Type	Method	Type	Method	Type
BAVARIE1_0052	Enterovirus	71	BRCR	RD	Absorbance	MTS - 498nm	Microscopy	Tox scoring

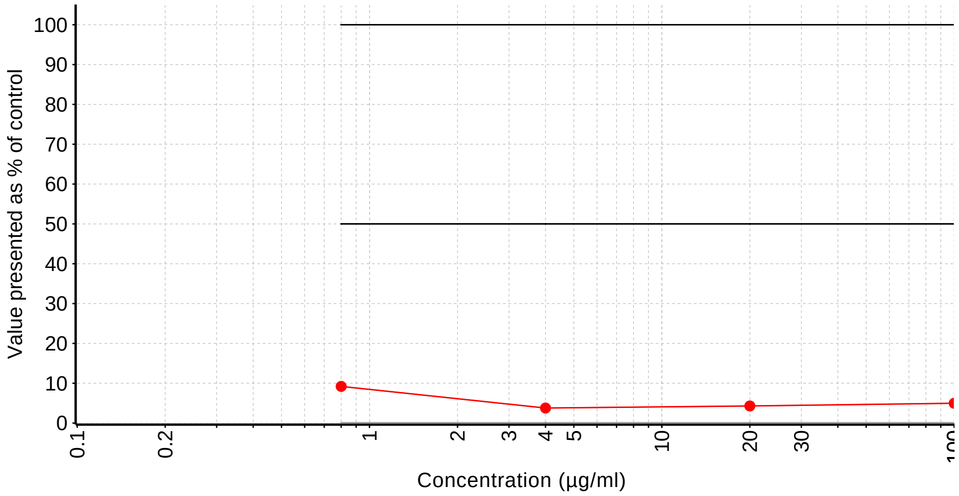

$CC50_{av}$		
EC50	>	100
EC90	>	100
SI_{av}		
SS_{av}		
TI_{av}		
Max %		9.21
[] Max		0.8
Approved AV		1
Interesting AV		0
Page		52 / 96

Anti-Viral
Anti-Metabolic

Export chart data to CSV

Summary values			
**Statistic**	**CC50**	**EC50**	**EC90**
Median		> 100	> 100
Med.Abs.Dev.			
Mean		> 100	> 100
Stdev.			

Compound	Virus			Cell	AV Method		AM Method	
Primary code	Species	Type	Strain	Type	Method	Type	Method	Type
BAVARIE1_0053	Enterovirus	71	BRCR	RD	Absorbance	MTS - 498nm	Microscopy	Tox scoring

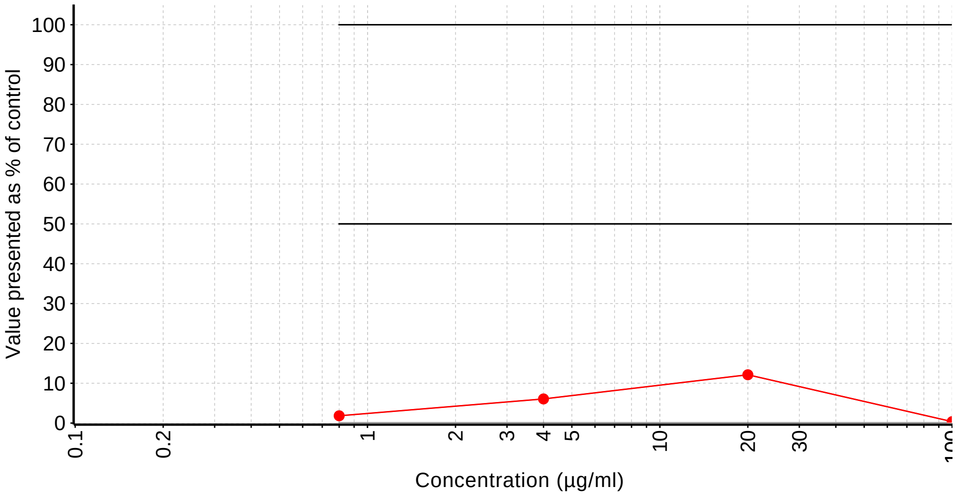

$CC50_{av}$		
EC50	>	100
EC90	>	100
SI_{av}		
SS_{av}		
TI_{av}		
Max %		12.13
[] Max		20
Approved AV		1
Interesting AV		0
Page		53 / 96

Anti-Viral
Anti-Metabolic

Export chart data to CSV

Summary values			
**Statistic**	**CC50**	**EC50**	**EC90**
Median		> 100	> 100
Med.Abs.Dev.			
Mean		> 100	> 100
Stdev.			

Compound	Virus			Cell	AV Method		AM Method	
Primary code	Species	Type	Strain	Type	Method	Type	Method	Type
BAVARIE1_0054	Enterovirus	71	BRCR	RD	Absorbance	MTS - 498nm	Microscopy	Tox scoring

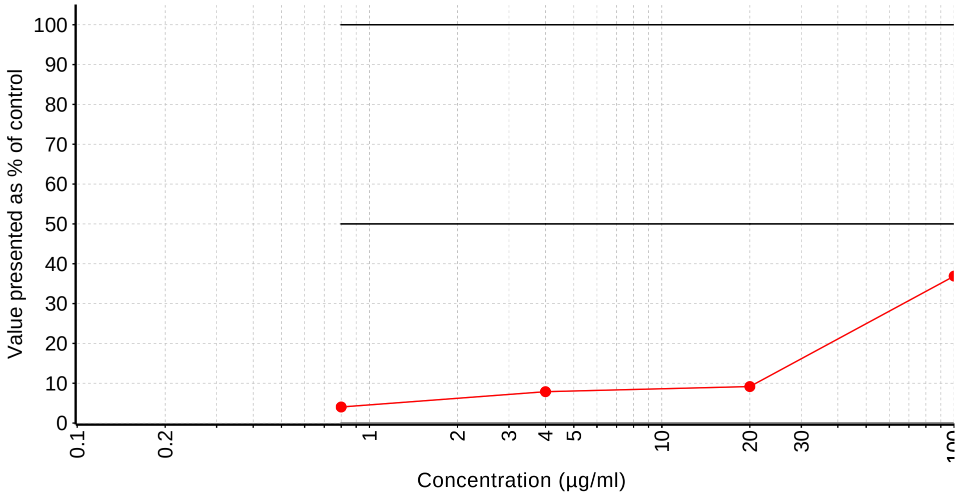

$CC50_{av}$		
EC50	>	100
EC90	>	100
SI_{av}		
SS_{av}		
TI_{av}		
Max %		36.90
[] Max		100
Approved AV		1
Interesting AV		0
Page		54 / 96

Anti-Viral
Anti-Metabolic

Export chart data to CSV

Summary values			
**Statistic**	**CC50**	**EC50**	**EC90**
Median		> 100	> 100
Med.Abs.Dev.			
Mean		> 100	> 100
Stdev.			

Compound	Virus			Cell	AV Method		AM Method	
Primary code	Species	Type	Strain	Type	Method	Type	Method	Type
BAVARIE1_0055	Enterovirus	71	BRCR	RD	Absorbance	MTS - 498nm	Microscopy	Tox scoring

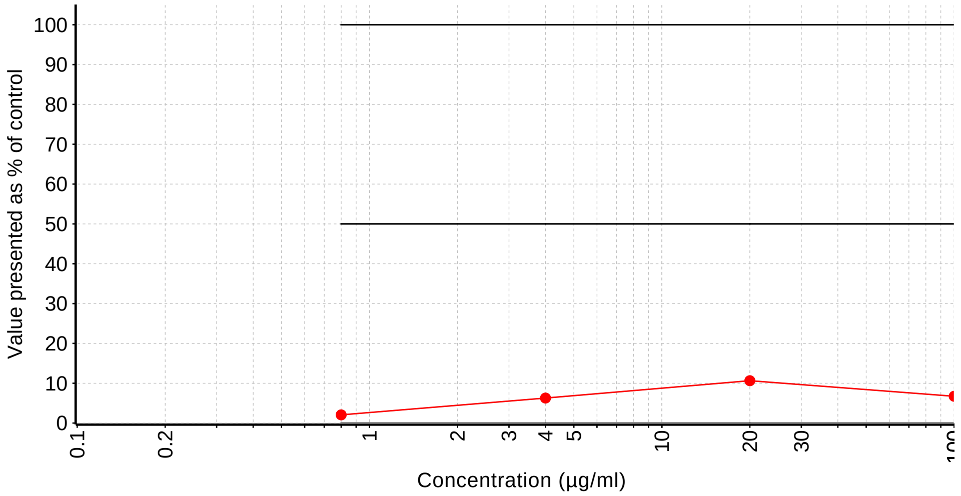

$CC50_{av}$		
EC50	>	100
EC90	>	100
SI_{av}		
SS_{av}		
TI_{av}		
Max %		10.64
[] Max		20
Approved AV		1
Interesting AV		0
Page		55 / 96

Anti-Viral
Anti-Metabolic

Export chart data to CSV

Summary values			
**Statistic**	**CC50**	**EC50**	**EC90**
Median		> 100	> 100
Med.Abs.Dev.			
Mean		> 100	> 100
Stdev.			

Compound	Virus			Cell	AV Method		AM Method	
Primary code	Species	Type	Strain	Type	Method	Type	Method	Type
BAVARIE1_0056	Enterovirus	71	BRCR	RD	Absorbance	MTS - 498nm	Microscopy	Tox scoring

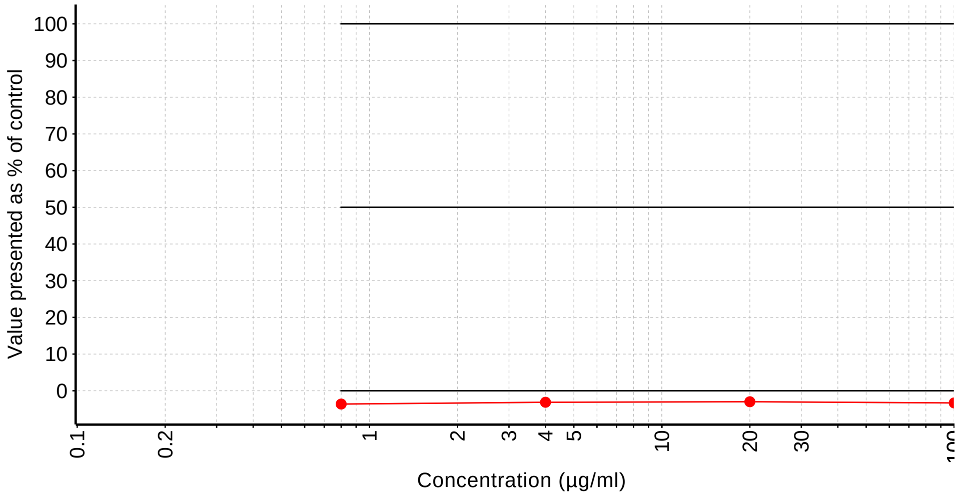

$CC50_{av}$		
EC50	>	100
EC90	>	100
SI_{av}		
SS_{av}		
TI_{av}		
Max %		-2.99
[] Max		20
Approved AV		1
Interesting AV		0
Page		56 / 96

Anti-Viral
Anti-Metabolic

Export chart data to CSV

Summary values			
**Statistic**	**CC50**	**EC50**	**EC90**
Median		> 100	> 100
Med.Abs.Dev.			
Mean		> 100	> 100
Stdev.			

Compound	Virus			Cell	AV Method		AM Method	
Primary code	Species	Type	Strain	Type	Method	Type	Method	Type
BAVARIE1_0057	Enterovirus	71	BRCR	RD	Absorbance	MTS - 498nm	Microscopy	Tox scoring

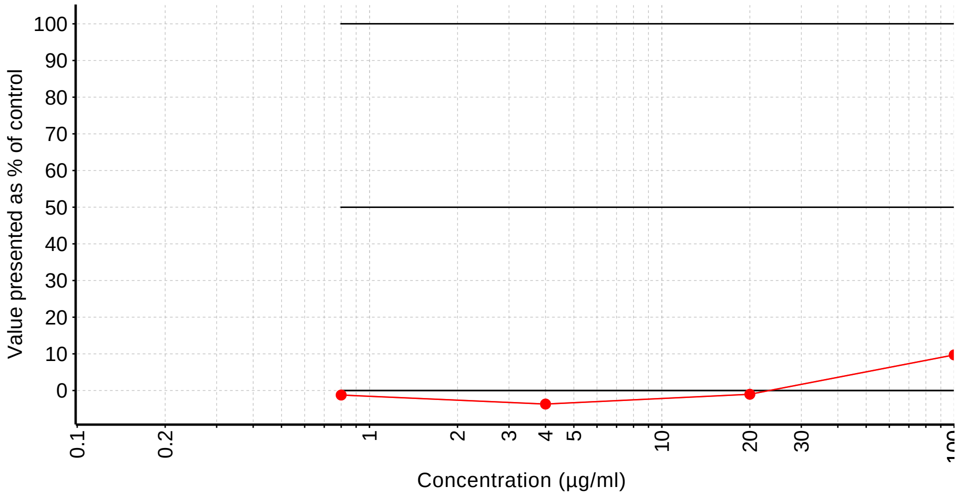

$CC50_{av}$		
EC50	>	100
EC90	>	100
SI_{av}		
SS_{av}		
TI_{av}		
Max %		9.71
[] Max		100
Approved AV		1
Interesting AV		0
Page		57 / 96

Anti-Viral
Anti-Metabolic

Export chart data to CSV

Summary values			
**Statistic**	**CC50**	**EC50**	**EC90**
Median		> 100	> 100
Med.Abs.Dev.			
Mean		> 100	> 100
Stdev.			

Compound	Virus			Cell	AV Method		AM Method	
Primary code	Species	Type	Strain	Type	Method	Type	Method	Type
BAVARIE1_0058	Enterovirus	71	BRCR	RD	Absorbance	MTS - 498nm	Microscopy	Tox scoring

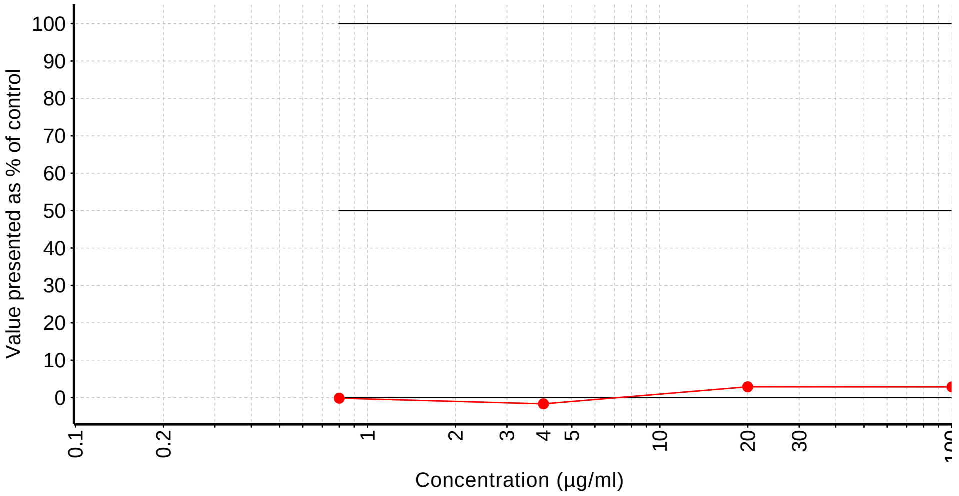

$CC50_{av}$		
EC50	>	100
EC90	>	100
SI_{av}		
SS_{av}		
TI_{av}		
Max %		2.89
[] Max		20
Approved AV		1
Interesting AV		0
Page		58 / 96

Anti-Viral
Anti-Metabolic

Export chart data to CSV

Summary values			
**Statistic**	**CC50**	**EC50**	**EC90**
Median		> 100	> 100
Med.Abs.Dev.			
Mean		> 100	> 100
Stdev.			

**Compound**	**Virus**			**Cell**	**AV Method**		**AM Method**	
Primary code	Species	Type	Strain	Type	Method	Type	Method	Type
BAVARIE1_0059	Enterovirus	71	BRCR	RD	Absorbance	MTS - 498nm	Microscopy	Tox scoring

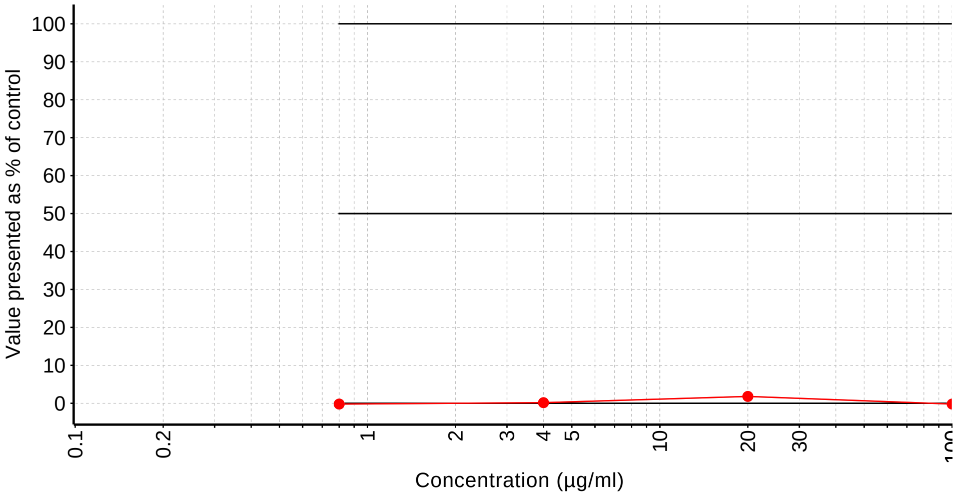

$CC50_{av}$		
EC50	>	100
EC90	>	100
SI_{av}		
SS_{av}		
TI_{av}		
Max %		1.81
[] Max		20
Approved AV		1
Interesting AV		0
Page		59 / 96

Anti-Viral
Anti-Metabolic

Export chart data to CSV

**Summary values**			
**Statistic**	**CC50**	**EC50**	**EC90**
Median		> 100	> 100
Med.Abs.Dev.			
Mean		> 100	> 100
Stdev.			

**Compound**	**Virus**			**Cell**	**AV Method**		**AM Method**	
Primary code	Species	Type	Strain	Type	Method	Type	Method	Type
BAVARIE1_0060	Enterovirus	71	BRCR	RD	Absorbance	MTS - 498nm	Microscopy	Tox scoring

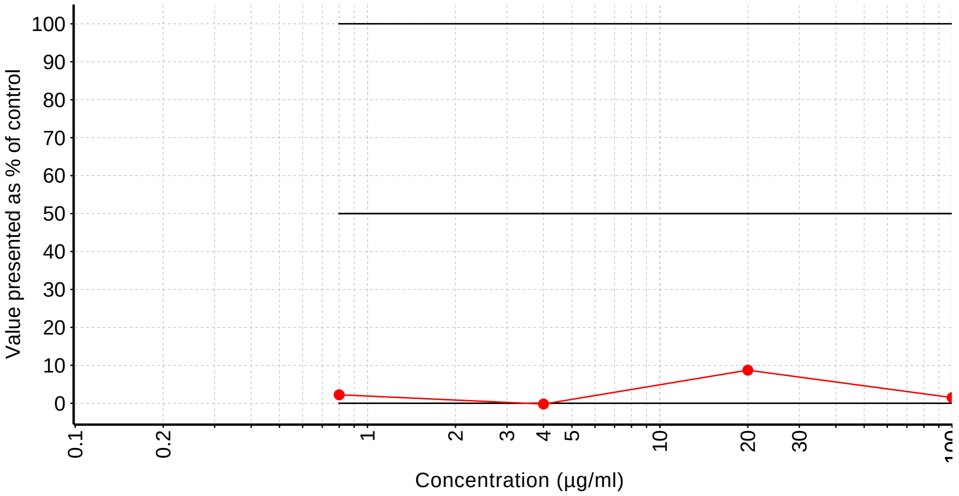

$CC50_{av}$		
EC50	>	100
EC90	>	100
SI_{av}		
SS_{av}		
TI_{av}		
Max %		8.73
[] Max		20
Approved AV		1
Interesting AV		0
Page		60 / 96

Anti-Viral
Anti-Metabolic

Export chart data to CSV

**Summary values**			
**Statistic**	**CC50**	**EC50**	**EC90**
Median		> 100	> 100
Med.Abs.Dev.			
Mean		> 100	> 100
Stdev.			
