# Supplementary material for: Antiviral and Cytotoxic Activity of Different Plant Parts of Banana (Musa spp.)
Source: Viruses. 2020 May 15;12(5):549. doi: 10.3390/v12050549 (PMC7291111; doi:10.3390/v12050549)
Supplement: Supplementary file 1 [file viruses-12-00549-s001.zip › Supplementary Files~/Supplementary material II-Chikungunya-SP.pdf]

Antiviral and Cytotoxic Activity of Different Plant Parts of Banana (*Musa* spp.)

Sujogya Kumar Panda [1,2,*], Ana Hortência Fonsêca Castro [1,3], Ramin Saleh Jouneghani [1], Pieter Leyssen [4], Johan Neyts [4], Rony Swennen [5,6,7] and Walter Luyten [1]

1 Department of Biology, Katholieke Universiteit Leuven, 3000 Leuven, Belgium; acastro905@gmail.com (A.H.F.C.); r.saleh.j@gmail.com (R.S.J.); walter.luyten@kuleuven.be (W.L.)
2 Mayurbhanj Biological Research (MBR), Bhanjpur, Baripada 757002, Odisha, India
3 Plant Physiology and Biochemistry, Universidade Federal de São João Del-Rei, Av. Sebastião Gonçalves Coelho, 400—Chanandour, Divinópolis MG 35501-296, Brazil
4 Rega Institute for Medical Research, Laboratory of Virology and Chemotherapy, Katholieke Universiteit Leuven, 3000 Leuven, Belgium; pieter.leyssen@kuleuven.be (P.L.); johan.neyts@kuleuven.be (J.N.)
5 International Institute of Tropical Agriculture, Arusha P.O. Box 447, Tanzania; rony.swennen@kuleuven.be
6 Laboratory of Tropical Crop Improvement, Division of Crop Biotechnics, Katholieke Universiteit Leuven, 3001 Leuven, Belgium
7 Bioversity International, 3001 Leuven, Belgium
* Correspondence: sujogya.panda@kuleuven.be; Tel.: +32-16-373467

Supplementary material II

Figure S2: Antiviral activity of extracts against Chikungunya virus. Extracts (primary code) tested mentioned under the header compounds in each panel. EC_{50} = 50% Effective Concentration (concentration at which 50% inhibition of virus replication is observed); EC_{90} = 90% Effective Concentration (concentration at which 90% inhibition of virus replication is observed); CC_{50} = 50% Cytostatic/Cytotoxic Concentration (concentration at which 50% adverse effect is observed on Vero cells in parallel with antiviral assay); SI = Selectivity Index (CC_{50}/EC_{50}); SS = Selectivity Surface (integrated surface delineated by the EC_{50} curve, the CC_{50} curve and the 50% horizontal); TI = Therapeutic Index (SS × 10logSI); AV-Antiviral; AM-Antimetabolic.

Compound	Virus			Cell		AV Method		AM Method	
Primary code	Species	Type	Strain	Type	Subtype	Method	Type	Method	Type
BAVARIE1_0001	Chikungunya virus	No Type	899	Vero	A	Absorbance	MTS - 498nm	Microscopy	Tox scoring

Needs more data.

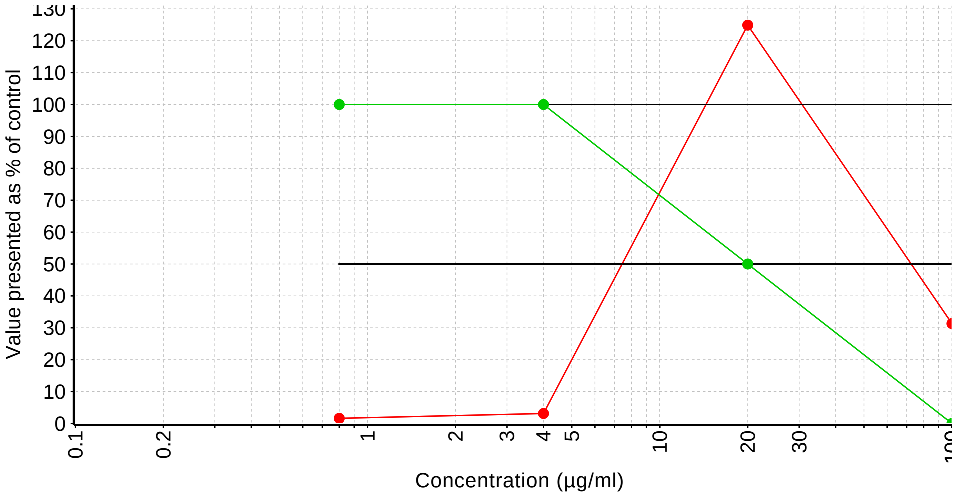

CC50	=	20
EC50	=	7.43
EC90		
SI	=	2.69
SS	=	4.69
TI	=	2.01
Max %		71.80
[] Max		13
Approved AV		1
Interesting AV		0
Page		1 / 96

Anti-Viral
Anti-Metabolic

Export chart data to CSV

Summary values			
**Statistic**	**CC50**	**EC50**	**EC90**
Median	= 46.3	= 7.43	= 12.6
Med.Abs.Dev.	26.3		
Mean	= 46.3	= 7.43	= 12.6
Stdev.	37.1		

**Compound**	**Virus**			**Cell**		**AV Method**		**AM Method**	
Primary code	Species	Type	Strain	Type	Subtype	Method	Type	Method	Type
BAVARIE1_0002	Chikungunya virus	No Type	899	Vero	A	Absorbance	MTS - 498nm	Microscopy	Tox scoring

Needs more data.

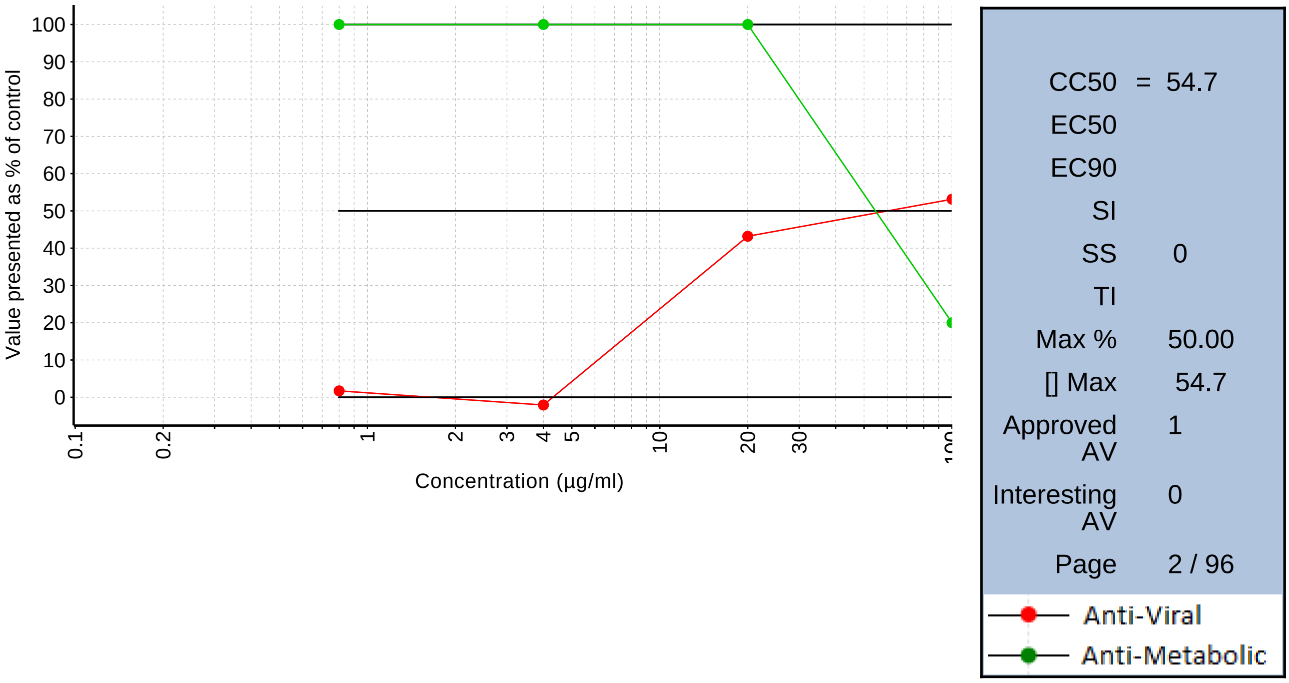

Export chart data to CSV

Summary values			
**Statistic**	**CC50**	**EC50**	**EC90**
Median	= 54.7	= 60.2	> 100
Med.Abs.Dev.			
Mean	= 54.7	= 60.2	> 100
Stdev.			

**Compound**	**Virus**			**Cell**		**AV Method**		**AM Method**	
Primary code	Species	Type	Strain	Type	Subtype	Method	Type	Method	Type
BAVARIE1_0003	Chikungunya virus	No Type	899	Vero	A	Absorbance	MTS - 498nm	Microscopy	Tox scoring

Needs more data.

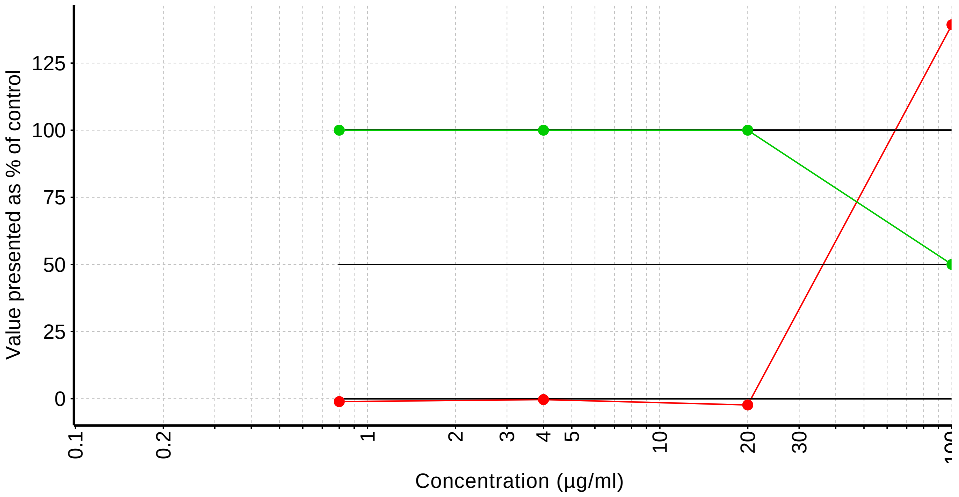

CC50 = 100
EC50 = 36.3
EC90
SI = 2.76
SS = 5.13
TI = 2.26
Max % 73.30
[] Max 62.7
Approved AV 1
Interesting AV 0
Page 3 / 96

Anti-Viral
Anti-Metabolic

[Export chart data to CSV]

Summary values			
**Statistic**	**CC50**	**EC50**	**EC90**
Median	= 100	= 36.3	= 57.1
Med.Abs.Dev.			
Mean	= 100	= 36.3	= 57.1
Stdev.			

Compound	Virus			Cell		AV Method		AM Method	
Primary code	Species	Type	Strain	Type	Subtype	Method	Type	Method	Type
BAVARIE1_0004	Chikungunya virus	No Type	899	Vero	A	Absorbance	MTS - 498nm	Microscopy	Tox scoring

Needs more data.

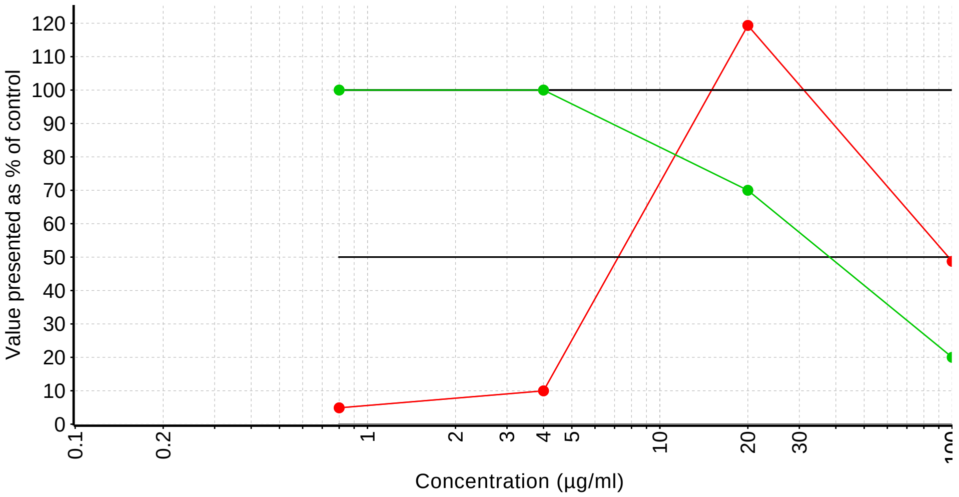

CC50	= 38.1
EC50	= 7.21
EC90	
SI	= 5.28
SS	= 11
TI	= 7.97
Max %	80.62
[] Max	14.3
Approved AV	1
Interesting AV	0
Page	4 / 96

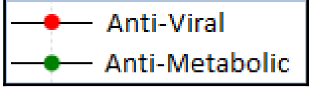

Export chart data to CSV

Summary values			
**Statistic**	**CC50**	**EC50**	**EC90**
Median	= 67.6	= 7.21	= 13
Med.Abs.Dev.	29.5		
Mean	= 67.6	= 7.21	= 13
Stdev.	41.8		

Compound	Virus			Cell		AV Method		AM Method	
Primary code	Species	Type	Strain	Type	Subtype	Method	Type	Method	Type
BAVARIE1_0005	Chikungunya virus	No Type	899	Vero	A	Absorbance	MTS - 498nm	Microscopy	Tox scoring

Needs more data.

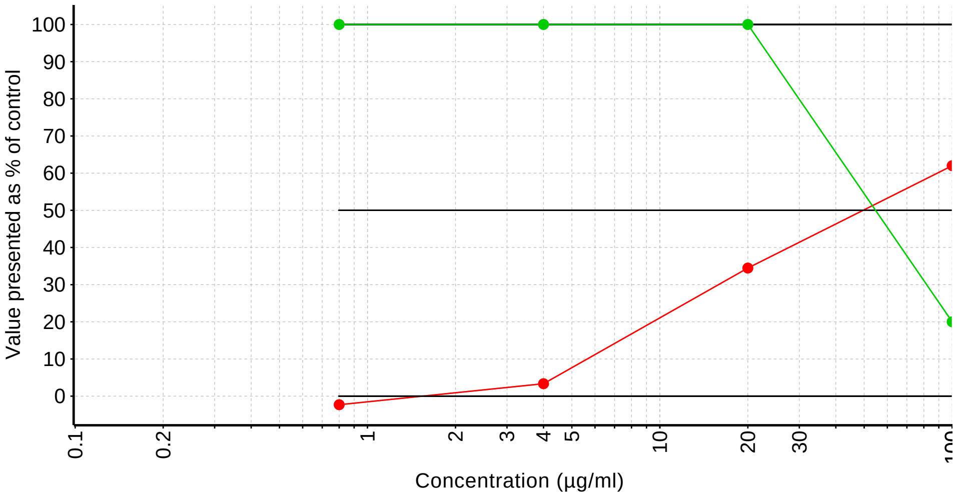

CC50 = 54.7
EC50 = 49.6
EC90
SI = 1.1
SS = 0.062
TI = 0.00264
Max % 51.25
[] Max 68.8
Approved AV 1
Interesting AV 0
Page 5 / 96

Anti-Viral
Anti-Metabolic

Export chart data to CSV

Summary values			
**Statistic**	**CC50**	**EC50**	**EC90**
Median	= 54.7	= 49.6	> 100
Med.Abs.Dev.			
Mean	= 54.7	= 49.6	> 100
Stdev.			

**Compound**	**Virus**			**Cell**		**AV Method**		**AM Method**	
Primary code	Species	Type	Strain	Type	Subtype	Method	Type	Method	Type
BAVARIE1_0006	Chikungunya virus	No Type	899	Vero	A	Absorbance	MTS - 498nm	Microscopy	Tox scoring

Needs more data.

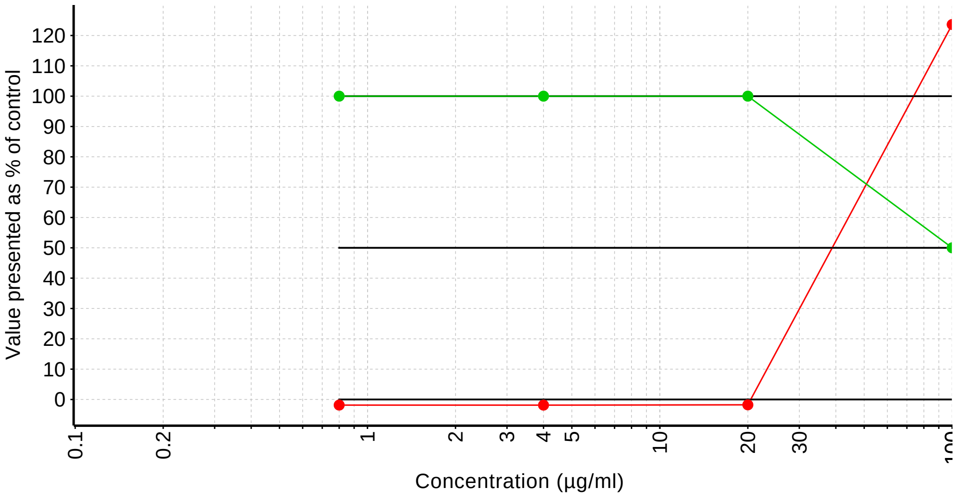

CC50	= 100
EC50	= 38.9
EC90	
SI	= 2.57
SS	= 4.31
TI	= 1.77
Max %	70.99
[] Max	66.4
Approved AV	1
Interesting AV	0
Page	6 / 96

Anti-Viral
Anti-Metabolic

Export chart data to CSV

**Summary values**			
**Statistic**	**CC50**	**EC50**	**EC90**
Median	= 100	= 38.9	= 65
Med.Abs.Dev.			
Mean	= 100	= 38.9	= 65
Stdev.			

Compound	Virus			Cell		AV Method		AM Method	
Primary code	Species	Type	Strain	Type	Subtype	Method	Type	Method	Type
BAVARIE1_0007	Chikungunya virus	No Type	899	Vero	A	Absorbance	MTS - 498nm	Microscopy	Tox scoring

Needs more data.

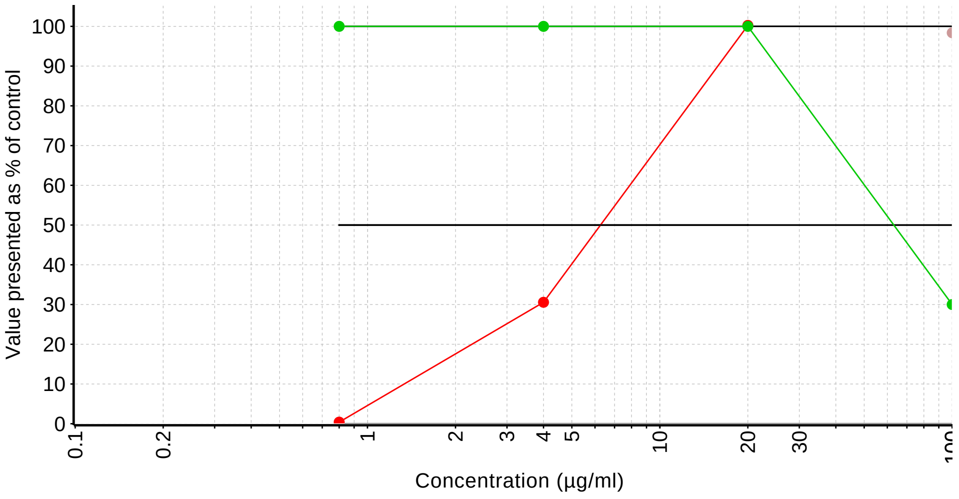

CC50	=	63.1
EC50	=	6.27
EC90	=	15.8
SI	=	10.1
SS	=	25.3
TI	=	25.4
Max %		100.00
[] Max		19.9
Approved AV		1
Interesting AV		0
Page		7 / 96

Anti-Viral
Anti-Metabolic

Export chart data to CSV

Summary values			
**Statistic**	**CC50**	**EC50**	**EC90**
Median	= 63.1	= 6.27	= 15.8
Med.Abs.Dev.			
Mean	= 63.1	= 6.27	= 15.8
Stdev.			

**Compound**	**Virus**			**Cell**		**AV Method**		**AM Method**	
Primary code	Species	Type	Strain	Type	Subtype	Method	Type	Method	Type
BAVARIE1_0008	Chikungunya virus	No Type	899	Vero	A	Absorbance	MTS - 498nm	Microscopy	Tox scoring

Needs more data.

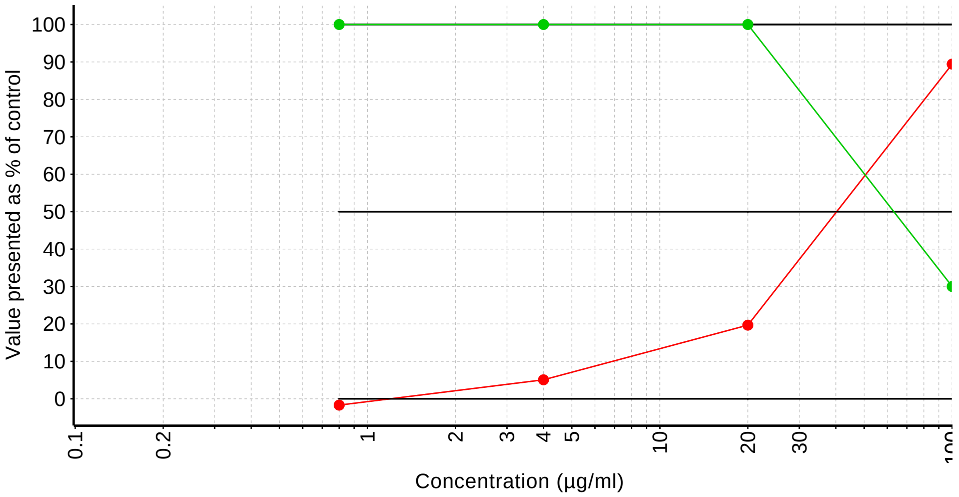

CC50	=	63.1
EC50	=	40.3
EC90		
SI	=	1.57
SS	=	0.0934
TI	=	0.0183
Max %		59.77
[] Max		66
Approved AV		1
Interesting AV		0
Page		8 / 96

Anti-Viral
Anti-Metabolic

Export chart data to CSV

**Summary values**			
**Statistic**	**CC50**	**EC50**	**EC90**
Median	= 63.1	= 40.3	> 100
Med.Abs.Dev.			
Mean	= 63.1	= 40.3	> 100
Stdev.			

**Compound**	**Virus**			**Cell**		**AV Method**		**AM Method**	
Primary code	Species	Type	Strain	Type	Subtype	Method	Type	Method	Type
BAVARIE1_0009	Chikungunya virus	No Type	899	Vero	A	Absorbance	MTS - 498nm	Microscopy	Tox scoring

Needs more data.

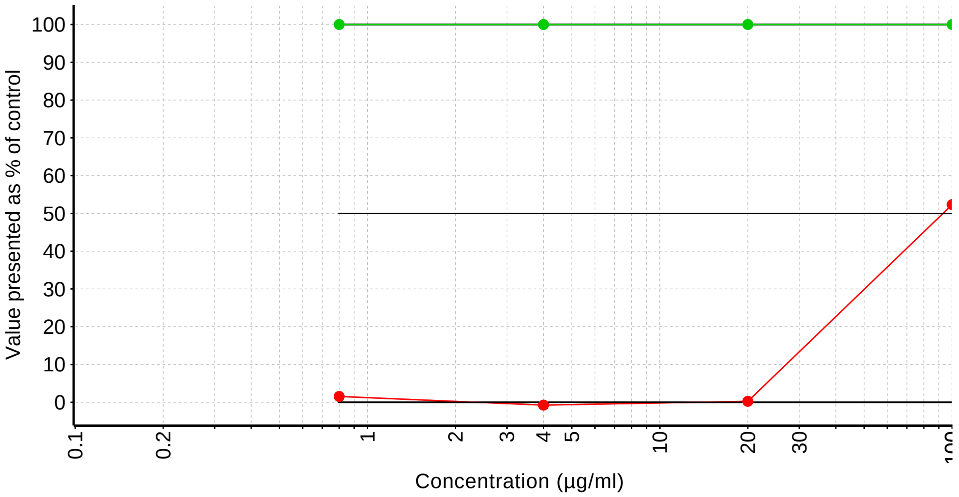

CC50	>	100
EC50	=	93
EC90	>	100
SI	>	1.07
SS	>	0.0365
TI	>	0.00114
Max %		52.33
[] Max		100
Approved AV		1
Interesting AV		0
Page		9 / 96

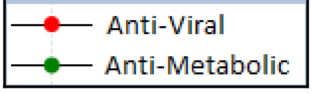

Export chart data to CSV

**Summary values**			
**Statistic**	**CC50**	**EC50**	**EC90**
Median	> 100	= 93	> 100
Med.Abs.Dev.			
Mean	> 100	= 93	> 100
Stdev.			

**Compound**	**Virus**			**Cell**		**AV Method**		**AM Method**	
Primary code	Species	Type	Strain	Type	Subtype	Method	Type	Method	Type
BAVARIE1_0010	Chikungunya virus	No Type	899	Vero	A	Absorbance	MTS - 498nm	Microscopy	Tox scoring

Needs more data.

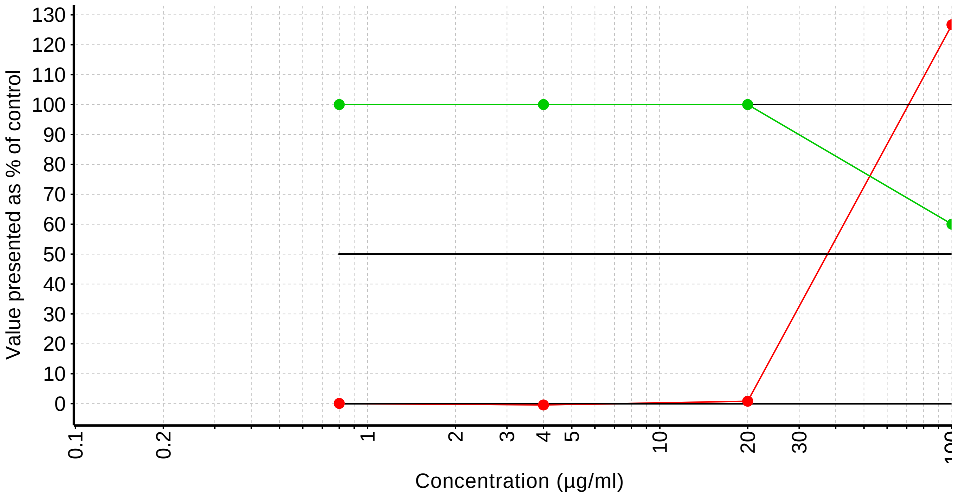

CC50	>	100
EC50	=	37.5
EC90		
SI	>	2.67
SS	>	6.4
TI	>	2.72
Max %		76.08
[] Max		67.8
Approved AV		1
Interesting AV		0
Page		10 / 96

Anti-Viral
Anti-Metabolic

Export chart data to CSV

**Summary values**			
**Statistic**	**CC50**	**EC50**	**EC90**
Median	> 100	= 37.5	= 62.6
Med.Abs.Dev.			
Mean	> 100	= 37.5	= 62.6
Stdev.			

Compound	Virus			Cell		AV Method		AM Method	
Primary code	Species	Type	Strain	Type	Subtype	Method	Type	Method	Type
BAVARIE1_0011	Chikungunya virus	No Type	899	Vero	A	Absorbance	MTS - 498nm	Microscopy	Tox scoring

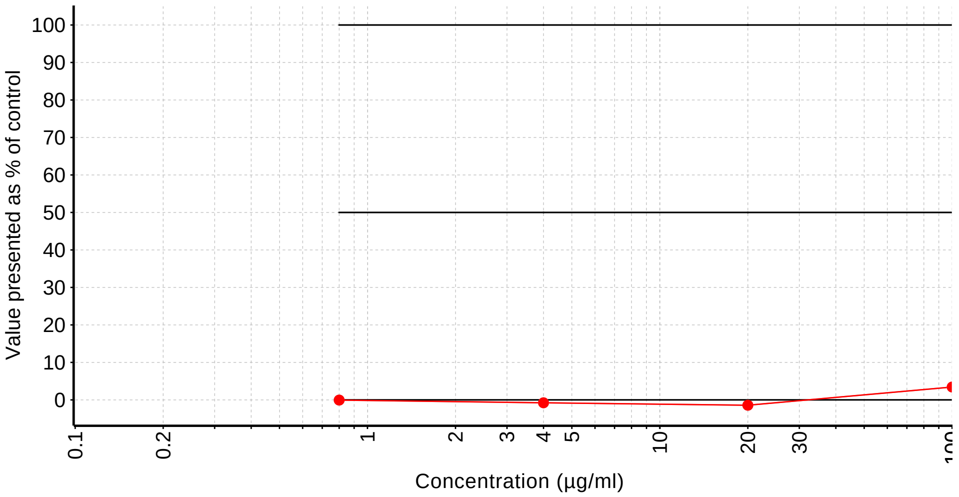

$CC50_{av}$		
EC50	>	100
EC90	>	100
SI_{av}		
SS_{av}		
TI_{av}		
Max %		3.43
[] Max		100
Approved AV		1
Interesting AV		0
Page		11 / 96

Anti-Viral
Anti-Metabolic

Export chart data to CSV

Summary values			
**Statistic**	**CC50**	**EC50**	**EC90**
Median		> 100	> 100
Med.Abs.Dev.			
Mean		> 100	> 100
Stdev.			

Compound	Virus			Cell		AV Method		AM Method	
Primary code	Species	Type	Strain	Type	Subtype	Method	Type	Method	Type
BAVARIE1_0012	Chikungunya virus	No Type	899	Vero	A	Absorbance	MTS - 498nm	Microscopy	Tox scoring

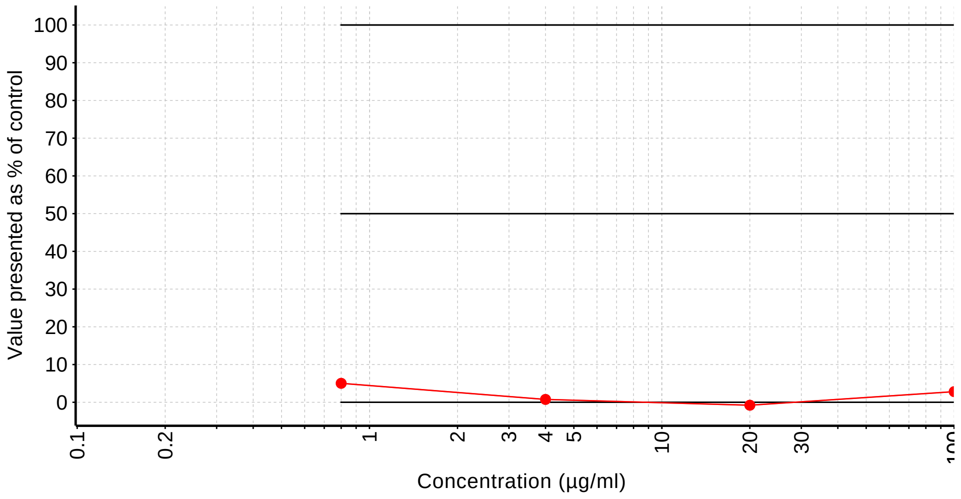

$CC50_{av}$		
EC50	>	100
EC90	>	100
SI_{av}		
SS_{av}		
TI_{av}		
Max %		5.02
[] Max		0.8
Approved AV		1
Interesting AV		0
Page		12 / 96

Anti-Viral
Anti-Metabolic

Export chart data to CSV

Summary values			
**Statistic**	**CC50**	**EC50**	**EC90**
Median		> 100	> 100
Med.Abs.Dev.			
Mean		> 100	> 100
Stdev.			

**Compound**	**Virus**			**Cell**		**AV Method**		**AM Method**	
Primary code	Species	Type	Strain	Type	Subtype	Method	Type	Method	Type
BAVARIE1_0013	Chikungunya virus	No Type	899	Vero	A	Absorbance	MTS - 498nm	Microscopy	Tox scoring

Needs more data.

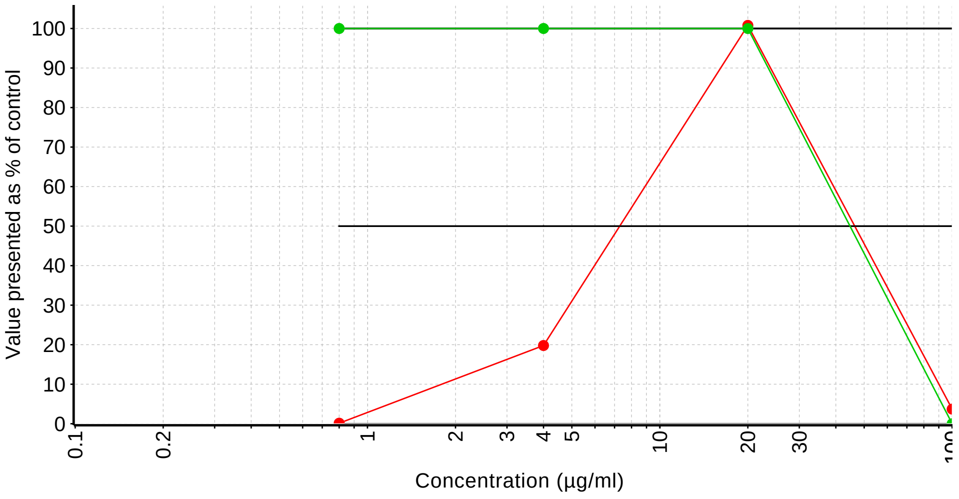

CC50	=	44.7
EC50	=	7.29
EC90	=	16.1
SI	=	6.13
SS	=	19.8
TI	=	15.6
Max %		100.00
[] Max		19.8
Approved AV		1
Interesting AV		1
Page		13 / 96

Anti-Viral
Anti-Metabolic

Export chart data to CSV

**Summary values**			
**Statistic**	**CC50**	**EC50**	**EC90**
Median	= 45.6	= 7.29	= 16.1
Med.Abs.Dev.	0.847		
Mean	= 45.6	= 7.29	= 16.1
Stdev.	1.2		

**Compound**	**Virus**			**Cell**		**AV Method**		**AM Method**	
Primary code	Species	Type	Strain	Type	Subtype	Method	Type	Method	Type
BAVARIE1_0014	Chikungunya virus	No Type	899	Vero	A	Absorbance	MTS - 498nm	Microscopy	Tox scoring

Needs more data.

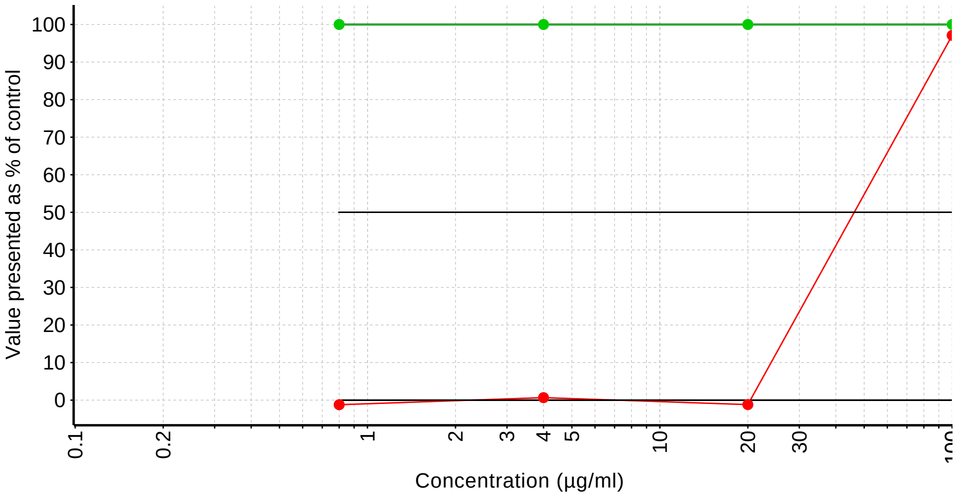

CC50	>	100
EC50	=	46.2
EC90	=	89
SI	>	2.16
SS	>	7.89
TI	>	2.64
Max %		97.10
[] Max		100
Approved AV		1
Interesting AV		0
Page		14 / 96

Anti-Viral
Anti-Metabolic

Export chart data to CSV

**Summary values**			
**Statistic**	**CC50**	**EC50**	**EC90**
Median	> 100	= 46.2	= 89
Med.Abs.Dev.			
Mean	> 100	= 46.2	= 89
Stdev.			

Compound	Virus			Cell		AV Method		AM Method	
Primary code	Species	Type	Strain	Type	Subtype	Method	Type	Method	Type
BAVARIE1_0015	Chikungunya virus	No Type	899	Vero	A	Absorbance	MTS - 498nm	Microscopy	Tox scoring

Needs more data.

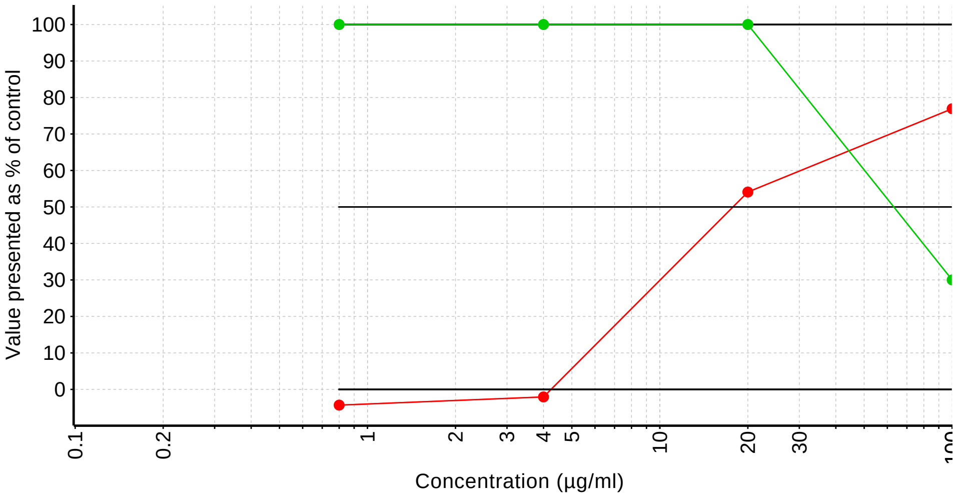

CC50	= 63.1
EC50	= 17.8
EC90	
SI	= 3.55
SS	= 4.91
TI	= 2.7
Max %	65.38
[] Max	59.6
Approved AV	1
Interesting AV	0
Page	15 / 96

Anti-Viral
Anti-Metabolic

Export chart data to CSV

Summary values			
**Statistic**	**CC50**	**EC50**	**EC90**
Median	= 63.1	= 17.8	> 100
Med.Abs.Dev.			
Mean	= 63.1	= 17.8	> 100
Stdev.			

Compound	Virus			Cell		AV Method		AM Method	
Primary code	Species	Type	Strain	Type	Subtype	Method	Type	Method	Type
BAVARIE1_0016	Chikungunya virus	No Type	899	Vero	A	Absorbance	MTS - 498nm	Microscopy	Tox scoring

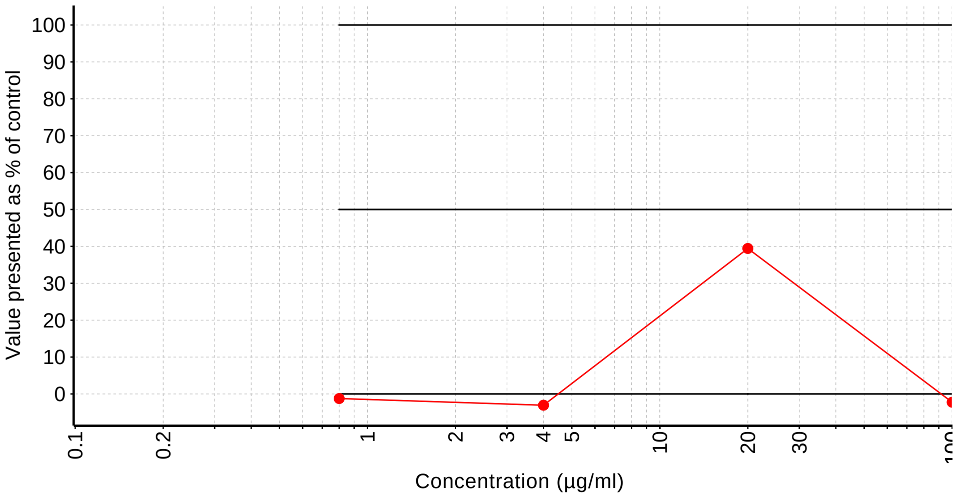

$CC50_{av}$	
EC50	
EC90	
SI_{av}	
SS_{av}	
TI_{av}	
Max %	39.43
[] Max	20
Approved AV	1
Interesting AV	0
Page	16 / 96

Anti-Viral
Anti-Metabolic

Export chart data to CSV

Summary values			
**Statistic**	**CC50**	**EC50**	**EC90**
Median			
Med.Abs.Dev.			
Mean			
Stdev.			

Compound	Virus			Cell		AV Method		AM Method	
Primary code	Species	Type	Strain	Type	Subtype	Method	Type	Method	Type
BAVARIE1_0017	Chikungunya virus	No Type	899	Vero	A	Absorbance	MTS - 498nm	Microscopy	Tox scoring

Needs more data.

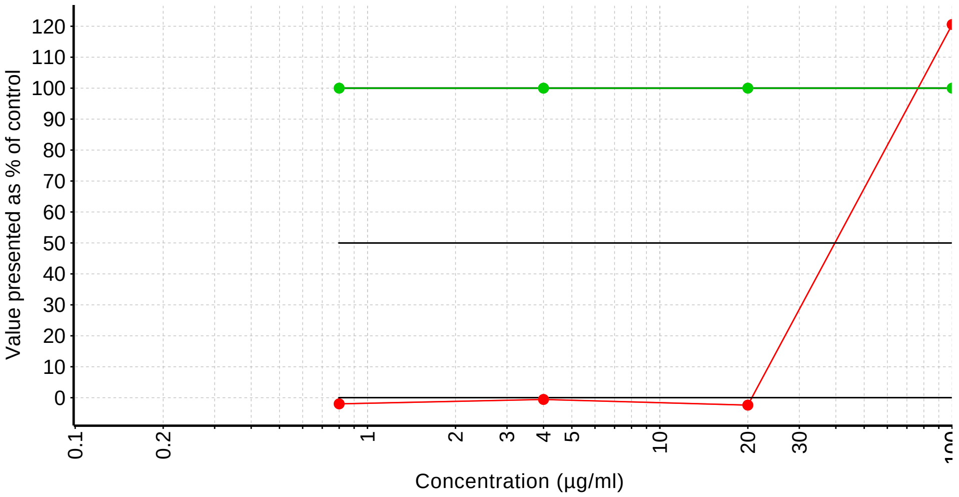

CC50	>	100
EC50	=	39.7
EC90	=	67
SI	>	2.52
SS	>	11.6
TI	>	4.65
Max %		100.00
[] Max		86.6
Approved AV		1
Interesting AV		0
Page		17 / 96

Anti-Viral
Anti-Metabolic

[Export chart data to CSV]

Summary values			
**Statistic**	**CC50**	**EC50**	**EC90**
Median	> 100	= 39.7	= 67
Med.Abs.Dev.			
Mean	> 100	= 39.7	= 67
Stdev.			

Compound	Virus			Cell		AV Method		AM Method	
Primary code	Species	Type	Strain	Type	Subtype	Method	Type	Method	Type
BAVARIE1_0018	Chikungunya virus	No Type	899	Vero	A	Absorbance	MTS - 498nm	Microscopy	Tox scoring

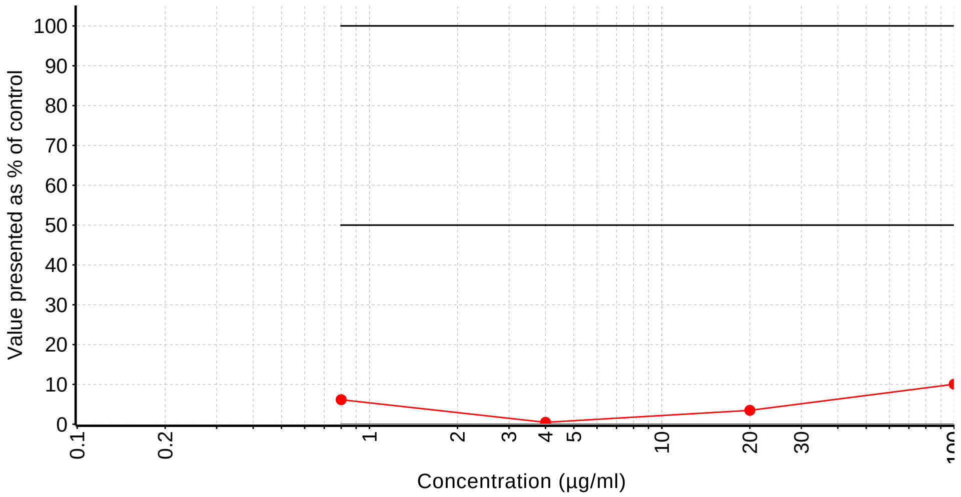

$CC50_{av}$		
EC50	>	100
EC90	>	100
SI_{av}		
SS_{av}		
TI_{av}		
Max %		10.04
[] Max		100
Approved AV		1
Interesting AV		0
Page		18 / 96

Anti-Viral
Anti-Metabolic

Export chart data to CSV

Summary values			
**Statistic**	**CC50**	**EC50**	**EC90**
Median		> 100	> 100
Med.Abs.Dev.			
Mean		> 100	> 100
Stdev.			

Compound	Virus			Cell		AV Method		AM Method	
Primary code	Species	Type	Strain	Type	Subtype	Method	Type	Method	Type
BAVARIE1_0019	Chikungunya virus	No Type	899	Vero	A	Absorbance	MTS - 498nm	Microscopy	Tox scoring

Needs more data.

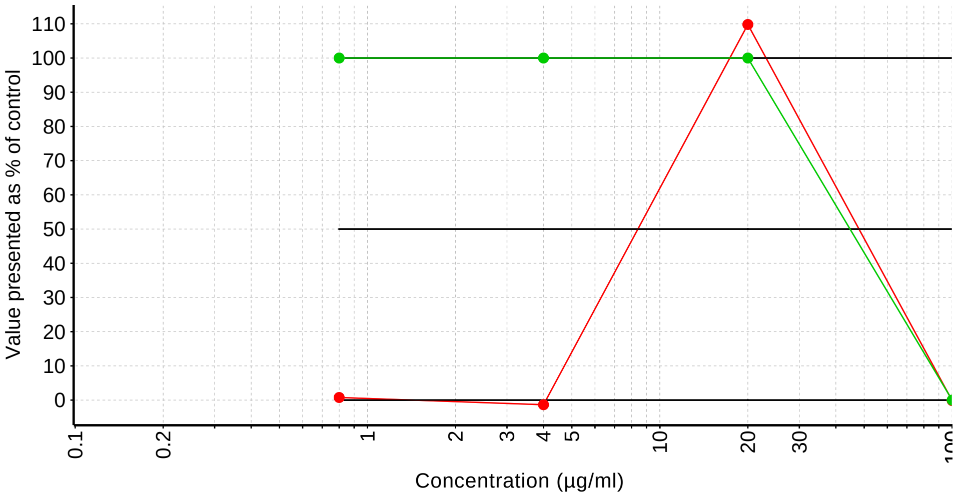

CC50	=	44.7
EC50	=	8.41
EC90	=	15
SI	=	5.32
SS	=	18.9
TI	=	13.7
Max %		100.00
[] Max		18.6
Approved AV		1
Interesting AV		0
Page		19 / 96

Anti-Viral
Anti-Metabolic

Export chart data to CSV

Summary values			
**Statistic**	**CC50**	**EC50**	**EC90**
Median	= 46.4	= 8.41	= 15
Med.Abs.Dev.	1.64		
Mean	= 46.4	= 8.41	= 15
Stdev.	2.32		

Compound	Virus			Cell		AV Method		AM Method	
Primary code	Species	Type	Strain	Type	Subtype	Method	Type	Method	Type
BAVARIE1_0020	Chikungunya virus	No Type	899	Vero	A	Absorbance	MTS - 498nm	Microscopy	Tox scoring

Needs more data.

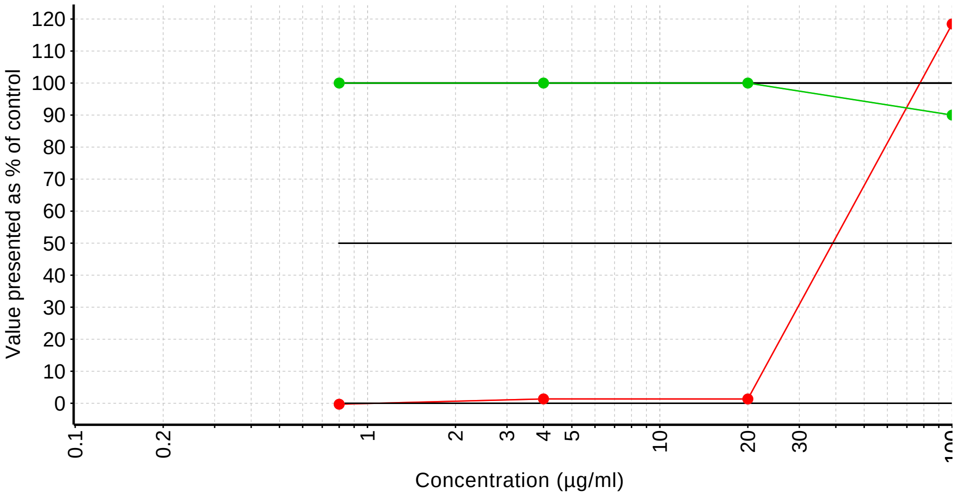

CC50	>	100
EC50	=	39
EC90	=	67.6
SI	>	2.56
SS	>	10.3
TI	>	4.22
Max %		92.24
[] Max		82.1
Approved AV		1
Interesting AV		0
Page		20 / 96

Anti-Viral
Anti-Metabolic

Export chart data to CSV

Summary values			
**Statistic**	**CC50**	**EC50**	**EC90**
Median	> 100	= 39	= 67.6
Med.Abs.Dev.			
Mean	> 100	= 39	= 67.6
Stdev.			

**Compound**	**Virus**			**Cell**		**AV Method**		**AM Method**	
Primary code	Species	Type	Strain	Type	Subtype	Method	Type	Method	Type
BAVARIE1_0021	Chikungunya virus	No Type	899	Vero	A	Absorbance	MTS - 498nm	Microscopy	Tox scoring

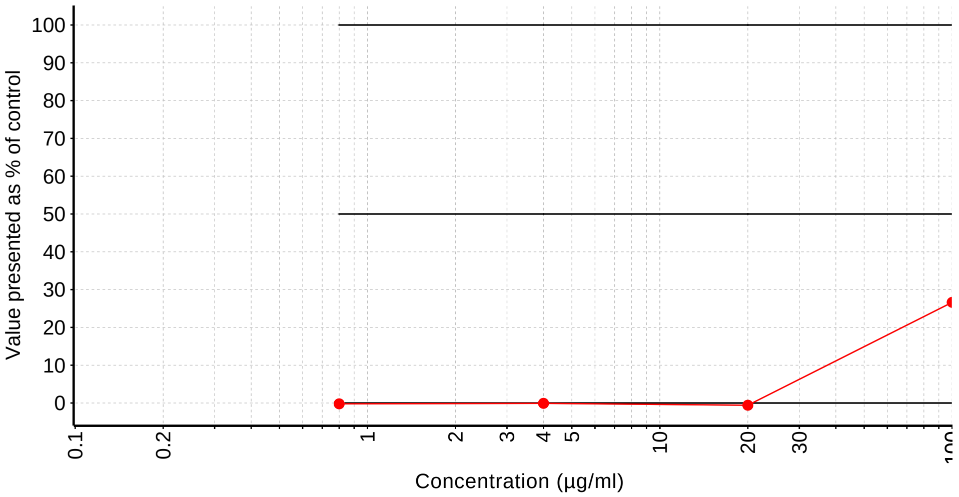

$CC50_{av}$		
EC50	>	100
EC90	>	100
SI_{av}		
SS_{av}		
TI_{av}		
Max %		26.63
[] Max		100
Approved AV		1
Interesting AV		0
Page		21 / 96

Anti-Viral
Anti-Metabolic

Export chart data to CSV

**Summary values**			
**Statistic**	**CC50**	**EC50**	**EC90**
Median		> 100	> 100
Med.Abs.Dev.			
Mean		> 100	> 100
Stdev.			

Compound	Virus			Cell		AV Method		AM Method	
Primary code	Species	Type	Strain	Type	Subtype	Method	Type	Method	Type
BAVARIE1_0022	Chikungunya virus	No Type	899	Vero	A	Absorbance	MTS - 498nm	Microscopy	Tox scoring

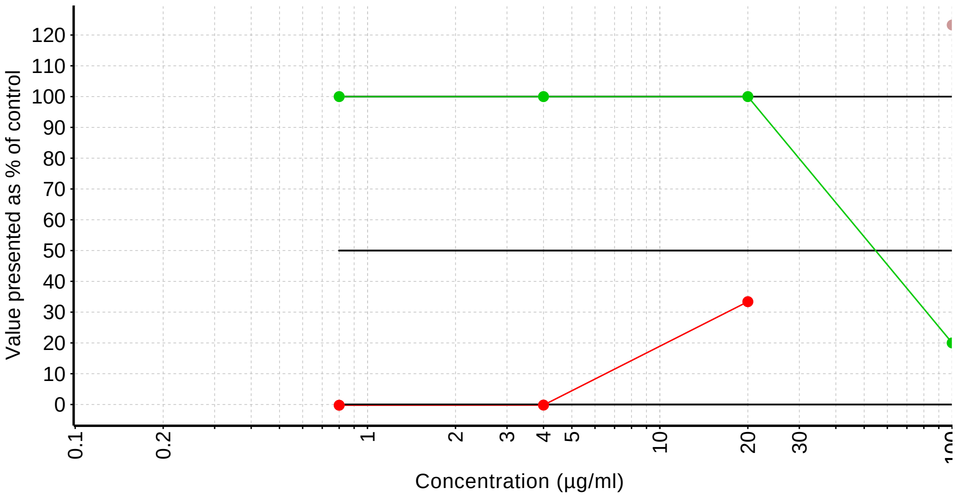

CC50	= 54.7
EC50	
EC90	
SI	
SS	
TI	
Max %	33.40
[] Max	20
Approved AV	1
Interesting AV	0
Page	22 / 96

Anti-Viral
Anti-Metabolic

Export chart data to CSV

Summary values			
**Statistic**	**CC50**	**EC50**	**EC90**
Median	= 54.7	> 20	> 20
Med.Abs.Dev.			
Mean	= 54.7	> 20	> 20
Stdev.			

Compound	Virus			Cell		AV Method		AM Method	
Primary code	Species	Type	Strain	Type	Subtype	Method	Type	Method	Type
BAVARIE1_0023	Chikungunya virus	No Type	899	Vero	A	Absorbance	MTS - 498nm	Microscopy	Tox scoring

Needs more data.

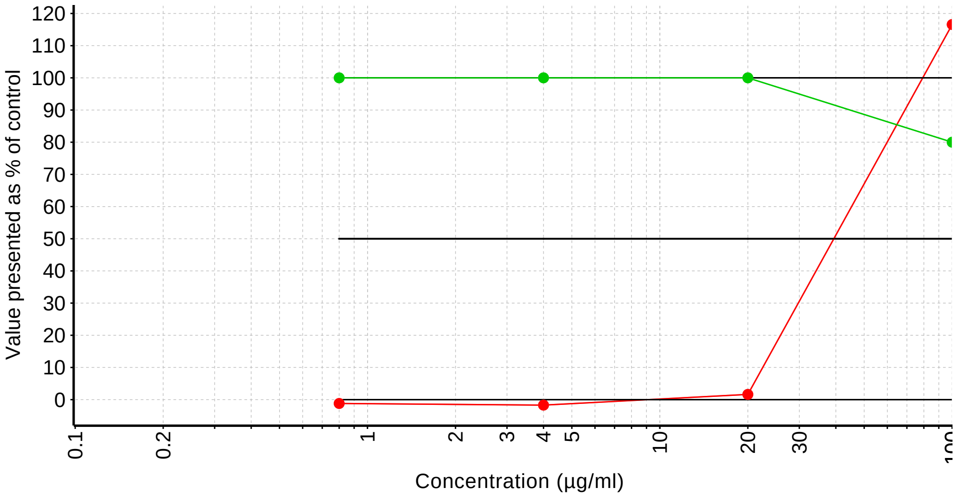

CC50	>	100
EC50	=	39.4
EC90		
SI	>	2.54
SS	>	8.76
TI	>	3.55
Max %		85.42
[] Max		78.3
Approved AV		1
Interesting AV		0
Page		23 / 96

Anti-Viral
Anti-Metabolic

Export chart data to CSV

Summary values			
**Statistic**	**CC50**	**EC50**	**EC90**
Median	> 100	= 39.4	= 68.9
Med.Abs.Dev.			
Mean	> 100	= 39.4	= 68.9
Stdev.			

**Compound**	**Virus**			**Cell**		**AV Method**		**AM Method**	
Primary code	Species	Type	Strain	Type	Subtype	Method	Type	Method	Type
BAVARIE1_0024	Chikungunya virus	No Type	899	Vero	A	Absorbance	MTS - 498nm	Microscopy	Tox scoring

Needs more data.

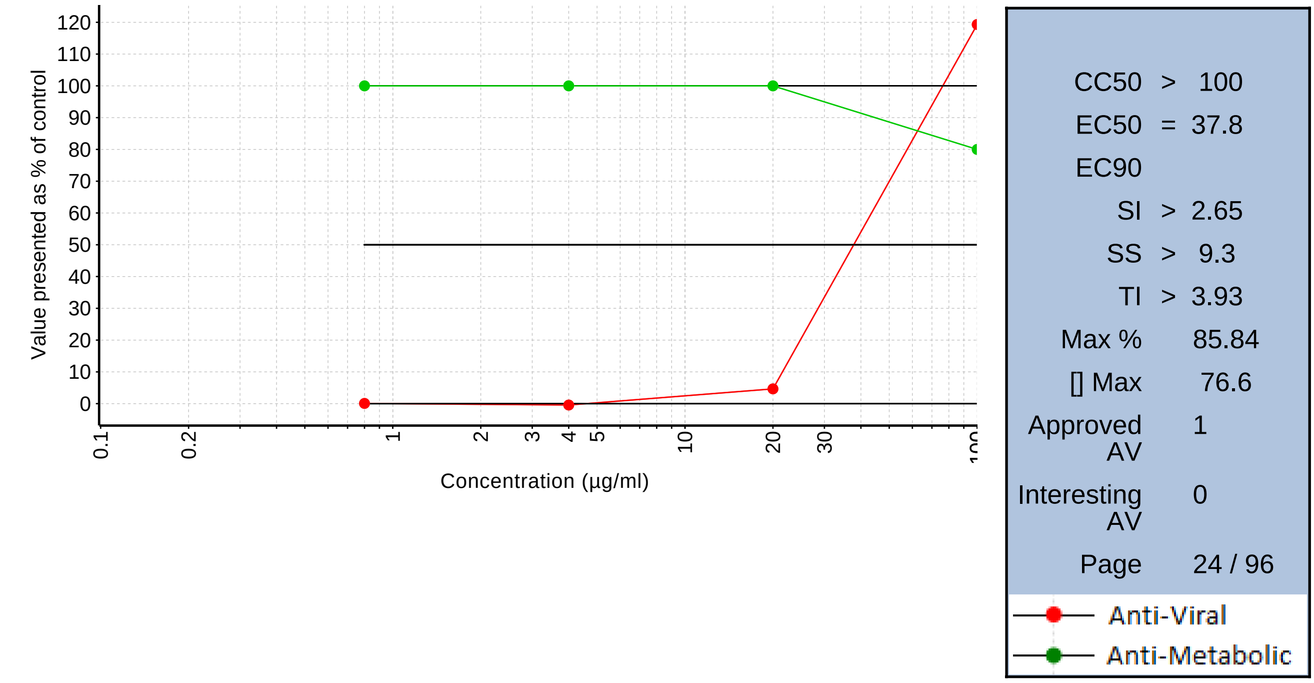

Export chart data to CSV

Summary values			
**Statistic**	**CC50**	**EC50**	**EC90**
Median	> 100	= 37.8	= 66.3
Med.Abs.Dev.			
Mean	> 100	= 37.8	= 66.3
Stdev.			

**Compound**	**Virus**			**Cell**		**AV Method**		**AM Method**	
Primary code	Species	Type	Strain	Type	Subtype	Method	Type	Method	Type
BAVARIE1_0025	Chikungunya virus	No Type	899	Vero	A	Absorbance	MTS - 498nm	Microscopy	Tox scoring

Needs more data.

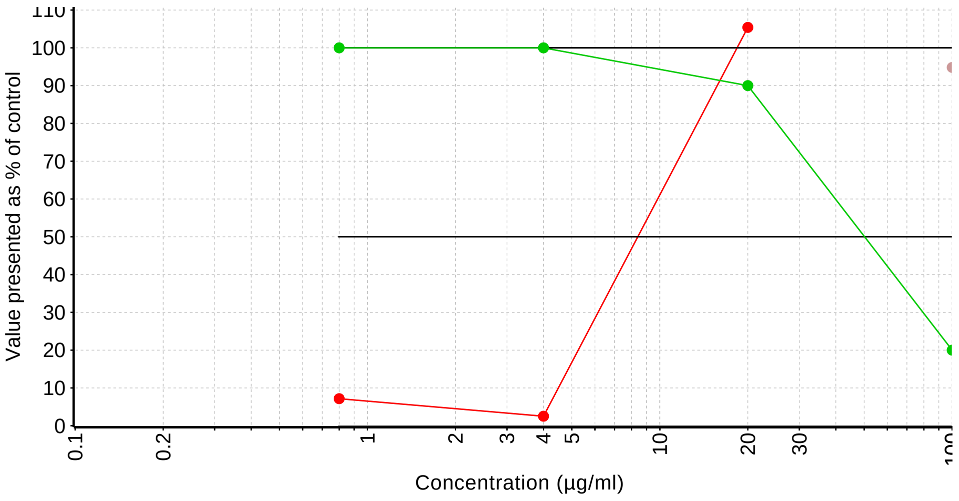

CC50 = 50.2
EC50 = 8.41
EC90 = 15.7
SI = 5.97
SS = 17
TI = 13.2
Max % 91.37
[] Max 17.8
Approved AV 1
Interesting AV 1
Page 25 / 96

Anti-Viral
Anti-Metabolic

Export chart data to CSV

**Summary values**			
**Statistic**	**CC50**	**EC50**	**EC90**
Median	= 50.2	= 8.41	= 15.7
Med.Abs.Dev.			
Mean	= 50.2	= 8.41	= 15.7
Stdev.			

Compound	Virus			Cell		AV Method		AM Method	
Primary code	Species	Type	Strain	Type	Subtype	Method	Type	Method	Type
BAVARIE1_0026	Chikungunya virus	No Type	899	Vero	A	Absorbance	MTS - 498nm	Microscopy	Tox scoring

Needs more data.

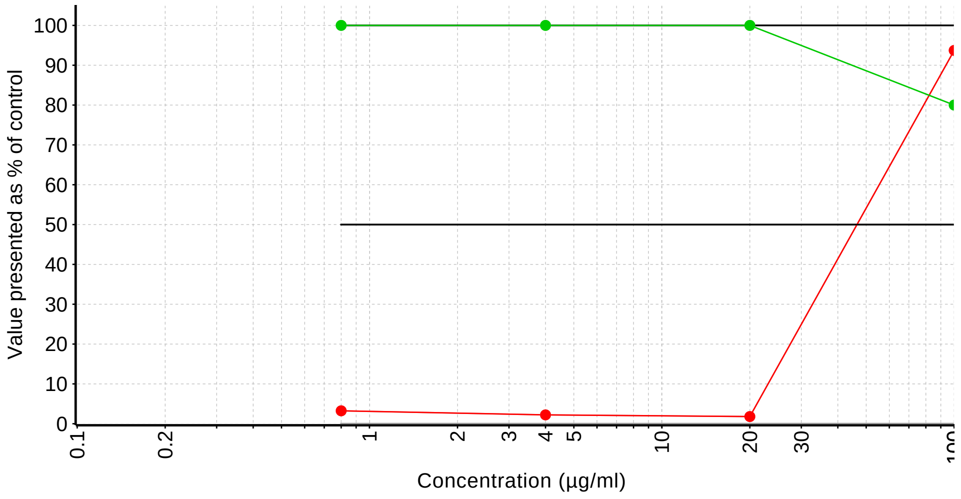

CC50	>	100
EC50	=	46.5
EC90		
SI	>	2.15
SS	>	6.07
TI	>	2.02
Max %		82.45
[] Max		90.2
Approved AV		1
Interesting AV		0
Page		26 / 96

Anti-Viral
Anti-Metabolic

Export chart data to CSV

Summary values			
**Statistic**	**CC50**	**EC50**	**EC90**
Median	> 100	= 46.5	= 93.7
Med.Abs.Dev.			
Mean	> 100	= 46.5	= 93.7
Stdev.			

Compound	Virus			Cell		AV Method		AM Method	
Primary code	Species	Type	Strain	Type	Subtype	Method	Type	Method	Type
BAVARIE1_0027	Chikungunya virus	No Type	899	Vero	A	Absorbance	MTS - 498nm	Microscopy	Tox scoring

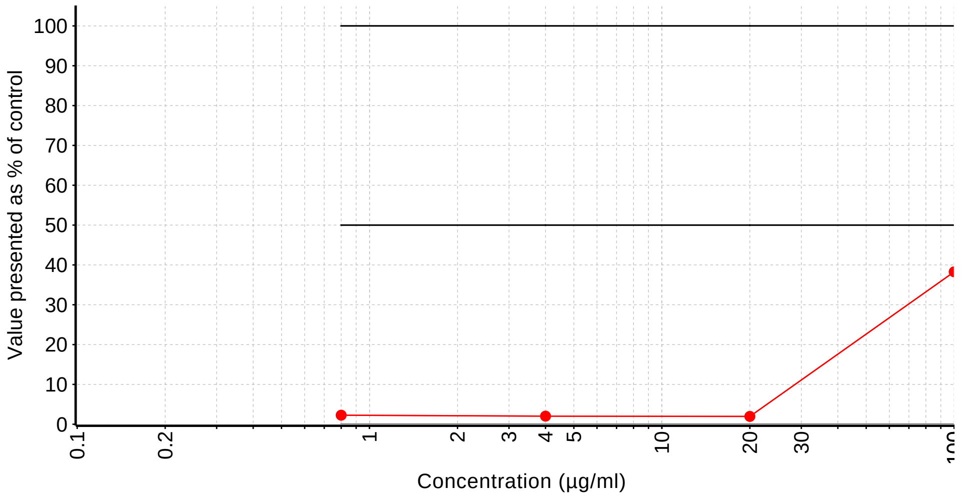

$CC50_{av}$		
EC50	>	100
EC90	>	100
SI_{av}		
SS_{av}		
TI_{av}		
Max %		38.26
[] Max		100
Approved AV		1
Interesting AV		0
Page		27 / 96

Anti-Viral
Anti-Metabolic

Export chart data to CSV

Summary values			
**Statistic**	**CC50**	**EC50**	**EC90**
Median		> 100	> 100
Med.Abs.Dev.			
Mean		> 100	> 100
Stdev.			

Compound	Virus			Cell		AV Method		AM Method	
Primary code	Species	Type	Strain	Type	Subtype	Method	Type	Method	Type
BAVARIE1_0028	Chikungunya virus	No Type	899	Vero	A	Absorbance	MTS - 498nm	Microscopy	Tox scoring

Needs more data.

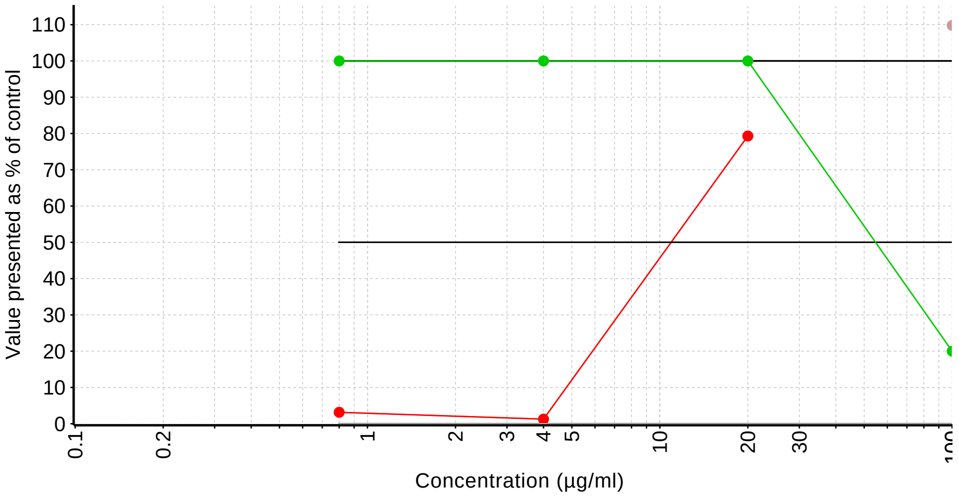

CC50 = 54.7
EC50 = 10.9
EC90
SI = 5
SS = 10.2
TI = 7.17
Max % 79.30
[] Max 20
Approved AV 1
Interesting AV 0
Page 28 / 96

Anti-Viral
Anti-Metabolic

Export chart data to CSV

Summary values			
**Statistic**	**CC50**	**EC50**	**EC90**
Median	= 54.7	= 10.9	> 20
Med.Abs.Dev.			
Mean	= 54.7	= 10.9	> 20
Stdev.			

Compound	Virus			Cell		AV Method		AM Method	
Primary code	Species	Type	Strain	Type	Subtype	Method	Type	Method	Type
BAVARIE1_0029	Chikungunya virus	No Type	899	Vero	A	Absorbance	MTS - 498nm	Microscopy	Tox scoring

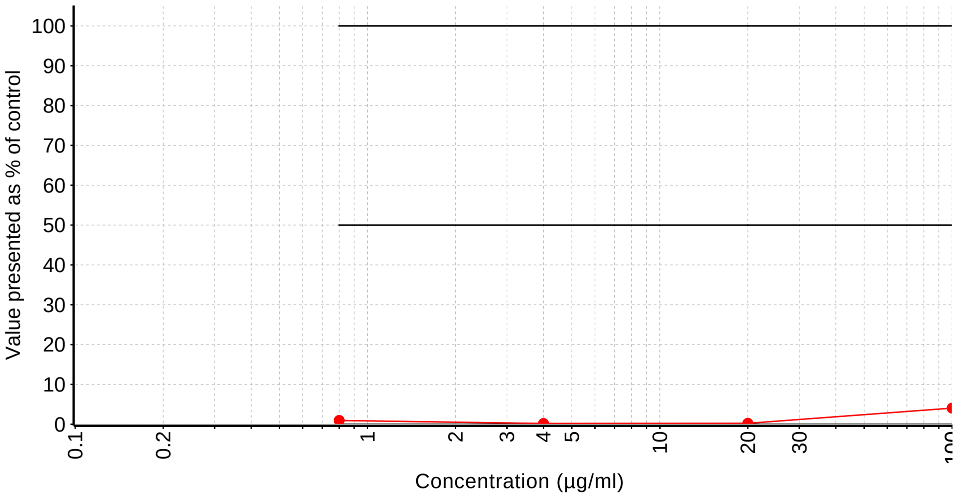

$CC50_{av}$		
EC50	>	100
EC90	>	100
SI_{av}		
SS_{av}		
TI_{av}		
Max %		4.05
[] Max		100
Approved AV		1
Interesting AV		0
Page		29 / 96

Anti-Viral
Anti-Metabolic

Export chart data to CSV

Summary values			
**Statistic**	**CC50**	**EC50**	**EC90**
Median		> 100	> 100
Med.Abs.Dev.			
Mean		> 100	> 100
Stdev.			

Compound	Virus			Cell		AV Method		AM Method	
Primary code	Species	Type	Strain	Type	Subtype	Method	Type	Method	Type
BAVARIE1_0030	Chikungunya virus	No Type	899	Vero	A	Absorbance	MTS - 498nm	Microscopy	Tox scoring

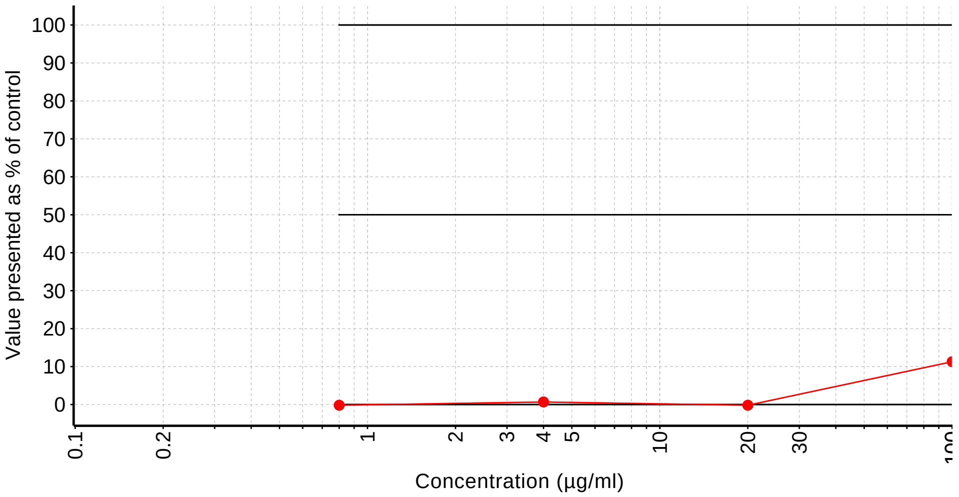

$CC50_{av}$		
EC50	>	100
EC90	>	100
SI_{av}		
SS_{av}		
TI_{av}		
Max %		11.28
[] Max		100
Approved AV		1
Interesting AV		0
Page		30 / 96

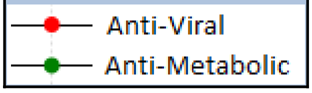

Export chart data to CSV

Summary values			
**Statistic**	**CC50**	**EC50**	**EC90**
Median		> 100	> 100
Med.Abs.Dev.			
Mean		> 100	> 100
Stdev.			

**Compound**	**Virus**			**Cell**		**AV Method**		**AM Method**	
Primary code	Species	Type	Strain	Type	Subtype	Method	Type	Method	Type
BAVARIE1_0031	Chikungunya virus	No Type	899	Vero	A	Absorbance	MTS - 498nm	Microscopy	Tox scoring

Needs more data.

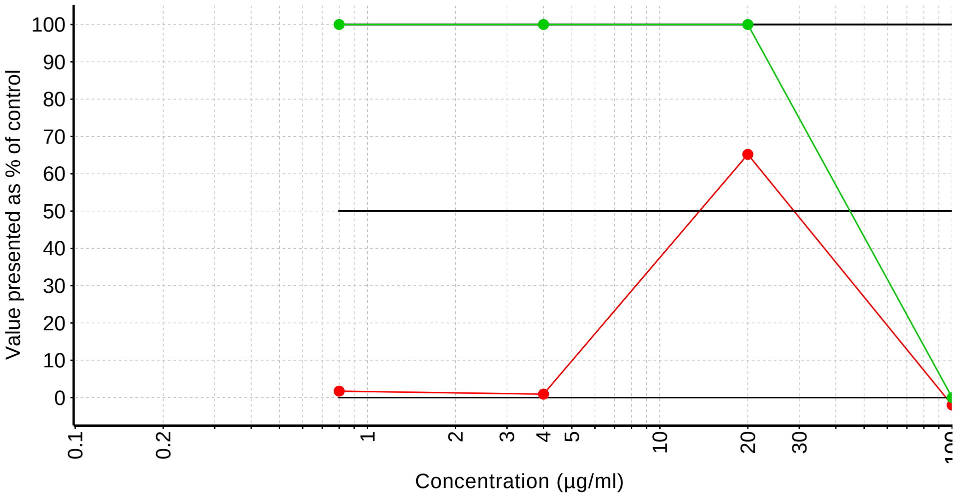

CC50	=	44.7
EC50	=	13.7
EC90		
SI	=	3.27
SS	=	2.46
TI	=	1.26
Max %		65.20
[] Max		20
Approved AV		1
Interesting AV		0
Page		31 / 96

Anti-Viral
Anti-Metabolic

Export chart data to CSV

**Summary values**			
**Statistic**	**CC50**	**EC50**	**EC90**
Median	= 36.8	= 13.7	
Med.Abs.Dev.	7.97		
Mean	= 36.8	= 13.7	
Stdev.	11.3		

Compound	Virus			Cell		AV Method		AM Method	
Primary code	Species	Type	Strain	Type	Subtype	Method	Type	Method	Type
BAVARIE1_0032	Chikungunya virus	No Type	899	Vero	A	Absorbance	MTS - 498nm	Microscopy	Tox scoring

Needs more data.

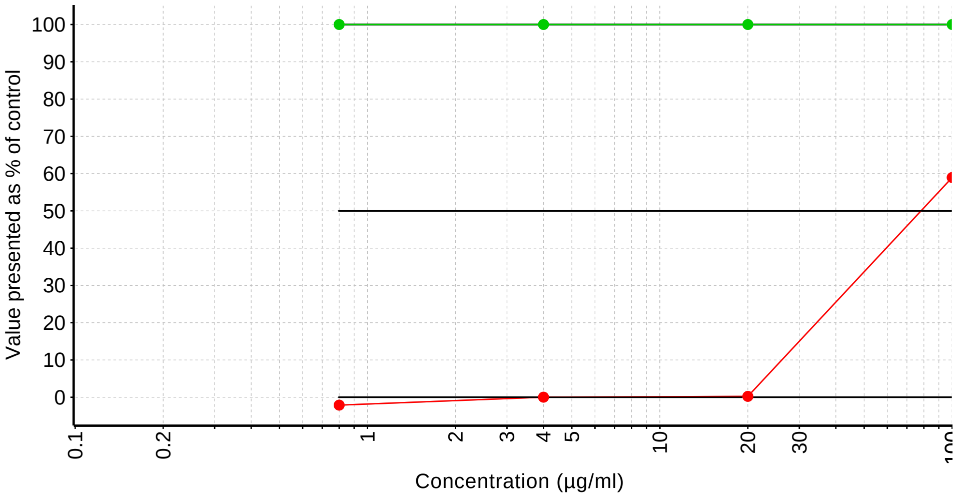

CC50	>	100
EC50	=	78.2
EC90	>	100
SI	>	1.28
SS	>	0.478
TI	>	0.0509
Max %		58.96
[] Max		100
Approved AV		1
Interesting AV		0
Page		32 / 96

Anti-Viral
Anti-Metabolic

Export chart data to CSV

Summary values			
**Statistic**	**CC50**	**EC50**	**EC90**
Median	> 100	= 78.2	> 100
Med.Abs.Dev.			
Mean	> 100	= 78.2	> 100
Stdev.			

Compound	Virus			Cell		AV Method		AM Method	
Primary code	Species	Type	Strain	Type	Subtype	Method	Type	Method	Type
BAVARIE1_0033	Chikungunya virus	No Type	899	Vero	A	Absorbance	MTS - 498nm	Microscopy	Tox scoring

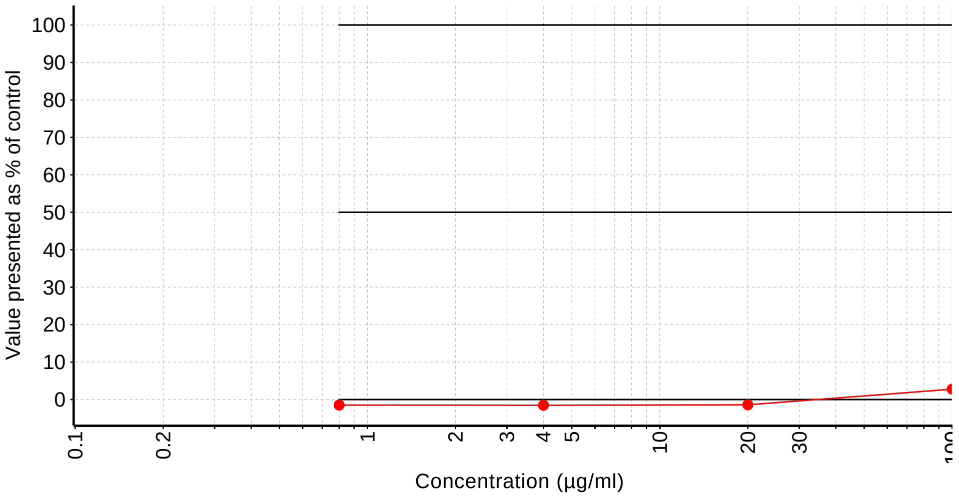

$CC50_{av}$		
EC50	>	100
EC90	>	100
SI_{av}		
SS_{av}		
TI_{av}		
Max %		2.77
[] Max		100
Approved AV		1
Interesting AV		0
Page		33 / 96

Anti-Viral
Anti-Metabolic

Export chart data to CSV

Summary values			
**Statistic**	**CC50**	**EC50**	**EC90**
Median		> 100	> 100
Med.Abs.Dev.			
Mean		> 100	> 100
Stdev.			

**Compound**	**Virus**			**Cell**		**AV Method**		**AM Method**	
Primary code	Species	Type	Strain	Type	Subtype	Method	Type	Method	Type
BAVARIE1_0034	Chikungunya virus	No Type	899	Vero	A	Absorbance	MTS - 498nm	Microscopy	Tox scoring

Needs more data.

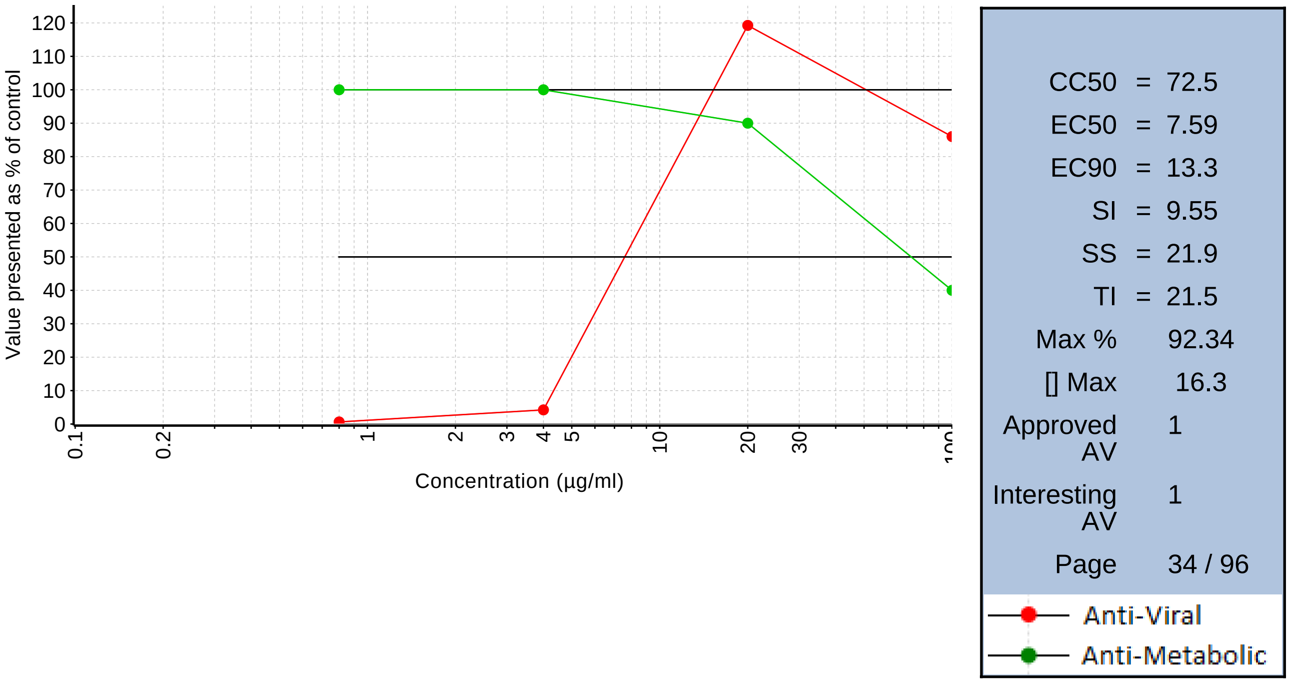

Export chart data to CSV

Summary values			
**Statistic**	**CC50**	**EC50**	**EC90**
Median	= 72.5	= 7.59	= 13.3
Med.Abs.Dev.			
Mean	= 72.5	= 7.59	= 13.3
Stdev.			

Compound	Virus			Cell		AV Method		AM Method	
Primary code	Species	Type	Strain	Type	Subtype	Method	Type	Method	Type
BAVARIE1_0035	Chikungunya virus	No Type	899	Vero	A	Absorbance	MTS - 498nm	Microscopy	Tox scoring

Needs more data.

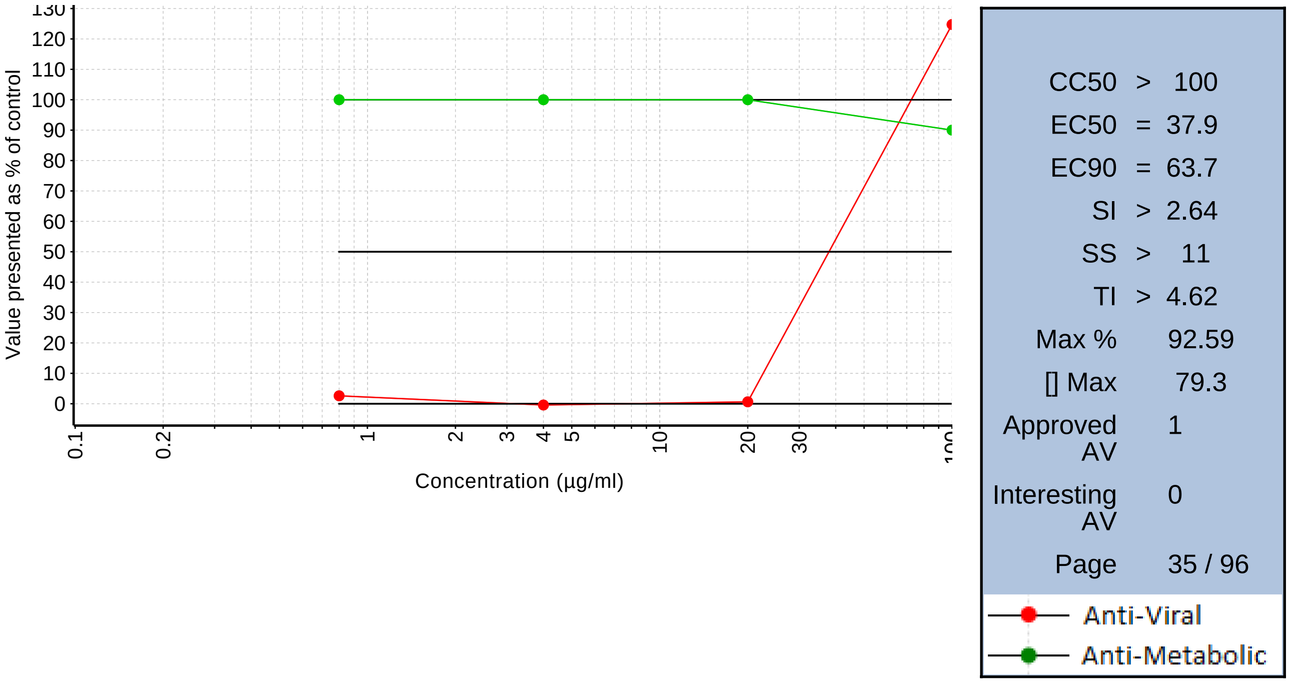

Export chart data to CSV

Summary values			
**Statistic**	**CC50**	**EC50**	**EC90**
Median	> 100	= 37.9	= 63.7
Med.Abs.Dev.			
Mean	> 100	= 37.9	= 63.7
Stdev.			

Compound	Virus			Cell		AV Method		AM Method	
Primary code	Species	Type	Strain	Type	Subtype	Method	Type	Method	Type
BAVARIE1_0036	Chikungunya virus	No Type	899	Vero	A	Absorbance	MTS - 498nm	Microscopy	Tox scoring

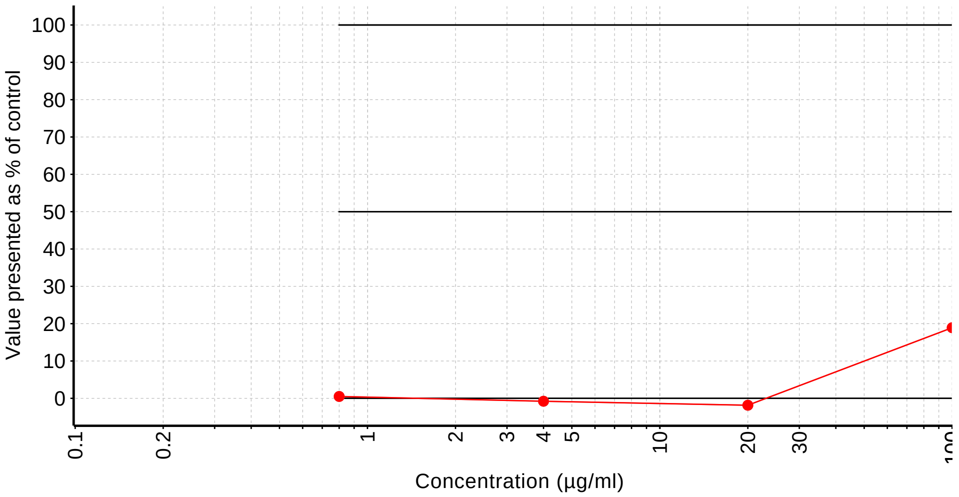

$CC50_{av}$		
EC50	>	100
EC90	>	100
SI_{av}		
SS_{av}		
TI_{av}		
Max %		18.92
[] Max		100
Approved AV		1
Interesting AV		0
Page		36 / 96

Anti-Viral
Anti-Metabolic

Export chart data to CSV

Summary values			
**Statistic**	**CC50**	**EC50**	**EC90**
Median		> 100	> 100
Med.Abs.Dev.			
Mean		> 100	> 100
Stdev.			

**Compound**	**Virus**			**Cell**		**AV Method**		**AM Method**	
Primary code	Species	Type	Strain	Type	Subtype	Method	Type	Method	Type
BAVARIE1_0037	Chikungunya virus	No Type	899	Vero	A	Absorbance	MTS - 498nm	Microscopy	Tox scoring

Needs more data.

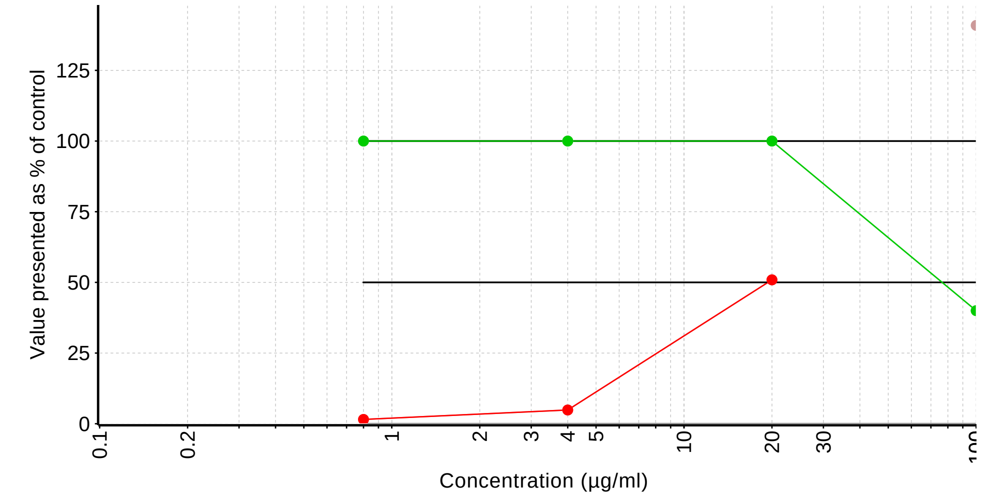

CC50	=	76.5
EC50	=	19.4
EC90		
SI	=	3.95
SS	=	0.268
TI	=	0.16
Max %		50.90
[] Max		20
Approved AV		1
Interesting AV		0
Page		37 / 96

Anti-Viral
Anti-Metabolic

Export chart data to CSV

**Summary values**			
**Statistic**	**CC50**	**EC50**	**EC90**
Median	= 76.5	= 19.4	> 20
Med.Abs.Dev.			
Mean	= 76.5	= 19.4	> 20
Stdev.			

Compound	Virus			Cell		AV Method		AM Method	
Primary code	Species	Type	Strain	Type	Subtype	Method	Type	Method	Type
BAVARIE1_0038	Chikungunya virus	No Type	899	Vero	A	Absorbance	MTS - 498nm	Microscopy	Tox scoring

Needs more data.

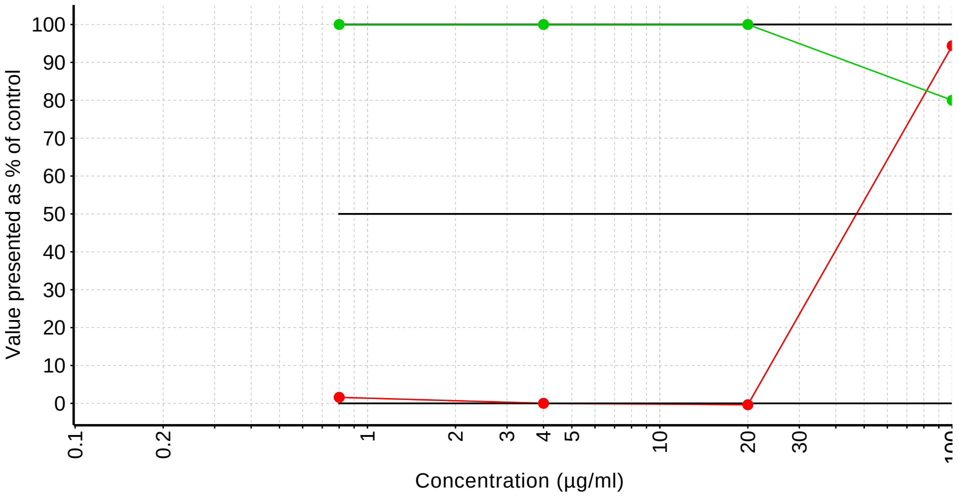

CC50	>	100
EC50	=	47
EC90		
SI	>	2.13
SS	>	6.01
TI	>	1.97
Max %		82.51
[] Max		90
Approved AV		1
Interesting AV		0
Page		38 / 96

Anti-Viral
Anti-Metabolic

Export chart data to CSV

Summary values			
**Statistic**	**CC50**	**EC50**	**EC90**
Median	> 100	= 47	= 92.8
Med.Abs.Dev.			
Mean	> 100	= 47	= 92.8
Stdev.			

**Compound**	**Virus**			**Cell**		**AV Method**		**AM Method**	
Primary code	Species	Type	Strain	Type	Subtype	Method	Type	Method	Type
BAVARIE1_0039	Chikungunya virus	No Type	899	Vero	A	Absorbance	MTS - 498nm	Microscopy	Tox scoring

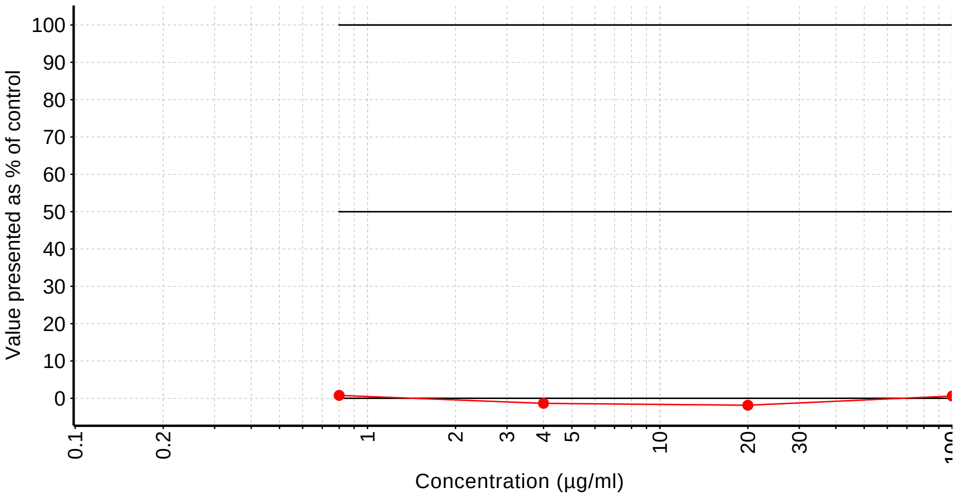

$CC50_{av}$		
EC50	>	100
EC90	>	100
SI_{av}		
SS_{av}		
TI_{av}		
Max %		0.78
[] Max		0.8
Approved AV		1
Interesting AV		0
Page		39 / 96

Anti-Viral
Anti-Metabolic

Export chart data to CSV

**Summary values**			
**Statistic**	**CC50**	**EC50**	**EC90**
Median		> 100	> 100
Med.Abs.Dev.			
Mean		> 100	> 100
Stdev.			

**Compound**	**Virus**			**Cell**		**AV Method**		**AM Method**	
Primary code	Species	Type	Strain	Type	Subtype	Method	Type	Method	Type
BAVARIE1_0040	Chikungunya virus	No Type	899	Vero	A	Absorbance	MTS - 498nm	Microscopy	Tox scoring

Needs more data.

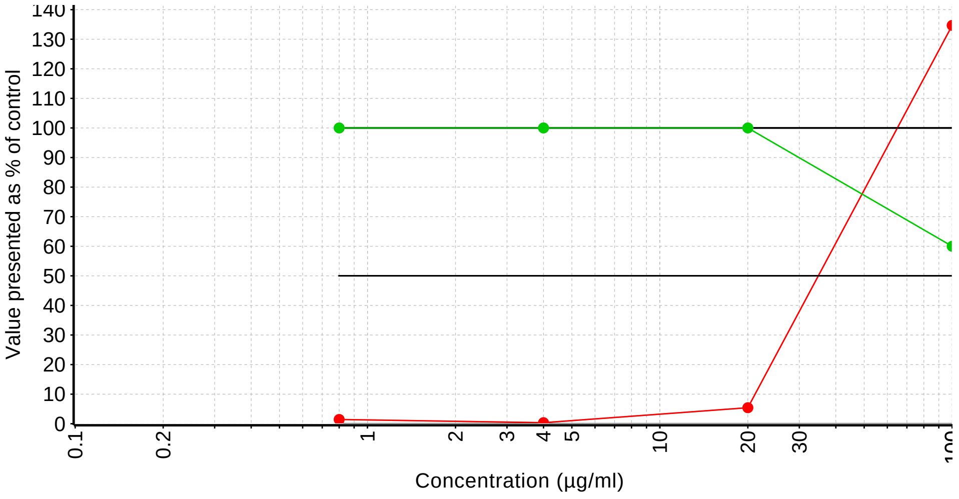

CC50	>	100
EC50	=	34.8
EC90		
SI	>	2.87
SS	>	7.28
TI	>	3.33
Max %		77.65
[] Max		64.7
Approved AV		1
Interesting AV		0
Page		40 / 96

Anti-Viral
Anti-Metabolic

Export chart data to CSV

Summary values			
**Statistic**	**CC50**	**EC50**	**EC90**
Median	> 100	= 34.8	= 57.3
Med.Abs.Dev.			
Mean	> 100	= 34.8	= 57.3
Stdev.			

Compound	Virus			Cell		AV Method		AM Method	
Primary code	Species	Type	Strain	Type	Subtype	Method	Type	Method	Type
BAVARIE1_0041	Chikungunya virus	No Type	899	Vero	A	Absorbance	MTS - 498nm	Microscopy	Tox scoring

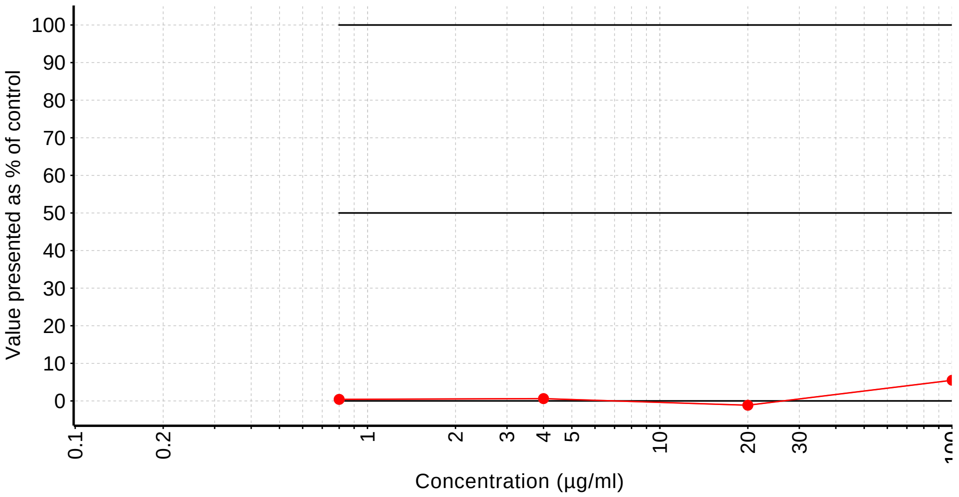

$CC50_{av}$		
EC50	>	100
EC90	>	100
SI_{av}		
SS_{av}		
TI_{av}		
Max %		5.50
[] Max		100
Approved AV		1
Interesting AV		0
Page		41 / 96

Anti-Viral
Anti-Metabolic

Export chart data to CSV

Summary values			
**Statistic**	**CC50**	**EC50**	**EC90**
Median		> 100	> 100
Med.Abs.Dev.			
Mean		> 100	> 100
Stdev.			

**Compound**	**Virus**			**Cell**		**AV Method**		**AM Method**	
Primary code	Species	Type	Strain	Type	Subtype	Method	Type	Method	Type
BAVARIE1_0042	Chikungunya virus	No Type	899	Vero	A	Absorbance	MTS - 498nm	Microscopy	Tox scoring

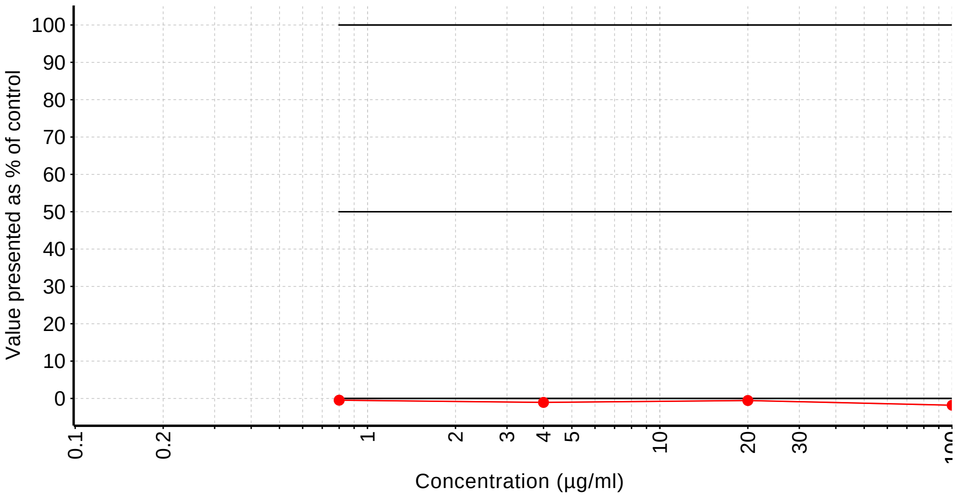

$CC50_{av}$		
EC50	>	100
EC90	>	100
SI_{av}		
SS_{av}		
TI_{av}		
Max %		-0.47
[] Max		0.8
Approved AV		1
Interesting AV		0
Page		42 / 96

Anti-Viral
Anti-Metabolic

Export chart data to CSV

**Summary values**			
**Statistic**	**CC50**	**EC50**	**EC90**
Median		> 100	> 100
Med.Abs.Dev.			
Mean		> 100	> 100
Stdev.			

Compound	Virus			Cell		AV Method		AM Method	
Primary code	Species	Type	Strain	Type	Subtype	Method	Type	Method	Type
BAVARIE1_0043	Chikungunya virus	No Type	899	Vero	A	Absorbance	MTS - 498nm	Microscopy	Tox scoring

Needs more data.

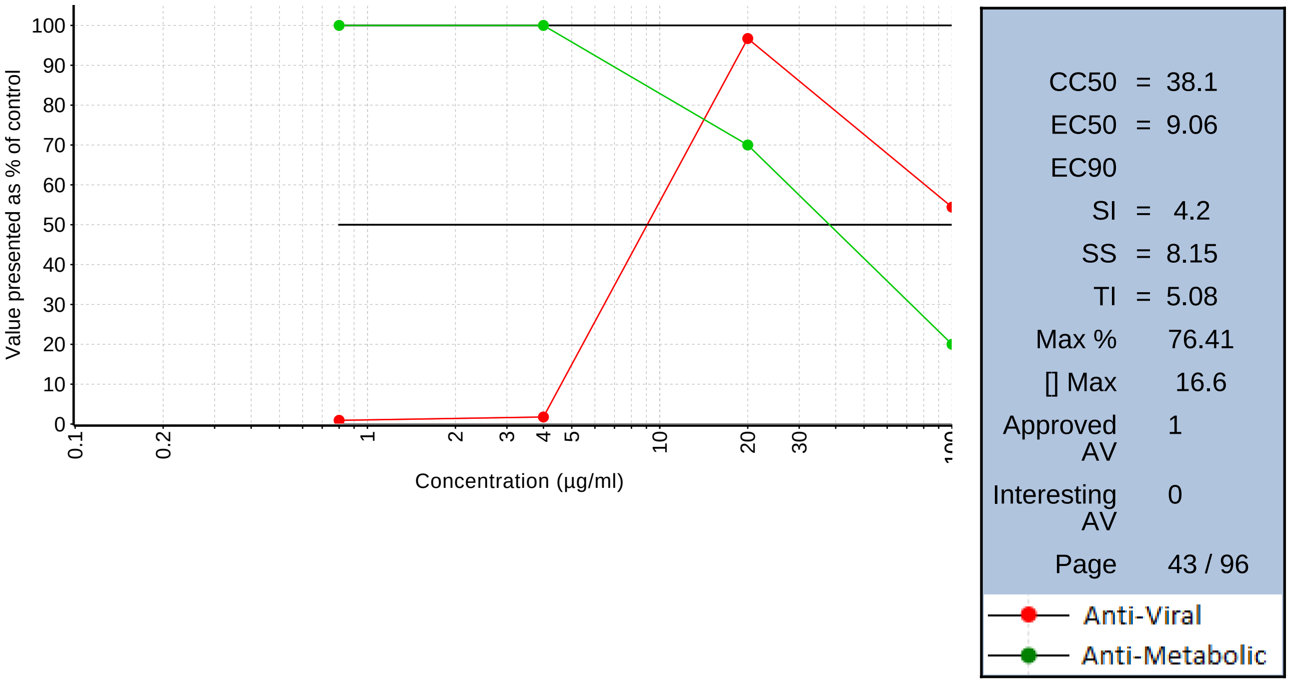

Export chart data to CSV

Summary values			
**Statistic**	**CC50**	**EC50**	**EC90**
Median	= 38.1	= 9.06	= 17.8
Med.Abs.Dev.			
Mean	= 38.1	= 9.06	= 17.8
Stdev.			

Compound	Virus			Cell		AV Method		AM Method	
Primary code	Species	Type	Strain	Type	Subtype	Method	Type	Method	Type
BAVARIE1_0044	Chikungunya virus	No Type	899	Vero	A	Absorbance	MTS - 498nm	Microscopy	Tox scoring

Needs more data.

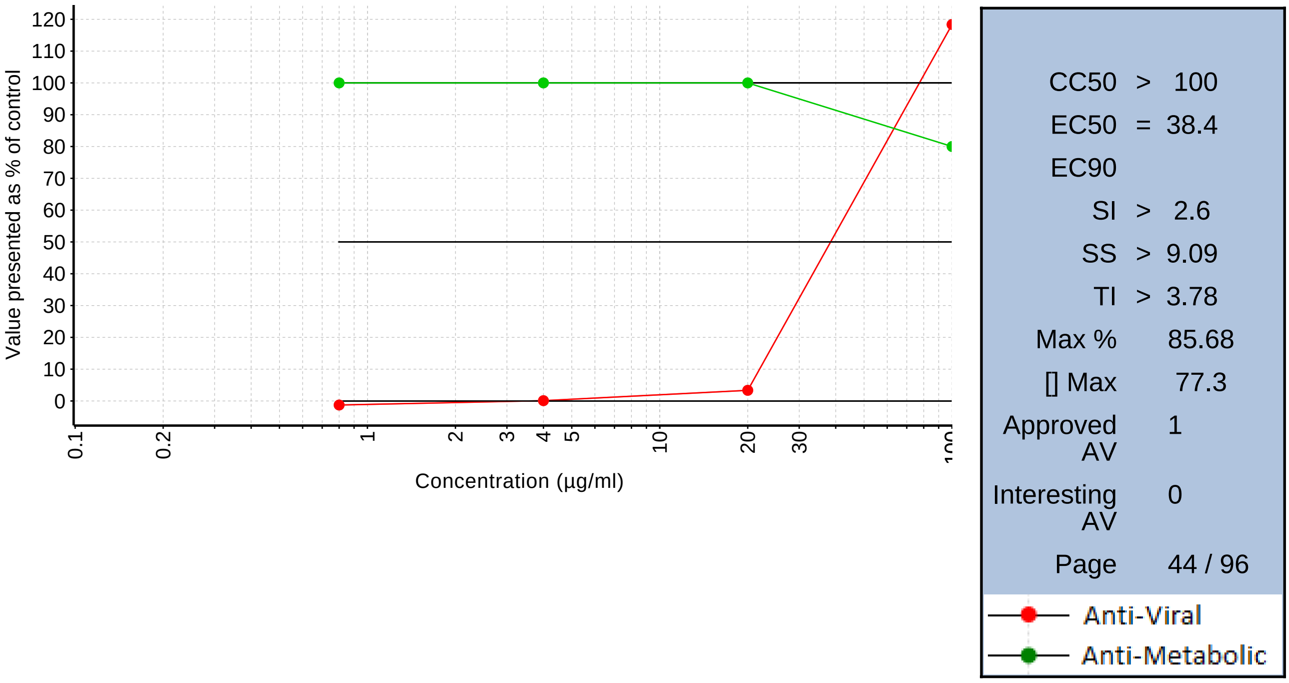

Export chart data to CSV

Summary values			
**Statistic**	**CC50**	**EC50**	**EC90**
Median	> 100	= 38.4	= 67.2
Med.Abs.Dev.			
Mean	> 100	= 38.4	= 67.2
Stdev.			

Compound	Virus			Cell		AV Method		AM Method	
Primary code	Species	Type	Strain	Type	Subtype	Method	Type	Method	Type
BAVARIE1_0045	Chikungunya virus	No Type	899	Vero	A	Absorbance	MTS - 498nm	Microscopy	Tox scoring

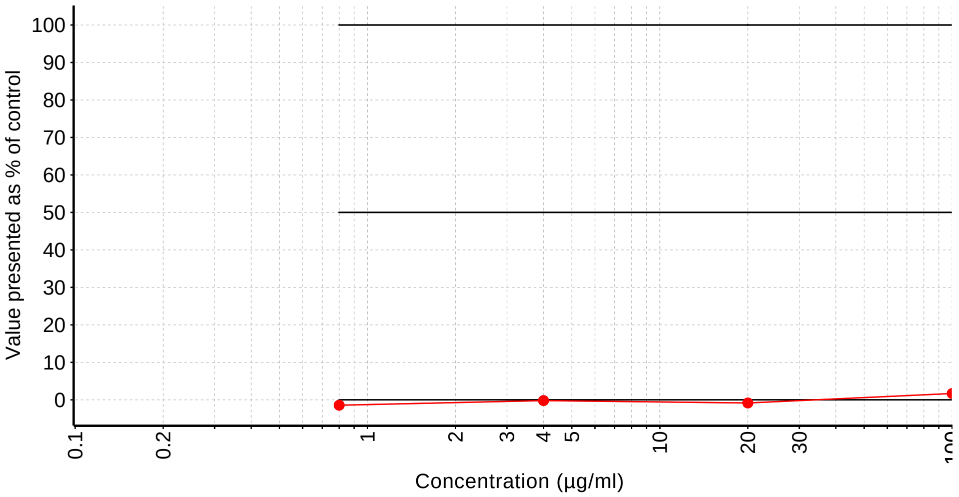

$CC50_{av}$		
EC50	>	100
EC90	>	100
SI_{av}		
SS_{av}		
TI_{av}		
Max %		1.69
[] Max		100
Approved AV		1
Interesting AV		0
Page		45 / 96

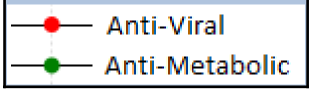

Export chart data to CSV

Summary values			
**Statistic**	**CC50**	**EC50**	**EC90**
Median		> 100	> 100
Med.Abs.Dev.			
Mean		> 100	> 100
Stdev.			

Compound	Virus			Cell		AV Method		AM Method	
Primary code	Species	Type	Strain	Type	Subtype	Method	Type	Method	Type
BAVARIE1_0046	Chikungunya virus	No Type	899	Vero	A	Absorbance	MTS - 498nm	Microscopy	Tox scoring

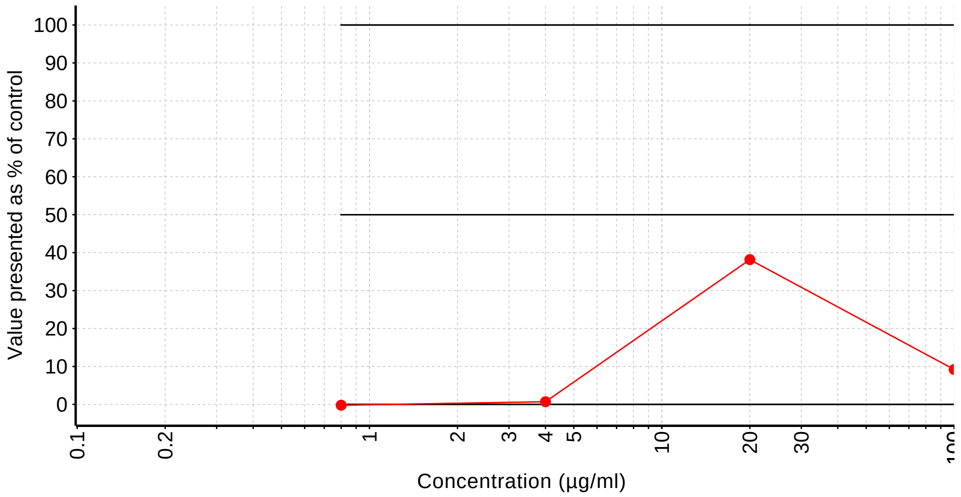

$CC50_{av}$	
EC50	
EC90	
SI_{av}	
SS_{av}	
TI_{av}	
Max %	38.16
[] Max	20
Approved AV	1
Interesting AV	0
Page	46 / 96

Anti-Viral
Anti-Metabolic

Export chart data to CSV

Summary values			
**Statistic**	**CC50**	**EC50**	**EC90**
Median			
Med.Abs.Dev.			
Mean			
Stdev.			

**Compound**	**Virus**			**Cell**		**AV Method**		**AM Method**	
Primary code	Species	Type	Strain	Type	Subtype	Method	Type	Method	Type
BAVARIE1_0047	Chikungunya virus	No Type	899	Vero	A	Absorbance	MTS - 498nm	Microscopy	Tox scoring

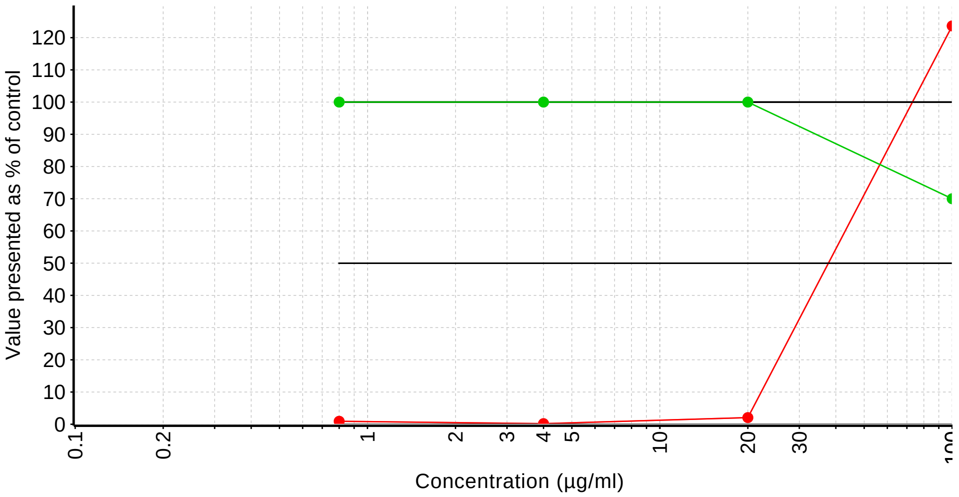

CC50	>	100
EC50	=	37.7
EC90		
SI	>	2.65
SS	>	7.93
TI	>	3.36
Max %		80.62
[] Max		71.7
Approved AV		1
Interesting AV		0
Page		47 / 96

Anti-Viral
Anti-Metabolic

Export chart data to CSV

Summary values			
**Statistic**	**CC50**	**EC50**	**EC90**
Median	> 100	= 37.7	= 64.1
Med.Abs.Dev.			
Mean	> 100	= 37.7	= 64.1
Stdev.			

Compound	Virus			Cell		AV Method		AM Method	
Primary code	Species	Type	Strain	Type	Subtype	Method	Type	Method	Type
BAVARIE1_0048	Chikungunya virus	No Type	899	Vero	A	Absorbance	MTS - 498nm	Microscopy	Tox scoring

Needs more data.

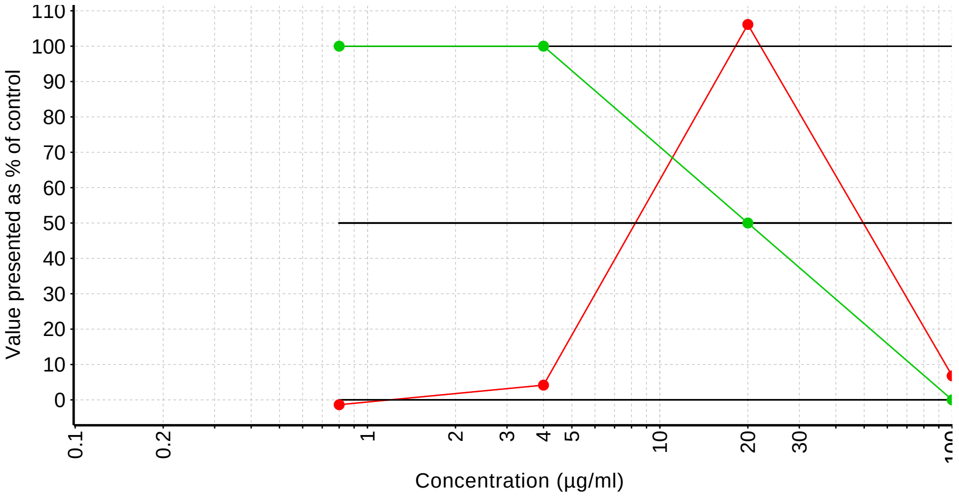

CC50 = 20
EC50 = 8.25
EC90
SI = 2.43
SS = 3.55
TI = 1.37
Max % 68.47
[] Max 14.1
Approved AV 1
Interesting AV 0
Page 48 / 96

Anti-Viral
Anti-Metabolic

Export chart data to CSV

Summary values			
**Statistic**	**CC50**	**EC50**	**EC90**
Median	= 34.8	= 8.25	= 15.5
Med.Abs.Dev.	14.8		
Mean	= 34.8	= 8.25	= 15.5
Stdev.	21		

Compound	Virus			Cell		AV Method		AM Method	
Primary code	Species	Type	Strain	Type	Subtype	Method	Type	Method	Type
BAVARIE1_0049	Chikungunya virus	No Type	899	Vero	A	Absorbance	MTS - 498nm	Microscopy	Tox scoring

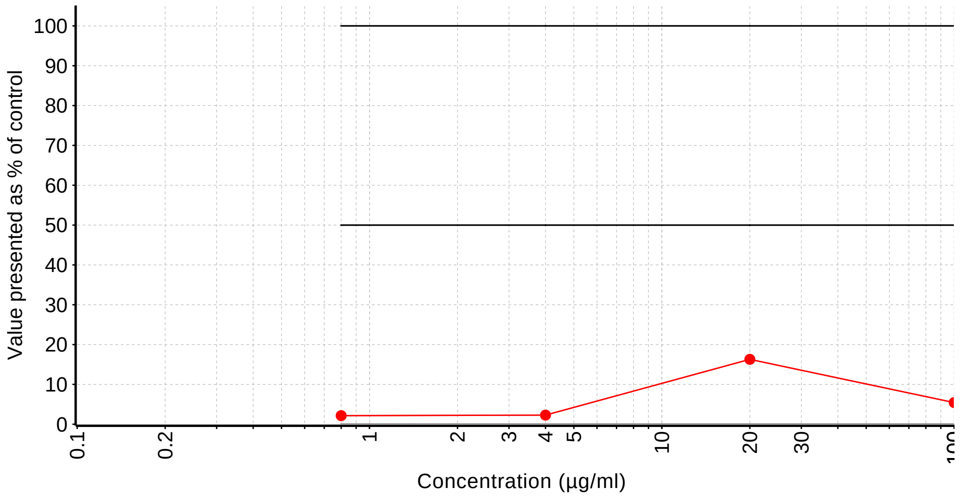

$CC50_{av}$		
EC50	>	100
EC90	>	100
SI_{av}		
SS_{av}		
TI_{av}		
Max %		16.29
[] Max		20
Approved AV		1
Interesting AV		0
Page		49 / 96

Anti-Viral
Anti-Metabolic

Export chart data to CSV

Summary values			
**Statistic**	**CC50**	**EC50**	**EC90**
Median		> 100	> 100
Med.Abs.Dev.			
Mean		> 100	> 100
Stdev.			

**Compound**	**Virus**			**Cell**		**AV Method**		**AM Method**	
Primary code	Species	Type	Strain	Type	Subtype	Method	Type	Method	Type
BAVARIE1_0050	Chikungunya virus	No Type	899	Vero	A	Absorbance	MTS - 498nm	Microscopy	Tox scoring

Needs more data.

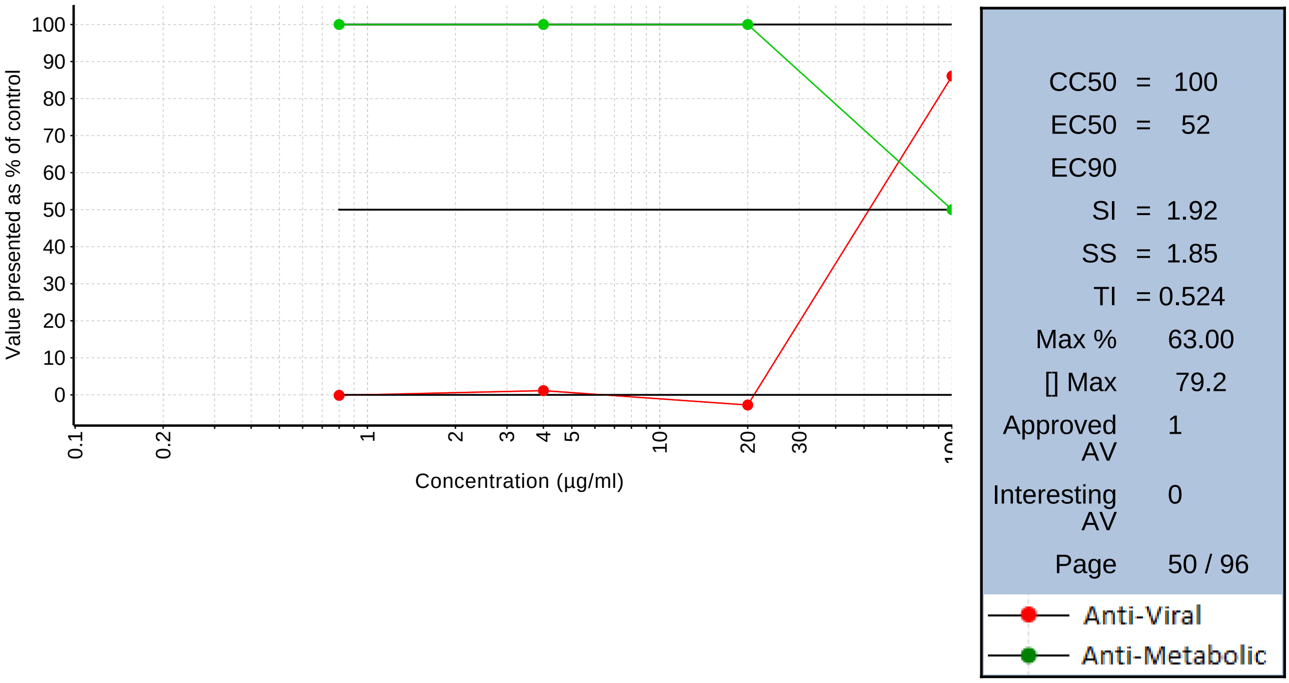

Export chart data to CSV

Summary values			
**Statistic**	**CC50**	**EC50**	**EC90**
Median	= 100	= 52	> 100
Med.Abs.Dev.			
Mean	= 100	= 52	> 100
Stdev.			

**Compound**	**Virus**			**Cell**		**AV Method**		**AM Method**	
Primary code	Species	Type	Strain	Type	Subtype	Method	Type	Method	Type
BAVARIE1_0051	Chikungunya virus	No Type	899	Vero	A	Absorbance	MTS - 498nm	Microscopy	Tox scoring

Needs more data.

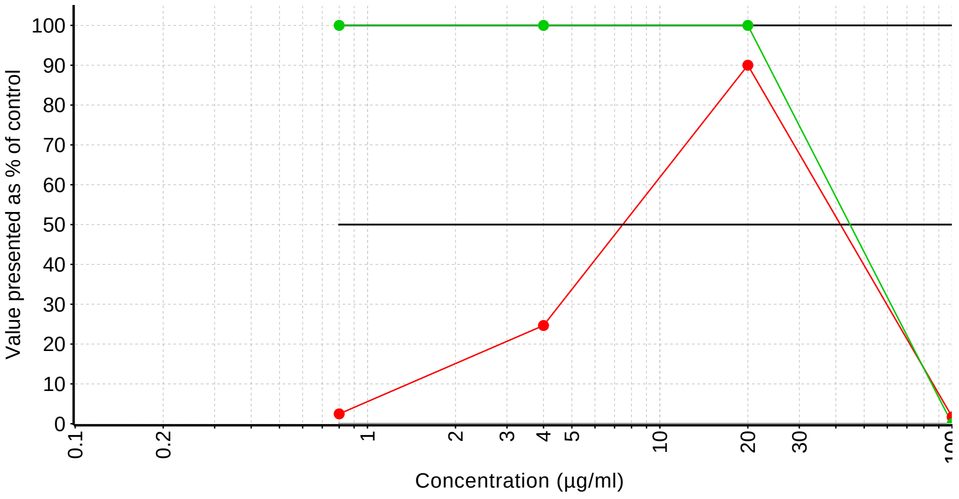

CC50	=	44.7
EC50	=	7.47
EC90	=	20
SI	=	5.99
SS	=	14.9
TI	=	11.6
Max %		90.00
[] Max		20
Approved AV		1
Interesting AV		0
Page		51 / 96

Anti-Viral
Anti-Metabolic

Export chart data to CSV

**Summary values**			
**Statistic**	**CC50**	**EC50**	**EC90**
Median	= 43.1	= 7.47	= 20
Med.Abs.Dev.	1.63		
Mean	= 43.1	= 7.47	= 20
Stdev.	2.3		

Compound	Virus			Cell		AV Method		AM Method	
Primary code	Species	Type	Strain	Type	Subtype	Method	Type	Method	Type
BAVARIE1_0052	Chikungunya virus	No Type	899	Vero	A	Absorbance	MTS - 498nm	Microscopy	Tox scoring

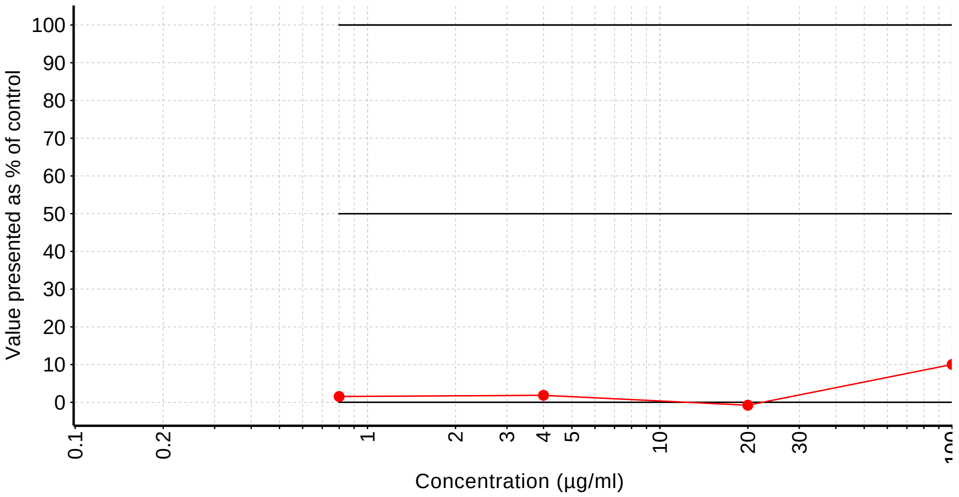

$CC50_{av}$		
EC50	>	100
EC90	>	100
SI_{av}		
SS_{av}		
TI_{av}		
Max %		10.04
[] Max		100
Approved AV		1
Interesting AV		0
Page		52 / 96

Anti-Viral
Anti-Metabolic

Export chart data to CSV

Summary values			
**Statistic**	**CC50**	**EC50**	**EC90**
Median		> 100	> 100
Med.Abs.Dev.			
Mean		> 100	> 100
Stdev.			

**Compound**	**Virus**			**Cell**		**AV Method**		**AM Method**	
Primary code	Species	Type	Strain	Type	Subtype	Method	Type	Method	Type
BAVARIE1_0053	Chikungunya virus	No Type	899	Vero	A	Absorbance	MTS - 498nm	Microscopy	Tox scoring

Needs more data.

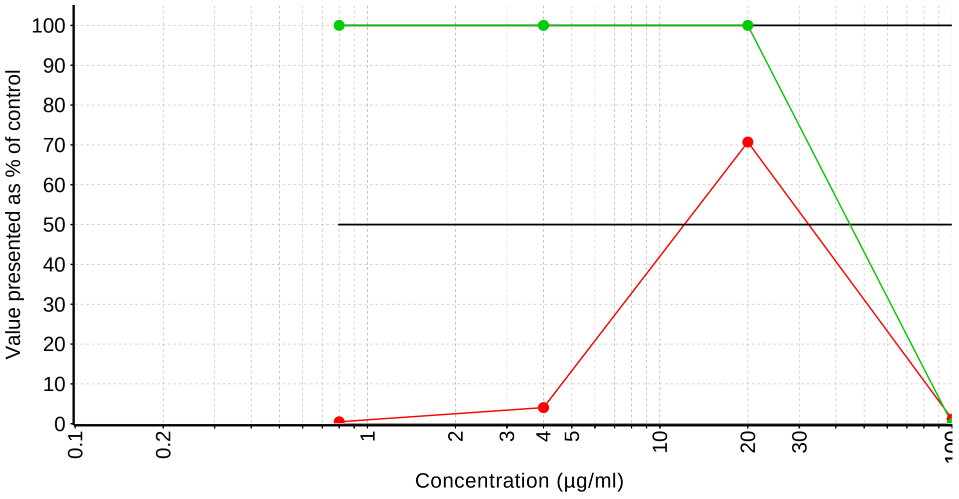

CC50 = 44.7

EC50 = 12.1

EC90

SI = 3.69

SS = 4.41

TI = 2.5

Max % 70.72

[] Max 20

Approved AV 1

Interesting AV 0

Page 53 / 96

Anti-Viral

Anti-Metabolic

Export chart data to CSV

Summary values			
**Statistic**	**CC50**	**EC50**	**EC90**
Median	= 38.5	= 12.1	
Med.Abs.Dev.	6.21		
Mean	= 38.5	= 12.1	
Stdev.	8.78		

**Compound**	**Virus**			**Cell**		**AV Method**		**AM Method**	
Primary code	Species	Type	Strain	Type	Subtype	Method	Type	Method	Type
BAVARIE1_0054	Chikungunya virus	No Type	899	Vero	A	Absorbance	MTS - 498nm	Microscopy	Tox scoring

Needs more data.

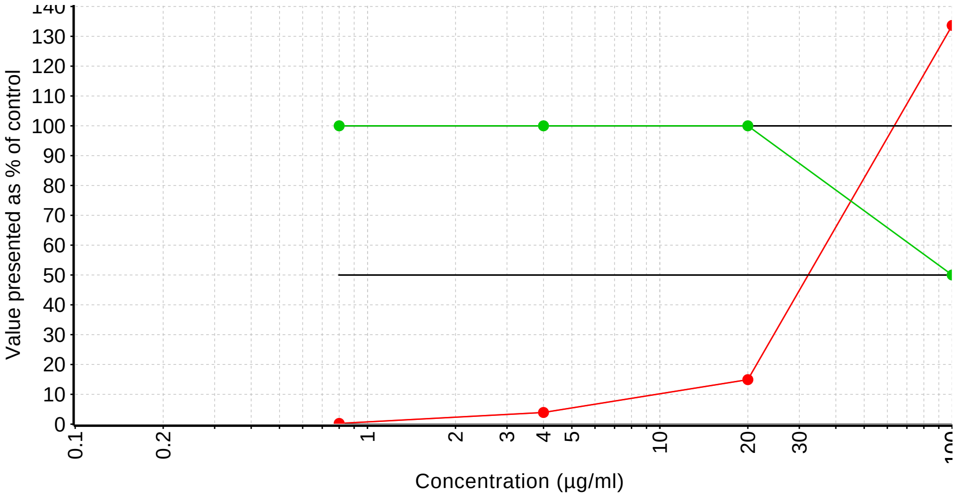

CC50	= 100
EC50	= 32.2
EC90	
SI	= 3.11
SS	= 6.1
TI	= 3.01
Max %	74.79
[] Max	60.3
Approved AV	1
Interesting AV	0
Page	54 / 96

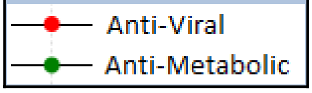

Export chart data to CSV

**Summary values**			
**Statistic**	**CC50**	**EC50**	**EC90**
Median	= 100	= 32.2	= 55.3
Med.Abs.Dev.			
Mean	= 100	= 32.2	= 55.3
Stdev.			

Compound	Virus			Cell		AV Method		AM Method	
Primary code	Species	Type	Strain	Type	Subtype	Method	Type	Method	Type
BAVARIE1_0055	Chikungunya virus	No Type	899	Vero	A	Absorbance	MTS - 498nm	Microscopy	Tox scoring

Needs more data.

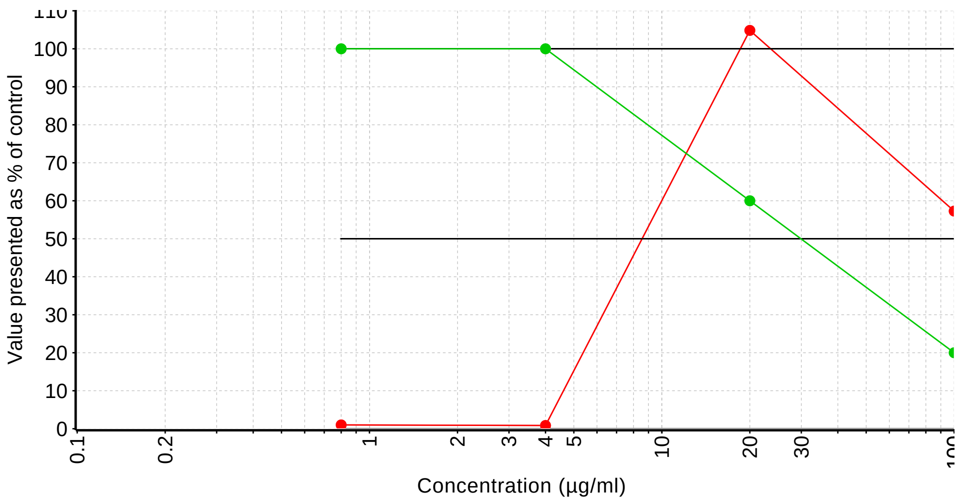

CC50	=	29.9
EC50	=	8.56
EC90		
SI	=	3.49
SS	=	5.64
TI	=	3.06
Max %		72.46
[] Max		15
Approved AV		1
Interesting AV		0
Page		55 / 96

Anti-Viral
Anti-Metabolic

Export chart data to CSV

Summary values			
**Statistic**	**CC50**	**EC50**	**EC90**
Median	= 29.9	= 8.56	= 15.9
Med.Abs.Dev.			
Mean	= 29.9	= 8.56	= 15.9
Stdev.			

**Compound**	**Virus**			**Cell**		**AV Method**		**AM Method**	
Primary code	Species	Type	Strain	Type	Subtype	Method	Type	Method	Type
BAVARIE1_0056	Chikungunya virus	No Type	899	Vero	A	Absorbance	MTS - 498nm	Microscopy	Tox scoring

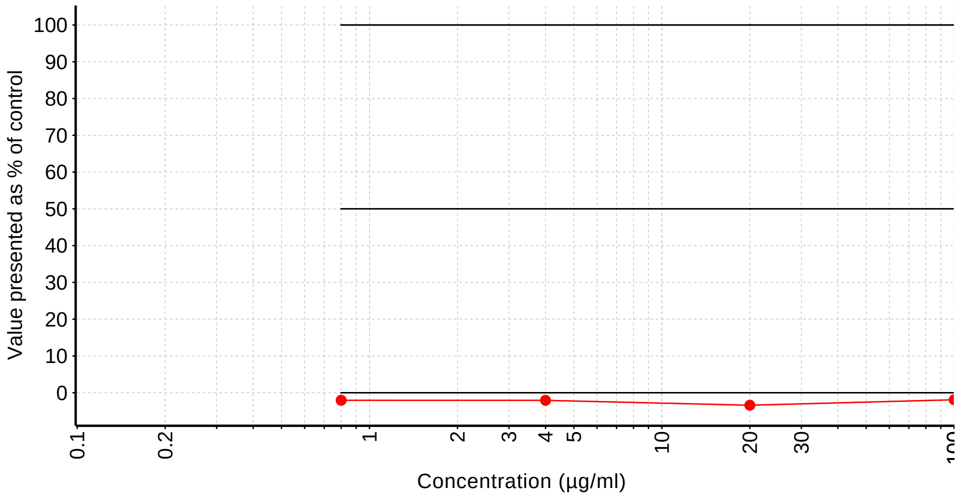

$CC50_{av}$		
EC50	>	100
EC90	>	100
SI_{av}		
SS_{av}		
TI_{av}		
Max %		-1.92
[] Max		100
Approved AV		1
Interesting AV		0
Page		56 / 96

Anti-Viral
Anti-Metabolic

Export chart data to CSV

Summary values			
**Statistic**	**CC50**	**EC50**	**EC90**
Median		> 100	> 100
Med.Abs.Dev.			
Mean		> 100	> 100
Stdev.			

**Compound**	**Virus**			**Cell**		**AV Method**		**AM Method**	
Primary code	Species	Type	Strain	Type	Subtype	Method	Type	Method	Type
BAVARIE1_0057	Chikungunya virus	No Type	899	Vero	A	Absorbance	MTS - 498nm	Microscopy	Tox scoring

Needs more data.

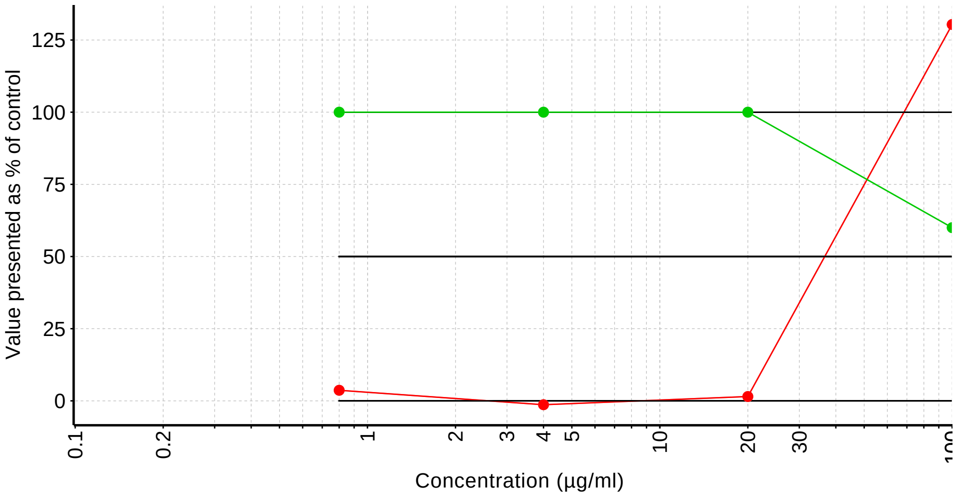

CC50	> 100
EC50	= 36.7
EC90	
SI	> 2.73
SS	> 6.69
TI	> 2.92
Max %	76.67
[] Max	66.7
Approved AV	1
Interesting AV	0
Page	57 / 96

Anti-Viral
Anti-Metabolic

Export chart data to CSV

**Summary values**			
**Statistic**	**CC50**	**EC50**	**EC90**
Median	> 100	= 36.7	= 60.4
Med.Abs.Dev.			
Mean	> 100	= 36.7	= 60.4
Stdev.			

Compound	Virus			Cell		AV Method		AM Method	
Primary code	Species	Type	Strain	Type	Subtype	Method	Type	Method	Type
BAVARIE1_0058	Chikungunya virus	No Type	899	Vero	A	Absorbance	MTS - 498nm	Microscopy	Tox scoring

Needs more data.

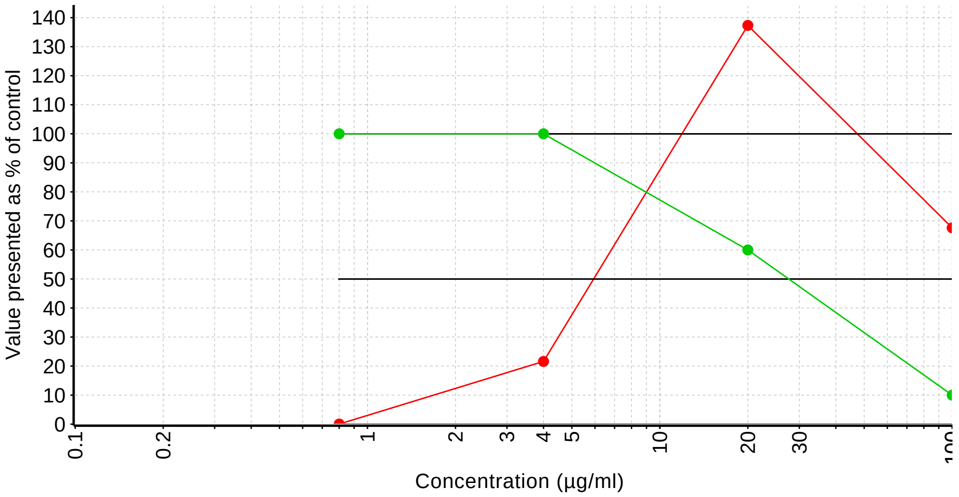

CC50 = 27.6
EC50 = 5.94
EC90
SI = 4.65
SS = 9.67
TI = 6.45
Max % 79.86
[] Max 12.1
Approved AV 1
Interesting AV 0
Page 58 / 96

Anti-Viral
Anti-Metabolic

Export chart data to CSV

Summary values			
**Statistic**	**CC50**	**EC50**	**EC90**
Median	= 27.6	= 5.94	= 10.4
Med.Abs.Dev.			
Mean	= 27.6	= 5.94	= 10.4
Stdev.			

**Compound**	**Virus**			**Cell**		**AV Method**		**AM Method**	
Primary code	Species	Type	Strain	Type	Subtype	Method	Type	Method	Type
BAVARIE1_0059	Chikungunya virus	No Type	899	Vero	A	Absorbance	MTS - 498nm	Microscopy	Tox scoring

Needs more data.

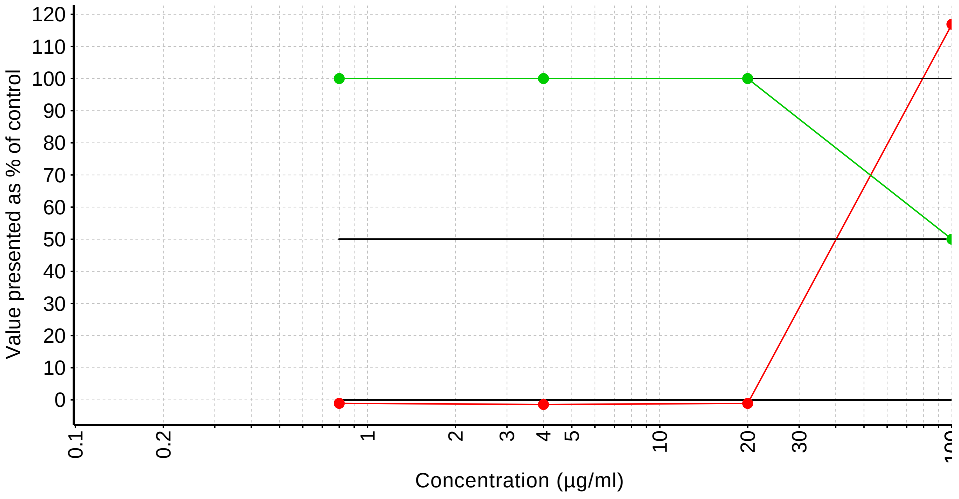

CC50 = 100

EC50 = 40.2

EC90

SI = 2.49

SS = 3.95

TI = 1.56

Max % 69.91

[] Max 68.1

Approved AV 1

Interesting AV 0

Page 59 / 96

Anti-Viral

Anti-Metabolic

Export chart data to CSV

Summary values			
**Statistic**	**CC50**	**EC50**	**EC90**
Median	= 100	= 40.2	= 69.3
Med.Abs.Dev.			
Mean	= 100	= 40.2	= 69.3
Stdev.			

Compound	Virus			Cell		AV Method		AM Method	
Primary code	Species	Type	Strain	Type	Subtype	Method	Type	Method	Type
BAVARIE1_0060	Chikungunya virus	No Type	899	Vero	A	Absorbance	MTS - 498nm	Microscopy	Tox scoring

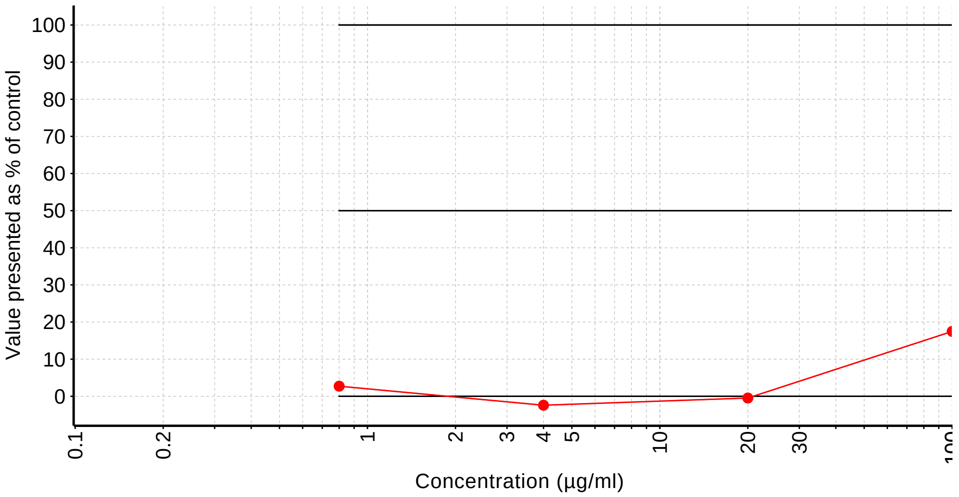

$CC50_{av}$		
EC50	>	100
EC90	>	100
SI_{av}		
SS_{av}		
TI_{av}		
Max %		17.47
[] Max		100
Approved AV		1
Interesting AV		0
Page		60 / 96

Anti-Viral
Anti-Metabolic

Export chart data to CSV

Summary values			
**Statistic**	**CC50**	**EC50**	**EC90**
Median		> 100	> 100
Med.Abs.Dev.			
Mean		> 100	> 100
Stdev.			

Repeated experiment on Chikungunya virus (select)

Compound	Virus			Cell		AV Method		AM Method	
Primary code	Species	Type	Strain	Type	Subtype	Method	Type	Method	Type
BAVARIE1_0004	Chikungunya virus	No Type	899	Vero	A	Absorbance	MTS - 498nm	Microscopy	Tox scoring

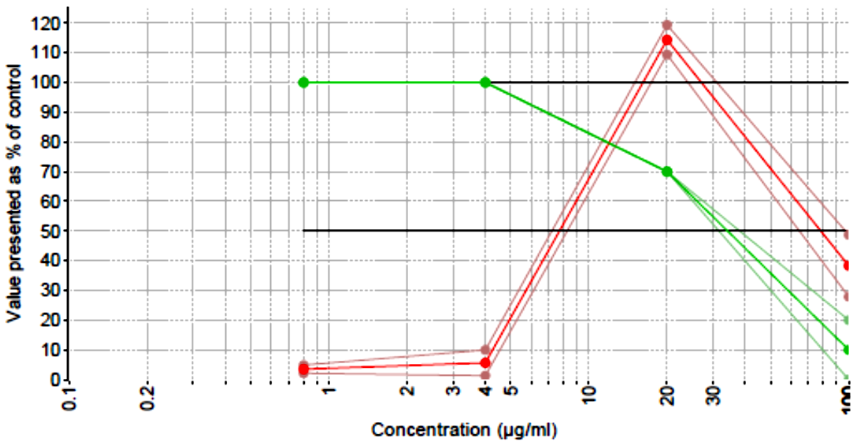

CC50	= 34.2
EC50	= 7.71
EC90	
SI	= 4.43
SS	= 9.74
TI	= 6.3
Max %	79.59
[] Max	14.9
Approved AV	2
Interesting AV	0
Page	4 / 79

Anti-Viral
Anti-Metabolic

Export chart data to CSV

Summary values			
Statistic	CC50	EC50	EC90
Median	= 51.3	= 7.73	= 14
Med.Abs.Dev.	16.5	0.525	0.999
Mean	= 57.9	= 7.73	= 14
Stdev.	29.8	0.742	1.41

Compound	Virus			Cell		AV Method		AM Method	
Primary code	Species	Type	Strain	Type	Subtype	Method	Type	Method	Type
BAVARIE1_0007	Chikungunya virus	No Type	899	Vero	A	Absorbance	MTS - 498nm	Microscopy	Tox scoring

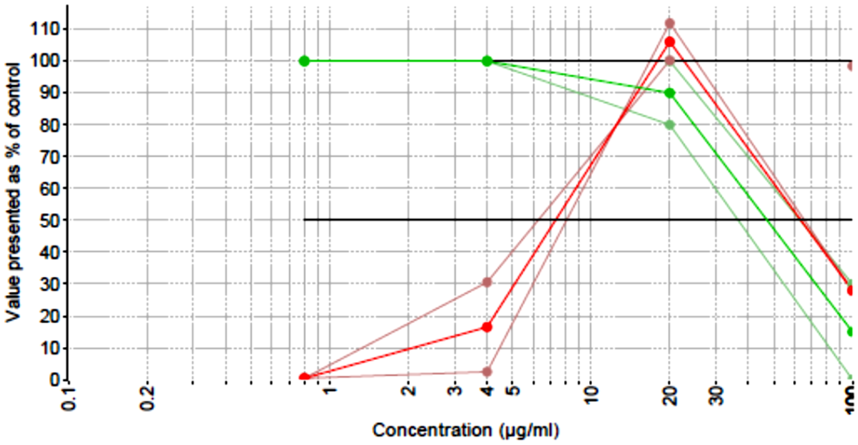

CC50 =	47.2
EC50 =	7.3
EC90 =	15
SI =	6.46
SS =	17.8
TI =	14.4
Max %	91.61
[] Max	17.4
Approved AV	2
Interesting AV	0
Page	7 / 79

Anti-Viral
Anti-Metabolic

Export chart data to CSV

Summary values			
Statistic	CC50	EC50	EC90
Median	= 63.1	= 7.16	= 15.1
Med.Abs.Dev.	2.4	0.891	0.647
Mean	= 55.1	= 7.16	= 15.1
Stdev.	16.1	1.26	0.915

Compound	Virus			Cell		AV Method		AM Method	
Primary code	Species	Type	Strain	Type	Subtype	Method	Type	Method	Type
BAVARIE1_0013	Chikungunya virus	No Type	899	Vero	A	Absorbance	MTS - 498nm	Microscopy	Tox scoring

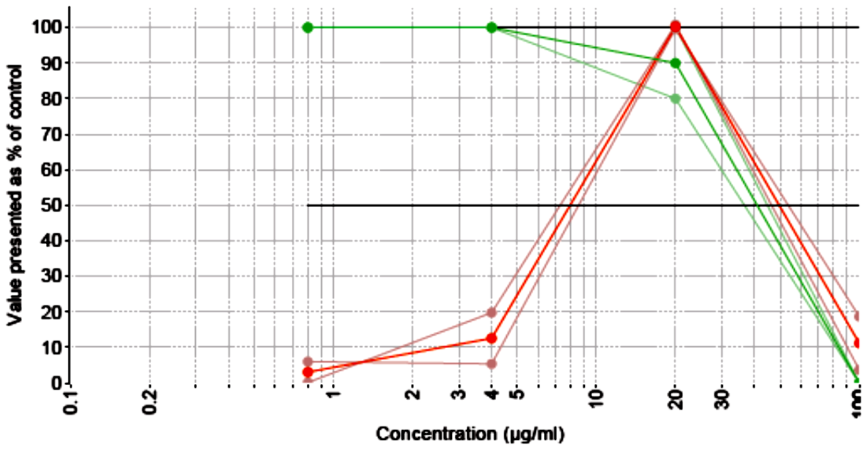

CC50 = 40.9
EC50 = 7.95
EC90 = 16.6
SI = 5.14
SS = 15.2
TI = 10.8
Max % 91.05
[] Max 18.3
Approved AV 2
Interesting AV 1
Page 13 / 79

Anti-Viral
Anti-Metabolic

Export chart data to CSV

Summary values			
Statistic	CC50	EC50	EC90
Median	= 45.6	= 7.93	= 16.6
Med.Abs.Dev.	4.48	0.638	0.409
Mean	= 45.3	= 7.93	= 16.6
Stdev.	7.02	0.902	0.579

Compound	Virus			Cell		AV Method		AM Method	
Primary code	Species	Type	Strain	Type	Subtype	Method	Type	Method	Type
BAVARIE1_0016	Chikungunya virus	No Type	899	Vero	A	Absorbance	MTS - 498nm	Microscopy	Tox scoring

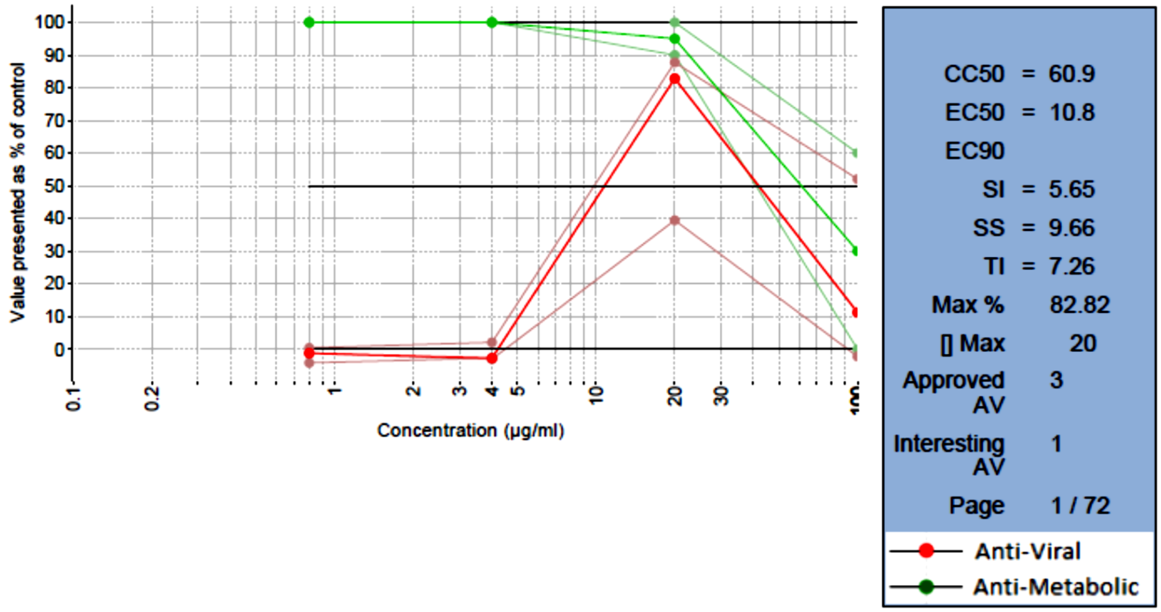

Export chart data to CSV

Summary values			
Statistic	CC50	EC50	EC90
Median	= 41.4	= 10.3	
Med.Abs.Dev.	0.466	0.472	
Mean	= 41.4	= 10.3	
Stdev.	0.659	0.667	

Compound	Virus			Cell		AV Method		AM Method	
Primary code	Species	Type	Strain	Type	Subtype	Method	Type	Method	Type
BAVARIE1_0019	Chikungunya virus	No Type	899	Vero	A	Absorbance	MTS - 498nm	Microscopy	Tox scoring

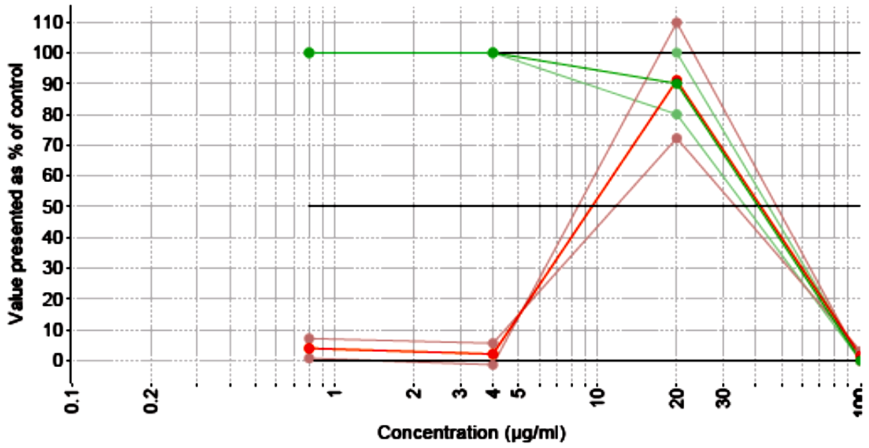

CC50	=	40.9
EC50	=	9.52
EC90	=	19.6
SI	=	4.3
SS	=	12.8
TI	=	8.08
Max %		90.10
[] Max		19.8
Approved AV		2
Interesting AV		0
Page		19 / 79

Anti-Viral
Anti-Metabolic

Export chart data to CSV

Summary values			
Statistic	CC50	EC50	EC90
Median	= 40.6	= 10.1	= 15
Med.Abs.Dev.	5.59	1.64	
Mean	= 40.7	= 10.1	= 15
Stdev.	6.77	2.31	

Compound	Virus			Cell		AV Method		AM Method	
Primary code	Species	Type	Strain	Type	Subtype	Method	Type	Method	Type
BAVARIE1_0022	Chikungunya virus	No Type	899	Vero	A	Absorbance	MTS - 498nm	Microscopy	Tox scoring

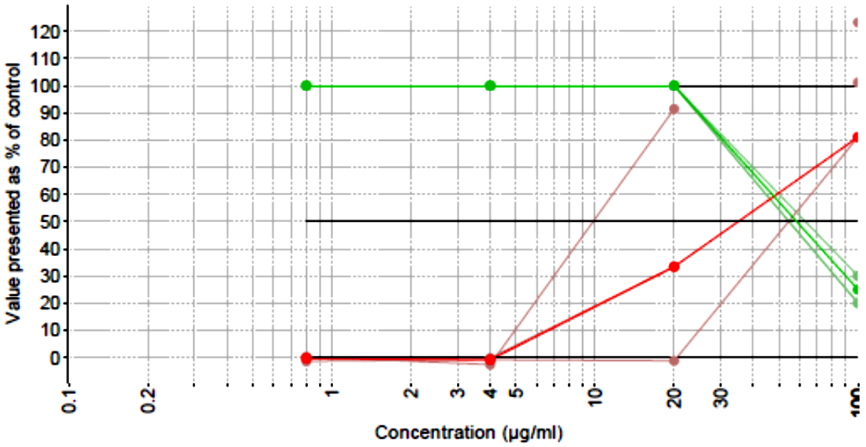

CC50	= 58.5
EC50	= 35
EC90	
SI	= 1.67
SS	= 0.164
TI	= 0.0365
Max %	59.28
[] Max	63.4
Approved AV	3
Interesting AV	1
Page	2 / 72

Anti-Viral
Anti-Metabolic

Export chart data to CSV

Summary values			
Statistic	CC50	EC50	EC90
Median	= 58.9	= 32.2	= 19.5
Med.Abs.Dev.	4.23	22.3	
Mean	= 58.9	= 32.2	= 19.5
Stdev.	4.88	31.6	

Compound	Virus			Cell		AV Method		AM Method	
Primary code	Species	Type	Strain	Type	Subtype	Method	Type	Method	Type
BAVARIE1_0025	Chikungunya virus	No Type	899	Vero	A	Absorbance	MTS - 498nm	Microscopy	Tox scoring

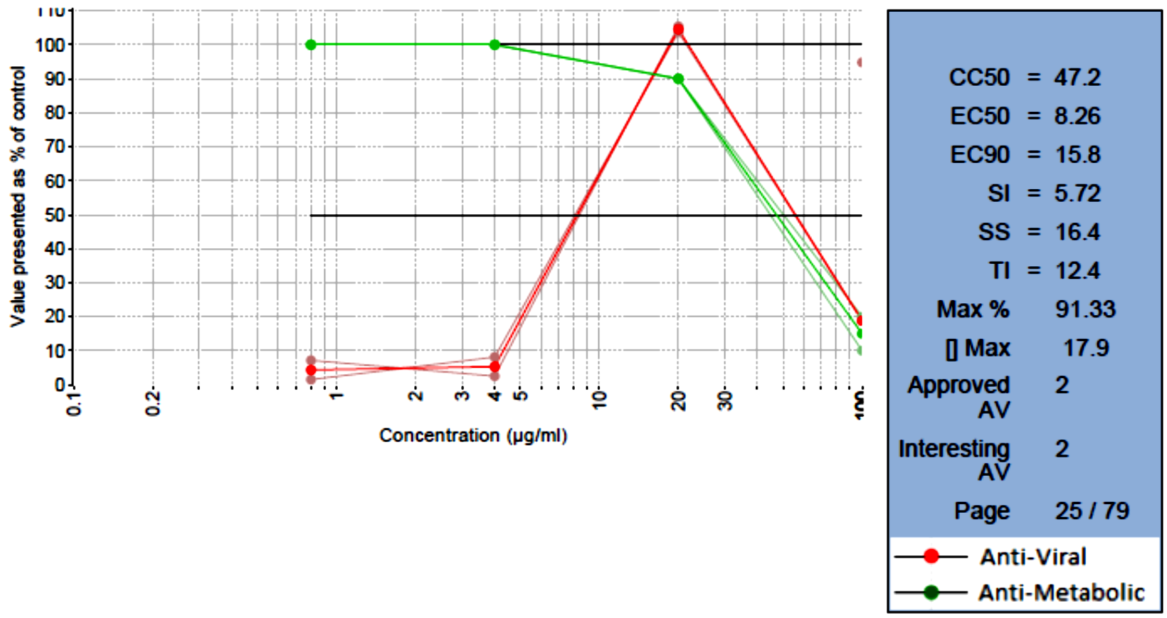

Export chart data to CSV

Summary values			
Statistic	CC50	EC50	EC90
Median	= 50.2	= 8.25	= 15.8
Med.Abs.Dev.	5.21	0.155	0.0801
Mean	= 50.1	= 8.25	= 15.8
Stdev.	5.33	0.219	0.113

Compound	Virus			Cell		AV Method		AM Method	
Primary code	Species	Type	Strain	Type	Subtype	Method	Type	Method	Type
BAVARIE1_0028	Chikungunya virus	No Type	899	Vero	A	Absorbance	MTS - 498nm	Microscopy	Tox scoring

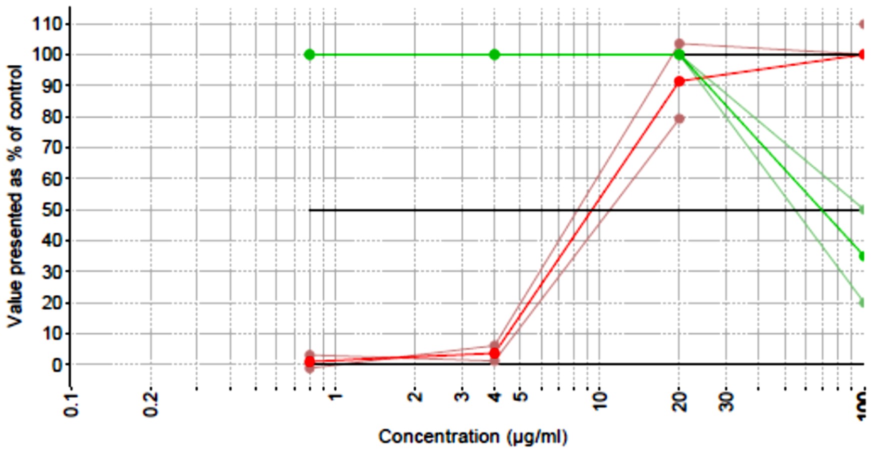

CC50	= 69
EC50	= 9.35
EC90	= 19.5
SI	= 7.38
SS	= 21.7
TI	= 18.8
Max %	92.42
[] Max	29.3
Approved AV	2
Interesting AV	1
Page	28 / 79

Anti-Viral
Anti-Metabolic

Export chart data to CSV

Summary values			
Statistic	CC50	EC50	EC90
Median	= 77.3	= 9.59	= 16
Med.Abs.Dev.	22.7	1.34	
Mean	= 77.3	= 9.59	= 16
Stdev.	32	1.89	

Compound	Virus			Cell		AV Method		AM Method	
Primary code	Species	Type	Strain	Type	Subtype	Method	Type	Method	Type
BAVARIE1_0034	Chikungunya virus	No Type	899	Vero	A	Absorbance	MTS - 498nm	Microscopy	Tox scoring

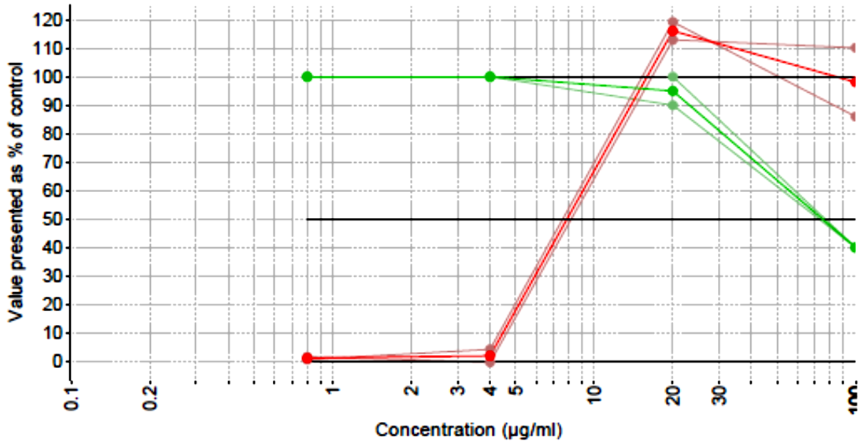

CC50	=	74.6
EC50	=	7.87
EC90	=	13.8
SI	=	9.48
SS	=	23.6
TI	=	23.1
Max %		95.89
[] Max		17.2
Approved AV		2
Interesting AV		2
Page		34 / 79

Anti-Viral
Anti-Metabolic

Export chart data to CSV

Summary values			
Statistic	CC50	EC50	EC90
Median	= 74.5	= 7.88	= 13.9
Med.Abs.Dev.	2	0.294	0.576
Mean	= 74.5	= 7.88	= 13.9
Stdev.	2.82	0.416	0.815

Compound	Virus			Cell		AV Method		AM Method	
Primary code	Species	Type	Strain	Type	Subtype	Method	Type	Method	Type
BAVARIE1_0046	Chikungunya virus	No Type	899	Vero	A	Absorbance	MTS - 498nm	Microscopy	Tox scoring

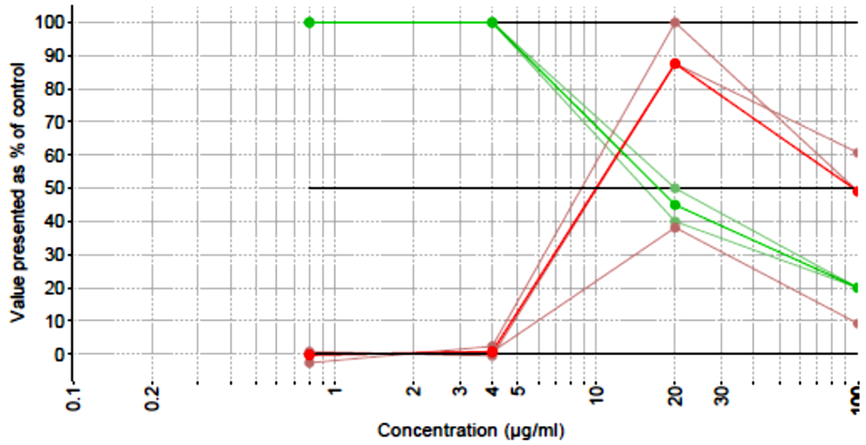

CC50	= 17.3
EC50	= 9.97
EC90	
SI	= 1.73
SS	= 1.37
TI	= 0.328
Max %	61.51
[] Max	15.2
Approved AV	3
Interesting AV	0
Page	3 / 72

Anti-Viral
Anti-Metabolic

Export chart data to CSV

Summary values			
Statistic	CC50	EC50	EC90
Median	= 20	= 9.42	= 17
Med.Abs.Dev.	4.71	0.647	
Mean	= 44.1	= 9.42	= 17
Stdev.	45.9	0.915	

Compound	Virus			Cell		AV Method		AM Method	
Primary code	Species	Type	Strain	Type	Subtype	Method	Type	Method	Type
BAVARIE1_0048	Chikungunya virus	No Type	899	Vero	A	Absorbance	MTS - 498nm	Microscopy	Tox scoring

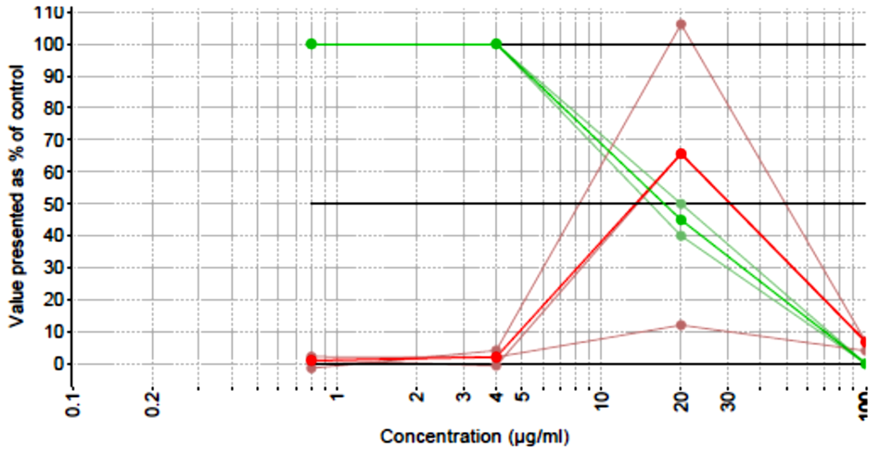

CC50	=	17.3
EC50	=	13.5
EC90		
SI	=	1.28
SS	=	0.249
TI	=	0.0271
Max %		54.59
[] Max		17.2
Approved AV		3
Interesting AV		0
Page		4 / 72

Anti-Viral
Anti-Metabolic

Export chart data to CSV

Summary values			
Statistic	CC50	EC50	EC90
Median	= 25.4	= 11	= 15.5
Med.Abs.Dev.	7.72	2.71	
Mean	= 28.9	= 11	= 15.5
Stdev.	15.3	3.83	

Compound	Virus			Cell		AV Method		AM Method	
Primary code	Species	Type	Strain	Type	Subtype	Method	Type	Method	Type
BAVARIE1_0049	Chikungunya virus	No Type	899	Vero	A	Absorbance	MTS - 498nm	Microscopy	Tox scoring

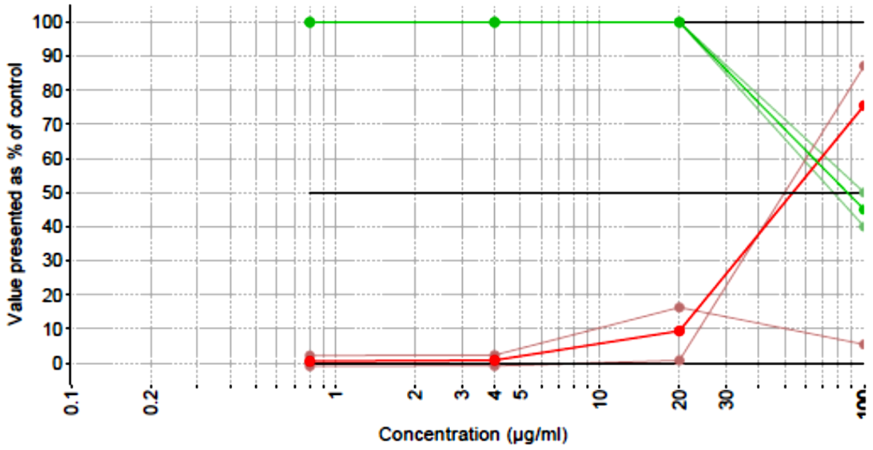

CC50	= 86.4
EC50	= 53.7
EC90	
SI	= 1.61
SS	= 0.916
TI	= 0.189
Max %	58.87
[] Max	79.8
Approved AV	3
Interesting AV	0
Page	5 / 72

Anti-Viral
Anti-Metabolic

Export chart data to CSV

Summary values			
Statistic	CC50	EC50	EC90
Median	= 88.2	= 51.9	> 100
Med.Abs.Dev.	11.8	1.84	
Mean	= 88.2	= 51.9	> 100
Stdev.	16.6	2.6	

Compound	Virus			Cell		AV Method		AM Method	
Primary code	Species	Type	Strain	Type	Subtype	Method	Type	Method	Type
BAVARIE1_0058	Chikungunya virus	No Type	899	Vero	A	Absorbance	MTS - 498nm	Microscopy	Tox scoring

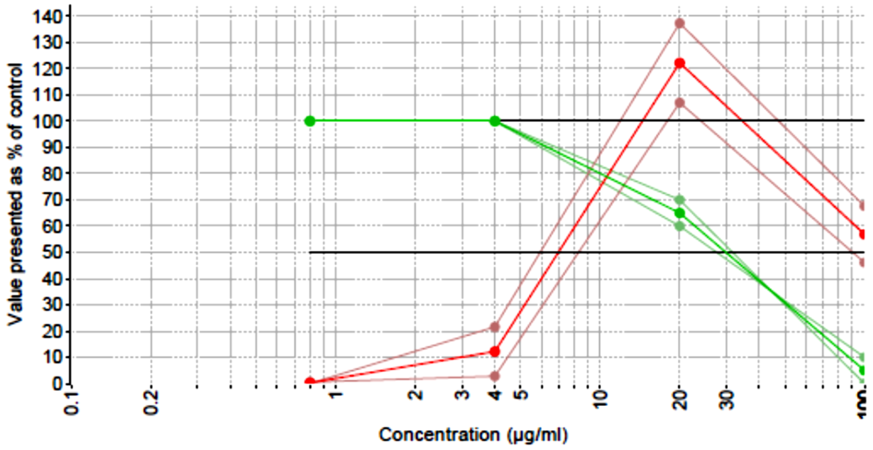

CC50	= 29.9
EC50	= 6.95
EC90	
SI	= 4.3
SS	= 9.15
TI	= 5.8
Max %	78.80
[] Max	13.7
Approved AV	2
Interesting AV	0
Page	58 / 79

Anti-Viral
Anti-Metabolic

Export chart data to CSV

Summary values			
Statistic	CC50	EC50	EC90
Median	= 31.7	= 7.11	= 12.9
Med.Abs.Dev.	4.08	1.18	2.51
Mean	= 49.8	= 7.11	= 12.9
Stdev.	35	1.66	3.55

Compound	Virus			Cell		AV Method		AM Method	
Primary code	Species	Type	Strain	Type	Subtype	Method	Type	Method	Type
BAVARIE1_0100	Chikungunya virus	No Type	899	Vero	A	Absorbance	MTS - 498nm	Microscopy	Tox scoring

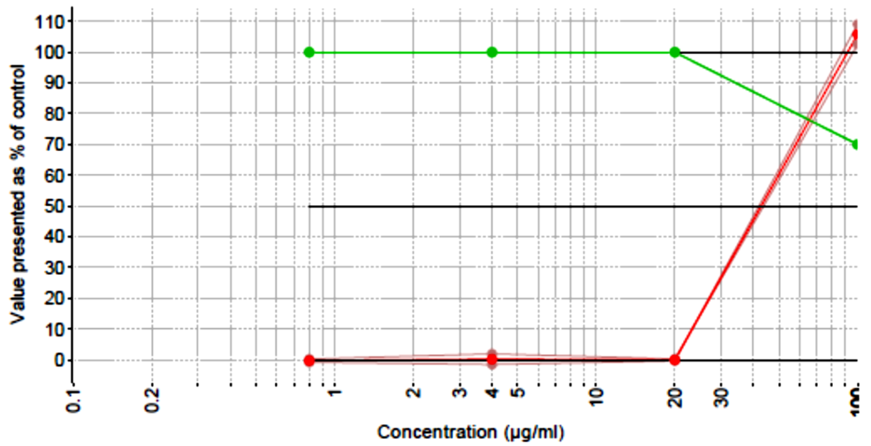

CC50	>	100
EC50	=	42.8
EC90		
SI	>	2.34
SS	>	6.17
TI	>	2.28
Max %		77.91
[] Max		78.9
Approved AV		2
Interesting AV		0
Page		1 / 49

Anti-Viral
Anti-Metabolic

Export chart data to CSV

Summary values			
Statistic	CC50	EC50	EC90
Median	> 100	= 42.8	= 78.8
Med.Abs.Dev.		0.9	3.28
Mean	> 100	= 42.8	= 78.8
Stdev.		1.27	4.64
