# Supplementary material for: Antiviral and Cytotoxic Activity of Different Plant Parts of Banana (Musa spp.)
Source: Viruses. 2020 May 15;12(5):549. doi: 10.3390/v12050549 (PMC7291111; doi:10.3390/v12050549)
Supplement: Supplementary file 1 [file viruses-12-00549-s001.zip › Supplementary Files~/Supplementary material III-Yellow fever-SP.pdf]

Antiviral and Cytotoxic Activity of Different Plant Parts of Banana (*Musa* spp.)

Sujogya Kumar Panda [1,2,*], Ana Hortência Fonsêca Castro [1,3], Ramin Saleh Jouneghani [1], Pieter Leyssen [4], Johan Neyts [4], Rony Swennen [5,6,7] and Walter Luyten [1]

[1] Department of Biology, Katholieke Universiteit Leuven, 3000 Leuven, Belgium; acastro905@gmail.com (A.H.F.C.); r.saleh.j@gmail.com (R.S.J.); walter.luyten@kuleuven.be (W.L.)
[2] Mayurbhanj Biological Research (MBR), Bhanjpur, Baripada 757002, Odisha, India
[3] Plant Physiology and Biochemistry, Universidade Federal de São João Del-Rei, Av. Sebastião Gonçalves Coelho, 400—Chanandour, Divinópolis MG 35501-296, Brazil
[4] Rega Institute for Medical Research, Laboratory of Virology and Chemotherapy, Katholieke Universiteit Leuven, 3000 Leuven, Belgium; pieter.leyssen@kuleuven.be (P.L.); johan.neyts@kuleuven.be (J.N.)
[5] International Institute of Tropical Agriculture, Arusha P.O. Box 447, Tanzania; rony.swennen@kuleuven.be
[6] Laboratory of Tropical Crop Improvement, Division of Crop Biotechnics, Katholieke Universiteit Leuven, 3001 Leuven, Belgium
[7] Bioversity International, 3001 Leuven, Belgium
* Correspondence: sujogya.panda@kuleuven.be; Tel.: +32-16-373467

Supplementary material III

Figure S3: Antiviral activity of extracts against yellow fever virus. Extracts (primary code) tested mentioned under the header compounds in each panel. EC_{50} = 50% Effective Concentration (concentration at which 50% inhibition of virus replication is observed); EC_{90} = 90% Effective Concentration (concentration at which 90% inhibition of virus replication is observed); CC_{50} = 50% Cytostatic/Cytotoxic Concentration (concentration at which 50% adverse effect is observed on Huh cells in parallel with antiviral assay); SI = Selectivity Index (CC_{50}/EC_{50}); SS = Selectivity Surface (integrated surface delineated by the EC_{50} curve, the CC_{50} curve and the 50% horizontal); TI = Therapeutic Index (SS × 10logSI); AV-Antiviral; AM-Antimetabolic.

Compound	Virus			Cell		AV Method		AM Method	
Primary code	Species	Type	Strain	Type	Subtype	Method	Type	Method	Type
BAVARIE1_0001	Yellow fever virus	No Type	17D Stamaril	Huh	7	Absorbance	MTS - 498nm	Microscopy	Tox scoring

Needs more data.

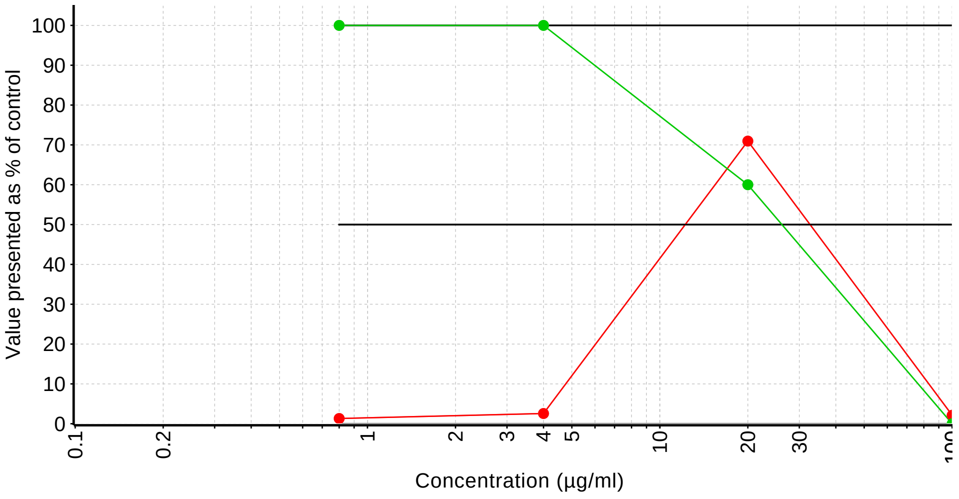

CC50 = 26.2
EC50 = 12.2
EC90
SI = 2.14
SS = 2.27
TI = 0.75
Max % 64.04
[] Max 18.4
Approved AV 1
Interesting AV 0
Page 1 / 96

Anti-Viral
Anti-Metabolic

Export chart data to CSV

Summary values			
**Statistic**	**CC50**	**EC50**	**EC90**
Median	= 29.4	= 12.2	
Med.Abs.Dev.	3.25		
Mean	= 29.4	= 12.2	
Stdev.	4.59		

Compound	Virus			Cell		AV Method		AM Method	
Primary code	Species	Type	Strain	Type	Subtype	Method	Type	Method	Type
BAVARIE1_0002	Yellow fever virus	No Type	17D Stamaril	Huh	7	Absorbance	MTS - 498nm	Microscopy	Tox scoring

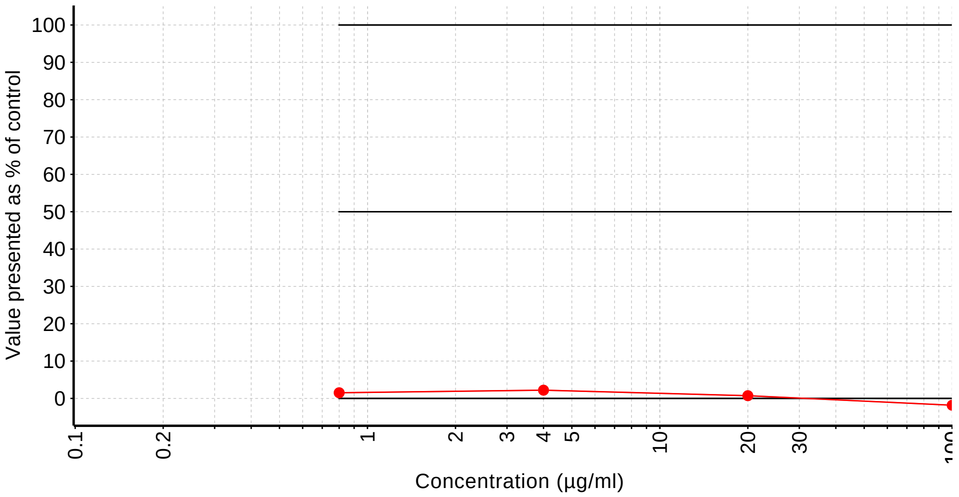

$CC50_{av}$		
EC50	>	100
EC90	>	100
SI_{av}		
SS_{av}		
TI_{av}		
Max %		2.21
[] Max		4
Approved AV		1
Interesting AV		0
Page		2 / 96

Anti-Viral
Anti-Metabolic

Export chart data to CSV

Summary values			
**Statistic**	**CC50**	**EC50**	**EC90**
Median		> 100	> 100
Med.Abs.Dev.			
Mean		> 100	> 100
Stdev.			

Compound	Virus			Cell		AV Method		AM Method	
Primary code	Species	Type	Strain	Type	Subtype	Method	Type	Method	Type
BAVARIE1_0003	Yellow fever virus	No Type	17D Stamaril	Huh	7	Absorbance	MTS - 498nm	Microscopy	Tox scoring

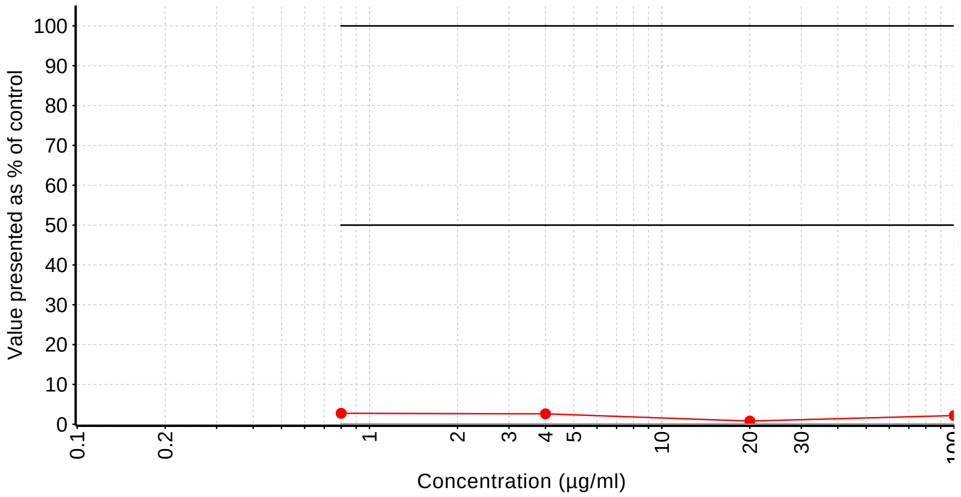

CC50av		
EC50	>	100
EC90	>	100
SIav		
SSav		
TIav		
Max %		2.74
[] Max		0.8
Approved AV		1
Interesting AV		0
Page		3 / 96

Anti-Viral
Anti-Metabolic

Export chart data to CSV

Summary values			
**Statistic**	**CC50**	**EC50**	**EC90**
Median		> 100	> 100
Med.Abs.Dev.			
Mean		> 100	> 100
Stdev.			

Compound	Virus			Cell		AV Method		AM Method	
Primary code	Species	Type	Strain	Type	Subtype	Method	Type	Method	Type
BAVARIE1_0004	Yellow fever virus	No Type	17D Stamaril	Huh	7	Absorbance	MTS - 498nm	Microscopy	Tox scoring

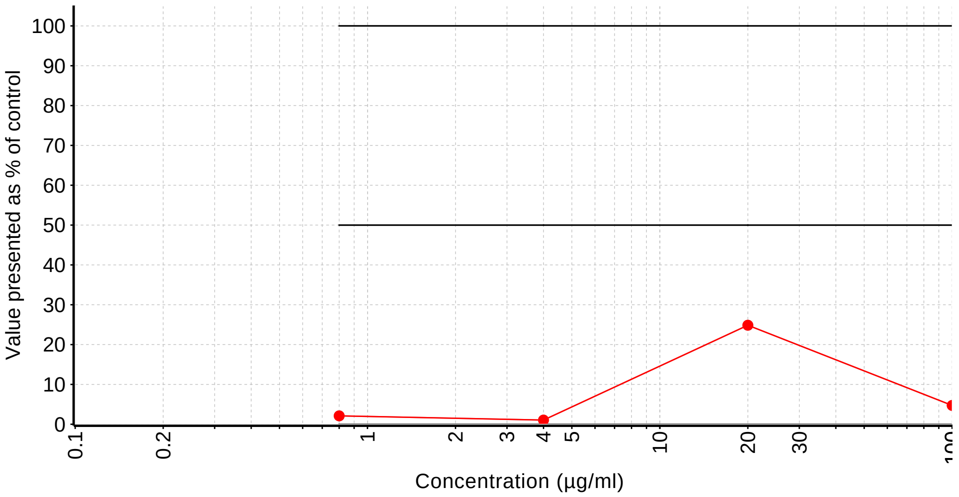

$CC50_{av}$	
EC50	
EC90	
SI_{av}	
SS_{av}	
TI_{av}	
Max %	24.85
[] Max	20
Approved AV	1
Interesting AV	0
Page	4 / 96

Anti-Viral
Anti-Metabolic

Export chart data to CSV

Summary values			
**Statistic**	**CC50**	**EC50**	**EC90**
Median			
Med.Abs.Dev.			
Mean			
Stdev.			

Compound	Virus			Cell		AV Method		AM Method	
Primary code	Species	Type	Strain	Type	Subtype	Method	Type	Method	Type
BAVARIE1_0005	Yellow fever virus	No Type	17D Stamaril	Huh	7	Absorbance	MTS - 498nm	Microscopy	Tox scoring

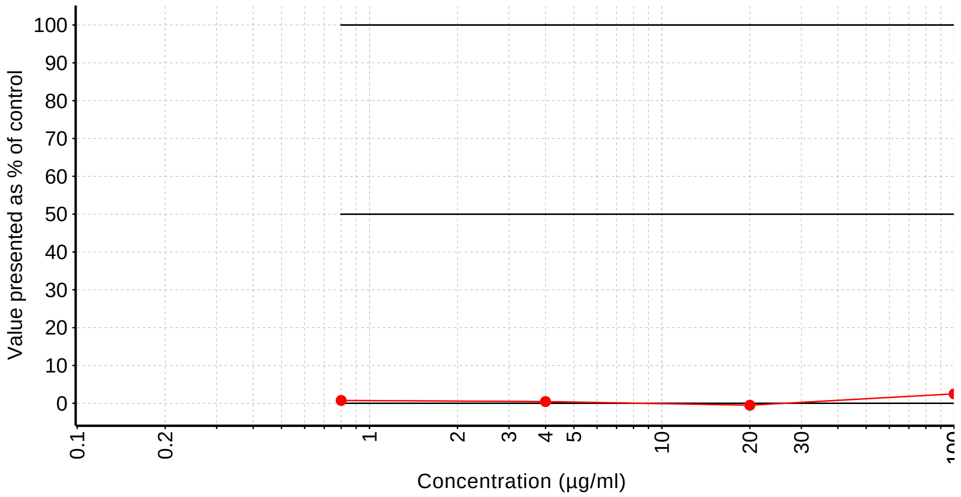

$CC50_{av}$		
EC50	>	100
EC90	>	100
SI_{av}		
SS_{av}		
TI_{av}		
Max %		2.50
[] Max		100
Approved AV		1
Interesting AV		0
Page		5 / 96

Anti-Viral
Anti-Metabolic

Export chart data to CSV

Summary values			
**Statistic**	**CC50**	**EC50**	**EC90**
Median		> 100	> 100
Med.Abs.Dev.			
Mean		> 100	> 100
Stdev.			

Compound	Virus			Cell		AV Method		AM Method	
Primary code	Species	Type	Strain	Type	Subtype	Method	Type	Method	Type
BAVARIE1_0006	Yellow fever virus	No Type	17D Stamaril	Huh	7	Absorbance	MTS - 498nm	Microscopy	Tox scoring

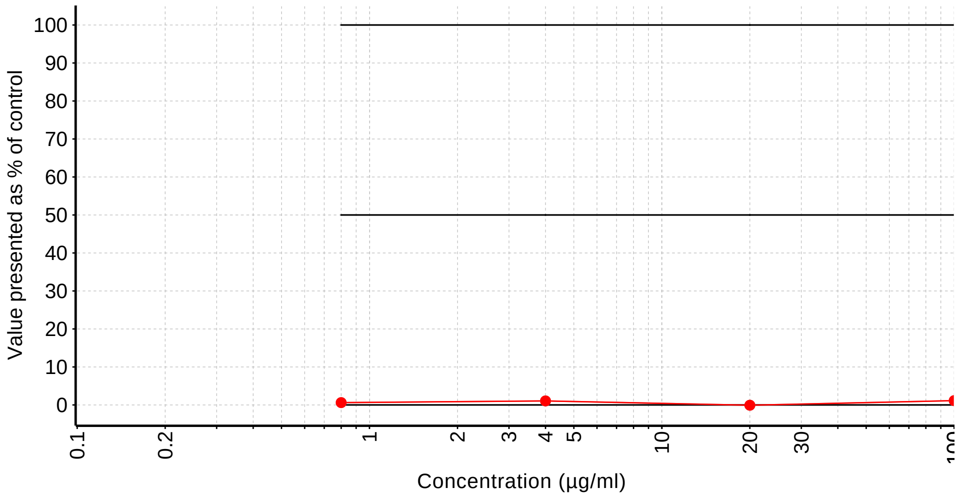

$CC50_{av}$		
EC50	>	100
EC90	>	100
SI_{av}		
SS_{av}		
TI_{av}		
Max %		1.14
[] Max		100
Approved AV		1
Interesting AV		0
Page		6 / 96

Anti-Viral
Anti-Metabolic

Export chart data to CSV

Summary values			
**Statistic**	**CC50**	**EC50**	**EC90**
Median		> 100	> 100
Med.Abs.Dev.			
Mean		> 100	> 100
Stdev.			

Compound	Virus			Cell		AV Method		AM Method	
Primary code	Species	Type	Strain	Type	Subtype	Method	Type	Method	Type
BAVARIE1_0007	Yellow fever virus	No Type	17D Stamaril	Huh	7	Absorbance	MTS - 498nm	Microscopy	Tox scoring

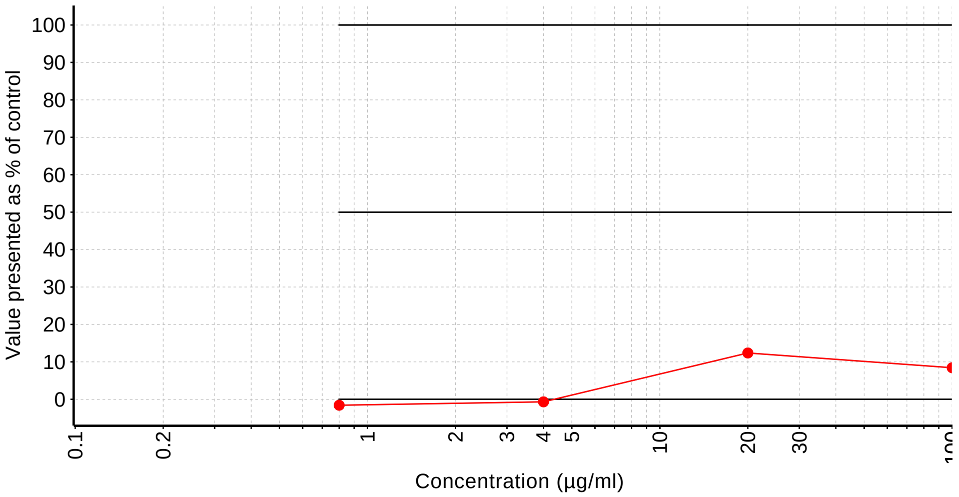

CC50$_{av}$		
EC50	>	100
EC90	>	100
SI$_{av}$		
SS$_{av}$		
TI$_{av}$		
Max %		12.37
[] Max		20
Approved AV		1
Interesting AV		0
Page		7 / 96

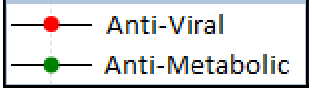

Export chart data to CSV

Summary values			
**Statistic**	**CC50**	**EC50**	**EC90**
Median		> 100	> 100
Med.Abs.Dev.			
Mean		> 100	> 100
Stdev.			

Compound	Virus			Cell		AV Method		AM Method	
Primary code	Species	Type	Strain	Type	Subtype	Method	Type	Method	Type
BAVARIE1_0008	Yellow fever virus	No Type	17D Stamaril	Huh	7	Absorbance	MTS - 498nm	Microscopy	Tox scoring

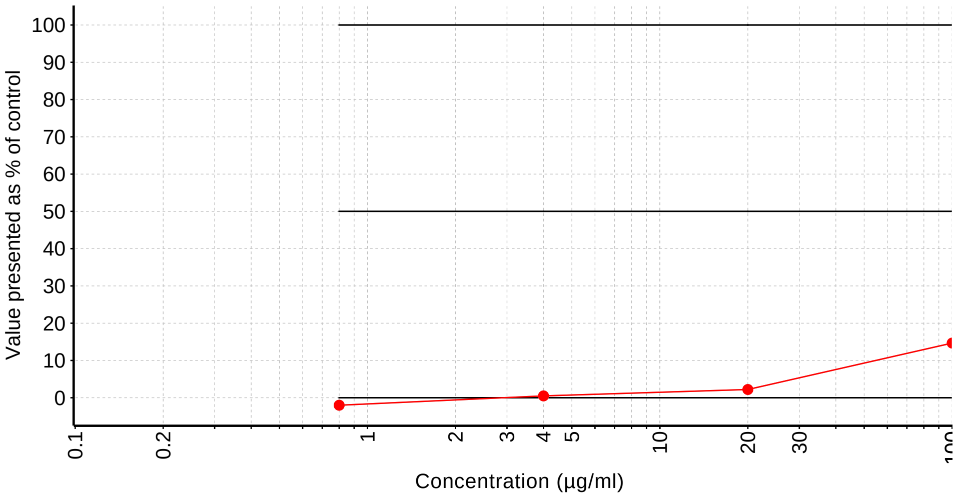

$CC50_{av}$		
EC50	>	100
EC90	>	100
SI_{av}		
SS_{av}		
TI_{av}		
Max %		14.67
[] Max		100
Approved AV		1
Interesting AV		0
Page		8 / 96

Anti-Viral
Anti-Metabolic

Export chart data to CSV

Summary values			
**Statistic**	**CC50**	**EC50**	**EC90**
Median		> 100	> 100
Med.Abs.Dev.			
Mean		> 100	> 100
Stdev.			

Compound	Virus			Cell		AV Method		AM Method	
Primary code	Species	Type	Strain	Type	Subtype	Method	Type	Method	Type
BAVARIE1_0009	Yellow fever virus	No Type	17D Stamaril	Huh	7	Absorbance	MTS - 498nm	Microscopy	Tox scoring

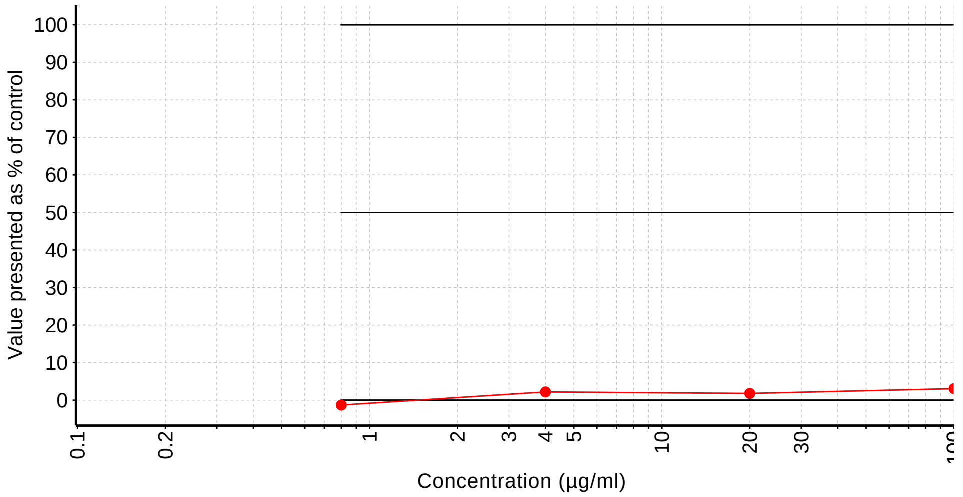

$CC50_{av}$		
EC50	>	100
EC90	>	100
SI_{av}		
SS_{av}		
TI_{av}		
Max %		3.07
[] Max		100
Approved AV		1
Interesting AV		0
Page		9 / 96

Anti-Viral
Anti-Metabolic

Export chart data to CSV

Summary values			
**Statistic**	**CC50**	**EC50**	**EC90**
Median		> 100	> 100
Med.Abs.Dev.			
Mean		> 100	> 100
Stdev.			

Compound	Virus			Cell		AV Method		AM Method	
Primary code	Species	Type	Strain	Type	Subtype	Method	Type	Method	Type
BAVARIE1_0010	Yellow fever virus	No Type	17D Stamaril	Huh	7	Absorbance	MTS - 498nm	Microscopy	Tox scoring

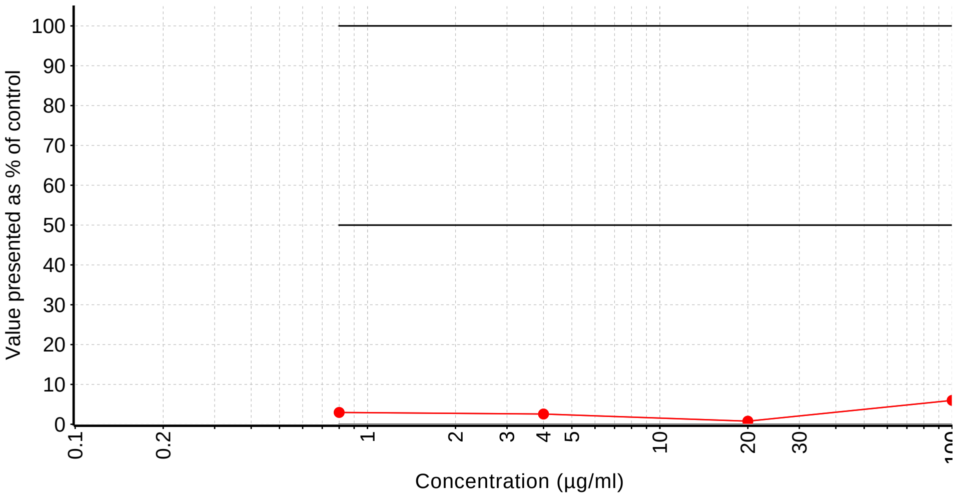

$CC50_{av}$		
EC50	>	100
EC90	>	100
SI_{av}		
SS_{av}		
TI_{av}		
Max %		5.99
[] Max		100
Approved AV		1
Interesting AV		0
Page		10 / 96

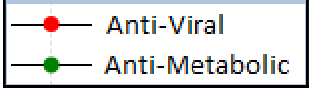

Export chart data to CSV

Summary values			
**Statistic**	**CC50**	**EC50**	**EC90**
Median		> 100	> 100
Med.Abs.Dev.			
Mean		> 100	> 100
Stdev.			

**Compound**	**Virus**			**Cell**		**AV Method**		**AM Method**	
Primary code	Species	Type	Strain	Type	Subtype	Method	Type	Method	Type
BAVARIE1_0011	Yellow fever virus	No Type	17D Stamaril	Huh	7	Absorbance	MTS - 498nm	Microscopy	Tox scoring

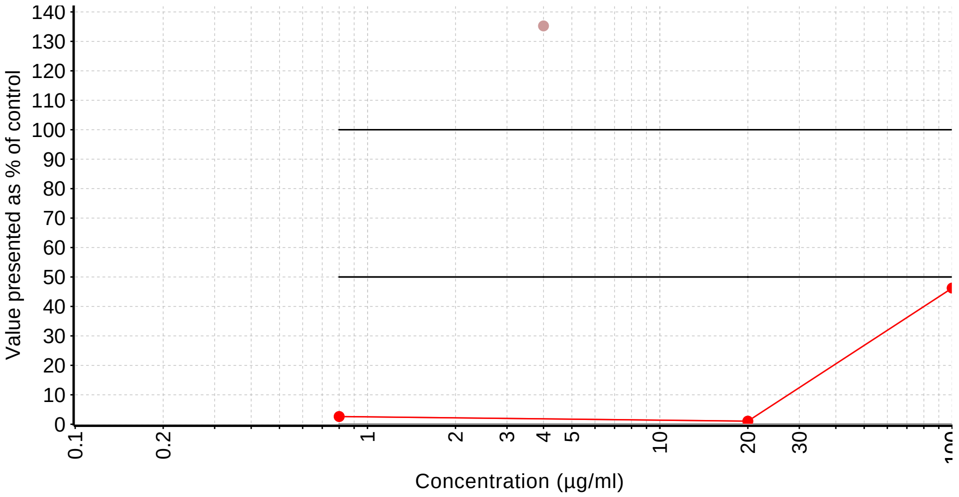

$CC50_{av}$		
EC50	>	100
EC90	>	100
SI_{av}		
SS_{av}		
TI_{av}		
Max %		46.24
[] Max		100
Approved AV		1
Interesting AV		0
Page		11 / 96

Anti-Viral
Anti-Metabolic

Export chart data to CSV

**Summary values**			
**Statistic**	**CC50**	**EC50**	**EC90**
Median		> 100	> 100
Med.Abs.Dev.			
Mean		> 100	> 100
Stdev.			

Compound	Virus			Cell		AV Method		AM Method	
Primary code	Species	Type	Strain	Type	Subtype	Method	Type	Method	Type
BAVARIE1_0012	Yellow fever virus	No Type	17D Stamaril	Huh	7	Absorbance	MTS - 498nm	Microscopy	Tox scoring

Needs more data.

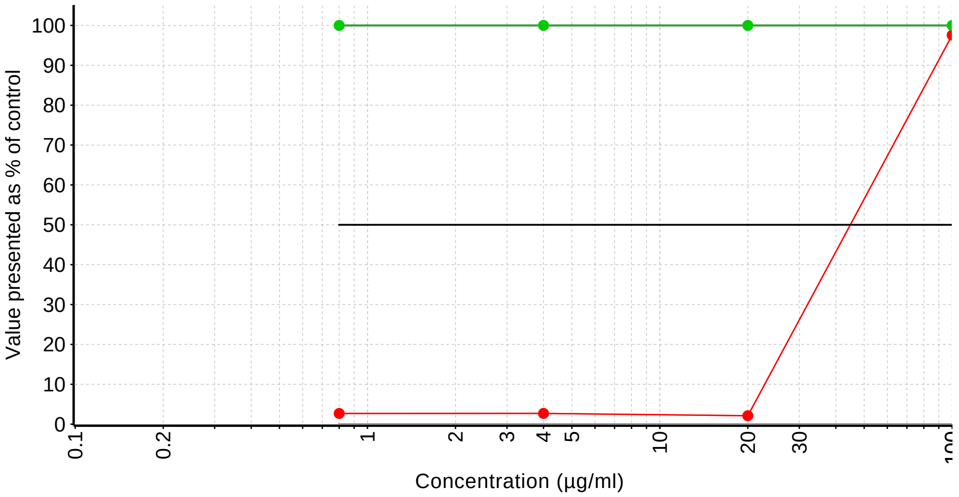

CC50	>	100
EC50	=	44.8
EC90	=	88
SI	>	2.23
SS	>	8.29
TI	>	2.89
Max %		97.57
[] Max		100
Approved AV		1
Interesting AV		0
Page		12 / 96

Anti-Viral
Anti-Metabolic

Export chart data to CSV

Summary values			
**Statistic**	**CC50**	**EC50**	**EC90**
Median	> 100	= 44.8	= 88
Med.Abs.Dev.			
Mean	> 100	= 44.8	= 88
Stdev.			

**Compound**	**Virus**			**Cell**		**AV Method**		**AM Method**	
Primary code	Species	Type	Strain	Type	Subtype	Method	Type	Method	Type
BAVARIE1_0013	Yellow fever virus	No Type	17D Stamaril	Huh	7	Absorbance	MTS - 498nm	Microscopy	Tox scoring

Needs more data.

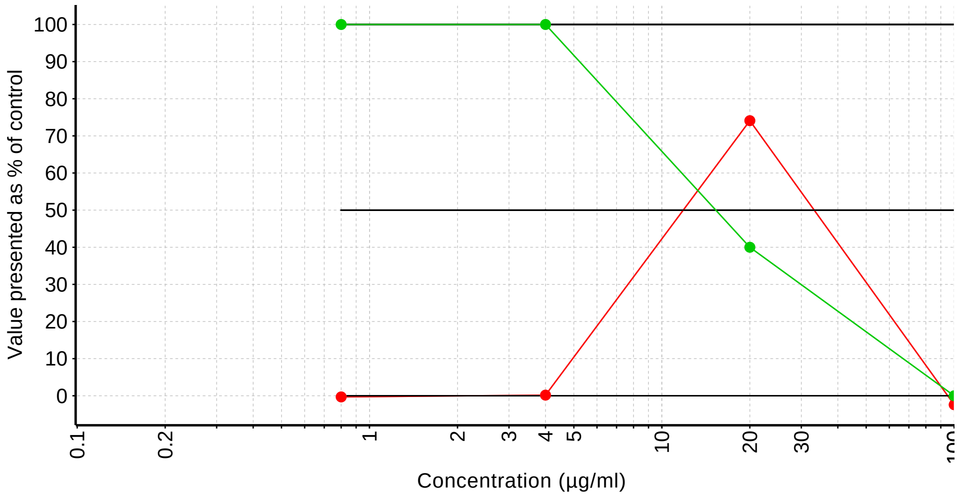

CC50	= 15.3
EC50	= 11.8
EC90	
SI	= 1.29
SS	= 0.888
TI	= 0.0988
Max %	55.27
[] Max	15.9
Approved AV	1
Interesting AV	0
Page	13 / 96

Anti-Viral
Anti-Metabolic

Export chart data to CSV

Summary values			
**Statistic**	**CC50**	**EC50**	**EC90**
Median	= 24.2	= 11.8	
Med.Abs.Dev.	8.95		
Mean	= 24.2	= 11.8	
Stdev.	12.7		

**Compound**	**Virus**			**Cell**		**AV Method**		**AM Method**	
Primary code	Species	Type	Strain	Type	Subtype	Method	Type	Method	Type
BAVARIE1_0014	Yellow fever virus	No Type	17D Stamaril	Huh	7	Absorbance	MTS - 498nm	Microscopy	Tox scoring

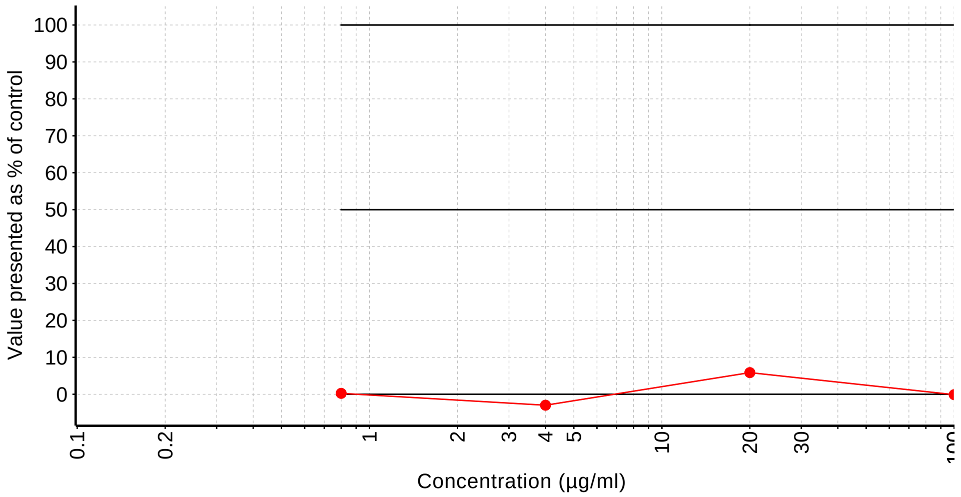

$CC50_{av}$		
EC50	>	100
EC90	>	100
SI_{av}		
SS_{av}		
TI_{av}		
Max %		5.86
[] Max		20
Approved AV		1
Interesting AV		0
Page		14 / 96

Anti-Viral
Anti-Metabolic

Export chart data to CSV

Summary values			
**Statistic**	**CC50**	**EC50**	**EC90**
Median		> 100	> 100
Med.Abs.Dev.			
Mean		> 100	> 100
Stdev.			

**Compound**	**Virus**			**Cell**		**AV Method**		**AM Method**	
Primary code	Species	Type	Strain	Type	Subtype	Method	Type	Method	Type
BAVARIE1_0015	Yellow fever virus	No Type	17D Stamaril	Huh	7	Absorbance	MTS - 498nm	Microscopy	Tox scoring

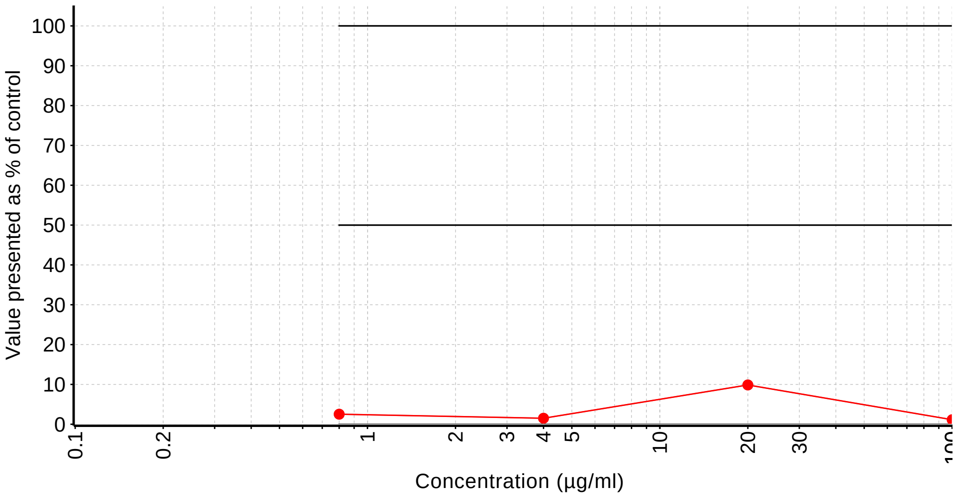

$CC50_{av}$		
EC50	>	100
EC90	>	100
SI_{av}		
SS_{av}		
TI_{av}		
Max %		9.86
[] Max		20
Approved AV		1
Interesting AV		0
Page		15 / 96

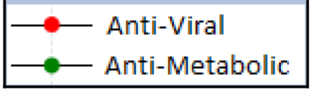

Export chart data to CSV

Summary values			
**Statistic**	**CC50**	**EC50**	**EC90**
Median		> 100	> 100
Med.Abs.Dev.			
Mean		> 100	> 100
Stdev.			

Compound	Virus			Cell		AV Method		AM Method	
Primary code	Species	Type	Strain	Type	Subtype	Method	Type	Method	Type
BAVARIE1_0016	Yellow fever virus	No Type	17D Stamaril	Huh	7	Absorbance	MTS - 498nm	Microscopy	Tox scoring

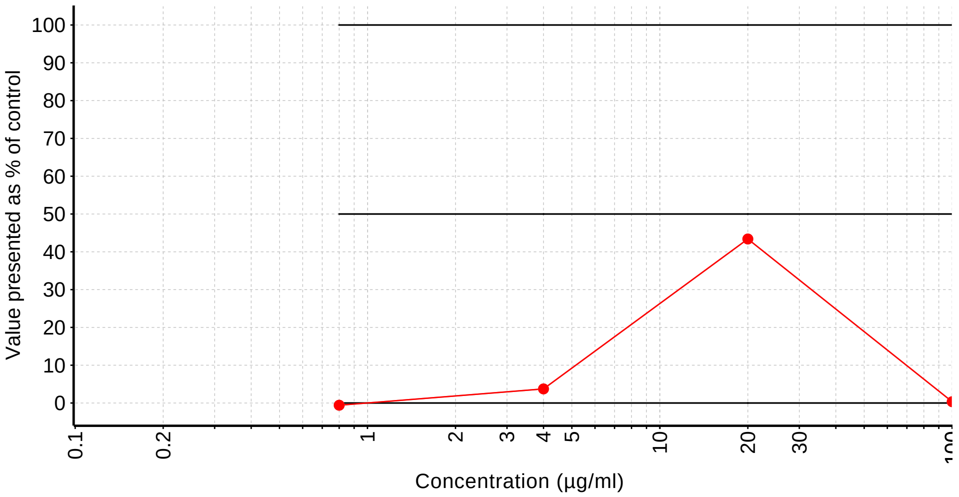

$CC50_{av}$	
EC50	
EC90	
SI_{av}	
SS_{av}	
TI_{av}	
Max %	43.39
[] Max	20
Approved AV	1
Interesting AV	0
Page	16 / 96

Anti-Viral
Anti-Metabolic

Export chart data to CSV

Summary values			
**Statistic**	**CC50**	**EC50**	**EC90**
Median			
Med.Abs.Dev.			
Mean			
Stdev.			

**Compound**	**Virus**			**Cell**		**AV Method**		**AM Method**	
Primary code	Species	Type	Strain	Type	Subtype	Method	Type	Method	Type
BAVARIE1_0017	Yellow fever virus	No Type	17D Stamaril	Huh	7	Absorbance	MTS - 498nm	Microscopy	Tox scoring

Needs more data.

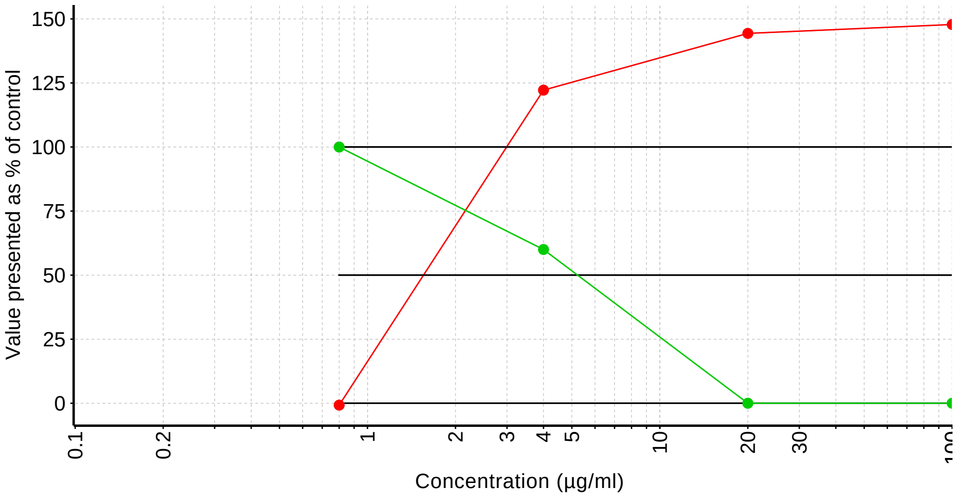

CC50	= 5.23
EC50	= 1.55
EC90	
SI	= 3.37
SS	= 6.56
TI	= 3.46
Max %	75.27
[] Max	2.78
Approved AV	1
Interesting AV	0
Page	17 / 96

Anti-Viral
Anti-Metabolic

Export chart data to CSV

**Summary values**			
**Statistic**	**CC50**	**EC50**	**EC90**
Median	= 5.23	= 1.55	= 2.62
Med.Abs.Dev.			
Mean	= 5.23	= 1.55	= 2.62
Stdev.			

Compound	Virus			Cell		AV Method		AM Method	
Primary code	Species	Type	Strain	Type	Subtype	Method	Type	Method	Type
BAVARIE1_0018	Yellow fever virus	No Type	17D Stamaril	Huh	7	Absorbance	MTS - 498nm	Microscopy	Tox scoring

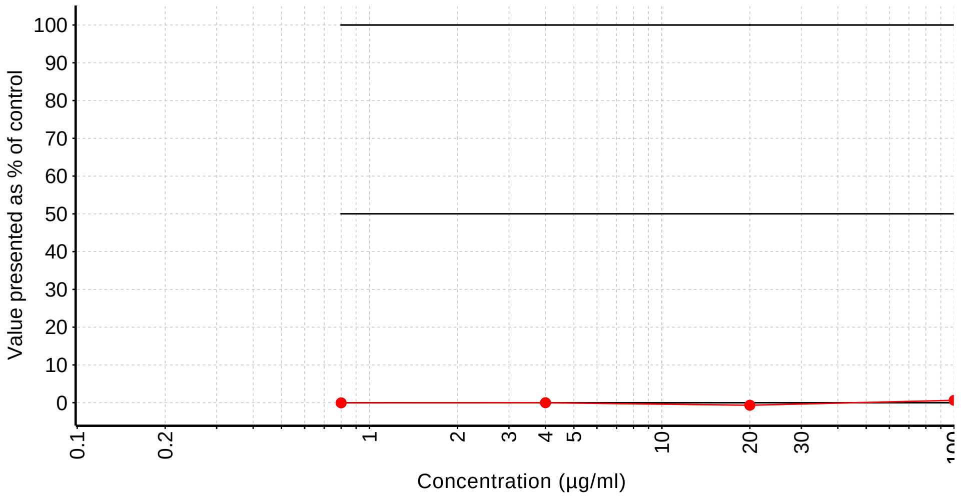

$CC50_{av}$		
EC50	>	100
EC90	>	100
SI_{av}		
SS_{av}		
TI_{av}		
Max %		0.62
[] Max		100
Approved AV		1
Interesting AV		0
Page		18 / 96

Anti-Viral
Anti-Metabolic

Export chart data to CSV

Summary values			
**Statistic**	**CC50**	**EC50**	**EC90**
Median		> 100	> 100
Med.Abs.Dev.			
Mean		> 100	> 100
Stdev.			

Compound	Virus			Cell		AV Method		AM Method	
Primary code	Species	Type	Strain	Type	Subtype	Method	Type	Method	Type
BAVARIE1_0019	Yellow fever virus	No Type	17D Stamaril	Huh	7	Absorbance	MTS - 498nm	Microscopy	Tox scoring

Needs more data.

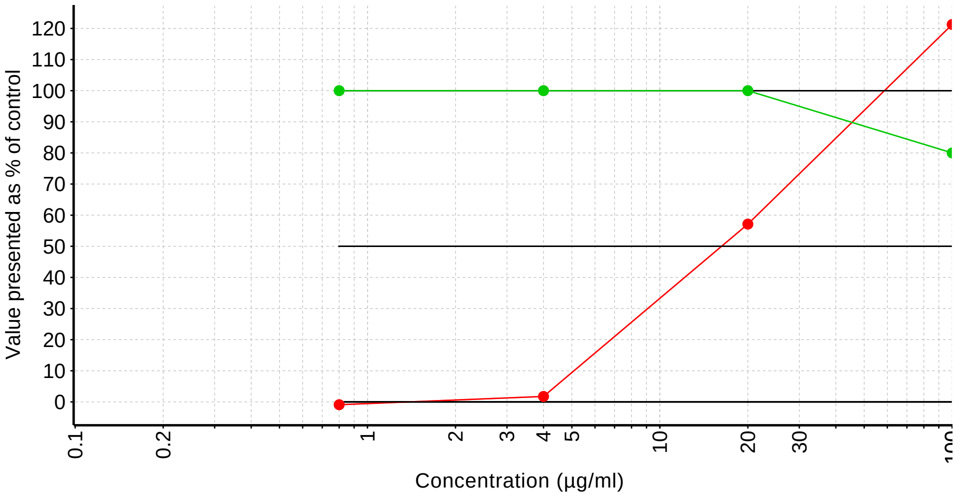

CC50	>	100
EC50	=	16.3
EC90		
SI	>	6.15
SS	>	19.2
TI	>	15.1
Max %		89.81
[] Max		60.8
Approved AV		1
Interesting AV		0
Page		19 / 96

Anti-Viral
Anti-Metabolic

Export chart data to CSV

Summary values			
**Statistic**	**CC50**	**EC50**	**EC90**
Median	> 100	= 16.3	= 45.6
Med.Abs.Dev.			
Mean	> 100	= 16.3	= 45.6
Stdev.			

**Compound**	**Virus**			**Cell**		**AV Method**		**AM Method**	
Primary code	Species	Type	Strain	Type	Subtype	Method	Type	Method	Type
BAVARIE1_0020	Yellow fever virus	No Type	17D Stamaril	Huh	7	Absorbance	MTS - 498nm	Microscopy	Tox scoring

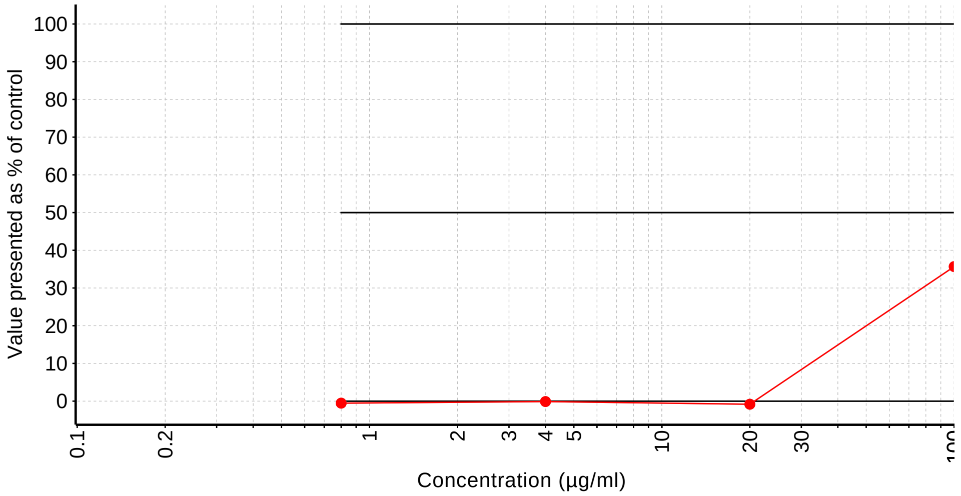

$CC50_{av}$		
EC50	>	100
EC90	>	100
SI_{av}		
SS_{av}		
TI_{av}		
Max %		35.67
[] Max		100
Approved AV		1
Interesting AV		0
Page		20 / 96

Anti-Viral
Anti-Metabolic

Export chart data to CSV

Summary values			
**Statistic**	**CC50**	**EC50**	**EC90**
Median		> 100	> 100
Med.Abs.Dev.			
Mean		> 100	> 100
Stdev.			

Compound	Virus			Cell		AV Method		AM Method	
Primary code	Species	Type	Strain	Type	Subtype	Method	Type	Method	Type
BAVARIE1_0021	Yellow fever virus	No Type	17D Stamaril	Huh	7	Absorbance	MTS - 498nm	Microscopy	Tox scoring

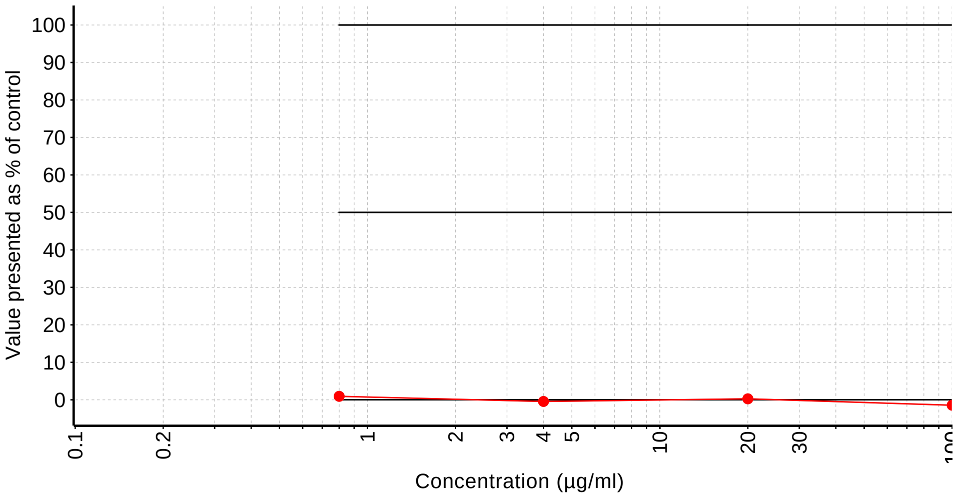

$CC50_{av}$		
EC50	>	100
EC90	>	100
SI_{av}		
SS_{av}		
TI_{av}		
Max %		0.93
[] Max		0.8
Approved AV		1
Interesting AV		0
Page		21 / 96

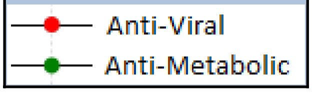

Export chart data to CSV

Summary values			
**Statistic**	**CC50**	**EC50**	**EC90**
Median		> 100	> 100
Med.Abs.Dev.			
Mean		> 100	> 100
Stdev.			

Compound	Virus			Cell		AV Method		AM Method	
Primary code	Species	Type	Strain	Type	Subtype	Method	Type	Method	Type
BAVARIE1_0022	Yellow fever virus	No Type	17D Stamaril	Huh	7	Absorbance	MTS - 498nm	Microscopy	Tox scoring

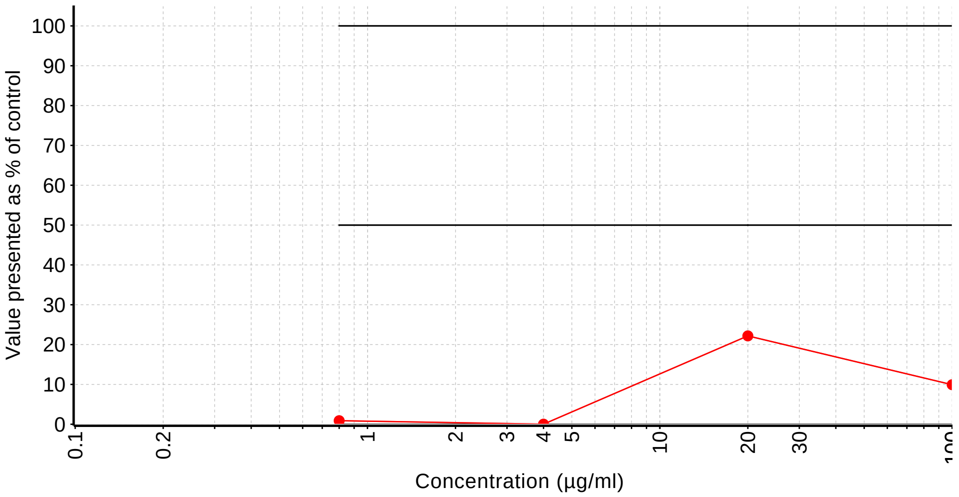

$CC50_{av}$	
EC50	
EC90	
SI_{av}	
SS_{av}	
TI_{av}	
Max %	22.18
[] Max	20
Approved AV	1
Interesting AV	0
Page	22 / 96

Anti-Viral
Anti-Metabolic

Export chart data to CSV

Summary values			
**Statistic**	**CC50**	**EC50**	**EC90**
Median			
Med.Abs.Dev.			
Mean			
Stdev.			

**Compound**	**Virus**			**Cell**		**AV Method**		**AM Method**	
Primary code	Species	Type	Strain	Type	Subtype	Method	Type	Method	Type
BAVARIE1_0023	Yellow fever virus	No Type	17D Stamaril	Huh	7	Absorbance	MTS - 498nm	Microscopy	Tox scoring

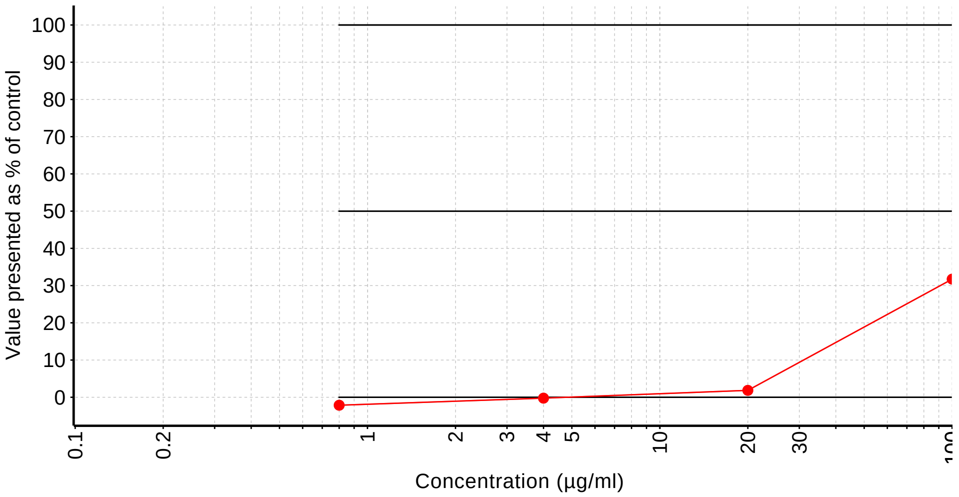

$CC50_{av}$		
EC50	>	100
EC90	>	100
SI_{av}		
SS_{av}		
TI_{av}		
Max %		31.73
[] Max		100
Approved AV		1
Interesting AV		0
Page		23 / 96

Anti-Viral
Anti-Metabolic

Export chart data to CSV

Summary values			
**Statistic**	**CC50**	**EC50**	**EC90**
Median		> 100	> 100
Med.Abs.Dev.			
Mean		> 100	> 100
Stdev.			

Compound	Virus			Cell		AV Method		AM Method	
Primary code	Species	Type	Strain	Type	Subtype	Method	Type	Method	Type
BAVARIE1_0024	Yellow fever virus	No Type	17D Stamaril	Huh	7	Absorbance	MTS - 498nm	Microscopy	Tox scoring

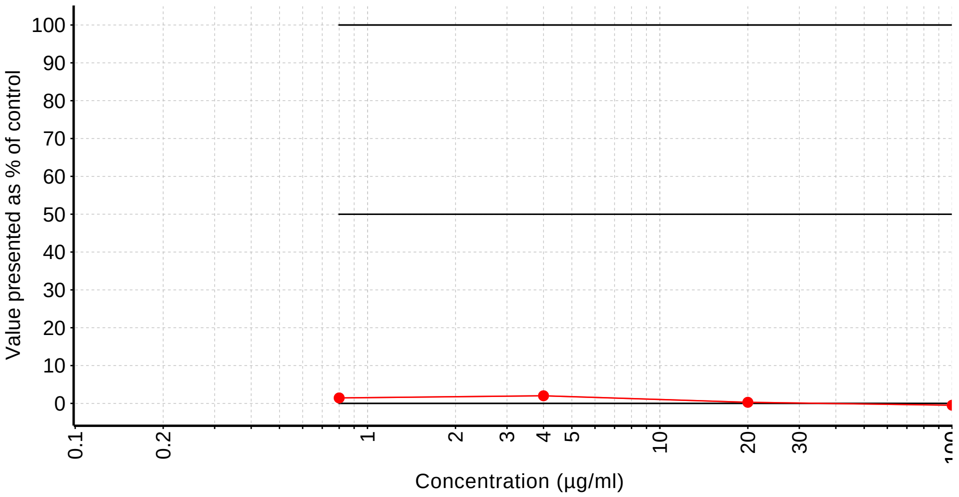

$CC50_{av}$		
EC50	>	100
EC90	>	100
SI_{av}		
SS_{av}		
TI_{av}		
Max %		2.02
[] Max		4
Approved AV		1
Interesting AV		0
Page		24 / 96

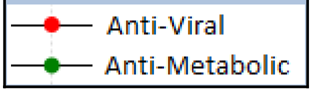

Export chart data to CSV

Summary values			
**Statistic**	**CC50**	**EC50**	**EC90**
Median		> 100	> 100
Med.Abs.Dev.			
Mean		> 100	> 100
Stdev.			

Compound	Virus			Cell		AV Method		AM Method	
Primary code	Species	Type	Strain	Type	Subtype	Method	Type	Method	Type
BAVARIE1_0025	Yellow fever virus	No Type	17D Stamaril	Huh	7	Absorbance	MTS - 498nm	Microscopy	Tox scoring

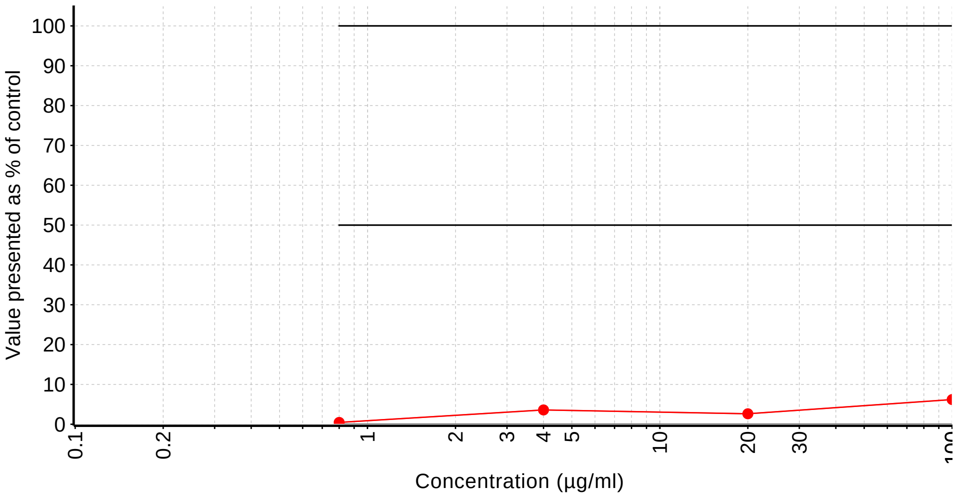

$CC50_{av}$		
EC50	>	100
EC90	>	100
SI_{av}		
SS_{av}		
TI_{av}		
Max %		6.20
[] Max		100
Approved AV		1
Interesting AV		0
Page		25 / 96

Anti-Viral
Anti-Metabolic

Export chart data to CSV

Summary values			
**Statistic**	**CC50**	**EC50**	**EC90**
Median		> 100	> 100
Med.Abs.Dev.			
Mean		> 100	> 100
Stdev.			

**Compound**	**Virus**			**Cell**		**AV Method**		**AM Method**	
Primary code	Species	Type	Strain	Type	Subtype	Method	Type	Method	Type
BAVARIE1_0026	Yellow fever virus	No Type	17D Stamaril	Huh	7	Absorbance	MTS - 498nm	Microscopy	Tox scoring

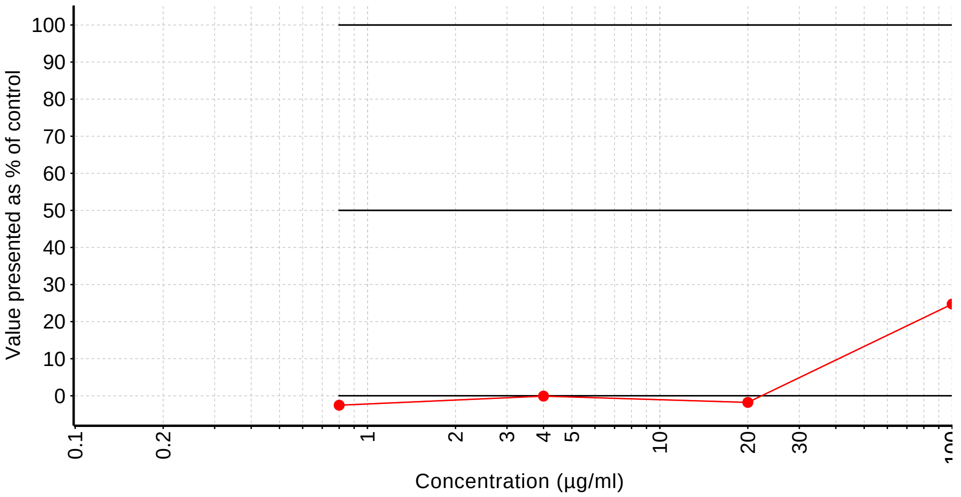

$CC50_{av}$		
EC50	>	100
EC90	>	100
SI_{av}		
SS_{av}		
TI_{av}		
Max %		24.74
[] Max		100
Approved AV		1
Interesting AV		0
Page		26 / 96

Anti-Viral
Anti-Metabolic

Export chart data to CSV

**Summary values**			
**Statistic**	**CC50**	**EC50**	**EC90**
Median		> 100	> 100
Med.Abs.Dev.			
Mean		> 100	> 100
Stdev.			

Compound	Virus			Cell		AV Method		AM Method	
Primary code	Species	Type	Strain	Type	Subtype	Method	Type	Method	Type
BAVARIE1_0027	Yellow fever virus	No Type	17D Stamaril	Huh	7	Absorbance	MTS - 498nm	Microscopy	Tox scoring

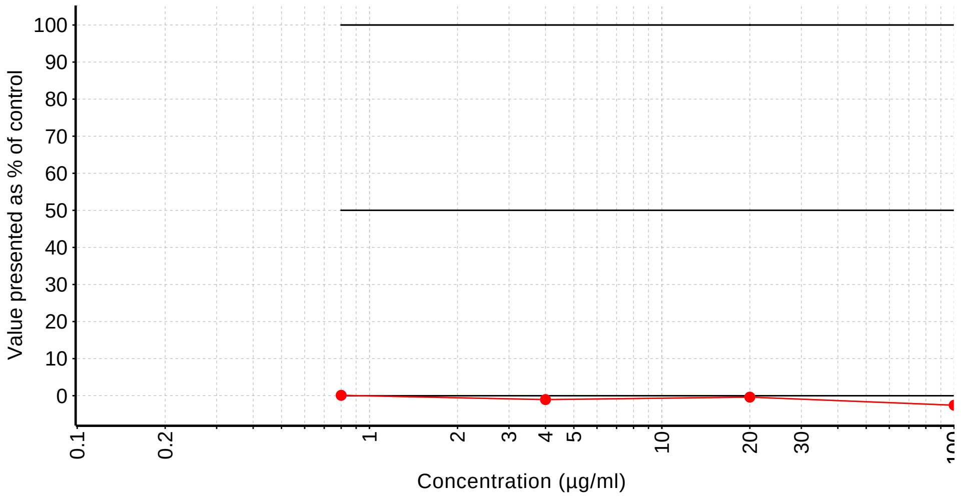

CC50av		
EC50	>	100
EC90	>	100
SIav		
SSav		
TIav		
Max %		0.10
[] Max		0.8
Approved AV		1
Interesting AV		0
Page		27 / 96

Anti-Viral
Anti-Metabolic

Export chart data to CSV

Summary values			
**Statistic**	**CC50**	**EC50**	**EC90**
Median		> 100	> 100
Med.Abs.Dev.			
Mean		> 100	> 100
Stdev.			

**Compound**	**Virus**			**Cell**		**AV Method**		**AM Method**	
Primary code	Species	Type	Strain	Type	Subtype	Method	Type	Method	Type
BAVARIE1_0028	Yellow fever virus	No Type	17D Stamaril	Huh	7	Absorbance	MTS - 498nm	Microscopy	Tox scoring

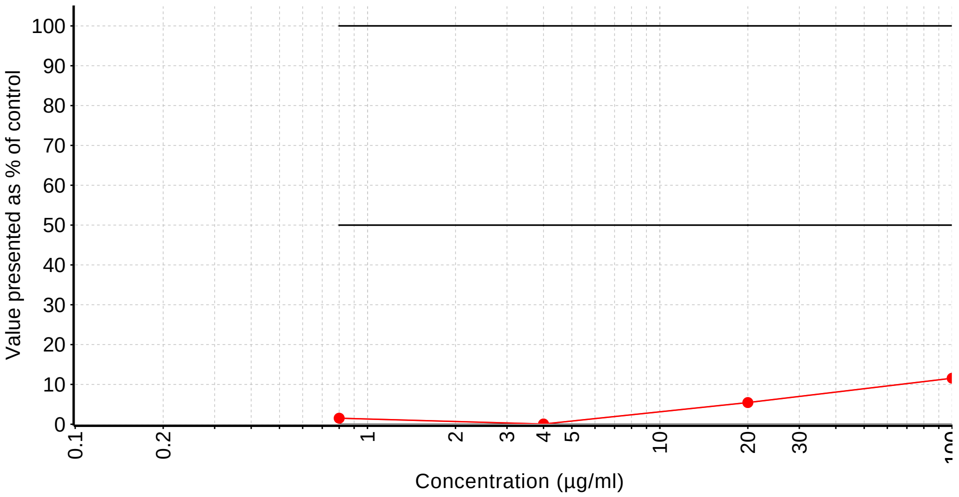

$CC50_{av}$		
EC50	>	100
EC90	>	100
SI_{av}		
SS_{av}		
TI_{av}		
Max %		11.56
[] Max		100
Approved AV		1
Interesting AV		0
Page		28 / 96

Anti-Viral
Anti-Metabolic

Export chart data to CSV

**Summary values**			
**Statistic**	**CC50**	**EC50**	**EC90**
Median		> 100	> 100
Med.Abs.Dev.			
Mean		> 100	> 100
Stdev.			

**Compound**	**Virus**			**Cell**		**AV Method**		**AM Method**	
Primary code	Species	Type	Strain	Type	Subtype	Method	Type	Method	Type
BAVARIE1_0029	Yellow fever virus	No Type	17D Stamaril	Huh	7	Absorbance	MTS - 498nm	Microscopy	Tox scoring

Needs more data.

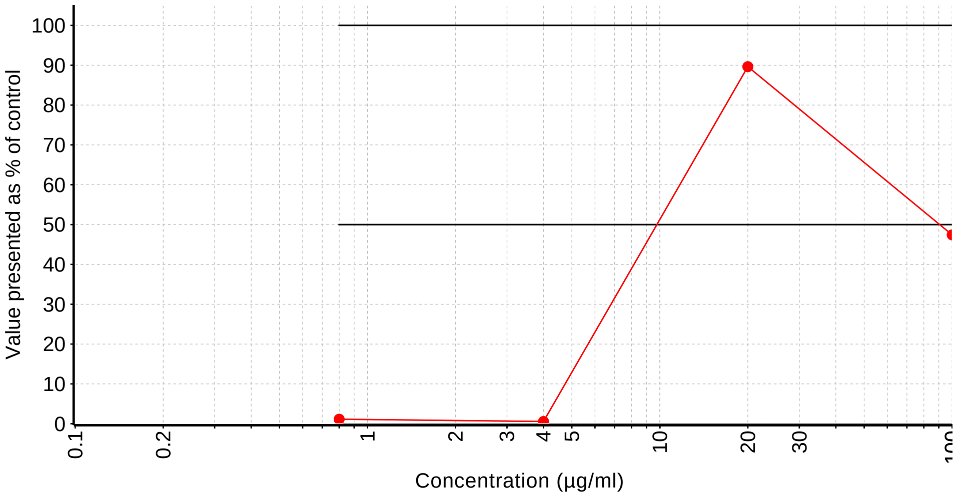

$CC50_{av}$	=	90.6
EC50	=	9.77
EC90		
SI_{av}	=	9.27
SS_{av}	=	19.2
TI_{av}	=	18.5
Max %		89.64
[] Max		20
Approved AV		1
Interesting AV		0
Page		29 / 96

Anti-Viral
Anti-Metabolic

Export chart data to CSV

**Summary values**			
**Statistic**	**CC50**	**EC50**	**EC90**
Median	= 90.6	= 9.77	
Med.Abs.Dev.			
Mean	= 90.6	= 9.77	
Stdev.			

Compound	Virus			Cell		AV Method		AM Method	
Primary code	Species	Type	Strain	Type	Subtype	Method	Type	Method	Type
BAVARIE1_0030	Yellow fever virus	No Type	17D Stamaril	Huh	7	Absorbance	MTS - 498nm	Microscopy	Tox scoring

Needs more data.

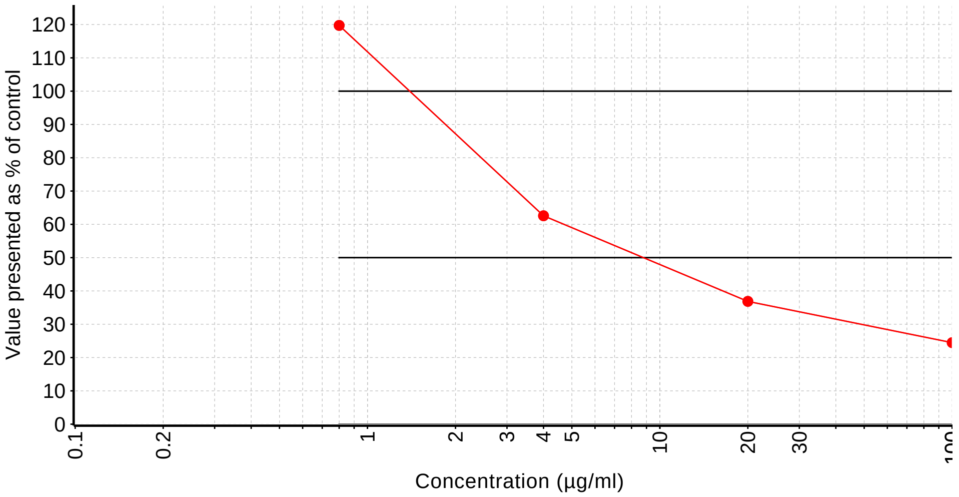

$CC50_{av}$	=	8.79
EC50	<	0.8
EC90	<	0.8
SI_{av}	>	11
SS_{av}	>	30.9
TI_{av}	>	32.2
Max %		119.74
[] Max		0.8
Approved AV		1
Interesting AV		0
Page		30 / 96

Anti-Viral
Anti-Metabolic

Export chart data to CSV

Summary values			
**Statistic**	**CC50**	**EC50**	**EC90**
Median	= 8.79	< 0.8	< 0.8
Med.Abs.Dev.			
Mean	= 8.79	< 0.8	< 0.8
Stdev.			

Compound	Virus			Cell		AV Method		AM Method	
Primary code	Species	Type	Strain	Type	Subtype	Method	Type	Method	Type
BAVARIE1_0031	Yellow fever virus	No Type	17D Stamaril	Huh	7	Absorbance	MTS - 498nm	Microscopy	Tox scoring

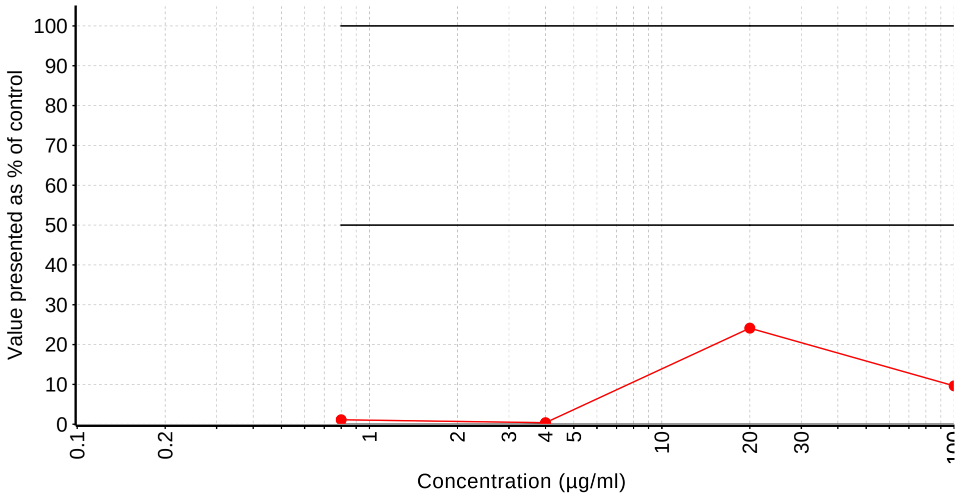

$CC50_{av}$	
EC50	
EC90	
SI_{av}	
SS_{av}	
TI_{av}	
Max %	24.12
[] Max	20
Approved AV	1
Interesting AV	0
Page	31 / 96

Anti-Viral
Anti-Metabolic

Export chart data to CSV

Summary values			
**Statistic**	**CC50**	**EC50**	**EC90**
Median			
Med.Abs.Dev.			
Mean			
Stdev.			

Compound	Virus			Cell		AV Method		AM Method	
Primary code	Species	Type	Strain	Type	Subtype	Method	Type	Method	Type
BAVARIE1_0032	Yellow fever virus	No Type	17D Stamaril	Huh	7	Absorbance	MTS - 498nm	Microscopy	Tox scoring

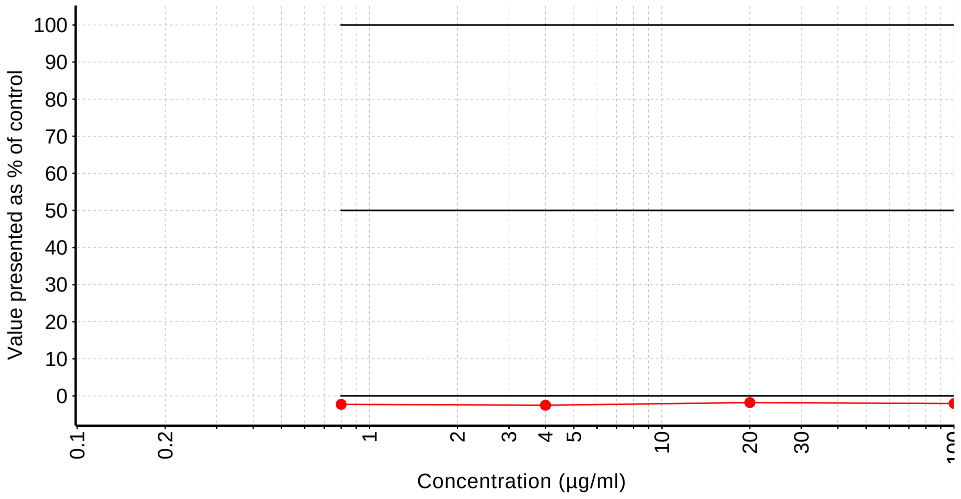

$CC50_{av}$		
EC50	>	100
EC90	>	100
SI_{av}		
SS_{av}		
TI_{av}		
Max %		-1.79
[] Max		20
Approved AV		1
Interesting AV		0
Page		32 / 96

Anti-Viral
Anti-Metabolic

Export chart data to CSV

Summary values			
**Statistic**	**CC50**	**EC50**	**EC90**
Median		> 100	> 100
Med.Abs.Dev.			
Mean		> 100	> 100
Stdev.			

**Compound**	**Virus**			**Cell**		**AV Method**		**AM Method**	
Primary code	Species	Type	Strain	Type	Subtype	Method	Type	Method	Type
BAVARIE1_0033	Yellow fever virus	No Type	17D Stamaril	Huh	7	Absorbance	MTS - 498nm	Microscopy	Tox scoring

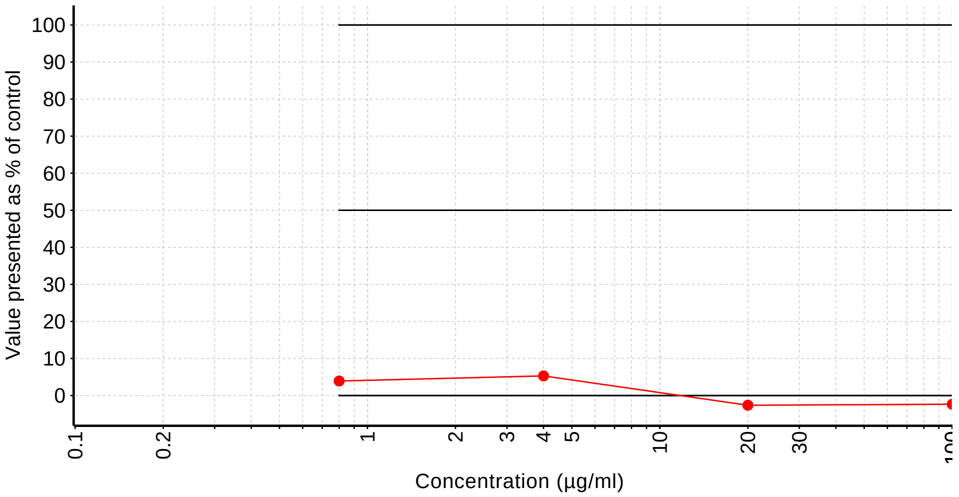

$CC50_{av}$		
EC50	>	100
EC90	>	100
SI_{av}		
SS_{av}		
TI_{av}		
Max %		5.30
[] Max		4
Approved AV		1
Interesting AV		0
Page		33 / 96

Anti-Viral
Anti-Metabolic

Export chart data to CSV

**Summary values**			
**Statistic**	**CC50**	**EC50**	**EC90**
Median		> 100	> 100
Med.Abs.Dev.			
Mean		> 100	> 100
Stdev.			

Compound	Virus			Cell		AV Method		AM Method	
Primary code	Species	Type	Strain	Type	Subtype	Method	Type	Method	Type
BAVARIE1_0034	Yellow fever virus	No Type	17D Stamaril	Huh	7	Absorbance	MTS - 498nm	Microscopy	Tox scoring

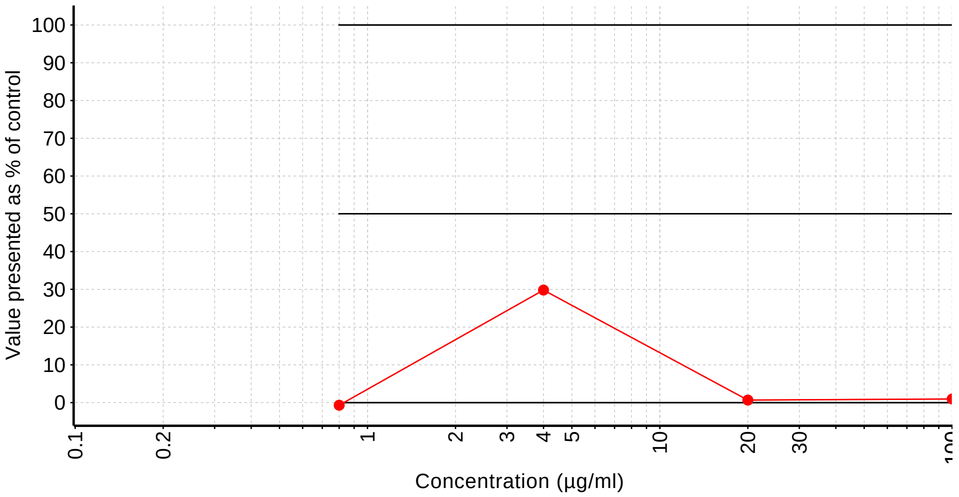

$CC50_{av}$	
EC50	
EC90	
SI_{av}	
SS_{av}	
TI_{av}	
Max %	29.80
[] Max	4
Approved AV	1
Interesting AV	0
Page	34 / 96

Anti-Viral
Anti-Metabolic

Export chart data to CSV

Summary values			
**Statistic**	**CC50**	**EC50**	**EC90**
Median			
Med.Abs.Dev.			
Mean			
Stdev.			

**Compound**	**Virus**			**Cell**		**AV Method**		**AM Method**	
Primary code	Species	Type	Strain	Type	Subtype	Method	Type	Method	Type
BAVARIE1_0035	Yellow fever virus	No Type	17D Stamaril	Huh	7	Absorbance	MTS - 498nm	Microscopy	Tox scoring

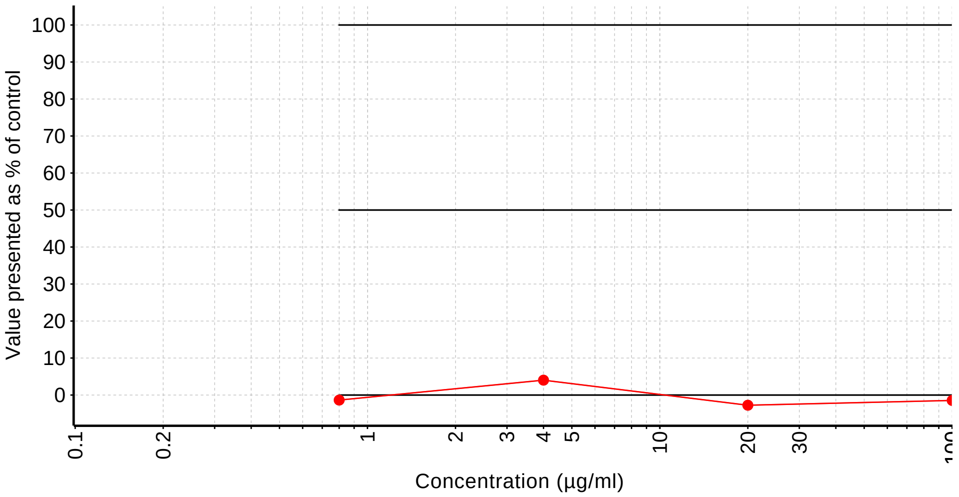

$CC50_{av}$		
EC50	>	100
EC90	>	100
SI_{av}		
SS_{av}		
TI_{av}		
Max %		4.02
[] Max		4
Approved AV		1
Interesting AV		0
Page		35 / 96

Anti-Viral
Anti-Metabolic

Export chart data to CSV

**Summary values**			
**Statistic**	**CC50**	**EC50**	**EC90**
Median		> 100	> 100
Med.Abs.Dev.			
Mean		> 100	> 100
Stdev.			

Compound	Virus			Cell		AV Method		AM Method	
Primary code	Species	Type	Strain	Type	Subtype	Method	Type	Method	Type
BAVARIE1_0036	Yellow fever virus	No Type	17D Stamaril	Huh	7	Absorbance	MTS - 498nm	Microscopy	Tox scoring

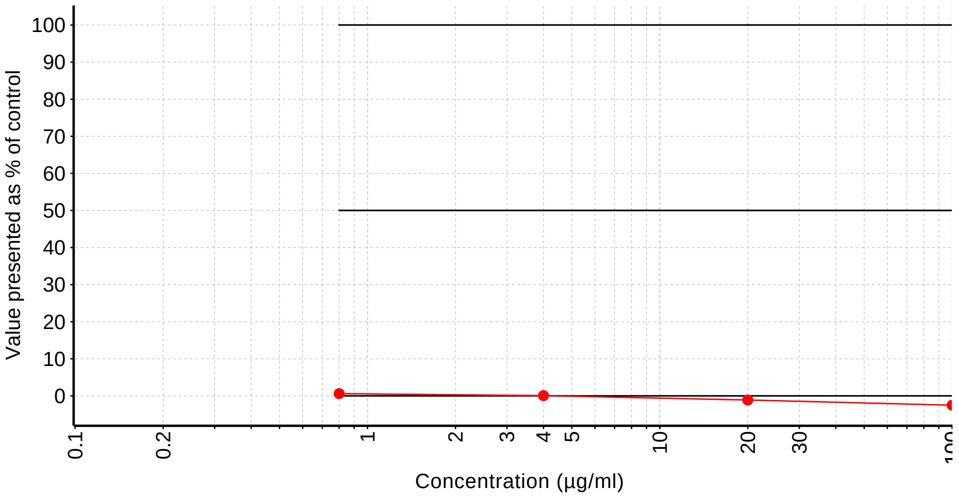

$CC50_{av}$		
EC50	>	100
EC90	>	100
SI_{av}		
SS_{av}		
TI_{av}		
Max %		0.61
[] Max		0.8
Approved AV		1
Interesting AV		0
Page		36 / 96

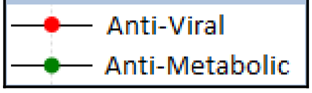

Export chart data to CSV

Summary values			
**Statistic**	**CC50**	**EC50**	**EC90**
Median		> 100	> 100
Med.Abs.Dev.			
Mean		> 100	> 100
Stdev.			

Compound	Virus			Cell		AV Method		AM Method	
Primary code	Species	Type	Strain	Type	Subtype	Method	Type	Method	Type
BAVARIE1_0037	Yellow fever virus	No Type	17D Stamaril	Huh	7	Absorbance	MTS - 498nm	Microscopy	Tox scoring

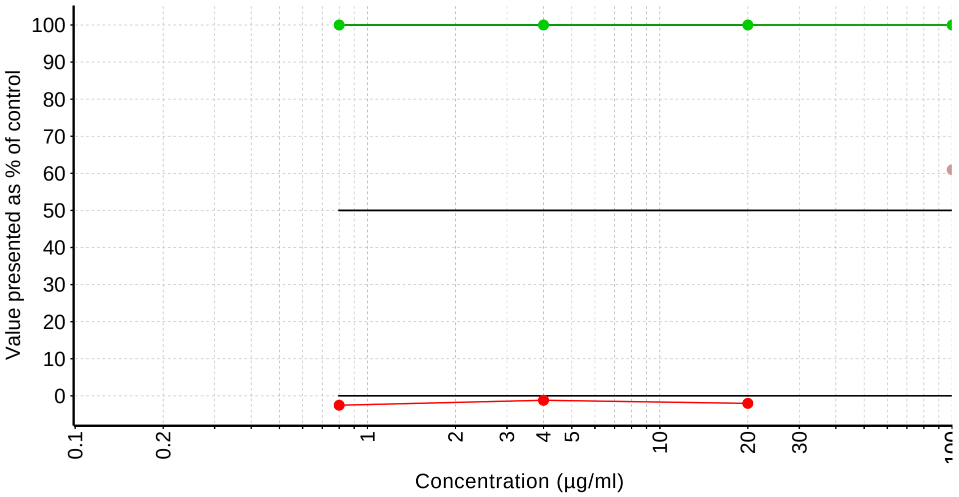

CC50	>	100
EC50	>	100
EC90	>	100
SI		
SS		
TI		
Max %		-1.20
[] Max		4
Approved AV		1
Interesting AV		0
Page		37 / 96

Anti-Viral
Anti-Metabolic

Export chart data to CSV

Summary values			
**Statistic**	**CC50**	**EC50**	**EC90**
Median		> 20	> 20
Med.Abs.Dev.			
Mean		> 20	> 20
Stdev.			

Compound	Virus			Cell		AV Method		AM Method	
Primary code	Species	Type	Strain	Type	Subtype	Method	Type	Method	Type
BAVARIE1_0038	Yellow fever virus	No Type	17D Stamaril	Huh	7	Absorbance	MTS - 498nm	Microscopy	Tox scoring

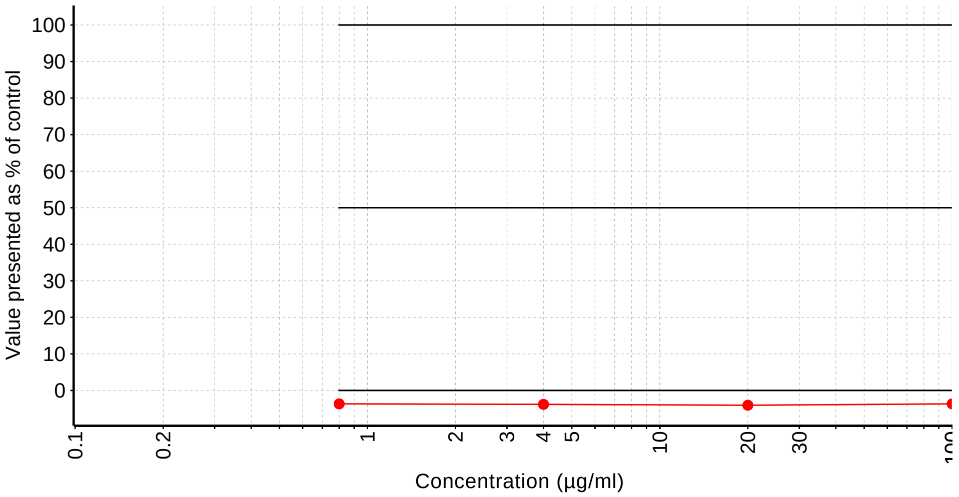

$CC50_{av}$		
EC50	>	100
EC90	>	100
SI_{av}		
SS_{av}		
TI_{av}		
Max %		-3.67
[] Max		100
Approved AV		1
Interesting AV		0
Page		38 / 96

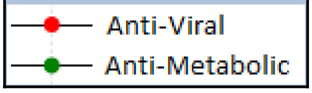

Export chart data to CSV

Summary values			
**Statistic**	**CC50**	**EC50**	**EC90**
Median		> 100	> 100
Med.Abs.Dev.			
Mean		> 100	> 100
Stdev.			

Compound	Virus			Cell		AV Method		AM Method	
Primary code	Species	Type	Strain	Type	Subtype	Method	Type	Method	Type
BAVARIE1_0039	Yellow fever virus	No Type	17D Stamaril	Huh	7	Absorbance	MTS - 498nm	Microscopy	Tox scoring

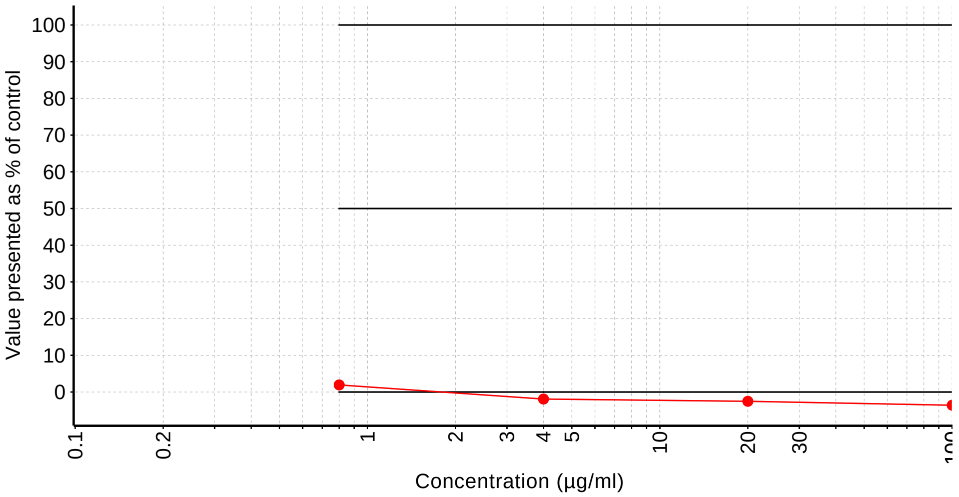

$CC50_{av}$		
EC50	>	100
EC90	>	100
SI_{av}		
SS_{av}		
TI_{av}		
Max %		1.93
[] Max		0.8
Approved AV		1
Interesting AV		0
Page		39 / 96

Anti-Viral
Anti-Metabolic

Export chart data to CSV

Summary values			
**Statistic**	**CC50**	**EC50**	**EC90**
Median		> 100	> 100
Med.Abs.Dev.			
Mean		> 100	> 100
Stdev.			

Compound	Virus			Cell		AV Method		AM Method	
Primary code	Species	Type	Strain	Type	Subtype	Method	Type	Method	Type
BAVARIE1_0040	Yellow fever virus	No Type	17D Stamaril	Huh	7	Absorbance	MTS - 498nm	Microscopy	Tox scoring

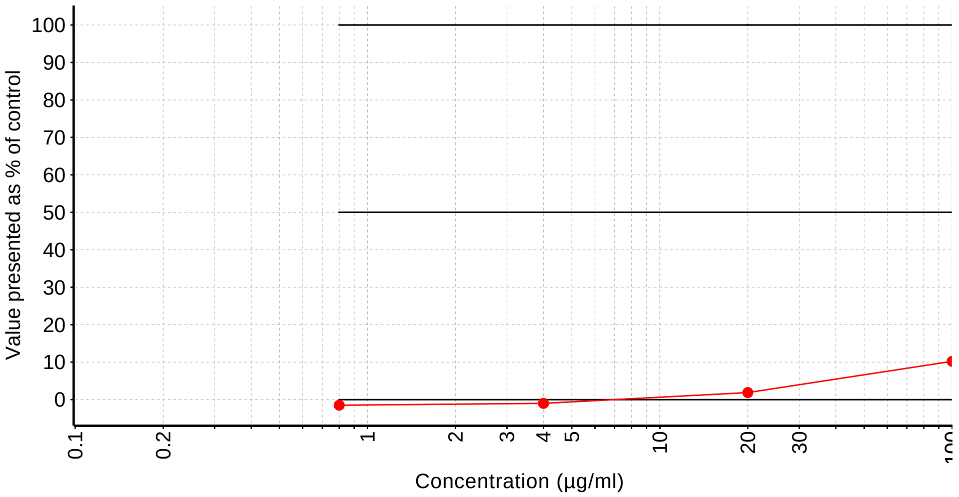

$CC50_{av}$		
EC50	>	100
EC90	>	100
SI_{av}		
SS_{av}		
TI_{av}		
Max %		10.22
[] Max		100
Approved AV		1
Interesting AV		0
Page		40 / 96

Anti-Viral
Anti-Metabolic

Export chart data to CSV

Summary values			
**Statistic**	**CC50**	**EC50**	**EC90**
Median		> 100	> 100
Med.Abs.Dev.			
Mean		> 100	> 100
Stdev.			

Compound	Virus			Cell		AV Method		AM Method	
Primary code	Species	Type	Strain	Type	Subtype	Method	Type	Method	Type
BAVARIE1_0041	Yellow fever virus	No Type	17D Stamaril	Huh	7	Absorbance	MTS - 498nm	Microscopy	Tox scoring

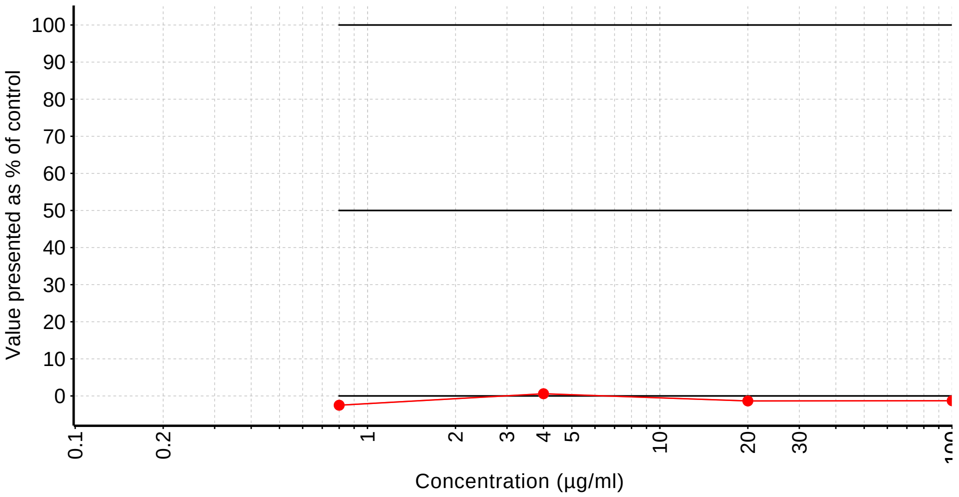

$CC50_{av}$		
EC50	>	100
EC90	>	100
SI_{av}		
SS_{av}		
TI_{av}		
Max %		0.60
[] Max		4
Approved AV		1
Interesting AV		0
Page		41 / 96

Anti-Viral
Anti-Metabolic

Export chart data to CSV

Summary values			
**Statistic**	**CC50**	**EC50**	**EC90**
Median		> 100	> 100
Med.Abs.Dev.			
Mean		> 100	> 100
Stdev.			

Compound	Virus			Cell		AV Method		AM Method	
Primary code	Species	Type	Strain	Type	Subtype	Method	Type	Method	Type
BAVARIE1_0042	Yellow fever virus	No Type	17D Stamaril	Huh	7	Absorbance	MTS - 498nm	Microscopy	Tox scoring

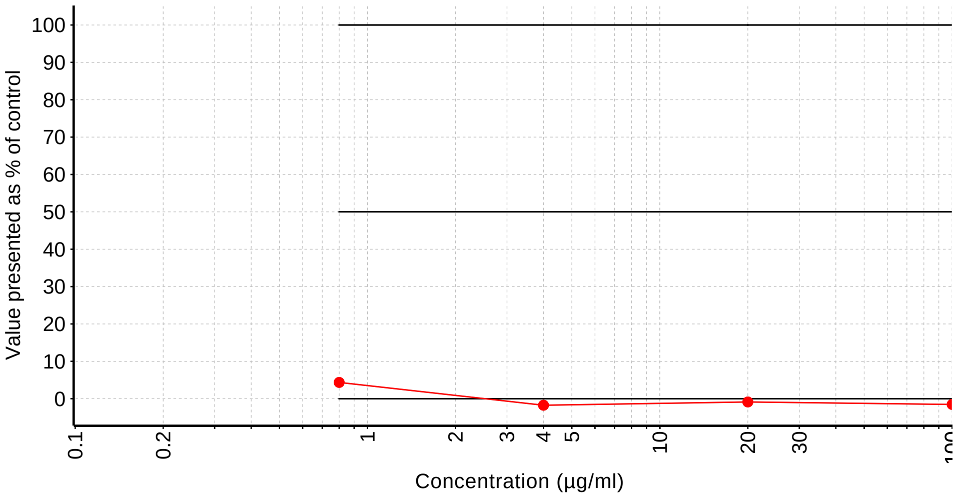

$CC50_{av}$		
EC50	>	100
EC90	>	100
SI_{av}		
SS_{av}		
TI_{av}		
Max %		4.35
[] Max		0.8
Approved AV		1
Interesting AV		0
Page		42 / 96

Anti-Viral
Anti-Metabolic

Export chart data to CSV

Summary values			
**Statistic**	**CC50**	**EC50**	**EC90**
Median		> 100	> 100
Med.Abs.Dev.			
Mean		> 100	> 100
Stdev.			

Compound	Virus			Cell		AV Method		AM Method	
Primary code	Species	Type	Strain	Type	Subtype	Method	Type	Method	Type
BAVARIE1_0043	Yellow fever virus	No Type	17D Stamaril	Huh	7	Absorbance	MTS - 498nm	Microscopy	Tox scoring

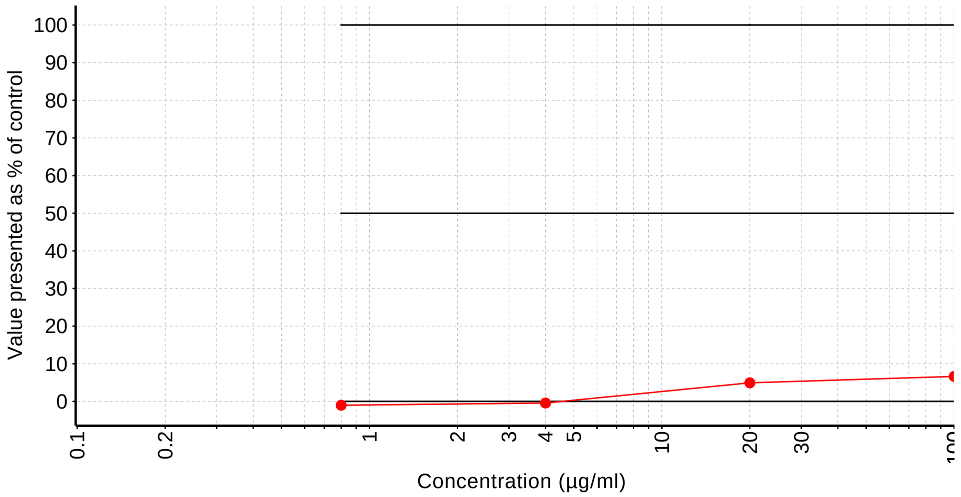

$CC50_{av}$		
EC50	>	100
EC90	>	100
SI_{av}		
SS_{av}		
TI_{av}		
Max %		6.63
[] Max		100
Approved AV		1
Interesting AV		0
Page		43 / 96

Anti-Viral
Anti-Metabolic

Export chart data to CSV

Summary values			
**Statistic**	**CC50**	**EC50**	**EC90**
Median		> 100	> 100
Med.Abs.Dev.			
Mean		> 100	> 100
Stdev.			

Compound	Virus			Cell		AV Method		AM Method	
Primary code	Species	Type	Strain	Type	Subtype	Method	Type	Method	Type
BAVARIE1_0044	Yellow fever virus	No Type	17D Stamaril	Huh	7	Absorbance	MTS - 498nm	Microscopy	Tox scoring

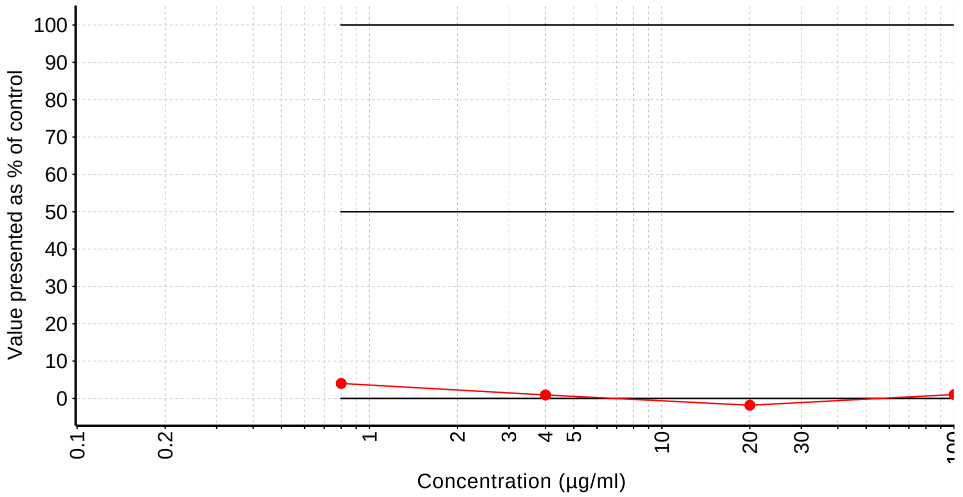

$CC50_{av}$		
EC50	>	100
EC90	>	100
SI_{av}		
SS_{av}		
TI_{av}		
Max %		4.01
[] Max		0.8
Approved AV		1
Interesting AV		0
Page		44 / 96

Anti-Viral
Anti-Metabolic

Export chart data to CSV

Summary values			
**Statistic**	**CC50**	**EC50**	**EC90**
Median		> 100	> 100
Med.Abs.Dev.			
Mean		> 100	> 100
Stdev.			

Compound	Virus			Cell		AV Method		AM Method	
Primary code	Species	Type	Strain	Type	Subtype	Method	Type	Method	Type
BAVARIE1_0045	Yellow fever virus	No Type	17D Stamaril	Huh	7	Absorbance	MTS - 498nm	Microscopy	Tox scoring

Needs more data.

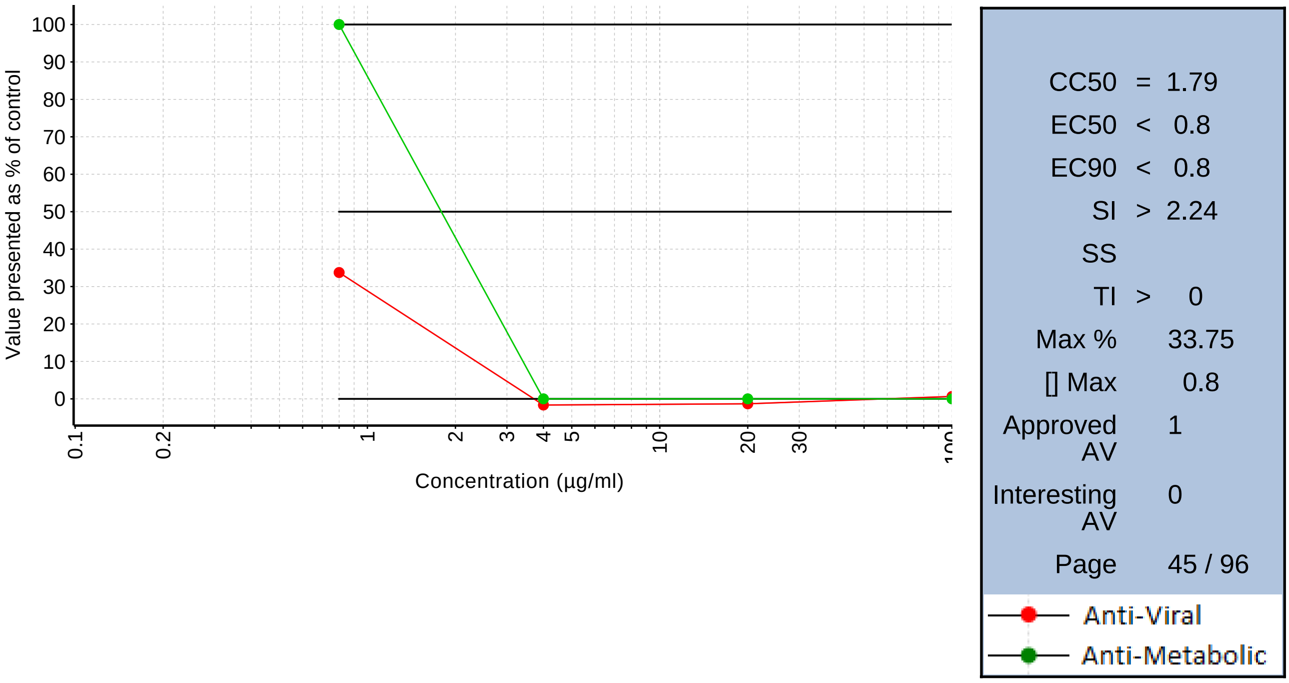

Export chart data to CSV

Summary values			
**Statistic**	**CC50**	**EC50**	**EC90**
Median	= 1.79	< 0.8	< 0.8
Med.Abs.Dev.			
Mean	= 1.79	< 0.8	< 0.8
Stdev.			

**Compound**	**Virus**			**Cell**		**AV Method**		**AM Method**	
Primary code	Species	Type	Strain	Type	Subtype	Method	Type	Method	Type
BAVARIE1_0046	Yellow fever virus	No Type	17D Stamaril	Huh	7	Absorbance	MTS - 498nm	Microscopy	Tox scoring

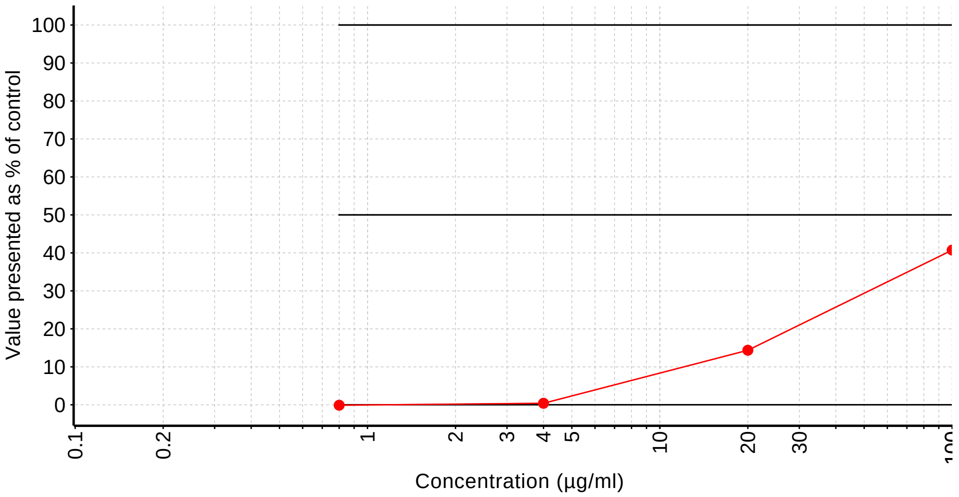

$CC50_{av}$		
EC50	>	100
EC90	>	100
SI_{av}		
SS_{av}		
TI_{av}		
Max %		40.74
[] Max		100
Approved AV		1
Interesting AV		0
Page		46 / 96

Anti-Viral
Anti-Metabolic

Export chart data to CSV

Summary values			
**Statistic**	**CC50**	**EC50**	**EC90**
Median		> 100	> 100
Med.Abs.Dev.			
Mean		> 100	> 100
Stdev.			

**Compound**	**Virus**			**Cell**		**AV Method**		**AM Method**	
Primary code	Species	Type	Strain	Type	Subtype	Method	Type	Method	Type
BAVARIE1_0047	Yellow fever virus	No Type	17D Stamaril	Huh	7	Absorbance	MTS - 498nm	Microscopy	Tox scoring

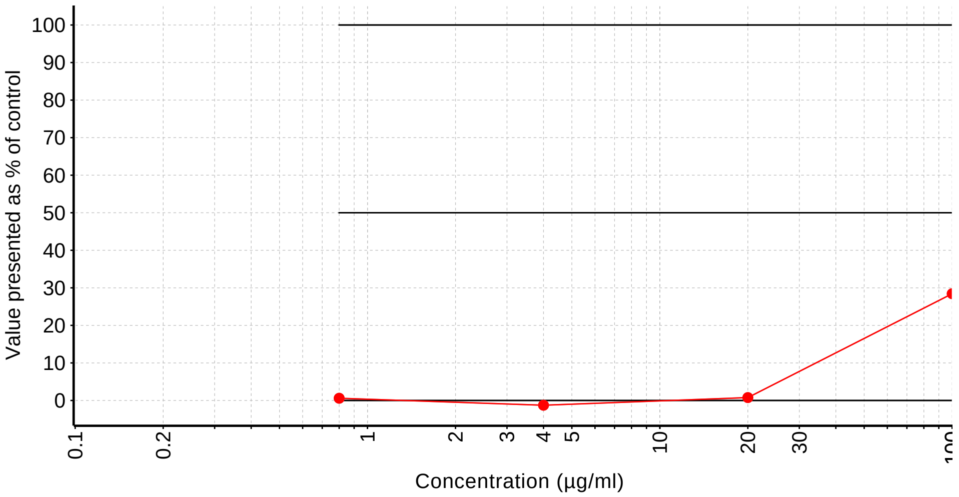

$CC50_{av}$		
EC50	>	100
EC90	>	100
SI_{av}		
SS_{av}		
TI_{av}		
Max %		28.43
[] Max		100
Approved AV		1
Interesting AV		0
Page		47 / 96

Anti-Viral
Anti-Metabolic

Export chart data to CSV

Summary values			
**Statistic**	**CC50**	**EC50**	**EC90**
Median		> 100	> 100
Med.Abs.Dev.			
Mean		> 100	> 100
Stdev.			

Compound	Virus			Cell		AV Method		AM Method	
Primary code	Species	Type	Strain	Type	Subtype	Method	Type	Method	Type
BAVARIE1_0048	Yellow fever virus	No Type	17D Stamaril	Huh	7	Absorbance	MTS - 498nm	Microscopy	Tox scoring

Needs more data.

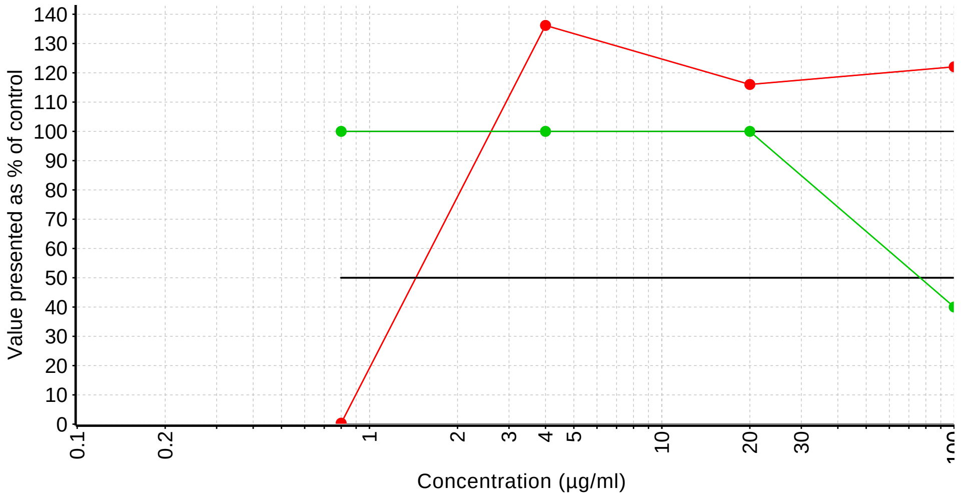

CC50	=	76.5
EC50	=	1.44
EC90	=	2.31
SI	=	53.1
SS	=	63.2
TI	=	109
Max %		100.00
[] Max		3.15
Approved AV		1
Interesting AV		0
Page		48 / 96

Anti-Viral
Anti-Metabolic

Export chart data to CSV

Summary values			
**Statistic**	**CC50**	**EC50**	**EC90**
Median	= 76.5	= 1.44	= 2.31
Med.Abs.Dev.			
Mean	= 76.5	= 1.44	= 2.31
Stdev.			

Compound	Virus			Cell		AV Method		AM Method	
Primary code	Species	Type	Strain	Type	Subtype	Method	Type	Method	Type
BAVARIE1_0049	Yellow fever virus	No Type	17D Stamaril	Huh	7	Absorbance	MTS - 498nm	Microscopy	Tox scoring

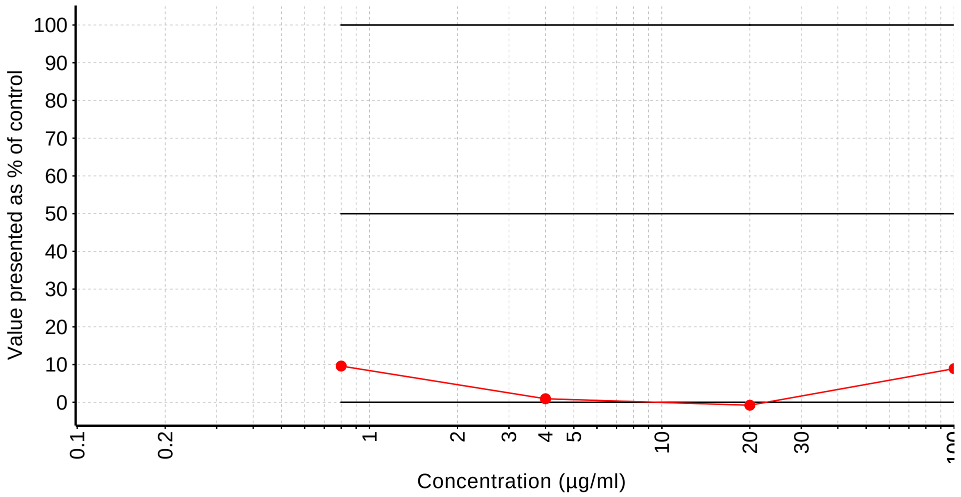

$CC50_{av}$		
EC50	>	100
EC90	>	100
SI_{av}		
SS_{av}		
TI_{av}		
Max %		9.60
[] Max		0.8
Approved AV		1
Interesting AV		0
Page		49 / 96

Anti-Viral
Anti-Metabolic

Export chart data to CSV

Summary values			
**Statistic**	**CC50**	**EC50**	**EC90**
Median		> 100	> 100
Med.Abs.Dev.			
Mean		> 100	> 100
Stdev.			

Compound	Virus			Cell		AV Method		AM Method	
Primary code	Species	Type	Strain	Type	Subtype	Method	Type	Method	Type
BAVARIE1_0050	Yellow fever virus	No Type	17D Stamaril	Huh	7	Absorbance	MTS - 498nm	Microscopy	Tox scoring

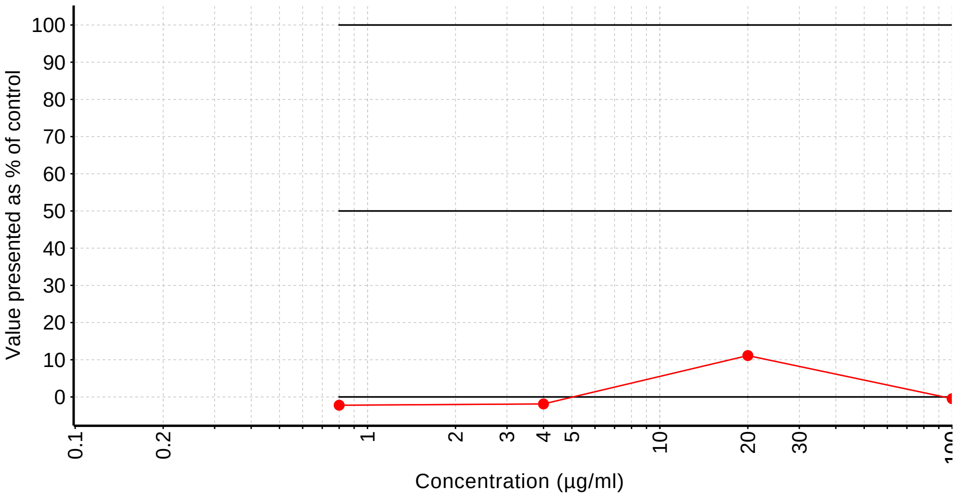

$CC50_{av}$		
EC50	>	100
EC90	>	100
SI_{av}		
SS_{av}		
TI_{av}		
Max %		11.13
[] Max		20
Approved AV		1
Interesting AV		0
Page		50 / 96

Anti-Viral
Anti-Metabolic

Export chart data to CSV

Summary values			
**Statistic**	**CC50**	**EC50**	**EC90**
Median		> 100	> 100
Med.Abs.Dev.			
Mean		> 100	> 100
Stdev.			

Compound	Virus			Cell		AV Method		AM Method	
Primary code	Species	Type	Strain	Type	Subtype	Method	Type	Method	Type
BAVARIE1_0051	Yellow fever virus	No Type	17D Stamaril	Huh	7	Absorbance	MTS - 498nm	Microscopy	Tox scoring

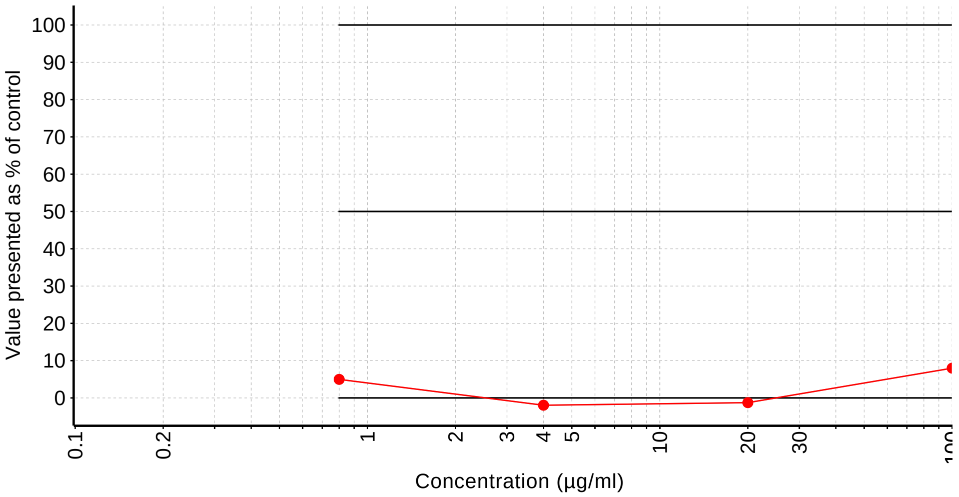

CC50$_{av}$		
EC50	>	100
EC90	>	100
SI$_{av}$		
SS$_{av}$		
TI$_{av}$		
Max %		7.99
[] Max		100
Approved AV		1
Interesting AV		0
Page		51 / 96

Anti-Viral
Anti-Metabolic

Export chart data to CSV

Summary values			
**Statistic**	**CC50**	**EC50**	**EC90**
Median		> 100	> 100
Med.Abs.Dev.			
Mean		> 100	> 100
Stdev.			

**Compound**	**Virus**			**Cell**		**AV Method**		**AM Method**	
Primary code	Species	Type	Strain	Type	Subtype	Method	Type	Method	Type
BAVARIE1_0052	Yellow fever virus	No Type	17D Stamaril	Huh	7	Absorbance	MTS - 498nm	Microscopy	Tox scoring

Needs more data.

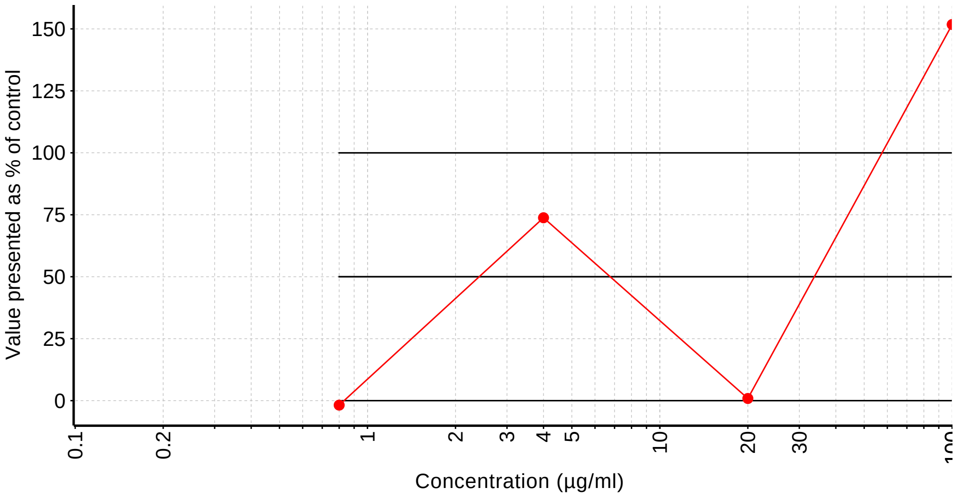

$CC50_{av}$	=	6.76
EC50	=	2.41
EC90	=	51.7
SI_{av}	=	2.81
SS_{av}	=	29.3
TI_{av}	=	13.1
Max %		151.76
[] Max		100
Approved AV		1
Interesting AV		0
Page		52 / 96

Anti-Viral
Anti-Metabolic

Export chart data to CSV

**Summary values**			
**Statistic**	**CC50**	**EC50**	**EC90**
Median	= 6.76	= 2.41	= 51.7
Med.Abs.Dev.			
Mean	= 6.76	= 2.41	= 51.7
Stdev.			

Compound	Virus			Cell		AV Method		AM Method	
Primary code	Species	Type	Strain	Type	Subtype	Method	Type	Method	Type
BAVARIE1_0053	Yellow fever virus	No Type	17D Stamaril	Huh	7	Absorbance	MTS - 498nm	Microscopy	Tox scoring

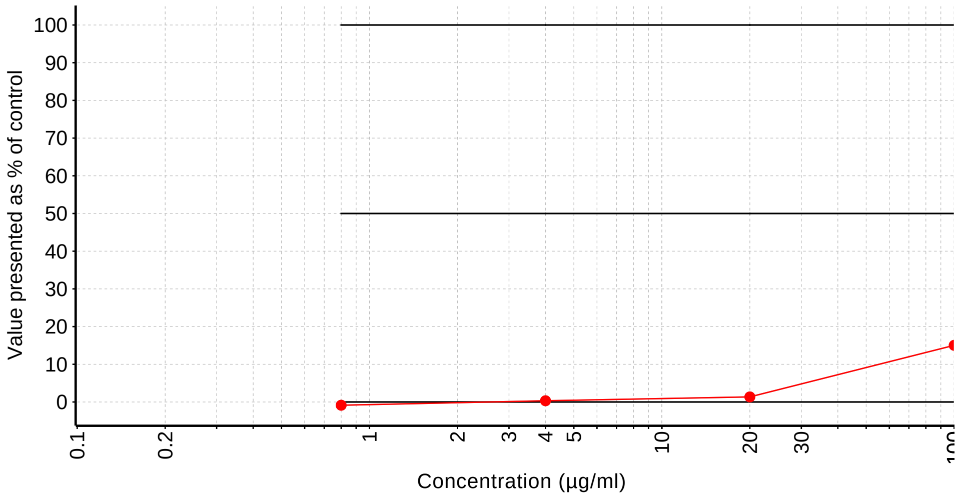

$CC50_{av}$		
EC50	>	100
EC90	>	100
SI_{av}		
SS_{av}		
TI_{av}		
Max %		15.03
[] Max		100
Approved AV		1
Interesting AV		0
Page		53 / 96

Anti-Viral
Anti-Metabolic

Export chart data to CSV

Summary values			
**Statistic**	**CC50**	**EC50**	**EC90**
Median		> 100	> 100
Med.Abs.Dev.			
Mean		> 100	> 100
Stdev.			

**Compound**	**Virus**			**Cell**		**AV Method**		**AM Method**	
Primary code	Species	Type	Strain	Type	Subtype	Method	Type	Method	Type
BAVARIE1_0054	Yellow fever virus	No Type	17D Stamaril	Huh	7	Absorbance	MTS - 498nm	Microscopy	Tox scoring

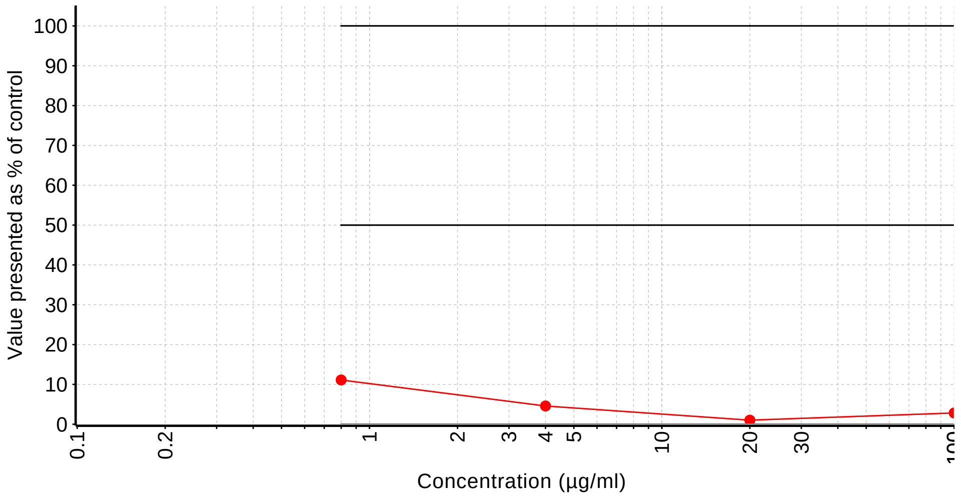

$CC50_{av}$		
EC50	>	100
EC90	>	100
SI_{av}		
SS_{av}		
TI_{av}		
Max %		11.11
[] Max		0.8
Approved AV		1
Interesting AV		0
Page		54 / 96

Anti-Viral
Anti-Metabolic

Export chart data to CSV

**Summary values**			
**Statistic**	**CC50**	**EC50**	**EC90**
Median		> 100	> 100
Med.Abs.Dev.			
Mean		> 100	> 100
Stdev.			

Compound	Virus			Cell		AV Method		AM Method	
Primary code	Species	Type	Strain	Type	Subtype	Method	Type	Method	Type
BAVARIE1_0055	Yellow fever virus	No Type	17D Stamaril	Huh	7	Absorbance	MTS - 498nm	Microscopy	Tox scoring

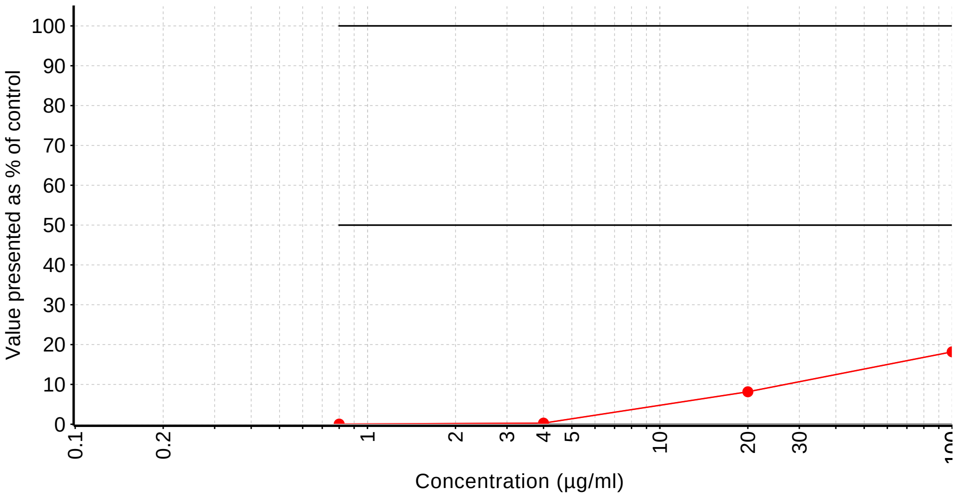

$CC50_{av}$		
EC50	>	100
EC90	>	100
SI_{av}		
SS_{av}		
TI_{av}		
Max %		18.18
[] Max		100
Approved AV		1
Interesting AV		0
Page		55 / 96

Anti-Viral
Anti-Metabolic

Export chart data to CSV

Summary values			
**Statistic**	**CC50**	**EC50**	**EC90**
Median		> 100	> 100
Med.Abs.Dev.			
Mean		> 100	> 100
Stdev.			

Compound	Virus			Cell		AV Method		AM Method	
Primary code	Species	Type	Strain	Type	Subtype	Method	Type	Method	Type
BAVARIE1_0056	Yellow fever virus	No Type	17D Stamaril	Huh	7	Absorbance	MTS - 498nm	Microscopy	Tox scoring

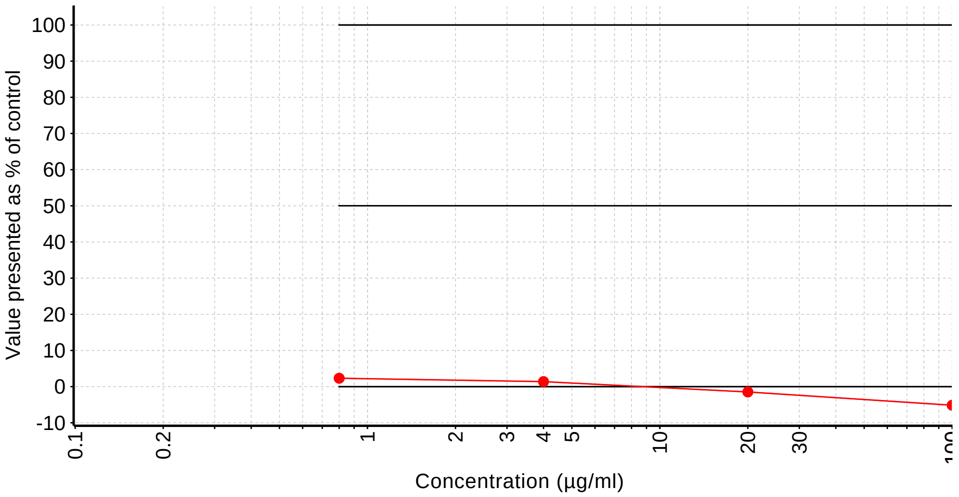

CC50$_{av}$		
EC50	>	100
EC90	>	100
SI$_{av}$		
SS$_{av}$		
TI$_{av}$		
Max %		2.32
[] Max		0.8
Approved AV		1
Interesting AV		0
Page		56 / 96

Anti-Viral
Anti-Metabolic

Export chart data to CSV

Summary values			
**Statistic**	**CC50**	**EC50**	**EC90**
Median		> 100	> 100
Med.Abs.Dev.			
Mean		> 100	> 100
Stdev.			

Compound	Virus			Cell		AV Method		AM Method	
Primary code	Species	Type	Strain	Type	Subtype	Method	Type	Method	Type
BAVARIE1_0057	Yellow fever virus	No Type	17D Stamaril	Huh	7	Absorbance	MTS - 498nm	Microscopy	Tox scoring

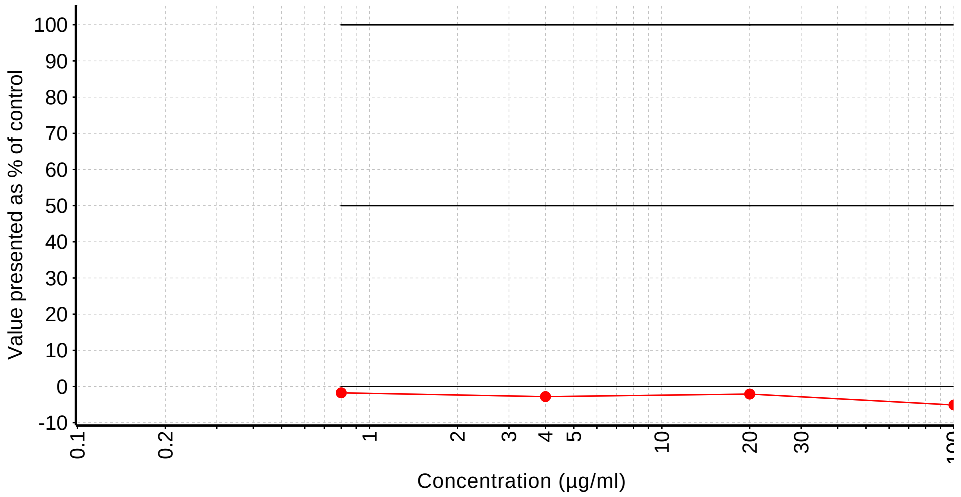

CC50$_{av}$		
EC50	>	100
EC90	>	100
SI$_{av}$		
SS$_{av}$		
TI$_{av}$		
Max %		-1.75
[] Max		0.8
Approved AV		1
Interesting AV		0
Page		57 / 96

Anti-Viral
Anti-Metabolic

Export chart data to CSV

Summary values			
**Statistic**	**CC50**	**EC50**	**EC90**
Median		> 100	> 100
Med.Abs.Dev.			
Mean		> 100	> 100
Stdev.			

Compound	Virus			Cell		AV Method		AM Method	
Primary code	Species	Type	Strain	Type	Subtype	Method	Type	Method	Type
BAVARIE1_0058	Yellow fever virus	No Type	17D Stamaril	Huh	7	Absorbance	MTS - 498nm	Microscopy	Tox scoring

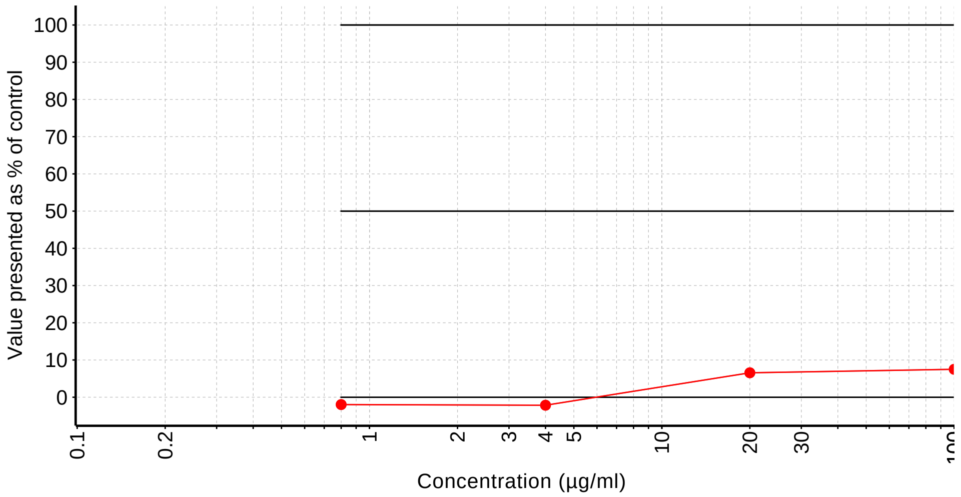

$CC50_{av}$		
EC50	>	100
EC90	>	100
SI_{av}		
SS_{av}		
TI_{av}		
Max %		7.50
[] Max		100
Approved AV		1
Interesting AV		0
Page		58 / 96

Anti-Viral
Anti-Metabolic

Export chart data to CSV

Summary values			
**Statistic**	**CC50**	**EC50**	**EC90**
Median		> 100	> 100
Med.Abs.Dev.			
Mean		> 100	> 100
Stdev.			

Compound	Virus			Cell		AV Method		AM Method	
Primary code	Species	Type	Strain	Type	Subtype	Method	Type	Method	Type
BAVARIE1_0059	Yellow fever virus	No Type	17D Stamaril	Huh	7	Absorbance	MTS - 498nm	Microscopy	Tox scoring

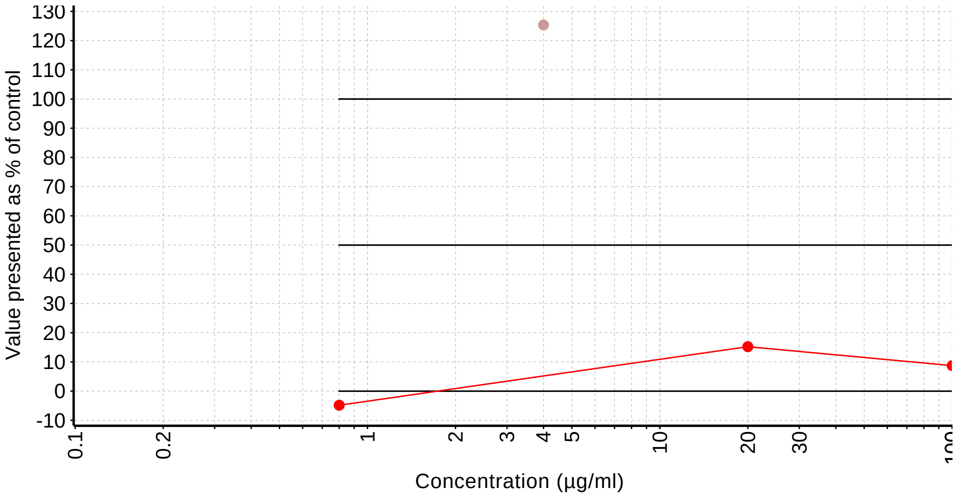

$CC50_{av}$		
EC50	>	100
EC90	>	100
SI_{av}		
SS_{av}		
TI_{av}		
Max %		15.21
[] Max		20
Approved AV		1
Interesting AV		0
Page		59 / 96

Anti-Viral
Anti-Metabolic

Export chart data to CSV

Summary values			
**Statistic**	**CC50**	**EC50**	**EC90**
Median		> 100	> 100
Med.Abs.Dev.			
Mean		> 100	> 100
Stdev.			

Compound	Virus			Cell		AV Method		AM Method	
Primary code	Species	Type	Strain	Type	Subtype	Method	Type	Method	Type
BAVARIE1_0060	Yellow fever virus	No Type	17D Stamaril	Huh	7	Absorbance	MTS - 498nm	Microscopy	Tox scoring

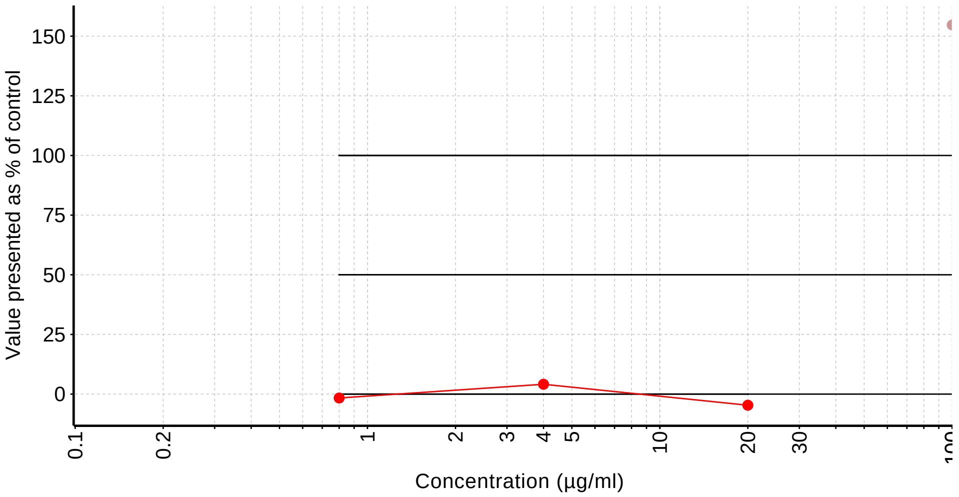

$CC50_{av}$		
EC50	>	20
EC90	>	20
SI_{av}		
SS_{av}		
TI_{av}		
Max %		4.11
[] Max		4
Approved AV		1
Interesting AV		0
Page		60 / 96

Anti-Viral
Anti-Metabolic

Export chart data to CSV

Summary values			
**Statistic**	**CC50**	**EC50**	**EC90**
Median		> 20	> 20
Med.Abs.Dev.			
Mean		> 20	> 20
Stdev.			

Repeated experiments with Yellow fever virus (select)

Compound	Virus			Cell		AV Method		AM Method	
Primary code	Species	Type	Strain	Type	Subtype	Method	Type	Method	Type
BAVARIE1_0001	Yellow fever virus	No Type	17D Stamaril	Huh	7	Absorbance	MTS - 498nm	Microscopy	Tox scoring

Needs more data.

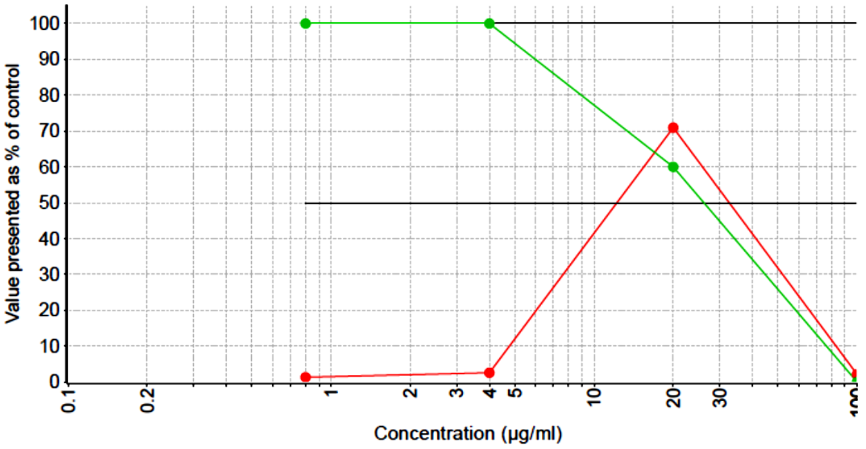

CC50	= 26.2
EC50	= 12.2
EC90	
SI	= 2.14
SS	= 2.27
TI	= 0.75
Max %	64.04
[] Max	18.4
Approved AV	1
Interesting AV	0
Page	1 / 96

Anti-Viral
Anti-Metabolic

Export chart data to CSV

Summary values			
Statistic	CC50	EC50	EC90
Median	= 29.4	= 12.2	
Med.Abs.Dev.	3.25		
Mean	= 29.4	= 12.2	
Stdev.	4.59		

Compound	Virus			Cell		AV Method		AM Method	
Primary code	Species	Type	Strain	Type	Subtype	Method	Type	Method	Type
BAVARIE1_0111	Yellow fever virus	No Type	17D Stamaril	Huh	7	Absorbance	MTS - 498nm	Microscopy	Tox scoring

Needs more data.

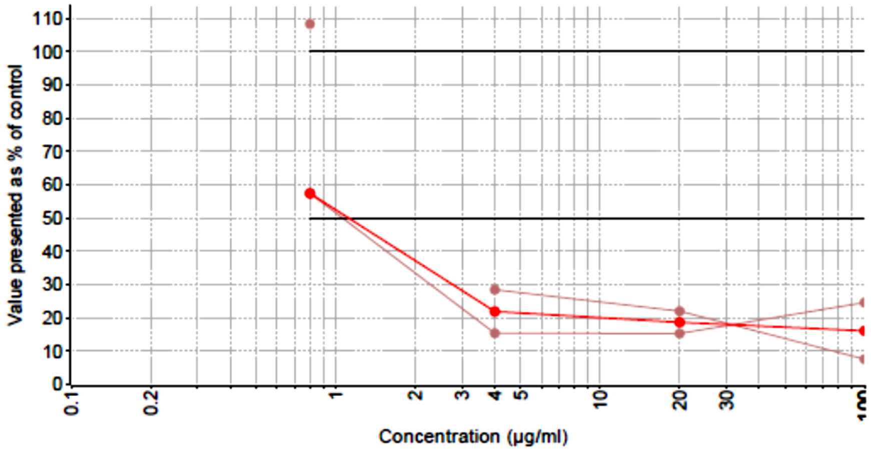

$CC50_{av}$	=	1.12
EC50	<	0.8
EC90	<	0.8
SI_{av}	>	1.4
SS_{av}	>	0.543
TI_{av}	>	0.0795
Max %		57.43
[] Max		0.8
Approved AV		2
Interesting AV		0
Page		4 / 44

Anti-Viral
Anti-Metabolic

Export chart data to CSV

Summary values			
Statistic	CC50	EC50	EC90
Median	= 1.06	< 0.8	< 0.8
Med.Abs.Dev.			
Mean	= 1.06	< 0.8	< 0.8
Stdev.			

Compound	Virus			Cell		AV Method		AM Method	
Primary code	Species	Type	Strain	Type	Subtype	Method	Type	Method	Type
BAVARIE1_0013	Yellow fever virus	No Type	17D Stamaril	Huh	7	Absorbance	MTS - 498nm	Microscopy	Tox scoring

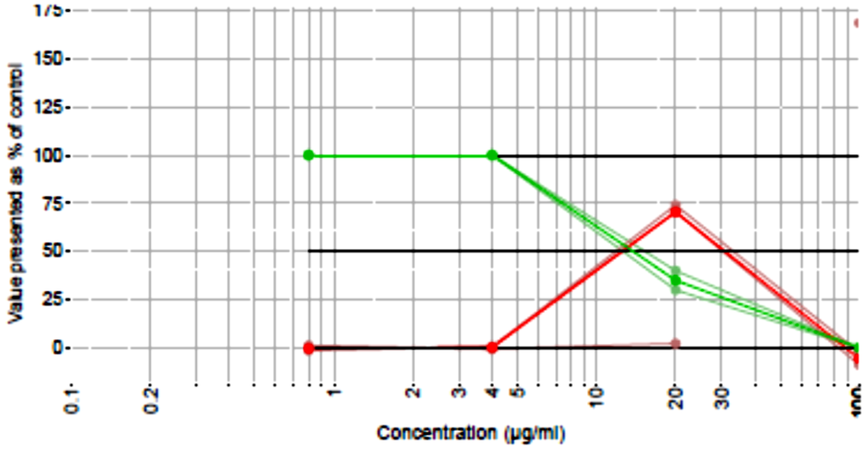

CC50	= 13.8
EC50	= 12.5
EC90	
SI	= 1.1
SS	= 0.358
TI	= 0.0152
Max %	52.05
[] Max	15.8
Approved AV	3
Interesting AV	0
Page	3 / 24

Anti-Viral
Anti-Metabolic

Export chart data to CSV

Summary values			
Statistic	CC50	EC50	EC90
Median	= 22.8	= 12.1	> 20
Med.Abs.Dev.	8.85	0.312	
Mean	= 22.9	= 12.1	> 20
Stdev.	10.4	0.441	

Compound	Virus			Cell		AV Method		AM Method	
Primary code	Species	Type	Strain	Type	Subtype	Method	Type	Method	Type
BAVARIE1_0118	Yellow fever virus	No Type	17D Stamaril	Huh	7	Absorbance	MTS - 498nm	Microscopy	Tox scoring

Needs more data.

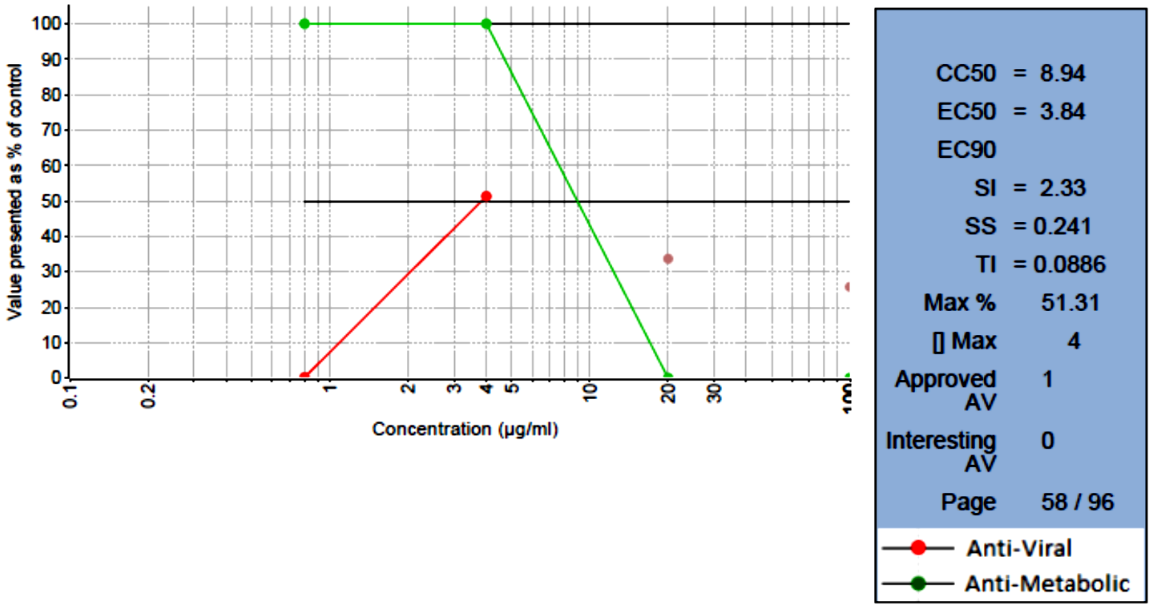

Export chart data to CSV

Summary values			
Statistic	CC50	EC50	EC90
Median	= 8.94	= 3.84	> 4
Med.Abs.Dev.			
Mean	= 8.94	= 3.84	> 4
Stdev.			

Compound	Virus			Cell		AV Method		AM Method	
Primary code	Species	Type	Strain	Type	Subtype	Method	Type	Method	Type
BAVARIE1_0029	Yellow fever virus	No Type	17D Stamaril	Huh	7	Absorbance	MTS - 498nm	Microscopy	Tox scoring

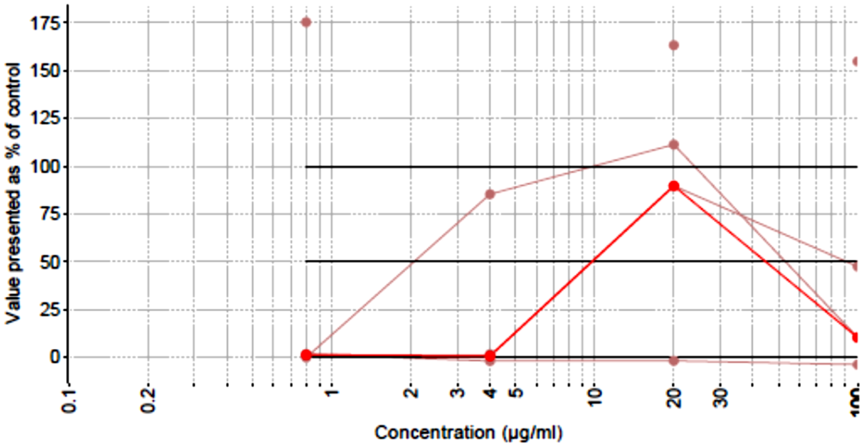

$CC50_{av}$	=	44.7
EC50	=	9.77
EC90		
SI_{av}	=	4.57
SS_{av}	=	13.1
TI_{av}	=	8.64
Max %		89.64
[] Max		20
Approved AV		3
Interesting AV		0
Page		1 / 44

Anti-Viral
Anti-Metabolic

Export chart data to CSV

Summary values			
Statistic	CC50	EC50	EC90
Median	= 71.8	= 5.92	= 5.36
Med.Abs.Dev.	18.8	3.86	
Mean	= 71.8	= 5.92	= 5.36
Stdev.	26.5	5.45	

Compound	Virus			Cell		AV Method		AM Method	
Primary code	Species	Type	Strain	Type	Subtype	Method	Type	Method	Type
BAVARIE1_0030	Yellow fever virus	No Type	17D Stamaril	Huh	7	Absorbance	MTS - 498nm	Microscopy	Tox scoring

Needs more data.

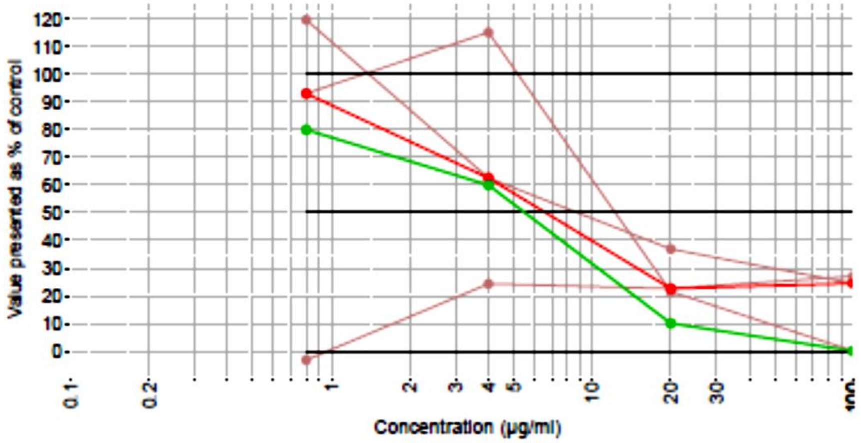

CC50	=	5.52
EC50	<	0.8
EC90	<	0.8
SI	>	6.9
SS	>	14.7
TI	>	12.3
Max %		80.00
[] Max		0.8
Approved AV		3
Interesting AV		0
Page		6 / 24

Anti-Viral
Anti-Metabolic

Export chart data to CSV

Summary values			
Statistic	CC50	EC50	EC90
Median	= 8.79		
Med.Abs.Dev.	3.27		
Mean	= 8.86		
Stdev.	3.37		

Compound	Virus			Cell		AV Method		AM Method	
Primary code	Species	Type	Strain	Type	Subtype	Method	Type	Method	Type
BAVARIE1_0030	Yellow fever virus	No Type	17D Stamaril	Huh	7	Absorbance	MTS - 498nm	Microscopy	Tox scoring

variation is linked to adverse effect of compound on cells

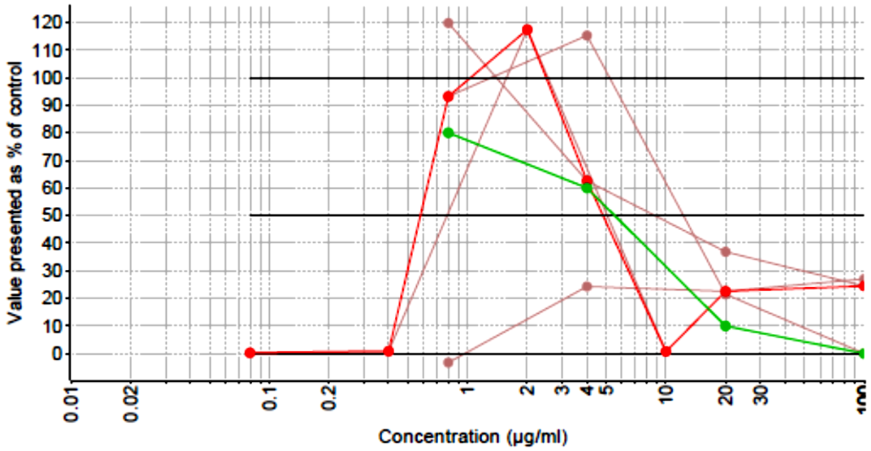

CC50 = 5.77
EC50 = 0.579
EC90
SI = 9.97
SS = 17.1
TI = 17.1
Max % 80.00
[] Max 0.8
Approved AV 4
Interesting AV 0
Page 2 / 44

Anti-Viral
Anti-Metabolic

Export chart data to CSV

Summary values			
Statistic	CC50	EC50	EC90
Median	= 7.15	=0.789	= 1.37
Med.Abs.Dev.	1.86		
Mean	= 7.91	=0.789	= 1.37
Stdev.	3.34		

Compound	Virus			Cell		AV Method		AM Method	
Primary code	Species	Type	Strain	Type	Subtype	Method	Type	Method	Type
BAVARIE1_0048	Yellow fever virus	No Type	17D Stamaril	Huh	7	Absorbance	MTS - 498nm	Microscopy	Tox scoring

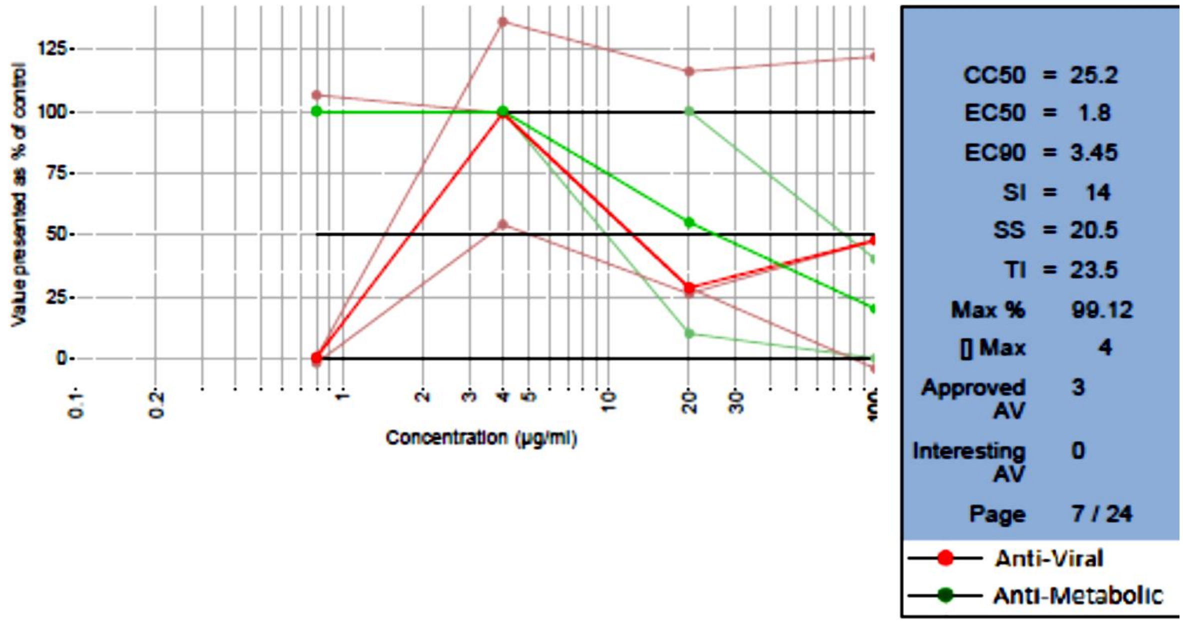

Export chart data to CSV

Summary values			
Statistic	CC50	EC50	EC90
Median	= 11	= 2.5	= 2.31
Med.Abs.Dev.	3.6	1.06	
Mean	= 25.9	= 2.5	= 2.31
Stdev.	33.8	1.49	

Compound	Virus			Cell		AV Method		AM Method	
Primary code	Species	Type	Strain	Type	Subtype	Method	Type	Method	Type
BAVARIE1_0104	Yellow fever virus	No Type	17D Stamaril	Huh	7	Absorbance	MTS - 498nm	Microscopy	Tox scoring

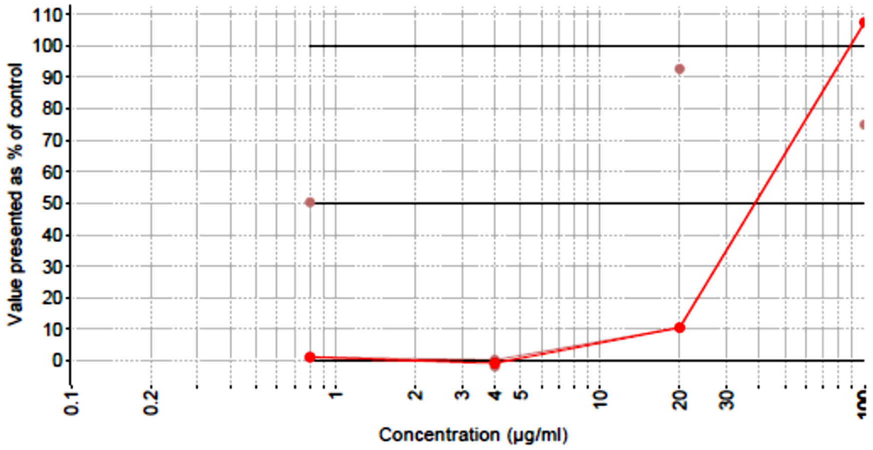

CC50$_{av}$ > 100

EC50 = 38.5

EC90 = 74.8

SI$_{av}$ > 2.6

SS$_{av}$ > 11.9

TI$_{av}$ > 4.93

Max % 107.45

[] Max 100

Approved AV 2

Interesting AV 0

Page 3 / 44

Anti-Viral

Anti-Metabolic

Export chart data to CSV

Summary values			
Statistic	CC50	EC50	EC90
Median	> 100	= 38.5	= 74.8
Med.Abs.Dev.			
Mean	> 100	= 38.5	= 74.8
Stdev.			
