# Supplementary material for: Antiviral and Cytotoxic Activity of Different Plant Parts of Banana (Musa spp.)
Source: Viruses. 2020 May 15;12(5):549. doi: 10.3390/v12050549 (PMC7291111; doi:10.3390/v12050549)
Supplement: Supplementary file 1 [file viruses-12-00549-s001.zip › Supplementary Files~/Supplementary material IV SP.pdf]

Antiviral and Cytotoxic Activity of Different Plant Parts of Banana (*Musa* spp.)

Sujogya Kumar Panda [1,2,*], Ana Hortência Fonsêca Castro [1,3], *Ramin Saleh Jouneghani [1]*, Pieter Leyssen [4], Johan Neyts [4], Rony Swennen [5,6,7] and Walter Luyten [1]

1 Department of Biology, Katholieke Universiteit Leuven, 3000 Leuven, Belgium; acastro905@gmail.com (A.H.F.C.); r.saleh.j@gmail.com (R.S.J.); walter.luyten@kuleuven.be (W.L.)
2 Mayurbhanj Biological Research (MBR), Bhanjpur, Baripada 757002, Odisha, India
3 Plant Physiology and Biochemistry, Universidade Federal de São João Del-Rei, Av. Sebastião Gonçalves Coelho, 400—Chanandour, Divinópolis MG 35501-296, Brazil
4 Rega Institute for Medical Research, Laboratory of Virology and Chemotherapy, Katholieke Universiteit Leuven, 3000 Leuven, Belgium; pieter.leyssen@kuleuven.be (P.L.); johan.neyts@kuleuven.be (J.N.)
5 International Institute of Tropical Agriculture, Arusha P.O. Box 447, Tanzania; rony.swennen@kuleuven.be
6 Laboratory of Tropical Crop Improvement, Division of Crop Biotechnics, Katholieke Universiteit Leuven, 3001 Leuven, Belgium
7 Bioversity International, 3001 Leuven, Belgium
* Correspondence: sujogya.panda@kuleuven.be; Tel.: +32-16-373467

Supplementary material IV

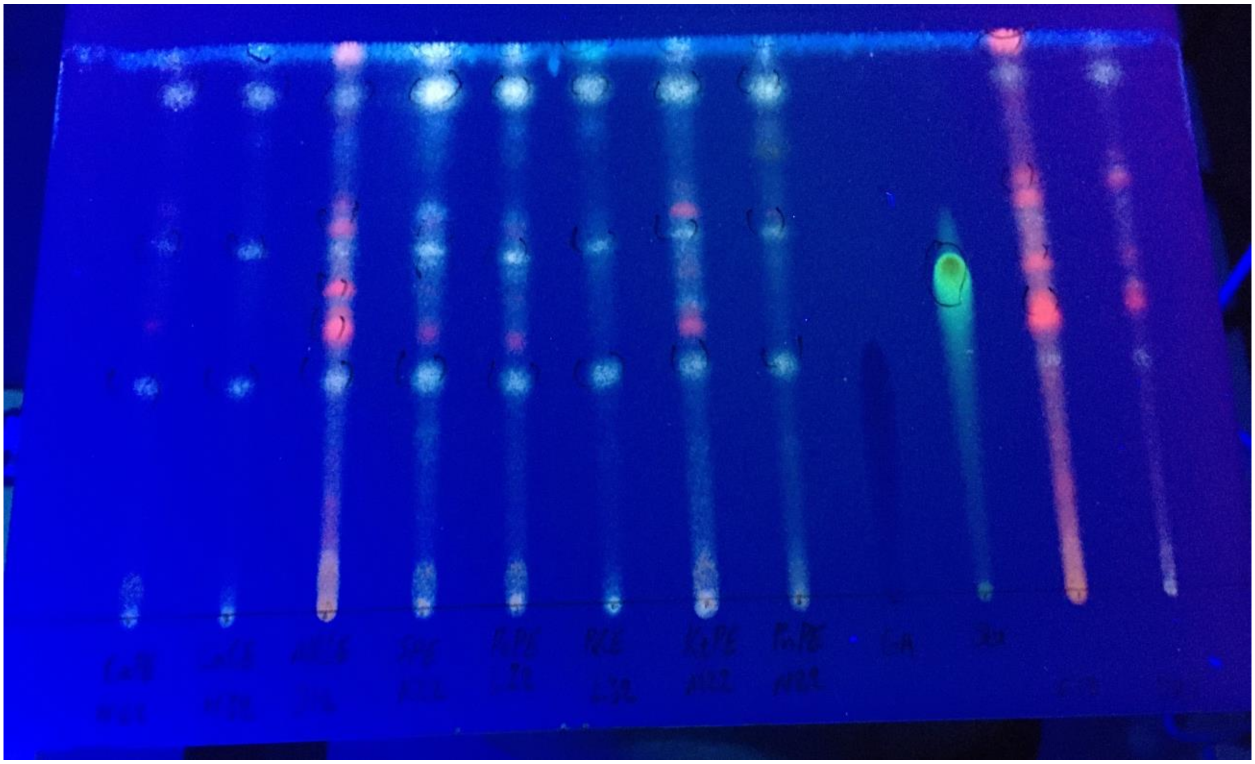

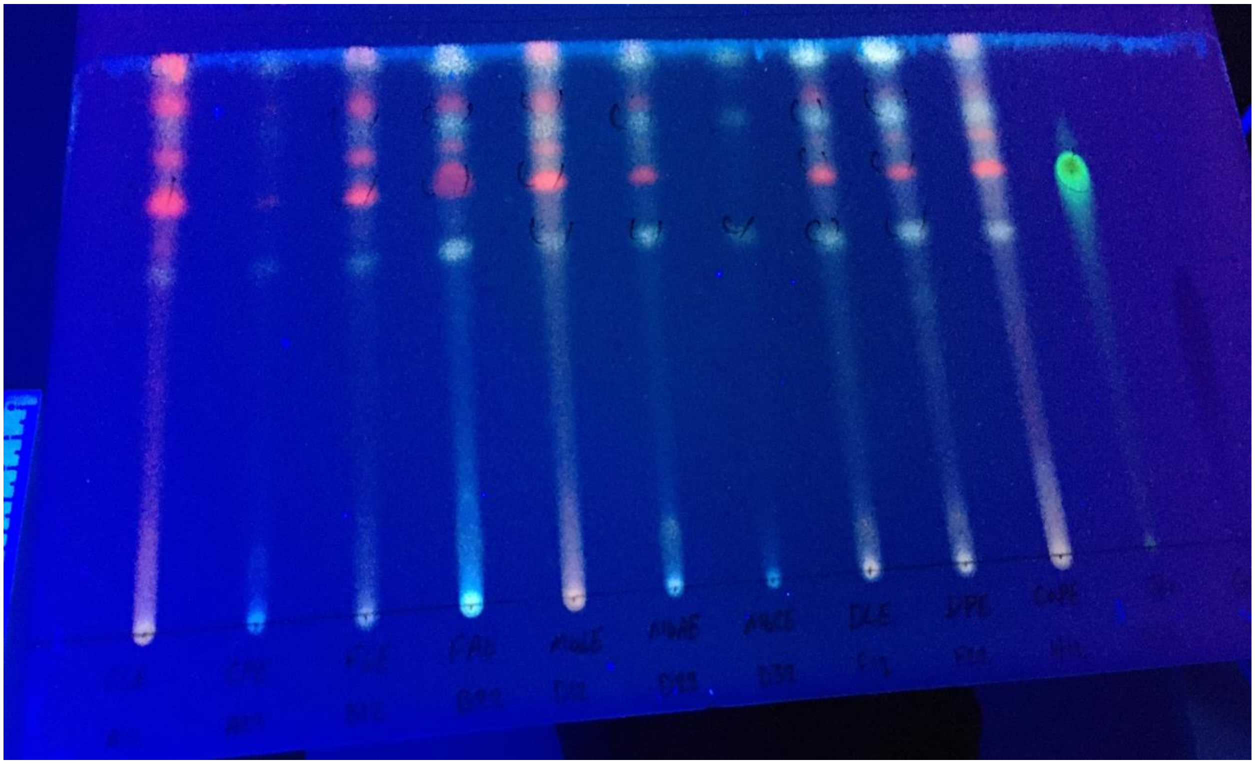

Figure S4 (a): TLC of selected ethanol extracts. Mobile phase-methanol: dichloromethane (1:9. v/v); exposed to UV at 360 nm

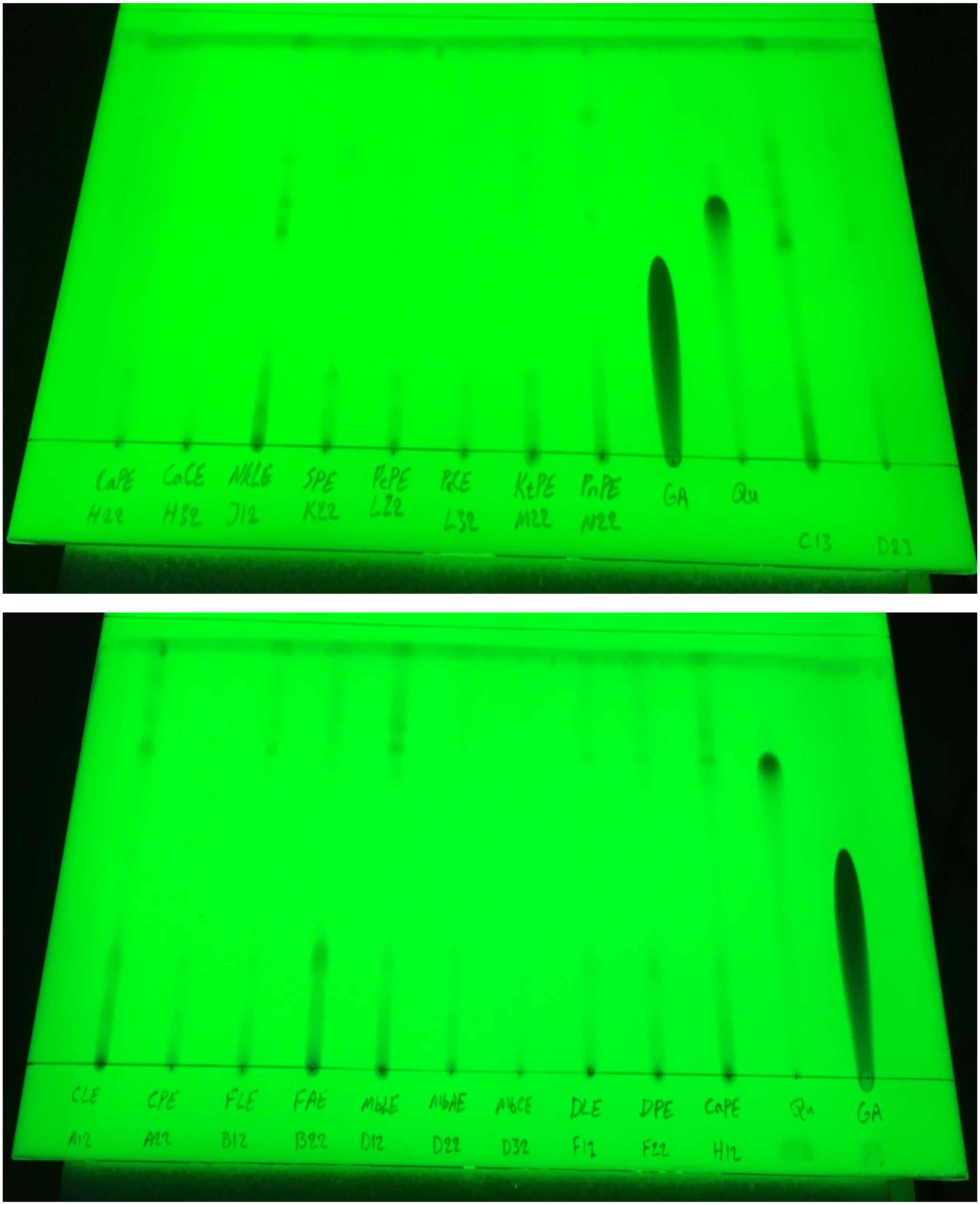

Figure S4 (b): TLC of selected ethanol extracts. Mobile phase-methanol: dichloromethane (1:9. v/v); exposed to UV at 254 nm

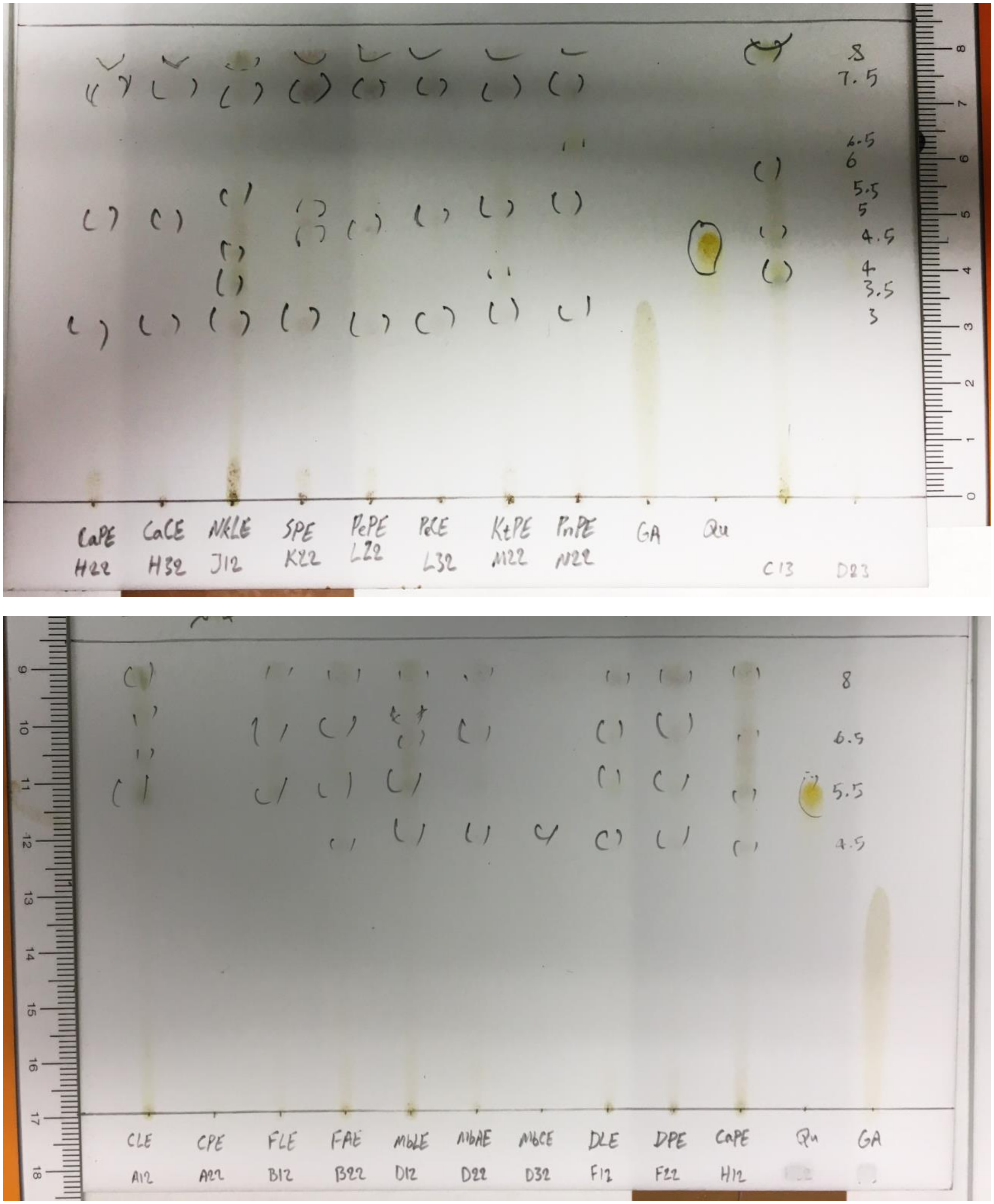

Figure S4 (c): TLC of selected ethanol extracts. Mobile phase-methanol: dichloromethane (1:9. v/v); after spray with 5% sulphuric acid in ethanol

Table S1 (a): Rf values of TLC spots (top panel, ethanol extracts)

Extract code, cultivar name, plant part	Ethanol extracts (top panel). Mobile phase- methanol: dichloromethane (1:9)			
	R_f value in 254 nm	R_f value in 360 nm	Appearance after exposure to 5% sulphuric acid	Possible phytochemical group(s)
CaPE Cachaco Pseudostem	-	0.37-Blue fluorescence	0.37-Light green	Saponin
	-	0.88-Blue fluorescence	0.88-Light green	Saponin
	-	0.95-Blue fluorescence	0.95- Light green	Saponin
CaCE Cachaco Corm	-	0.37- Blue fluorescence	0.37-Light green	Saponin
	-	0.67-Light blue	0.67-Light green	Saponin
	-	0.88- Blue fluorescence	0.88- Light green	Saponin
	-	0.95- Blue fluorescence	0.95-Light green	Saponin
NkLE Namwah khom Leaf	-	0.37-Light blue	0.37- Light green	Saponin
	-	0.47-Pink	0.47-Light green	Phenolics
	0.54-Brown	0.54-Pink	0.54-Light brown	Phenolics
	-	0.64- Blue fluorescence	-	-
	0.75-Brown	0.75-Pink	0.75-Light brown	Phenolics
	-	0.88-Light blue	0.88- Light green	Saponin
	0.95-Brown	0.95-Pink	0.95-Light green	Phenolics
SPE Saba Pseudostem	-	0.37-Light blue	0.37- Light green	Saponin
	-	0.47-Pink	0.47-Light green	Phenolics
	-	0.64- Blue fluorescence	-	-
	-	0.75- Blue fluorescence	0.75-Light brown	Phenolics
	-	0.88-Bright blue	0.88-Brown	Terpenoids
	-	0.95-Bright blue	0.95-Light brown	-
PePE Pelipita Pseudostem	-	0.37-Light blue	0.37- Light green	Saponin
	-	0.47-Pink	0.47-Light green	Phenolics
	-	0.64- Blue fluorescence	-	-
	-	0.88-Bright blue	0.88-Brown	Terpenoids
	0.95-Brown	0.95-Bright blue	0.95-Light brown	-
PeCE Pepipita Corm	-	0.37-Light blue	0.37- Light green	Saponin
	-	0.64- Blue fluorescence	-	-
	-	0.88-Bright blue	0.88-Brown	Terpenoids
	-	0.95-Bright blue	0.95-Light brown	-
KtPE Kluai tiparot Pseudostem	-	0.37-Light blue	0.37- Light green	Saponin
	-	0.47-Pink	0.47-Light green	Phenolics
	-	0.54- Blue fluorescence	-	-
	-	0.64- Blue fluorescence	-	-
	-	0.75-Pink	0.75-Light brown	Phenolics
	-	0.88-Bright blue	0.88-Brown	Terpenoids
	-	0.95-Bright blue	0.95-Light brown	-
PnPE Petit naine Pseudostem	-	0.37-Light blue	0.37- Light green	Saponin
	-	0.64- Blue fluorescence	-	-
	0.88-Brown	0.88-Bright blue	0.88-Brown	Terpenoids
	0.95-Brown	0.95-Bright blue	0.95-Light brown	-

Quercetin	Quercetin	0.68-Brown	0.68- Pink	Phenolics
Gallic acid	Gallic acid	0.37-Brown	0.37-Blue	Phenolics

Table S1 (b): Rf value of TLC spots (bottom panel, ethanol extracts)

**Extract code, cultivar name, plant part**	**Ethanol extracts (bottom panel). Mobile phase- methanol: dichloromethane (1:9)**			
	**R_f value in 254 nm**	**R_f value in 360 nm**	**Appearance after exposure to 5% sulphuric acid**	**Possible phytochemical group(s)**
CLE Cavendish Leaf	-	0.67-Light blue	0.67-Light green	Saponin
	0.75-Brown	0.75-Pink	0.75-Light green	Phenolics
	0.81-Brown	0.81-Pink	0.81-Green	Phenolics
	0.91-Brown	0.91-Pink	0.91-Green	Phenolics
	-	0.95-Pink	0.95-Green	Phenolics
CPE Cavendish Pseudostem	-	0.67-Light blue	-	-
	-	0.95-Light blue	0.95-Light green	Saponin
FLE Fougamou Leaf	-	0.67-Light blue	0.67-Light green	Saponin
	0.75-Brown	0.75-Pink	0.75-Light green	Phenolics
	-	0.81-Pink	0.81-Green	Phenolics
	-	0.91-Pink	0.91-Green	Phenolics
	-	0.95-Light pink	0.95-Green	Phenolics
FPE Fougamou Pseudostem	-	0.67-Light blue	0.67-Light green	Saponin
		0.75-Pink	0.75-Light green	Phenolics
		0.81-Pink	0.81-Green	Phenolics
		0.82-Blue	-	-
		0.91-Pink	0.91-Green	Phenolics
	-	0.95- Blue fluorescence	0.95-Green	-
MbLE Mbwazirume Leaf	0.67-Brown	0.67-Blue	0.67-Light green	Saponin
	0.75-Brown	0.75-Pink	0.75-Light green	Phenolics
	0.81- Brown	0.81-Pink	0.81-Green	Phenolics
	-	0.82-Pale yellow	-	-
	0.91- Brown	0.91-Pink	0.91-Green	Phenolics
	-	0.95- Pink	0.95-Green	-
MbPE Mbwazirume Pseudostem	-	0.67-Blue	0.67-Light green	Saponin
	-	0.75-Pink	0.75-Light green	Phenolics
	-	0.91-Blue	0.91-Green	Saponin
	-	0.95-Bright blue	0.95-Brown	Terpenoids
MbCE Mbwazirume Corm	-	0.67-Blue	0.67-Light green	Saponin
	-	0.91-Blue	-	-
	-	0.95-Bright blue	-	-
DLE Dole Leaf	-	0.67-Blue	0.67-Light green	Saponin
	-	0.75-Pink	0.75-Light green	Phenolics
	-	0.91-Blue	0.91-Green	Saponin
	-	0.95-Bright blue	0.95-Brown	Terpenoids
DPE	-	0.67-Blue	0.67-Light green	Saponin

Dole Pseudostem	-	0.75-Pink	0.75-Light green	Phenolics
	-	0.91-Blue	0.91-Green	Saponin
	-	0.95-Bright blue	0.95-Brown	Terpenoids
CaPE Cachaco Pseudostem	0.67-Brown	0.67-Blue	0.67-Light green	Saponin
	0.75-Brown	0.75-Pink	0.75-Light green	Phenolics
	0.81-Brown	0.81-Pink	0.81-Green	Phenolics
	-	0.82-Pale yellow	-	-
	0.91-Brown	0.91-Pink	0.91-Green	Phenolics
Quercetin	0.68-Brown	0.68- Pink	0.68-Green	Phenolics
Gallic acid	0.37-Brown	0.37-Blue	0.37-Light brown	Phenolics

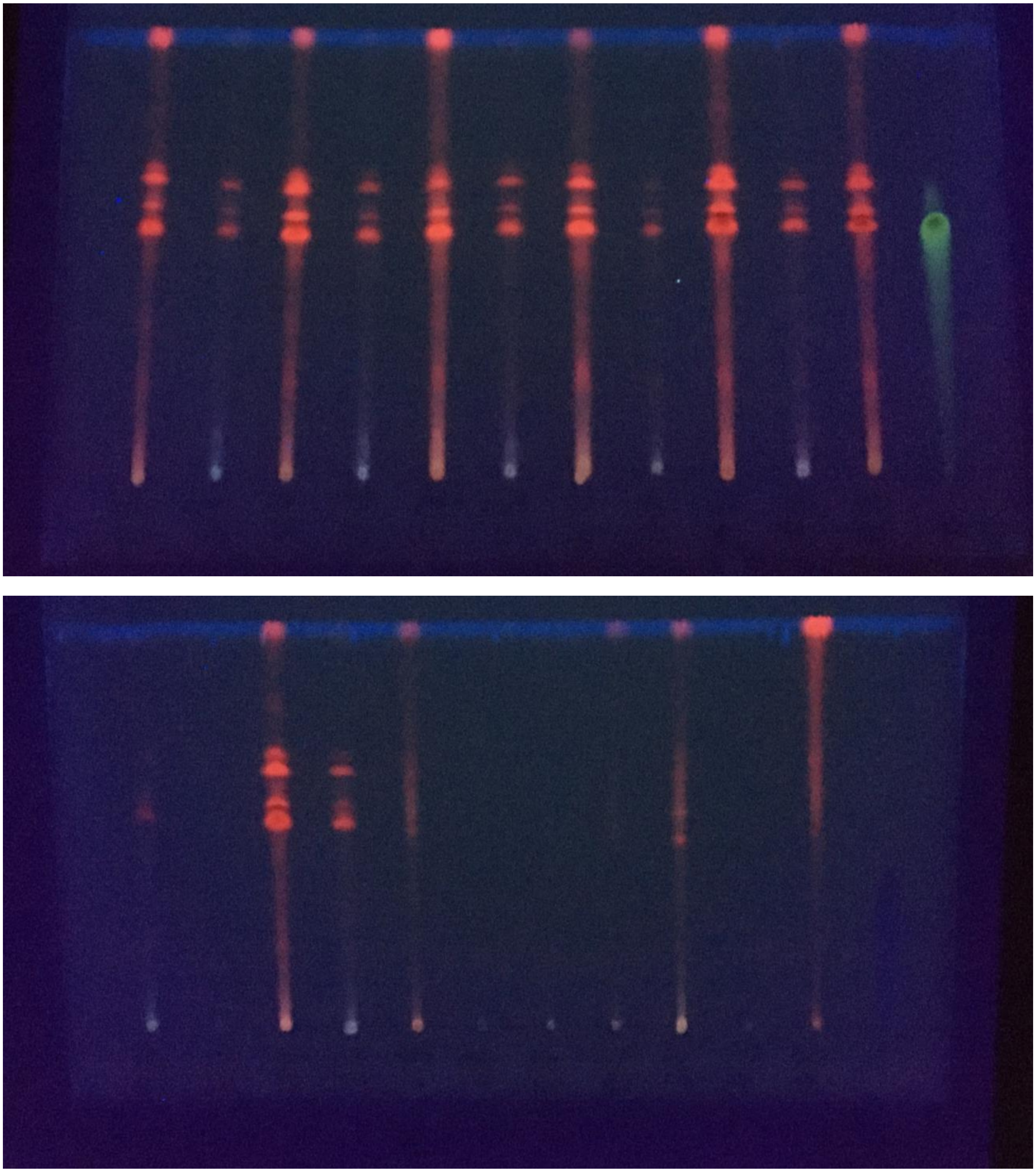

Figure S4 (d): TLC of selected acetone and hexane extracts. Mobile phase-hexane: ethyl acetate (5:5, v/v); exposed to UV at 360 nm

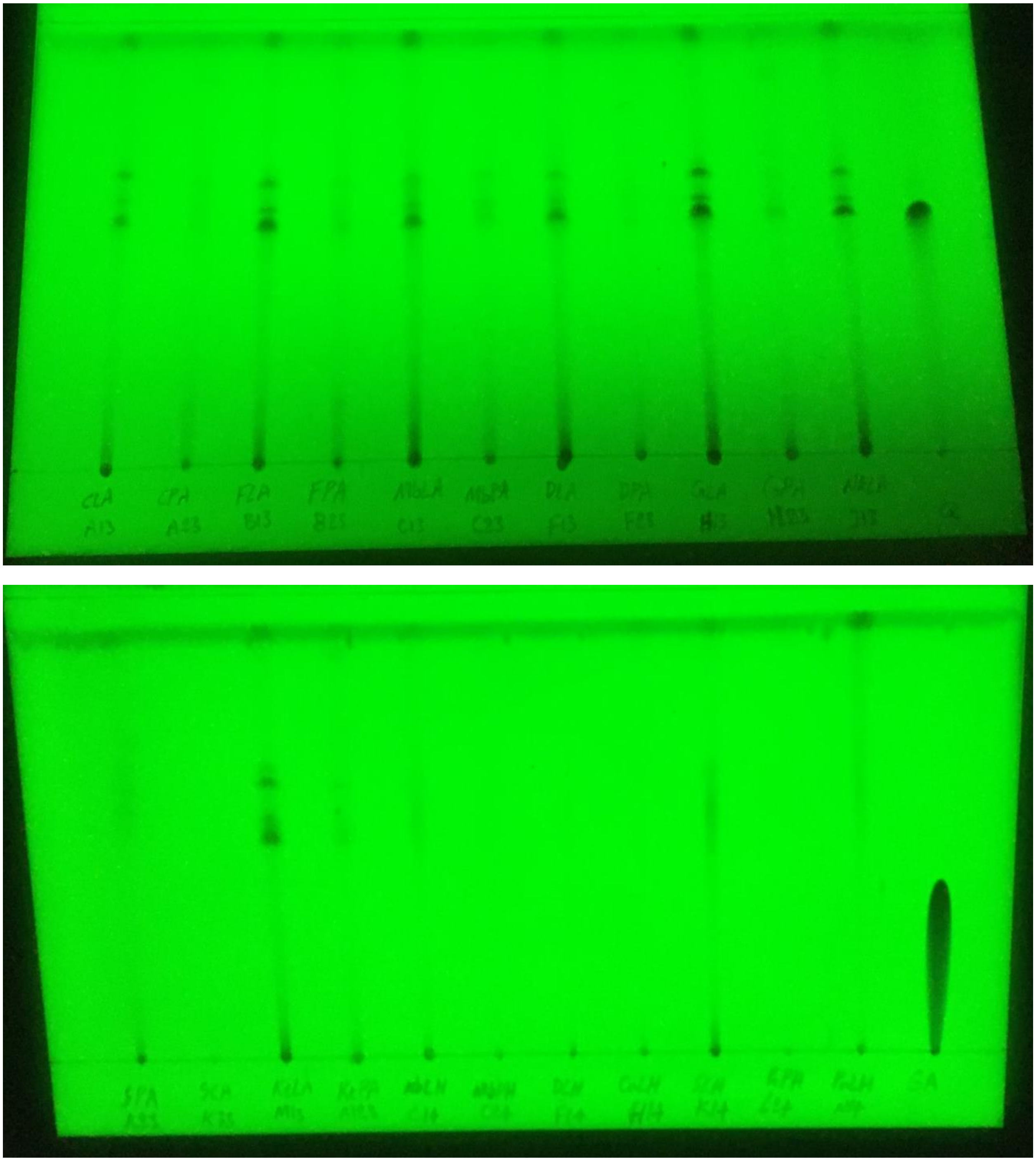

Figure S4 (e): TLC of selected acetone and hexane extracts. Mobile phase-hexane: ethyl-acetate (5:5, v/v); exposed to UV at 254 nm

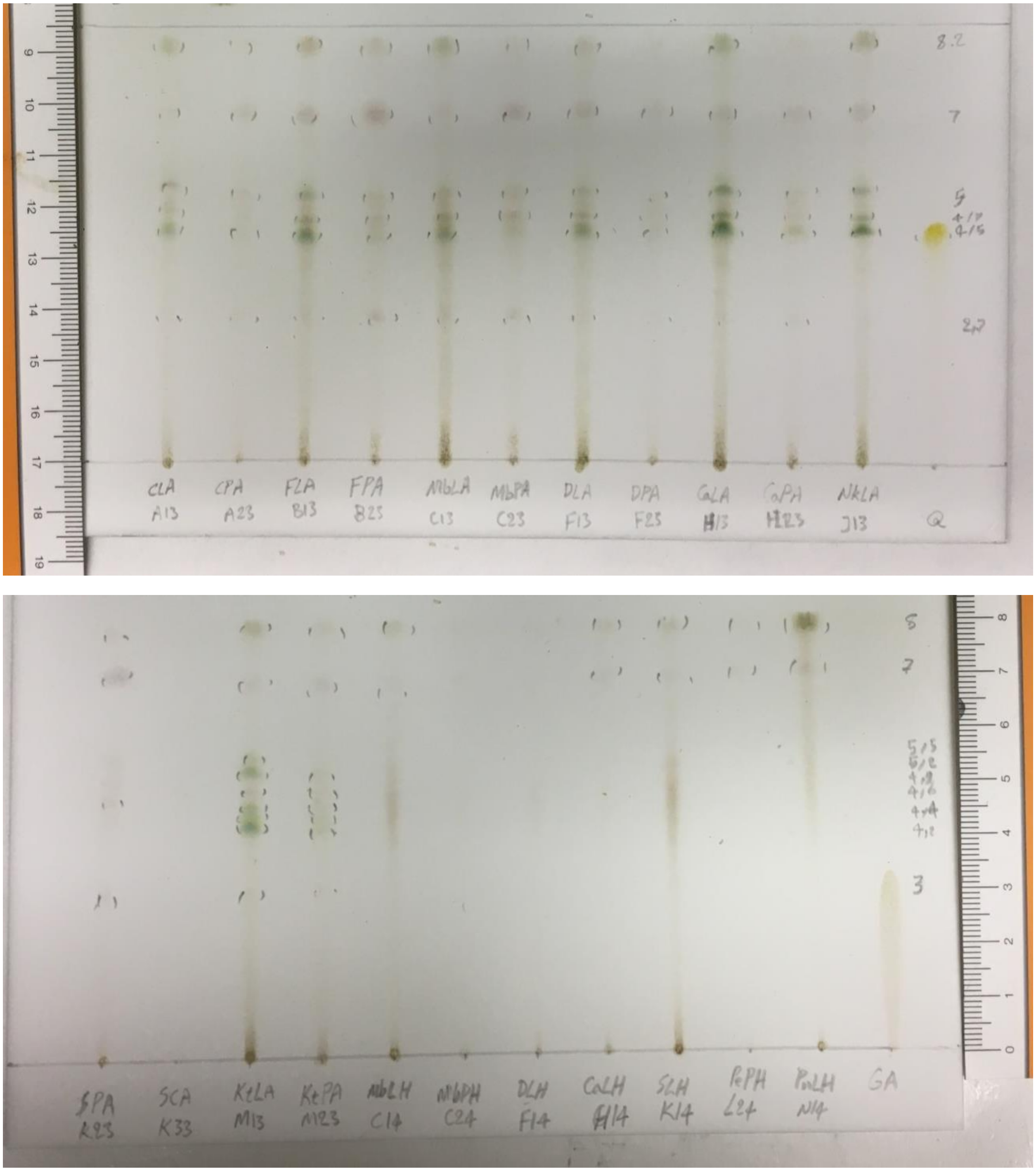

Figure S4 (f): TLC of selected acetone and hexane extracts. Mobile phase-hexane: ethyl acetate (5:5, v/v); after spraying with 5% sulphuric acid in ethanol

Table S1 (c): Rf value of TLC spots (acetone extracts, top panel)

**Extract code, cultivar name, part(s)**	**Acetone extracts (top panel). Mobile phase- hexane: ethyl acetate (5:5. v/v)**			
	**R_f value in 254 nm**	**R_f value in 360 nm**	**Appearance after exposure to 5% sulphuric acid**	**Possible phytochemical group(s)**
CLA Cavendish Leaf	0.54-Brown	0.54-Pink	0.54-Green	Phenolics
	0.61-Light brown	0.61-Pink	0.61-Green	Phenolics
	0.67- Brown	0.67-Pink	0.67-Green	Phenolics
	-	-	0.87-Light brown	-
	0.95- Brown	0.95-Pink	0.95-Green	Phenolics
CPA Cavendish Pseudostem	0.54-Brown	0.54-Light pink	0.54-Green	Phenolics
	0.61-Light brown	-	0.61-Green	-
	0.67- Brown	0.67- Light pink	0.67-Green	Phenolics
	-	-	0.87-Light brown	-
FLA Fougamou Leaf	0.54-Brown	0.54-Pink	0.54-Dark green	Phenolics
	0.61-Light brown	0.61-Pink	0.61-Green	Phenolics
	0.67- Brown	0.67-Pink	0.67-Green	Phenolics
	-	-	0.87-Light brown	-
	0.95- Brown	0.95-Pink	0.95-Green	Phenolics
FPA Fougamou Pseudostem	-	-	0.31-Light brown	
	0.54-Light brown	0.54-Light pink	0.54-Light green	Phenolics
	0.61-Light brown	-	0.61-Light green	-
	0.67- Brown	0.67- Light pink	0.67-Light green	Phenolics
	-	-	0.87-Light brown	-
	-	-	0.95-Light brown	-
MbLA Mbwazirume Leaf	0.54-Brown	0.54-Pink	0.54-Dark green	Phenolics
	0.61-Light brown	0.61-Pink	0.61-Green	Phenolics
	0.67- Brown	0.67-Pink	0.67-Green	Phenolics
	-	-	0.87-Light brown	-
	0.95- Brown	0.95-Pink	0.95-Green	Phenolics
MbPA Mbwazirume Pseudostem	-	-	0.31-Light brown	
	0.54-Light brown	0.54-Light pink	0.54-Light green	Phenolics
	0.61-Light brown	-	0.61-Light green	-
	0.67- Brown	0.67- Light pink	0.67-Light green	Phenolics
	-	-	0.87-Light brown	-
	-	-	0.95-Light brown	-
DLA Dole Leaf	0.54-Brown	0.54-Pink	0.54-Dark green	Phenolics
	0.61-Light brown	0.61-Pink	0.61-Green	Phenolics
	0.67- Brown	0.67-Pink	0.67-Green	Phenolics
	-	-	0.87-Light brown	-
	0.95- Brown	0.95-Pink	0.95-Green	Phenolics
DPA Dole Pseudostem	0.54-Brown	0.54-Light pink	0.54-Green	Phenolics
	0.61-Light brown	-	0.61-Green	-
	0.67- Brown	-	0.67-Green	Phenolics
CaLA	0.54-Brown	0.54-Pink	0.54-Dark green	Phenolics

Cachaco<br>Leaf	0.61-Light brown	0.61-Pink	0.61-Green	Phenolics
	0.67- Brown	0.67-Pink	0.67-Green	Phenolics
	-	-	0.87-Light brown	-
	0.95- Brown	0.95-Pink	0.95-Green	Phenolics
CaPA<br>Cachaco<br>Pseudostem	0.54-Brown	0.54-Light pink	0.54-Green	Phenolics
	0.61-Light brown	-	0.61-Green	-
	0.67- Brown	0.67- Light pink	0.67-Green	Phenolics
	-		0.87-Light brown	-
NkLA<br>Namwah khom<br>Leaf	0.54-Brown	0.54-Pink	0.54-Dark green	Phenolics
	0.61-Light brown	0.61-Pink	0.61-Green	Phenolics
	0.67- Brown	0.67-Pink	0.67-Green	Phenolics
	-	-	0.87-Light brown	-
	0.95- Brown	0.95-Pink	0.95-Green	Phenolics
Quercetin	0.54-Dark	0.54-Green	0.54-Yellow	Phenolics

Table S1 (d): Rf value of TLC spots (acetone and hexane extracts, bottom panel)

**Extract code, cultivar name, part(s)**	**Acetone and hexane extracts (bottom panel). Mobile phase- hexane: ethyl acetate (5:5. v/v)**			
	**R_f value in 254 nm**	**R_f value in 360 nm**	**Appearance after exposure to 5% sulphuric acid**	**Possible phytochemical group(s)**
SPA<br>Saba<br>Pseudostem	-	-	0.57-Light brown	-
	-	0.61-Light pink	0.61-Light brown	Phenolics
	-	-	0.87-Light brown	-
	-	-	0.94-Light brown	-
SCA<br>Saba<br>Corm	-	-	-	-
	-	-	-	-
	-	-	-	-
KtLA<br>Kluai tiparot<br>Leaf	-	-	0.31-Light brown	-
	-	-	0.51-Dark green	-
	0.57-Derak brown	0.57-Pink	0.57-Light green	Phenolics
	0.61-Light brown	0.61- Pink	0.61-Light brown	-
	0.65-Lightbrown	0.65- Pink	0.65-Dark green	Phenolics
	-	0.67-Pink	0.67-Light green	Phenolics
	-	-	0.87-Light brown	-
	0.94-Dak brown	0.94-Pink	0.94-Light green	-
KtPA<br>Kluai tiparot<br>Pseudostem	-	-	0.51-Dark green	-
	0.57-Derak brown	0.57-Pink	0.57-Light green	Phenolics
	0.61-Light brown	0.61- Pink	0.61-Light brown	-
	0.65-Lightbrown	0.65- Pink	0.65-Dark green	Phenolics
	-	-	0.87-Light brown	-
	-	-	0.94-Light green	-
MbLH	0.61-Dark brown	0.61-Light pink		Phenolics

Mbwazirume	-	-	0.87-Light brown	-
Leaf	0.94-Light green	0.94-Light pink	0.94-Light green	Phenolics
MbPh	-	-	-	-
Mbwazirume	-	-	-	-
Pseudostem	-	-	-	-
DLH	-	-	0.87-Light brown	-
Dole	-	-	0.94-Light green	-
Leaf	-	-	-	-
CaLH	-	-	0.87-Light brown	-
Cachaco	-	0.94-Light pink	0.94-Light green	Phenolics
Leaf	-	-	-	-
SLH	0.61-Dark brown	0.61-Light pink	-	Phenolics
Saba	-	-	0.87-Light brown	-
Leaf	0.94-Light green	0.94-Light pink	0.94-Light green	Phenolics
PePH	-	-	0.87-Light brown	-
Pelipita	-	-	0.94-Light green	-
Pseudostem	-	-	-	-
PnLH	-	-	0.87-Light brown	-
Petit naine	0.94-Light green	0.94-Pink	0.94-Light green	Phenolics
Leaf	-	-	-	-
Gallic acid	0.37-Dark	0.37-Blue	0.37-Light brown	Phenolic

Table S1 (e): Total phenolic content (gallic acid equivalents, μg/mg) of different extracts

Name	Leaf				Pseudostem				Corm	Average	St Dev
	A	W	E	H	A	W	E	H	E		
Dole	185	ND	150	147	160	ND	140	ND	ND	157	18
Cachaco	246	ND	164	181	110	ND	150	ND	103	159	53
Saba	ND	ND	ND	149	180	ND	ND	ND	ND	165	22
Kluai Tiparot	184	ND	ND	ND	152	ND	170	ND	ND	169	16
Pelipita	ND	ND	ND	ND	ND	ND	111	73	168	117	48
Namwah Khom	ND	187	168	ND	ND	ND	ND	ND	ND	178	13
Fougamou	147	ND	24	ND	ND	129	309	ND	ND	152	118
Petite naine	ND	ND	ND	151	ND	ND	205	ND	ND	178	38
Giant Cavendish	213	ND	236	ND	77	ND	43	ND	ND	142	97
Mbwazirume	178	ND	187	154	136	ND	176	73	ND	151	42

Note: The phenolic concentration of extracts was evaluated from a gallic acid calibration curve (shown below), and data are expressed in gallic acid equivalents (GAE) as μg/mg of the crude extract. A- Acetone, W-Water, E-Ethanol, H-Hexane, ND-Not determined (because extract showed little antiviral activity, or insufficient sample was left after bioactivity testing).

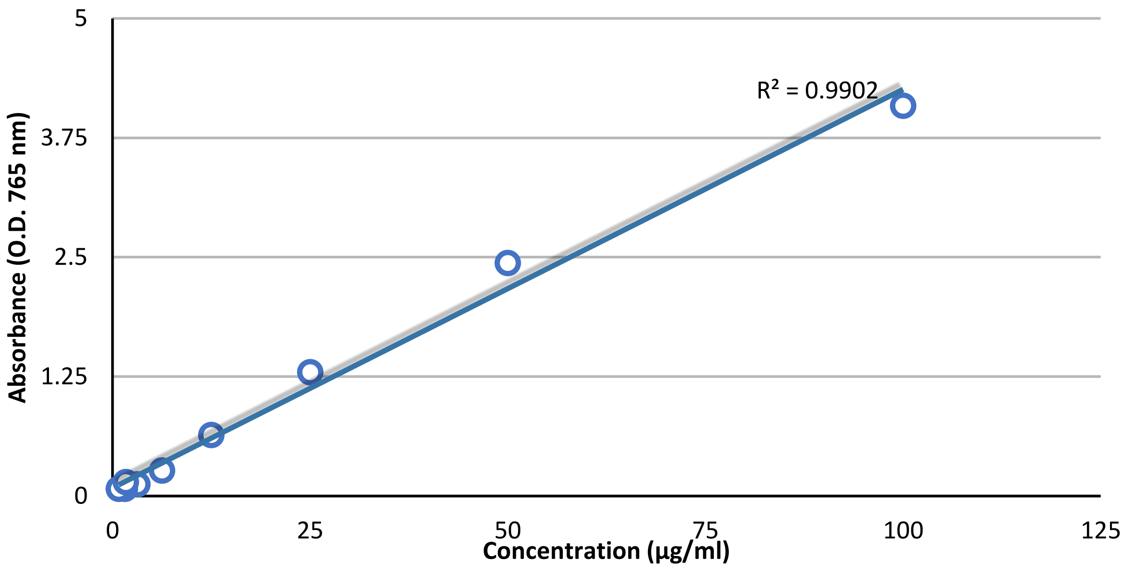

Figure S4 (g): Regression curve for gallic acid assayed with Folin–Ciocalteu reagent (source: https://doi.org/10.3390/foods9040435).

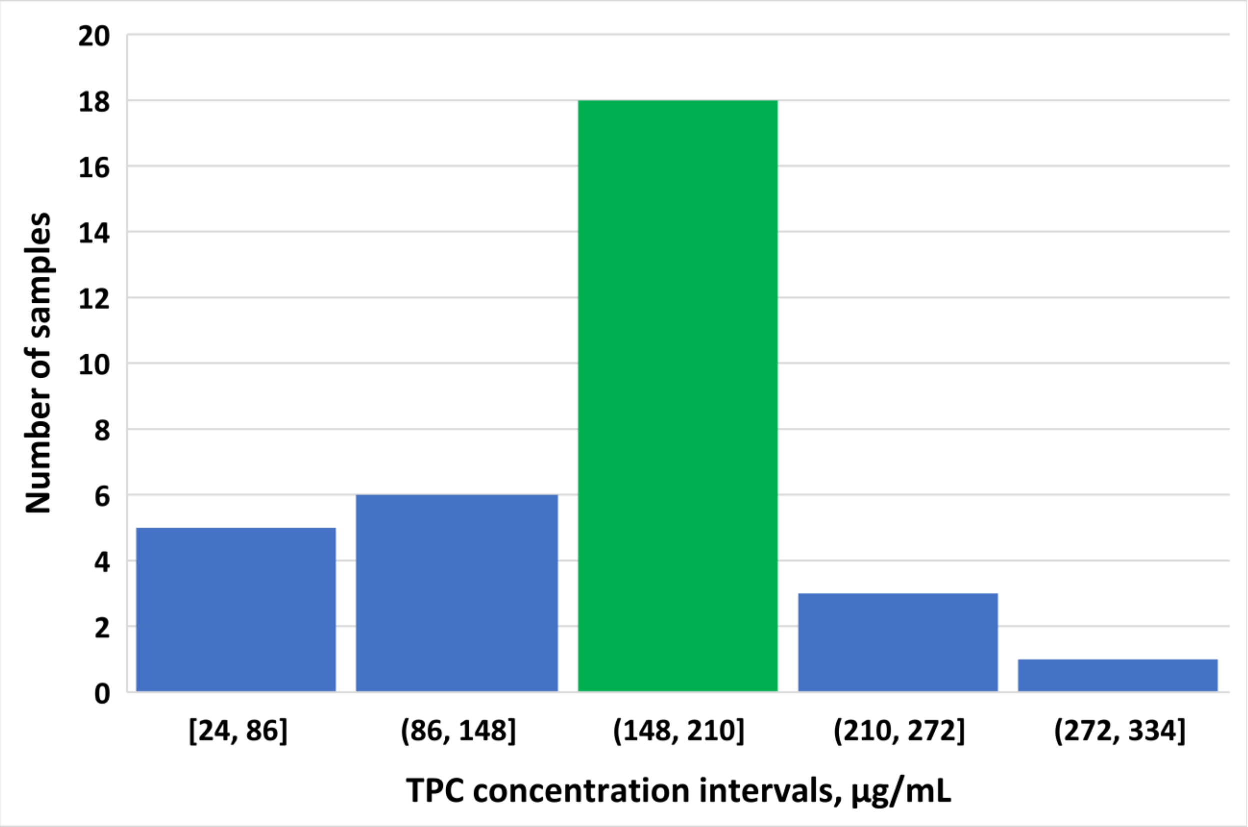

Figure S4 (h): Distribution of total phenolic content across all samples analysed.
